# Antihypertensive Treatment and Risk of Metabolic Associated Fatty Liver Disease: A Drug-Target Mendelian Randomisation

**DOI:** 10.1161/HYPERTENSIONAHA.126.27526

**Published:** 2026-09-18

**Authors:** Yifan Hu, Karl Smith-Byrne, Zeinab Bidel, Xiong Xiao, Xing Zhao, Abbas Dehghan, Dexter Canoy, Malgorzata Wamil, Nathalie Conrad, Shishir Rao, Ben Omega Petrazzini, Qianqian Yang, Dipender Gill, Venexia M Walker, Charalampos Sigalas, Reza Malekzadeh, Johan Sundström, Mark Woodward, George Davey Smith, Kazem Rahimi, Milad Nazarzadeh

**Affiliations:** 1 Sichuan Provincial People’s Hospital, University of Electronic Science and Technology of China, Chengdu, China; 2 Deep Medicine, Nuffield Department of Women’s and Reproductive Health, Medical Science Division, University of Oxford, Oxford, UK; 3 Department of Epidemiology and Health Statistics, West China School of Public Health, Sichuan University, Chengdu, China; 4 Cancer Epidemiology Unit, Nuffield Department of Population Health, University of Oxford, Oxford, UK; 5 Department of Epidemiology and Biostatistics, Imperial College London, London, UK; 6 Population Health Sciences Institute, Newcastle University, Newcastle, UK; 7 Cardiology Department, Great Western Hospital NHS Trust, Swindon, UK; 8 Cardiology Department, Mayo Clinic Healthcare in London, London, UK; 9 Department of Cardiovascular Sciences, KU Leuven, Leuven, Belgium; 10 Medical Research Council Integrative Epidemiology Unit, University of Bristol, Bristol, UK; 11 Population Health Sciences, Bristol Medical School, University of Bristol, Bristol, UK; 12 Department of Surgery, University of Pennsylvania Perelman School of Medicine, Philadelphia, USA; 13 Department of Pharmacology, University of Oxford, Oxford, UK; 14 Liver and Pancreatobiliary Diseases Research Center, Digestive Diseases Research Institute, Tehran University of Medical Sciences, Tehran, Iran; 15 Department of Medical Sciences, Uppsala University, Sweden; 16 The George Institute for Global Health, University of New South Wales, Sydney, Australia; 17 The George Institute for Global Health, School of Public Health, Imperial College London, London, UK

**Keywords:** antihypertensive treatment, blood pressure, drug repurposing, metabolic dysfunction–associated fatty liver disease, Mendelian randomisation, non-alcoholic fatty liver disease, renin–angiotensin system

## Abstract

**Background:**

Metabolic dysfunction–associated fatty liver disease, conventionally known as non-alcoholic fatty liver disease (NAFLD), affects over a quarter of the global population and lacks approved pharmacological treatments. We investigated whether elevated blood pressure (BP) is a modifiable risk factor for NAFLD and whether antihypertensive drugs could be repurposed for prevention.

**Methods:**

We conducted whole-genome Mendelian randomisation (MR) using genetic data from over one million individuals of European ancestry to assess systolic BP as a risk factor for NAFLD. Drug-target MR was used to investigate the class-specific effects of antihypertensive agents, including renin–angiotensin system (RAS) inhibitors, beta-blockers, calcium channel blockers, and diuretics. NAFLD was defined by persistently elevated alanine aminotransferase levels after excluding alternative liver diseases and alcohol use disorders. Genetic estimates were compared with individual-participant meta-analyses of randomised controlled trials (n = 358,636) and summary data from 104 antihypertensive drug trials (~500,000 participants), using coronary heart disease (CHD) as a positive control.

**Results:**

A genetically proxied 5-mmHg reduction in systolic BP was associated with an 8% lower risk of NAFLD (OR 0.92; 95% CI, 0.90–0.94). RAS inhibition was associated with a 27% lower risk (OR 0.73; 95% CI, 0.62–0.85), whereas other drug classes showed no substantial associations. For CHD, effects were directionally consistent across classes in genetic and trial analyses.

**Conclusion:**

Genetically lower systolic BP is associated with reduced NAFLD risk, with RAS inhibition showing the most favourable association. These findings support BP lowering, particularly RAS inhibition, as a potential preventive strategy warranting dedicated randomised trials.

## Graphical abstract

**Graphical Abstract:**
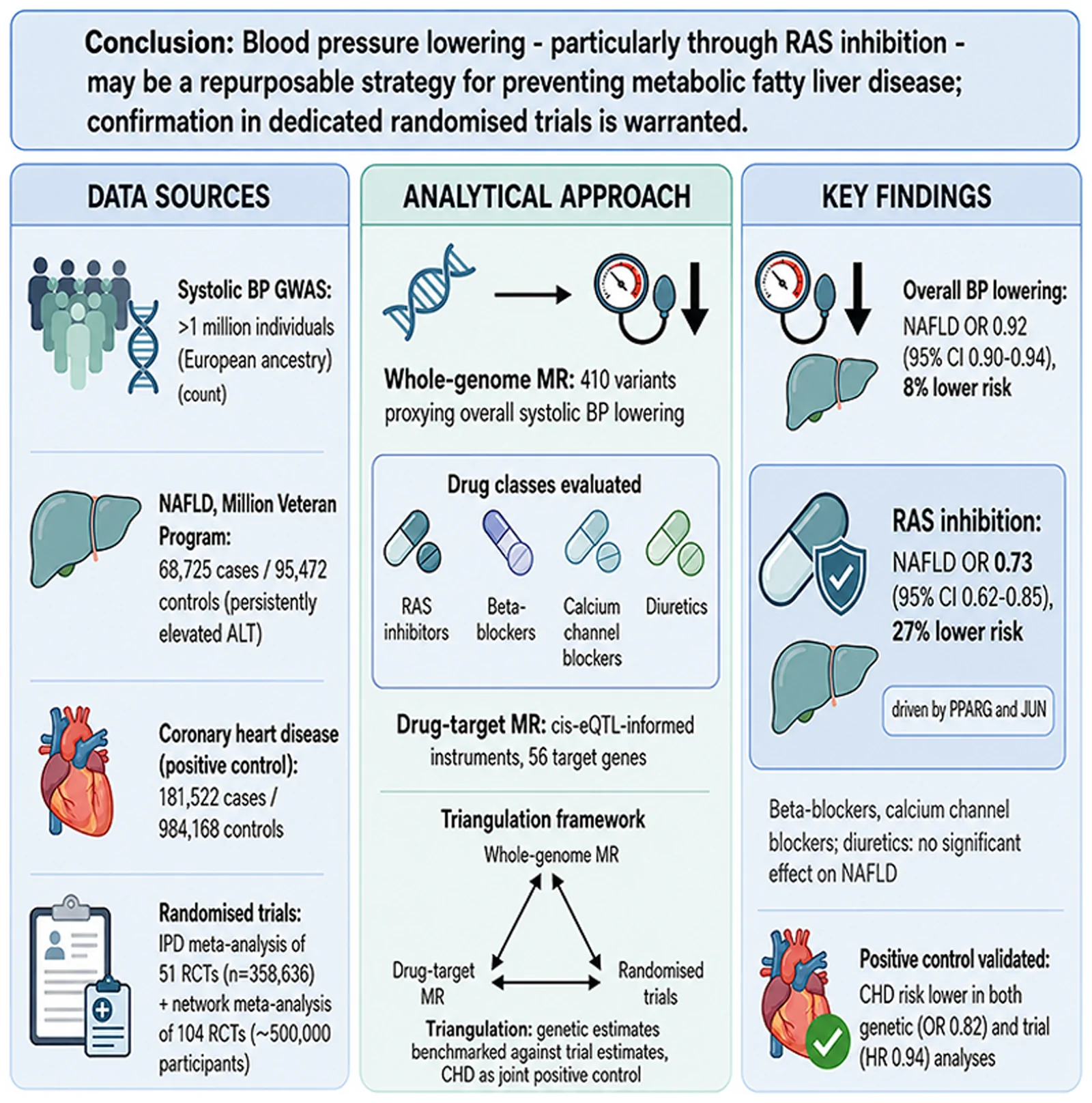

## Nonstandard Abbreviations and Acronyms

ALT: alanine aminotransferase
BP: blood pressure
BPLTTC: Blood Pressure Lowering Treatment Trialists’ Collaboration
CHD: coronary heart disease
cis-eQTL: cis-expression quantitative trait locus
eQTL: expression quantitative trait locus
GTEx: Genotype-Tissue Expression Project
GWAS: genome-wide association study
IPD: individual participant data
IVW: inverse-variance weighted
LD: linkage disequilibrium
MAF: minor allele frequency
MAFLD: metabolic dysfunction–associated fatty liver disease
MR: Mendelian randomisation
NAFLD: non-alcoholic fatty liver disease
RAS: renin–angiotensin system
RCT: randomized controlled trial
SBP: systolic blood pressure

## Introduction

Non-alcoholic fatty liver disease (NAFLD), now frequently termed metabolic dysfunction–associated fatty liver disease (MAFLD), is the most prevalent chronic hepatic disorder, affecting approximately 32% of the global population.^1^ It is the primary cause of cirrhosis, liver cancer, and liver transplantation and is also linked to several non-hepatic conditions, such as cardiovascular diseases.^2,3^ Pharmacological treatment options for NAFLD remain limited, with only thiazolidinediones, a glucose-lowering drug class, being recommended in clinical guidelines for advanced liver fibrosis.^4–8^ However, the use of thiazolidinediones plummeted after 2007 due to safety concerns regarding a potential increase in the risk of ischaemic myocardial events^9,10^ and is also contraindicated in patients with certain cardiovascular conditions, particularly heart failure.^11–13^

NAFLD shares several metabolic risk factors with cardiovascular diseases, particularly elevated blood pressure (BP),^14^ which is recognised as the most important modifiable risk factor and the leading contributor to cardiovascular diseases and death worldwide, for which effective and affordable pharmacological treatments are readily available.^15^ Observational evidence indicates that hypertension increases the risk of NAFLD by up to 63%.^16^ Given the close pathophysiological link between elevated BP and NAFLD,^17–19^ the substantial co-occurrence of these conditions,^11–13^ and the well-established beneficial effects of antihypertensive medications on a wide range of cardiovascular and cardiometabolic diseases,^20–23^ we hypothesised that elevated BP could be a modifiable risk factor for NAFLD and that antihypertensive drugs could be repurposed for its treatment and prevention. Despite the high prevalence of both conditions, the widespread availability of antihypertensive medications, and their well-known preventive effects, elucidating this association is crucial; however, this area remains insufficiently investigated. The majority of the existing evidence originates from non-randomised studies and animal experiments, thereby constraining its clinical applicability.^24–27^

Evidence from well-designed Mendelian randomisation studies, when interpreted in the context of other studies as part of a triangulation of evidence exercise, can be regarded as providing strong inference in the evidence-based medicine tradition.^28^ Two recent Mendelian randomisation studies have examined the general association between genetically predicted blood pressure and NAFLD risk^29,30^, both reporting protective effects of lower blood pressure. However, neither study investigated the class-specific effects of antihypertensive drug targets, employed eQTL-based instrument selection to enhance biological specificity, nor triangulated genetic findings with randomised controlled trial (RCT) data. Our study addresses these gaps by applying a comprehensive drug-target MR framework that integrates cis-eQTL-informed instrument selection, class-specific analyses of four major antihypertensive drug classes, and direct comparison with trial-derived effect sizes using individual participant data.

## Method

### Data Availability statement

The data that support the findings of this study are available from the corresponding author upon reasonable request. Summary-level genetic data are available from the original studies cited in this article. Access to individual participant data from the Blood Pressure Lowering Treatment Trialists’ Collaboration is subject to the Collaboration’s established data-access procedures and applicable data-use agreements.

### Study design

A Mendelian randomisation framework was utilised,^28,31,32^ leveraging the genetic principle of the random inheritance of variants to emulate the design of an RCT to investigate (i) BP reduction overall effect by any means, and (ii) BP reduction effect through major antihypertensive drug class targets, on the risk of NAFLD (Figure S1). To investigate the overall effect of BP reduction, we used two-sample Mendelian randomisation using genetic data from variants associated with systolic BP across the whole genome.^33^ To estimate the effect of each drug class, we employed a drug-target Mendelian randomisation approach. This method leverages genetic variants within gene(s) encoding proteins associated with drug targets to infer the effect of modulating the key drug-effect biomarker (i.e., systolic BP) through this targeted gene(s) on clinical outcomes.^34^ Systolic BP was selected as the primary exposure because it is the principal measure of hypertension burden in contemporary clinical guidelines^35–37^ and is the primary pharmacodynamic endpoint in antihypertensive randomised controlled trials. Although diastolic BP may have clinically relevant associations that are partly independent of systolic BP^38^, the two traits are highly genetically correlated. Given this substantial genetic overlap and the prespecified focus of our study, we restricted the analyses to systolic BP rather than examining both BP traits in parallel. Coronary heart disease was selected as a positive control outcome because it shares closely overlapping cardiometabolic risk factors with NAFLD—including hypertension, insulin resistance, dyslipidaemia, and obesity—and the two conditions frequently co-occur, with up to 40–70% of patients with NAFLD also having concurrent hypertension.^39^ The causal architecture linking systolic blood pressure to CHD is among the best-established in cardiovascular medicine, making CHD an ideal benchmark for validating our analytical framework. The positive control is not intended as a direct proxy for a hypothetical NAFLD–SBP trial, but rather serves to demonstrate that our genetic instruments and methods faithfully recover a known causal relationship, thereby providing assurance that the same approach, when applied to NAFLD, is operating as intended. The ratio of MR-to-trial effect sizes observed for CHD further enables calibration of the expected magnitude of a trial-equivalent NAFLD effect.

Recognising that treatment effects estimated using genetic data may not exactly reflect those observed in real-world RCT settings—owing to differences in follow-up duration, participants’ baseline risk, drug adherence, and discontinuation rates—we sought to enhance the clinical validity and interpretability of the genetically derived estimates.^40^ To facilitate direct comparison with evidence from phase III RCTs, we conducted a one-stage individual participant data (IPD) meta-analysis of RCTs (Supplemental Text 1) alongside a network meta-analysis (Supplemental Text 2), incorporating coronary heart disease as a joint positive control outcome (i.e., effect sizes estimated using both RCT and genetic data). Given the well-established effect of BP-lowering treatments on coronary heart disease and the abundance of large-scale RCTs, we selected coronary heart disease as a positive control outcome for this study.

### Data sources

Genetic data for systolic BP in all the analyses were obtained from the largest genome-wide association study (GWAS) currently available, which included data from over 1 million participants of European ancestry.^41^ For NAFLD as the primary outcome, we obtained genetic data for corresponding variants from the Million Veteran Program, which included 68,725 cases and 95,472 controls of European ancestry.^42^ Additionally, for coronary heart disease, we utilised GWAS data comprising 181,522 cases and 984,168 controls, predominantly of European ancestry.^43^ To refine the process of variant selection for drug-target Mendelian randomisation, we used cis-expression quantitative trait loci (eQTL) data from eQTLGen Consortium, incorporating whole blood-derived expression data from 31,684 individuals across 37 datasets.^44^ For drug classes that demonstrated a significant effect on NAFLD, a post-hoc sensitivity analysis was conducted to enhance biological relevance. This analysis utilised tissue-specific cis-eQTL data from the Genotype-Tissue Expression (GTEx) project.^45^ Details of all genetic data used in this study are described in Table S1.

To estimate the overall effect of BP reduction on coronary heart disease using trial data, we utilised individual-level data from the Blood Pressure Lowering Treatment Trialists’ Collaboration (BPLTTC). This collaboration, hosted at the University of Oxford (Oxford, UK), provides access to individual-level data from 51 randomised trials investigating the effects of pharmacological blood pressure-lowering treatments on a variety of cardiovascular outcomes (Table S2).^20–22,46–48^ To investigate the effects of each drug class, we conducted an aggregate data network meta-analysis, built on our previously published systematic review and meta-analysis (Supplemental Text 2 and Table S3).^49^ For this analysis, data from 104 RCTs investigating specific classes of antihypertensive drugs versus placebo or other antihypertensive agents were included.

### Outcomes definition

NAFLD was defined as (1) elevated alanine aminotransferase (ALT) levels of higher than 40 U/L for men or 30 U/L for women on at least two occasions, separated by a minimum of six months, within a two-year timeframe prior to enrolment; and (2) the exclusion of other causes of liver disease, chronic liver conditions, systemic diseases, or alcohol use disorders (Table S4).^42^ In the GWAS study used for joint positive control outcome, the ascertainment of coronary heart disease was predominantly based on diagnostic codes obtained from primary and secondary healthcare records (Table S1).^43^

In trial data analyses, coronary heart disease was defined as fatal or non-fatal myocardial infarction, ischaemic heart disease, or surgical procedures including coronary artery bypass grafting and percutaneous transluminal coronary angioplasty. Coronary heart disease outcome ascertainment was conducted individually by each trial, with the data subsequently shared with the BPLTTC (Table S2).^20,21,47–49^

### Selection of genetic variants

All variants with a minor allele frequency (MAF) greater than 1%, with linkage disequilibrium (LD) threshold of r^2^ < 0.001, and a clumping window of 10,000 kb, which were associated with systolic BP (*P* < 5 × 10^−8^) at the whole genome level, were included in the analysis to estimate the overall effect of BP reduction.^41^ We did not limit variant selection to specific gene regions; instead, variants meeting the inclusion criteria were included from across the genome, to estimate the overall effect of systolic BP reduction.

We followed a predefined and sequential pipeline for the drug-target analysis, which was developed and validated specifically for this study (Figure S2). In brief, we began with a comprehensive review of the literature and relevant databases to identify all commonly used antihypertensive medications and their associated target genes. Next, we extracted cis-variants located within 500 kb on each side of target genes. Subsequently, we filtered variants based on the presence of significant cis-expression quantitative trait loci (cis-eQTL) signals in whole blood samples. These variants were further refined by assessing whether they demonstrated a significant causal association between gene expression and GWAS-derived systolic BP effect sizes, using single-variant two-sample Mendelian randomisation. Filtered variants were subsequently grouped by gene, and the overall correlation between gene expression and systolic blood pressure was examined using Z-Z plots. An evaluation of the biological mechanisms of each refined gene was then conducted to further assess its relevance to blood pressure pathways (Table S5). Finally, we estimated the effect of each drug class, based on the refined variants with LD threshold of r^2^ < 0.05, on the risk of coronary heart disease as a secondary outcome and then NAFLD as the primary outcome, using two-sample Mendelian randomisation.

### Statistical analysis

For the Mendelian randomisation, odds ratios (ORs) were estimated using the inverse-variance-weighted (IVW) method, serving as the primary analytical approach.^50^ Further methods were utilised in sensitivity analyses to evaluate the robustness of the findings against potential violations of underlying assumptions (Supplemental Text 3). The IVW is considered robust when the results remain consistent across various models with different assumptions. The MR-Egger regression intercept was used to estimate the existence of potential directional horizontal pleiotropy.^51^ Mendelian randomisation estimates were initially obtained on the log-odds scale per 1-mmHg higher systolic BP. To express associations per 5-mmHg lower systolic BP, the log-odds estimates were multiplied by −5 and exponentiated to obtain ORs with 95% CIs. The estimates were rescaled to a 5-mmHg reduction, a convenient round number approximating the average systolic BP reduction observed in RCTs. ^47^ Given the association between obesity, hypertension, and NAFLD,^52,53^ we conducted a multivariable Mendelian randomisation analysis, adjusting for genetically predicted BMI to mitigate potential pleiotropic effects through BMI. The magnitude of bias and Type I error rate inflation arising from sample overlap in two-sample Mendelian randomisation was also estimated (Supplemental Text 4).^54^

The drug-target analysis involved first estimating individual target gene effects on outcomes using the Mendelian randomisation IVW method, and subsequently employing random- and fixed-effect meta-analysis models to combine these effects across associated genes to determine the drug class effect. This approach enables the estimation of class-specific effects by considering each gene as a unit of analysis, accommodates heterogeneity among genes targeting the same drug class, and facilitates the testing of consistency across genes. The estimates of between-study variance in a random-effects meta-analysis were employed to quantify heterogeneity among target genes associated with the same drug class, providing a measure of inconsistency across genes. A random-effects model was deemed the most appropriate estimation method when I^2^ exceeded 60%, Cochran’s Q test was significant and τ^2^ demonstrated substantial variability, indicating significant heterogeneity. For drug classes that showed a significant effect on NAFLD, we conducted a post hoc analysis using cis-eQTL data from the GTEx (Supplemental Text 5). Colocalisation analyses were conducted using both *coloc.abf* (assuming a single causal variant) and *coloc.susie* (allowing for multiple causal variants). In addition, LocusZoom-style regional association plots were generated for each gene region. These analyses were performed for any drug target that exhibited a significant association with the risk of NAFLD, to further evaluate the assumptions underlying the Mendelian randomisation analysis (Supplemental Text 6).^55^

In trial data analysis, to assess the effect of overall BP reduction on coronary heart disease, we used a fixed-effect one-stage IPD meta-analysis that fits a single statistical model to the IPD from all trials simultaneously.^21^ The hazard ratio (HR) was calculated using a Cox proportional hazard model stratified by trial and standardised for a 5 mm Hg systolic BP reduction.^21,22,48,56^ Kaplan-Meier estimates of cumulative incidence were used to compute event rates, plotted separately for trial intervention and comparator arms.

We also performed a frequentist network meta-analysis to estimate the effect of each antihypertensive drug class compared with placebo on the risk of coronary heart disease.^57^ This analysis utilised aggregate data from RCTs, incorporating direct and indirect evidence to provide a comprehensive estimate of treatment effects (Table S3). Pairwise comparisons were performed within a network framework, and effect sizes were expressed as relative risks (RR) with 95% confidence intervals.

To assess the validity of the genetically derived estimate, the effect size obtained from Mendelian randomisation (ORs) was compared with that derived from the trial data (HRs), provided that the following criteria were met: (i) the cumulative incidence of coronary heart disease is below 10%; (ii) the exposure–outcome association is linear; and (iii) both analyses employed the same exposure unit.

All analyses were predefined unless specified as a post-hoc sensitivity analysis. All statistical analyses were conducted using R version 4.3.0.

### Role of the funding source

The funders of the study had no role in study design, data collection, data analysis, data interpretation, or writing of the report.

## Results

**Figure 1:**
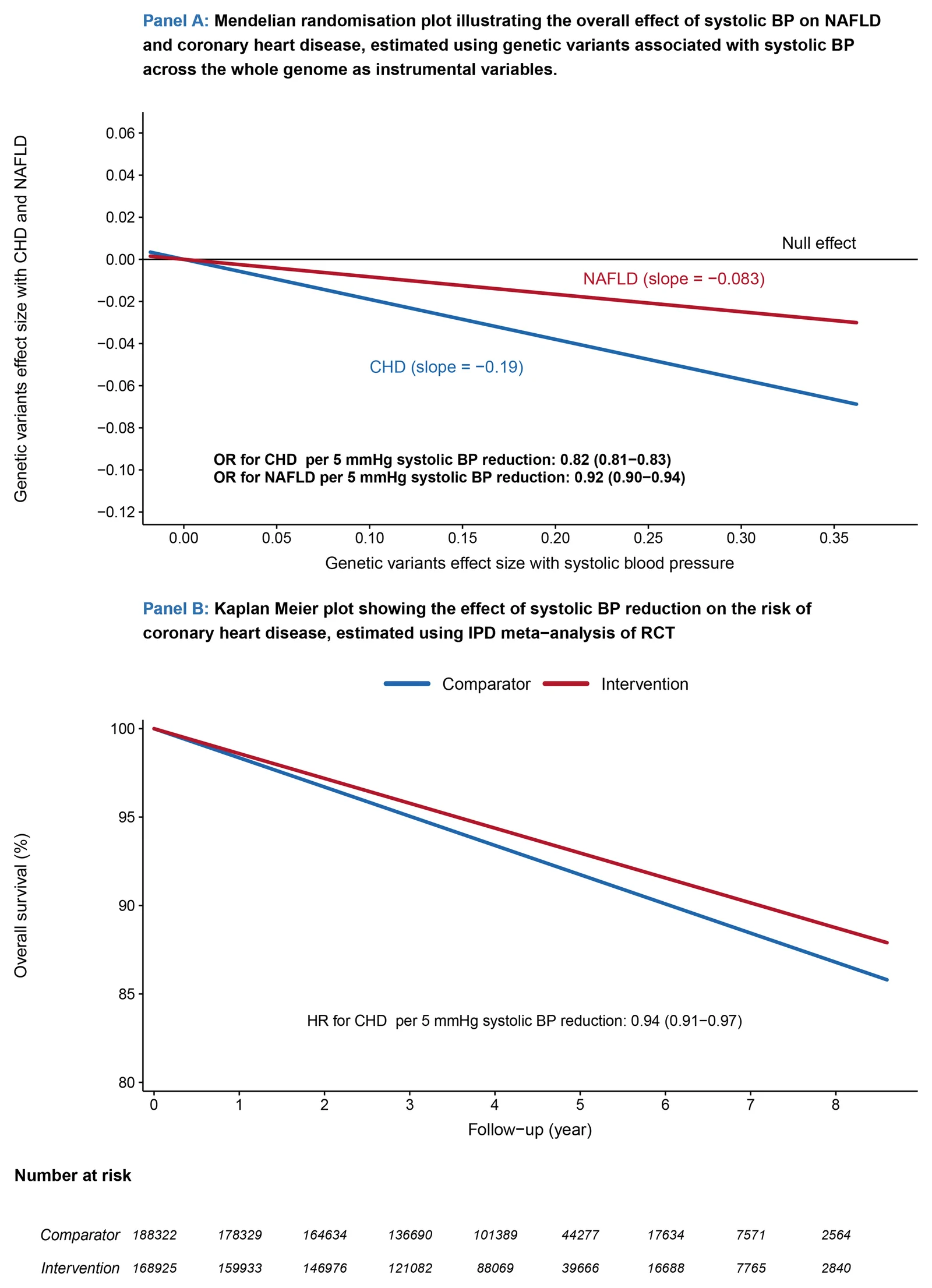
Results of Mendelian randomisation and individual participant data meta-analysis of randomised controlled trials for the effect of overall systolic blood pressure reduction on the risk of non-alcoholic fatty liver disease and coronary heart disease. BP: blood pressure, RCT: randomised controlled trial, NAFLD: non-alcoholic fatty liver disease, IPD: individual participant data, CHD: coronary heart disease, HR: hazard ratio, OR: odds ratio

The general characteristics of the population included in the GWAS used for this study are shown in Table S1. To investigate the overall effect of systolic BP-lowering, 410 genetic variants met the eligibility criteria and were included in the analysis (Table S6). We observed a protective effect of systolic BP reduction on NAFLD, with each 5 mmHg reduction leading to an 8% reduction in the risk (OR 0.92 [95% CI 0.90 to 0.94]) (Figure 1, Panel A). Evidence of directional horizontal pleiotropy was initially detected through the MR-Egger intercept (β = -0.005, P = 0.005), which was considerably reduced after excluding outlier variants (β = -0.002, P = 0.15). When replicating the analysis using other methods of Mendelian randomisation with different assumptions and different NAFLD GWAS data, we found that the estimated effect size remained unchanged. Furthermore, the findings from the rest of the sensitivity analyses further supported the main estimates (Figures S3– S8 and Tables S7–S9). In a multivariable Mendelian randomisation analysis adjusting for genetically predicted body mass index, the association remained robust (OR 0.79, 95% CI 0.66 to 0.96; Figure S3).

**Figure 2:**
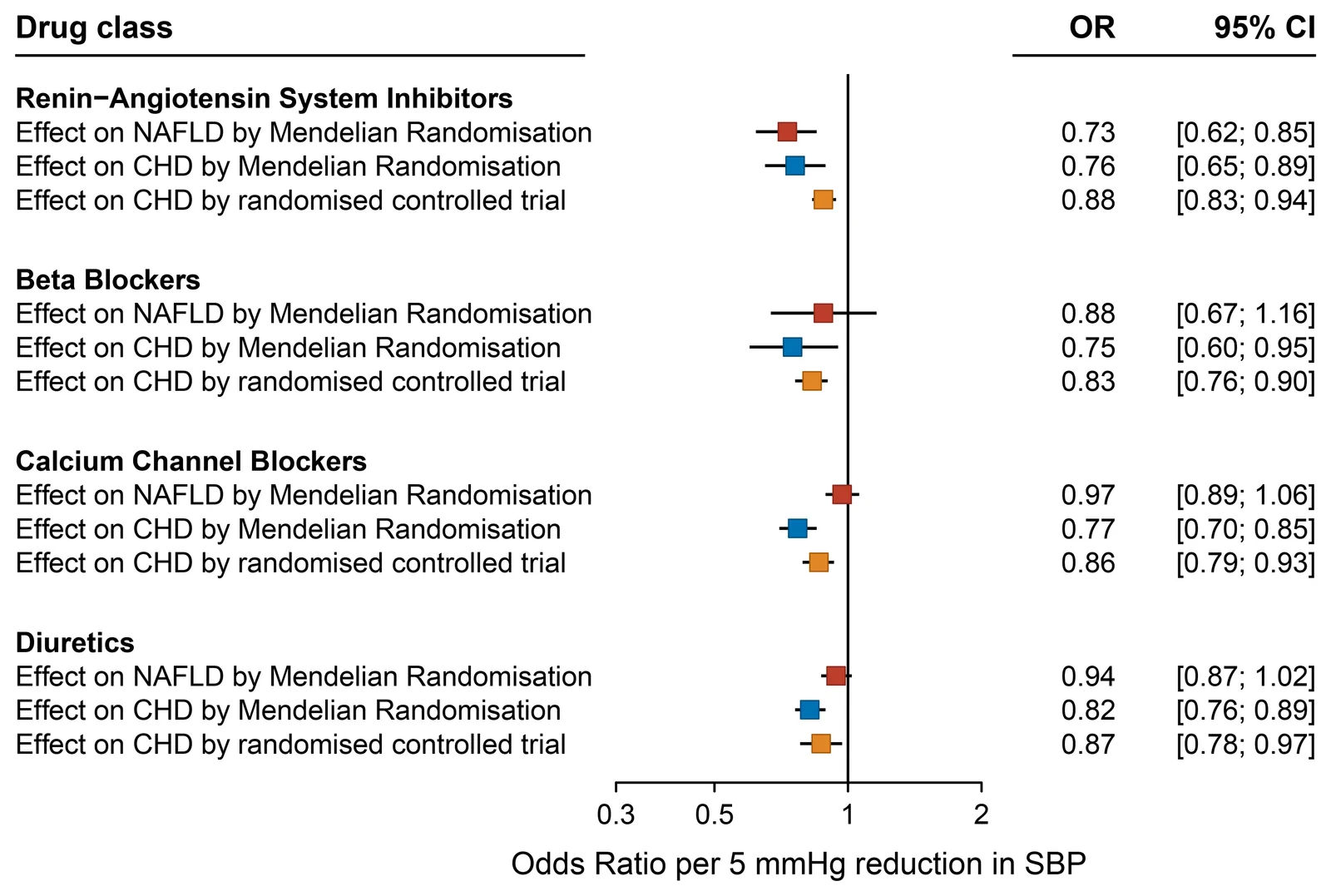
Effect of major antihypertensive drug classes on non-alcoholic fatty liver disease as the primary outcome and coronary heart disease as the joint positive control outcome. SBP: systolic blood pressure; NAFLD: non-alcoholic fatty liver disease; CHD: coronary heart disease; OR: odds ratio; CI: confidence interval.

In the drug-target analysis, we identified 92 target genes for four major classes of antihypertensive drugs (Tables S10–S11). Of these, 63 genes that were significantly expressed in whole blood were queried using the eQTLGen data. Among the 63 genes, 56 had genetic variants significantly associated with systolic BP (P < 0.05), and Z-Z plots for these variants generally displayed a correlation trend (Figure S9). After clumping, independent genetic variants for each gene were taken forward to the drug-target Mendelian randomisation study (Tables S12–S15). All four drug classes demonstrated a protective effect against coronary heart disease, yet only RAS inhibitors exhibited a protective effect against NAFLD (odds ratio [OR] 0.73, 95% CI 0.62 to 0.85) (Figure 2). No significant effects were observed for the other drug classes in relation to NAFLD. These result remain consistent when in replication analyses using genome-wide association studies using diagnostic codes for NAFLD (Table S7).

Coronary heart disease was examined as a positive-control outcome to assess whether the selected genetic instruments recovered the established protective association of systolic BP lowering observed in clinical trials. Using the same genome-wide variants, a genetically proxied 5-mmHg lower systolic BP was associated with an 18% lower risk of coronary heart disease (OR, 0.82 [95% CI, 0.81–0.83]; Figure 1, **Panel A**). In the individual participant data meta-analysis of 358,636 participants from 51 randomised controlled trials, a 5-mmHg reduction in systolic BP was associated with a hazard ratio of 0.94 (95% CI, 0.91–0.97) for coronary heart disease (Figure 1, **Panel B**). The genetic and trial estimates were directionally concordant, although the genetically proxied association was stronger (Figure 1). At the drug-class level, the network meta-analysis of 104 randomised controlled trials showed lower coronary heart disease risk for all four antihypertensive drug classes, consistent in direction with the corresponding drug-target Mendelian randomisation estimates, which generally indicated stronger relative associations (Figure 2). MR estimates reflect lifelong genetically determined exposure and are expected to be stronger than trial-derived estimates, which capture finite treatment durations. If the same MR-to-trial ratio observed for CHD (log-OR ratio ≈ 3.1) were applied to the overall NAFLD estimate, the corresponding trial-equivalent effect would be approximately HR 0.97 per 5 mmHg SBP reduction—modest but potentially meaningful at a population level. For RAS inhibitors, the calibrated trial-equivalent effect (HR ≈ 0.90) would remain clinically significant. Details of the included trials and analyses are presented in Supplemental Texts 1–2 and Tables S2–S3.

**Figure 3:**
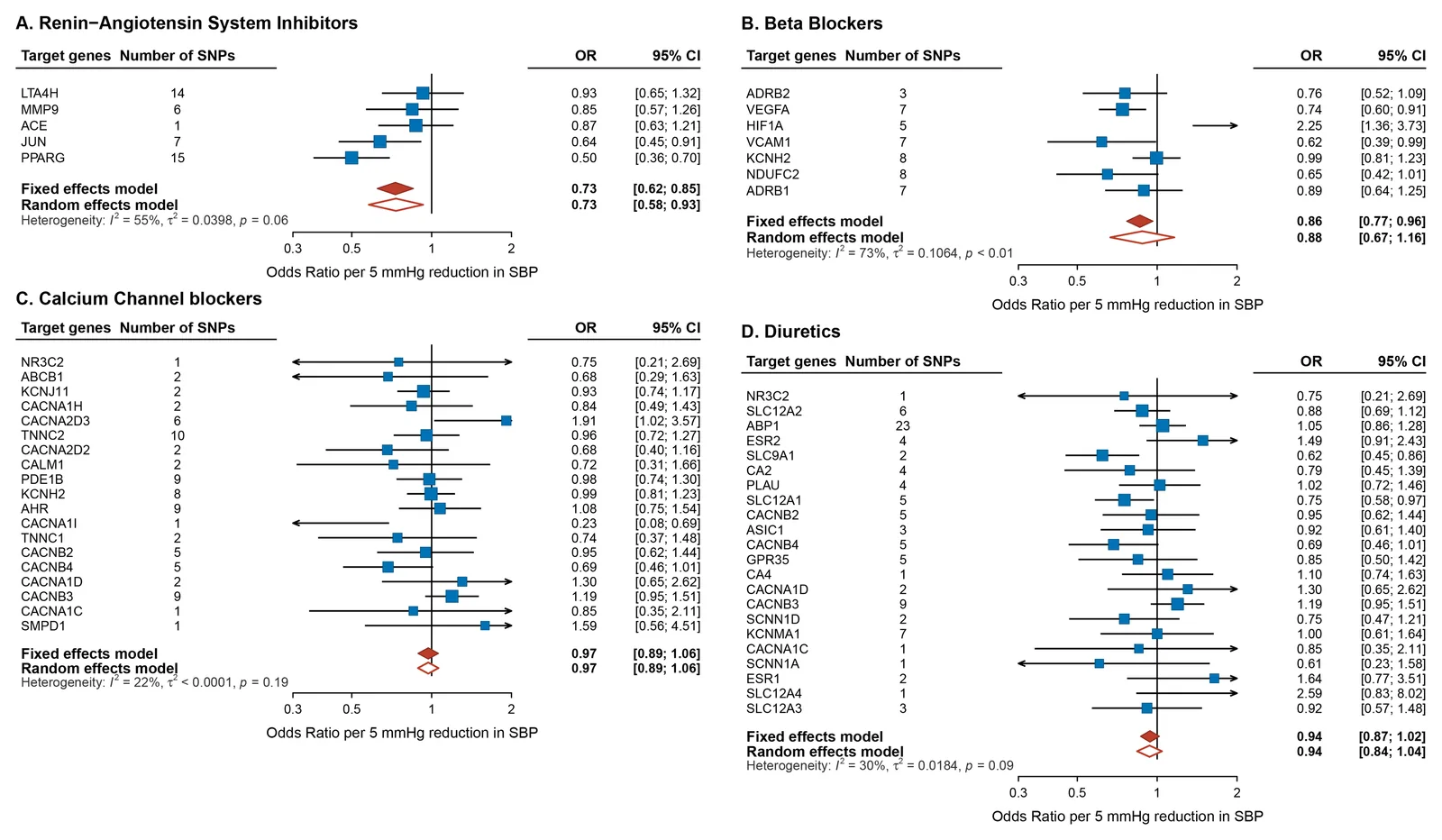
Effect of individual target genes associated with major antihypertensive drug classes on non-alcoholic fatty liver disease. SBP: systolic blood pressure; NAFLD: non-alcoholic fatty liver disease, SNPs: single nucleotide polymorphisms; OR: odds ratio; CI: confidence interval.

Figure 3. illustrates the separate effects of each gene within each drug class on NAFLD and the heterogeneity among them. Except for beta-blockers, the target genes of the other three drug classes showed homogeneous effects on NAFLD, with low I^2^ and τ^2^ statistics and non-significant Cochran’s Q test results. The gene-level meta-analyses should be interpreted as descriptive summaries of the consistency and heterogeneity of individual target-gene effects within each drug class, rather than as estimates of the true pharmacological contribution of each gene to the overall drug class effect. For the RAS inhibitors, which demonstrated a protective effect on NAFLD, the genes *JUN* and *PPARG* showed significant protective effects, while point estimates for other target genes indicated consistent but weak protective effects, with confidence intervals crossing the null (Figure 3). In post hoc analyses of the RAS class, we employed complementary approaches to validate our primary IVW findings, and both yielded consistent results (Figures S10–S11 and Table S16). Furthermore, RAS inhibitors remained protective on NAFLD, with an OR of 0.80 (95% CI 0.68 to 0.95), when using genetic instruments further validated by cis-eQTL data from GTEx (Figure S12). Colocalisation analysis showed stronger evidence for PPARG than JUN. PPARG had moderate signals under the single variant assumption and stronger signals under the multiple variant assumption. The LocusZoom-style regional association plots did not provide evidence against colocalisation for either gene. Besides, VEGFA—targeted by beta-blockers—also demonstrated significant colocalisation under the multiple-variant assumption; however, the regional association plot did not support colocalisation (Figure S13).

## Discussion

Our findings indicate that systolic BP is a modifiable risk factor for NAFLD, and that a modest and achievable systolic BP reduction of 5 mmHg could be an effective strategy for preventing NAFLD. We identified evidence that systolic BP reduction through RAS inhibition may represent a promising preventive strategy for NAFLD. The clinical relevance of these findings is further strengthened by supplementary analyses showing that genetically estimated drug effects, when triangulated by biological, genetic, and clinical evidence, align closely with results observed in clinical trials testing similar hypotheses; with effect estimates derived from Mendelian randomisation frameworks slightly stronger than those observed in trial-based evidence.

There is currently no published high-quality phase III trial specifically addressing the BP-lowering treatment effect on risk of metabolic liver diseases. Most existing knowledge is derived from small trials or studies investigating cardiovascular risk reduction treatments in patients with NAFLD (Table S17). For example, in the PolyIran-Liver trial, a pragmatic trial involving 2,400 participants, the efficacy of the Polypill—comprising fixed doses of aspirin, a thiazide diuretic, a statin, and an angiotensin receptor blocker—was assessed.^58^ While no significant changes in ALT levels were observed in participants with fatty liver overall, individuals with presumed non-alcoholic steatohepatitis demonstrated a significant reduction in ALT levels by 10.5 IU and 12 IU after 30 and 60 months of Polypill therapy, respectively.^58^ Several small-scale RCTs, with sample sizes ranging from 7 to 150 participants, have explored the effects of RAS inhibitors, either alone or in combination with other antihypertensive agents, on the progression of NAFLD. However, the low quality of evidence and substantial limitations in the trial design—such as recruitment challenges, single-arm study structures, and insufficient statistical power—have impeded the ability to draw clinical conclusions.^59–63^

We investigated the effect of both overall BP reduction and of specific antihypertensive drug classes to address two distinct but complementary clinical questions. First, if elevated BP is considered a modifiable risk factor and is lowered by a modest amount—regardless of therapeutic targets or pathways—what is the estimated relative effect on NAFLD? We found that for every 5 mmHg reduction in systolic BP, the risk of developing NAFLD is reduced by approximately 8% compared with individuals without BP-lowering treatment. While modest, this effect is nonetheless clinically meaningful, particularly when combined with established cardiovascular benefits and readily achievable at low cost through conventional antihypertensive therapy.

Second, if we reduce BP specifically through pathways targeted by major antihypertensive drug classes, what is the relative effect? In this context, RAS inhibition conferred a markedly greater impact on incident NAFLD, yielding a 27% relative risk reduction. This pronounced effect suggests that RAS blockade may afford hepatometabolic protection beyond known cardiovascular benefits, possibly through anti-inflammatory, anti-fibrotic and insulin-sensitising mechanisms inherent to this pathway. From a clinical standpoint, these data support the preferential use of angiotensin-converting enzyme inhibitors or angiotensin receptor blockers in hypertensive patients at high risk of developing metabolic liver disease. It should be noted, however, that evidence derived from genetic (Mendelian randomisation) analyses does not directly translate into identical effect sizes in real-world practice, where dosage, duration of administration, adherence and other factors play pivotal roles. In our analysis, these variables were assumed to be optimal—an ideal seldom met in routine care.

The protective effect of BP reduction on NAFLD, particularly via RAS inhibition, is biologically plausible and corroborated by preclinical studies. Hypertension exacerbates hepatic injury by elevating vascular resistance, diminishing insulin sensitivity, impairing liver microcirculation, and fostering oxidative stress, thereby intensifying hepatic steatosis and progressing to liver fibrosis.^64^ Lowering BP mitigates these haemodynamic stresses, enhancing hepatic perfusion and reducing hypoxia-induced inflammation and fibrosis. Moreover, hypertension-induced endothelial dysfunction and arterial stiffness amplify liver damage through disrupted nitric oxide signalling and increased pro-inflammatory cytokine production.^65^ RAS inhibitors also indirectly benefit hepatic outcomes by improving insulin sensitivity and modulating dysregulated adipokine levels, such as leptin and adiponectin, which are pivotal in NAFLD pathogenesis.^66^ Hypertension-induced renal dysfunction further aggravates hepatic injury, perpetuating BP elevation and its adverse effects on liver health. Effective BP control alleviates these renal contributions, reducing hepatic congestion and restoring metabolic homeostasis. Animal studies support these mechanisms, demonstrating that BP reduction alone attenuates liver fibrosis and steatosis, and hence underscoring the central role of haemodynamic normalisation in liver protection.^67^ Collectively, these findings highlight the significance of RAS inhibition and BP management in NAFLD pathophysiology, suggesting that modulating this pathway may mitigate the hepatic consequences of hypertension.

This study has limitations. First, Mendelian randomisation analysis assumes the absence of alternative causal pathways, with the selected variants serving as instrumental variables for systolic BP influencing NAFLD solely through the exposure of interest (i.e., no pleiotropic effects). While it is not possible to entirely rule out the presence of pleiotropy in the variants used in this study, extensive sensitivity analyses did not yield any strong evidence supporting such effects. Second, drug-target genetic estimates reflect the effects of lifelong exposure to a biomarker on an outcome and cannot directly estimate the expected effects of short-term pharmacological changes in that biomarker (e.g., systolic BP). However, in this study, we conducted independent analyses of RCT data and compared those findings with genetic estimates of joint positive outcomes that share closely related pathophysiological mechanisms and risk factors, and also a high percentage of co-incidence with NAFLD. While drug effect estimates derived from genetic data tended to indicate slightly stronger relative effect sizes than those observed in RCTs, the overall relative risk reductions were materially similar. When the analytical assumptions are satisfied,^68^ and the findings are triangulated with other sources of evidence,^69,70^ the estimated effect size from Mendelian randomisation framework can offer a reliable approximation of the effects of a drug class, comparable to those observed in RCTs. Therefore, it is reasonable to assume that a large-scale RCT with an optimal drug compliance rate and long follow-up time would yield comparable results. Third, the genetic data used for NAFLD reflects the new onset events rather than its progression. Thus, whether BP-lowering treatment influences the progression of NAFLD at various stages of the disease warrants further investigation through RCTs incorporating repeated measurements of NAFLD progression biomarkers as primary outcomes. Fourth, we focused on systolic BP and did not separately evaluate diastolic BP. Given that diastolic BP may have clinically relevant associations partly independent of systolic BP, its potential role in metabolic dysfunction–associated fatty liver disease warrants further investigation.

## Conclusion

This study provides concordant genetic evidence supporting a potential causal role of lower systolic BP in reducing the risk of NAFLD and identifies RAS-related targets as the most promising antihypertensive pathway for further investigation. These findings highlight the potential of BP-related pathways in the prevention of metabolic liver disease and support the further evaluation of RAS inhibitors as candidates for drug repurposing. Given their widespread clinical use, affordability, and established cardiovascular benefits, RAS inhibitors may represent a particularly promising therapeutic strategy, although their effects on NAFLD require confirmation in dedicated clinical studies.

## Perspectives

These findings highlight the potential broader benefits of blood pressure control beyond cardiovascular disease and support further evaluation of RAS inhibition as a candidate strategy for preventing metabolic liver disease. Given the widespread use, affordability, and established cardiovascular benefits of RAS inhibitors, confirmation of a hepatoprotective effect could have important implications for patients with coexisting hypertension and metabolic risk. Prospective observational studies and adequately powered randomised controlled trials should determine whether RAS inhibitors reduce the incidence or progression of NAFLD. Future studies should incorporate prespecified liver outcomes, repeated assessments of hepatic steatosis and fibrosis, and detailed evaluation of treatment duration, adherence, baseline metabolic risk, and blood pressure response.

## Supplementary Material

Supplemental Publication Material

## Novelty and Relevance

### What Is New?

- This is the first study to use drug-target Mendelian randomisation to evaluate the potential effects of major antihypertensive drug classes on metabolic dysfunction–associated fatty liver disease.
- Because genetically inferred treatment effects may not mirror outcomes observed in real-world randomised controlled trials owing to differences in follow-up duration, baseline participant risk, and adherence, we integrated genetic data from over one million individuals with individual participant data from 358,636 participants in 51 randomised controlled trials and aggregate data from more than 500,000 participants across 104 trials.

### What Is Relevant?

- A genetically proxied 5-mmHg lower systolic blood pressure was associated with an 8% lower risk of metabolic dysfunction–associated fatty liver disease(MAFLD; OR, 0.92 [95% CI, 0.90–0.94]).
- Drug-target Mendelian randomisation identified renin–angiotensin system-related targets as the most promising antihypertensive pathway for lowering the risk of MAFLD (OR, 0.73 [95% CI, 0.62–0.85]).

### Clinical/Pathophysiological Implications?

- These findings support further clinical evaluation of blood pressure lowering, particularly through renin–angiotensin system inhibition, for the prevention of MAFLD.
