# Supplementary material for: Antihypertensive Treatment and Risk of Metabolic Associated Fatty Liver Disease: A Drug-Target Mendelian Randomisation
Source: Hypertension. Author manuscript; Available in PMC 2026 Oct 21. (PMC7619608; doi:10.1161/HYPERTENSIONAHA.126.27526)

Tile page

Title: Antihypertensive Treatment and Risk of Metabolic Associated Fatty Liver Disease: A Drug-Target Mendelian Randomisation

Short Title: Antihypertensive Treatment and MAFLD

Yifan Hu, PhD[1,2,3]; Karl Smith-Byrne, DPhil[4]; Zeinab Bidel, MSc[2]; Xiong Xiao, PhD[3]; Xing Zhao, PhD[3]; Prof Abbas Dehghan, PhD[5]; Dexter Canoy, PhD[6]; Malgorzata Wamil, PhD[2,7,8]; Nathalie Conrad, DPhil[2,9]; Shishir Rao, DPhil[2]; Ben Omega Petrazzini, BSc[2]; Qianqian Yang, MSc[2]; Dipender Gill, PhD[5]; Venexia M Walker, PhD[10,11,12]; Charalampos Sigalas, PhD[13]; Prof Reza Malekzadeh, MD[14]; Prof Johan Sundström, PhD[15,16]; Prof Mark Woodward, PhD[16,17]; Prof George Davey Smith, FRS[10]; Prof Kazem Rahimi, FRCP[2]; Milad Nazarzadeh, DPhil[2]

1. Sichuan Provincial People's Hospital, University of Electronic Science and Technology of China, Chengdu, China
2. Deep Medicine, Nuffield Department of Women 's and Reproductive Health, Medical Science Division, University of Oxford, Oxford, UK
3. Department of Epidemiology and Health Statistics, West China School of Public Health, Sichuan University, Chengdu, China
4. Cancer Epidemiology Unit, Nuffield Department of Population Health, University of Oxford, Oxford, UK
5. Department of Epidemiology and Biostatistics, Imperial College London, London, UK
6. Population Health Sciences Institute, Newcastle University, Newcastle, UK
7. Cardiology Department, Great Western Hospital NHS Trust, Swindon, UK
8. Cardiology Department, Mayo Clinic Healthcare in London, London, UK
9. Department of Cardiovascular Sciences, KU Leuven, Leuven, Belgium
10. Medical Research Council Integrative Epidemiology Unit, University of Bristol, Bristol, UK
11. Population Health Sciences, Bristol Medical School, University of Bristol, Bristol, UK
12. Department of Surgery, University of Pennsylvania Perelman School of Medicine, Philadelphia, USA
13. Department of Pharmacology, University of Oxford, Oxford, UK
14. Liver and Pancreatobiliary Diseases Research Center, Digestive Diseases Research Institute, Tehran University of Medical Sciences, Tehran, Iran
15. Department of Medical Sciences, Uppsala University, Sweden
16. The George Institute for Global Health, University of New South Wales, Sydney, Australia

Part A — Supplementary Text
Part B — Supplementary References
Part C — Supplementary Tables
Part D — Supplementary Figures

Part A — Supplementary Text

No.	Contents
Supplemental Text 1	Details of individual participant data meta-analysis of randomised controlled trials for assessing the effect of systolic blood pressure reduction on the risk of coronary heart disease as a joint positive outcome
Supplemental Text 2	Details of network meta-analysis to assess the effect of each antihypertensive drug class versus placebo on risk of coronary heart disease using aggregate level data from randomised controlled trials
Supplemental Text 3	Details of the Mendelian randomisation sensitivity analysis methods
Supplemental Text 4	Details of the sensitivity analysis method for estimating bias and Type I error rate due to the use of genome-wide association study data with sample overlap
Supplemental Text 5	Details of the post hoc analysis method using cis-eQTL data from the Adult Genotype-Tissue Expression (GTEx) Project
Supplemental Text 6	Details of the colocalisation analysis method for further assessing the Mendelian randomisation assumption

Part B — Supplementary References

Part C — Supplementary Tables

No.	Contents
Table S1	Details of the genome-wide association studies from which summary-level data were used for Mendelian randomisation analyses
Table S2	Details of 51 randomised trials from the Blood Pressure Lowering Treatment Trialists' Collaboration (BPLTTC) used to investigate the effects of pharmacological blood pressure-lowering treatments on the risk of coronary heart disease using an individual participant data meta-analysis design
Table S3	Characteristics of randomised controlled trials included in the network meta-analysis to investigate the effect of each antihypertensive drug class versus placebo on the risk of coronary heart disease
Table S4	The diagnostic criteria in each cohort included in the genome-wide association study of non-alcoholic fatty liver disease (NAFLD)
Table S5	Biological mechanisms of the refined final-stage genes involved in blood pressure regulation
Table S6	Characteristics of the genetic variants included in the main Mendelian randomisation analysis
Table S7	Effect of overall systolic blood pressure reduction and major antihypertensive drug classes on non-alcoholic fatty liver disease, replicated using genome-wide association studies that employed diagnostic codes for identifying non-alcoholic fatty liver disease
Table S8	Whole-genome Mendelian randomisation estimate of systolic blood pressure reduction on the risk of NAFLD, accounting for residual linkage disequilibrium (LD-robustness analysis)
Table S9	Extended multivariable Mendelian randomisation: direct effect of systolic blood pressure on NAFLD, jointly adjusted for BMI, waist-to-hip ratio, HbA1c and LDL cholesterol
Table S10	Initially retrieved target genes for commonly prescribed antihypertensive drugs
Table S11	Positional information of target genes for commonly prescribed antihypertensive drugs
Table S12	Genetic variants proxies for renin-angiotensin system (RAS) inhibitors used in drug-target Mendelian randomisation
Table S13	Genetic variants proxies for beta blockers (BBs) used in drug-target Mendelian randomisation
Table S14	Genetic variants proxies for calcium channel blockers (CCBs) used in drug-target Mendelian randomisation
Table S15	Genetic variants proxies for diuretics used in drug-target Mendelian randomisation
Table S16	Sensitivity analysis: exclusion of RAS-inhibitor instruments nominally associated with NAFLD
Table S17	Summary of published and ongoing clinical trials and systematic reviews/meta-analyses assessing the effect of renin–angiotensin system (RAS) inhibitors on non-alcoholic fatty liver disease (NAFLD)/non-alcoholic steatohepatitis (NASH) outcomes

Part D — Supplementary Figures

No.	Contents
Figure S1	Schematic overview of the Mendelian randomisation principle and its comparison to randomised controlled trials.
Figure S2	Flowchart of the stages of the drug-target Mendelian randomisation study for evaluating the effects of antihypertensive drug classes.
Figure S3	Results of the Mendelian randomisation analysis using inverse variance weighting, along with sensitivity analyses, to assess the association between systolic blood pressure lowering and non-alcoholic fatty liver disease
Figure S4	Scatter plot of genetic variant-outcome associations versus variant-exposure associations for the effect of systolic blood pressure reduction on the risk of non-alcoholic fatty liver disease (NAFLD)
Figure S5	Funnel plot of variants, displaying each variant's causal estimate against instrument strength
Figure S6	Cook's Distance bar plot for identifying outlier variants to perform sensitivity analysis excluding outlier variants
Figure S7	Results of the Mendelian randomisation analysis assessing the association between systolic blood pressure lowering and non-alcoholic fatty liver disease, replicated using genome-wide association studies that employed diagnostic codes for identifying non-alcoholic fatty liver disease
Figure S8	Results of the Mendelian randomisation analysis assessing the association between systolic blood pressure lowering and liver enzymes (ALT and AST) as proxies for non-alcoholic fatty liver disease
Figure S9	Z-Z plot for target genes of the four major antihypertensive drug classes before and after filtering each variant by its causal association between the eQTL Z-score and the systolic blood pressure GWAS Z-score
Figure S10	Sensitivity drug-target Mendelian randomisation analyses for renin–angiotensin system (RAS) inhibitor targets using GLS-IVW and GSMR with HEIDI-outlier filtering
Figure S11	Leave-one-out meta-analysis of the renin–angiotensin system (RAS) inhibitor target genes for the effect on non-alcoholic fatty liver disease (NAFLD)
Figure S12	Results of the replication analysis for the effect of renin-angiotensin system (RAS) inhibitors on non-alcoholic fatty liver disease, using genetic instruments validated by cis-eQTL data from GTEx
Figure S13	Results of the colocalisation analyses and LocusZoom plots for significant genes

PART A

Supplementary Text

Supplemental Text 1. Details of individual participant data meta-analysis of randomised controlled trials for assessing the effect of systolic blood pressure reduction on the risk of coronary heart disease as a joint positive outcome

Study governance and data source

This meta-analysis utilised data provided by the Blood Pressure Lowering Treatment Trialists' Collaboration (BPLTTC). The BPLTTC is a collaborative effort comprising principal investigators of major randomised controlled trials of antihypertensive therapy, maintains the largest source of individual participant-level data from such trials currently available.[1] The BPLTTC Steering Committee provides overall direction and scientific leadership, overseeing proposal development, analysis, interpretation, and reporting. An international network of collaborators contributes significantly to all scientific endeavours and discussions. The collaboration is headquartered at the University of Oxford (Oxford, UK) and currently has access to individual participant data (IPD) from 51 randomised trials. The primary inclusion criteria for BPLTTC trials are as follows: randomisation of participants to antihypertensive medications versus placebo, other antihypertensive classes, or different treatment intensities; and at least 1000 person-years of follow-up in each randomised group. Trials exclusively involving participants with heart failure or short-term interventions in those with acute myocardial infarction or other acute conditions were excluded. Ethical approval for this phase of the study was obtained from the Oxford Tropical Research Ethics Committee (OxTREC Reference 545–14). The study protocol was approved by the BPLTTC Steering Committee and collaborators prior to data release for analysis.[1]

Study selection criteria

For this analysis, we included BPLTTC trials that had collected and shared data on baseline blood pressure measurements, coronary heart disease outcomes, including event timing, and the presence or absence of pre-existing cardiovascular disease at baseline. Cardiovascular disease at baseline was defined as any history of stroke, myocardial infarction, or ischaemic heart disease prior to randomisation. Participants with a known diagnosis of heart failure at baseline were excluded from the analysis, consistent with the overall exclusion of trials solely conducted in this population.[2]

Comparison Groups

Individual trials were categorised into two groups: intervention and comparator. In placebo-controlled trials, the placebo group was the comparator, and the active drug group was the intervention. In trials with two or more active groups, the group with the greater blood pressure reduction was designated as the intervention, and the other treatment group(s) as the comparator. Trials comparing different treatment intensities were classified with the more intensive treatment group as the intervention, and the less intensive group as the comparator.[1-4]

More details on statistical analysis

Data were analysed on an intention-to-treat basis, according to the group to which each participant was initially assigned (intervention vs. comparator). Individual participant data from each trial were harmonised and merged into a single dataset to facilitate a one-stage IPD meta-analysis. Stratified Cox proportional hazards models, with fixed treatment effects and participants as the unit of analysis, were employed. Stratification allowed for the specification of the baseline hazard function for each trial, thus ensuring adherence to the proportional hazards assumption. Participants were followed up from the date of randomisation until the earliest occurrence of coronary heart disease, death, or the end of the trial. The average systolic blood pressure reduction between randomised groups, excluding the first 12 months, across all included trials was 6.3 mmHg (95% CI 6.1–6.4). Effect sizes were standardised for a 5 mmHg reduction in systolic blood pressure between randomised groups, a convenient round number approximating the average systolic blood pressure reduction.[5]

Standardisation is appropriate when pooling the effects of antihypertensive treatments and expressing the effect for a fixed level of blood pressure reduction. This approach is crucial because the magnitude of blood pressure reduction

varies across trials. Standardisation adjusts for this heterogeneity by assigning greater weight to trials with larger blood pressure reductions. Standardisation adjusts for this heterogeneity by assigning greater weight to trials with larger blood pressure reductions, particularly when evaluating the blood pressure-mediated effect. In practical terms, trials with minimal blood pressure reductions between treatment arms are assigned proportionately lower weights compared to unstandardised analyses. Standardisation allows for the inclusion of a wide range of antihypertensive trials without imposing an arbitrary threshold for achieved blood pressure reduction. This enhances statistical power while appropriately accounting for the smaller achieved blood pressure reductions in some trials.[6] For each trial, the average achieved blood pressure reduction was estimated using a linear mixed-effects model. This trial-level blood pressure reduction was incorporated into the dataset as a new variable. A stratified Cox model was fitted to estimate the hazard ratio for the treatment effect, adjusted for this trial-level blood pressure reduction. The relative effect size was then rescaled to correspond to a 5 mmHg reduction in systolic blood pressure. [1-6]

Supplemental Text 2. Details of network meta-analysis to assess the effect of each antihypertensive drug class versus placebo on risk of coronary heart disease using aggregate level data from randomised controlled trials

Study Design and Data Sources

A network meta-analysis (NMA) was conducted to evaluate the effects of different antihypertensive drug classes on the risk of coronary heart disease (CHD). The analysis combined data from randomised controlled trials (RCTs) comparing antihypertensive drug classes with placebo or other antihypertensive agents. This approach enabled both direct and indirect comparisons of treatment effects, providing comprehensive estimates of relative risk reductions. The NMA was based on a previously published systematic review and meta-analysis,[7] from which data from 104 RCTs that reported CHD outcomes were included in the current analysis (Appendix 5). The selection of trials followed a predefined protocol, ensuring the inclusion of studies that met the eligibility criteria for assessing the cardiovascular outcomes of antihypertensive treatments.

Eligibility Criteria

RCTs were eligible for inclusion if they:

- Randomly allocated participants to receive one of the major antihypertensive drug classes (renin-angiotensin system [RAS] inhibitors, beta-blockers, calcium channel blockers, or diuretics) or a placebo/comparator antihypertensive drug.
- Reported CHD events as an outcome measure, defined as fatal or non-fatal myocardial infarction, ischaemic heart disease, or major coronary revascularisation procedures (e.g., coronary artery bypass grafting or percutaneous coronary intervention).
- Had a minimum follow-up of six months to ensure the assessment of long-term treatment effects.
- Provided effect estimates as hazard ratios (HRs), risk ratios (RRs), or odds ratios (ORs) with 95% confidence intervals (CIs) or contained sufficient data to derive these estimates.

Statistical Analysis

The NMA was performed using a frequentist framework, implemented in R version 4.3.0 using the netmeta package.[8] A fixed-effects model was applied as the primary model, with a random-effects model used for sensitivity analysis to account for between-study heterogeneity. Heterogeneity was assessed using Cochran's Q test, I^2 statistics, and τ^2 estimates. Direct and indirect treatment estimates were compared using local inconsistency tests (node-splitting approach). Treatment estimates were presented as network forest plots with corresponding RRs and 95% confidence intervals. A comparison-adjusted funnel plot was used to evaluate potential publication bias.

Results

Treatments considered

BB: Beta-Blockers
CCB: Calcium Channel Blockers
Diu: Diuretics
RASi: Renin-Angiotensin System Inhibitors (ACE inhibitors/ARBs)
Com: Any type of combination therapies
RENi: Renin Inhibitors

Main Findings based on fixed effect model

Treatment effect versus placebo	RR (95% CI)	z-score	p-value
BB	0.83 (0.76; 0.90)	-5.95	<0.0001
CCB	0.86 (0.79; 0.93)	-5.76	<0.0001
Com	0.88 (0.81; 0.96)	-2.95	0.0032
Diu	0.87 (0.78;0.97)	-3.55	0.0004
Placebo	—	—	—
RASi	0.88 (0.83; 0.94)	-6.07	<0.0001
RENi	1.04 [0.83; 1.32]	0.33	0.7448

Sensitivity analysis based on random effects model

Treatment effect versus placebo	RR (95% CI)	z-score	p-value
BB	0.83 (0.76; 0.91)	-4.25	<0.0001
CCB	0.86 (0.79; 0.93)	-3.75	0.0002
Com	0.89 (0.79; 1.01)	-1.92	0.0547
Diu	0.88 (0.78; 0.99)	-2.22	0.0265
Placebo	—	—	—
RASi	0.89 (0.83; 0.94)	-3.96	<0.0001
RENi	1.04 (0.75; 1.45)	0.23	0.8169

Heterogeneity and Inconsistency Assessment Heterogeneity (I^2 statistic): 37.9% (Moderate heterogeneity) Tau^2 = 0.0140,

Tests of Heterogeneity and Inconsistency:

Test	Q-Statistic	Degrees of Freedom (df)	p-value
Total	161.1	100	0.0001
Within Designs	133.95	83	0.0003
Between Designs	27.15	17	0.06

The overall heterogeneity is moderate, indicating some variability across trials. There is some evidence

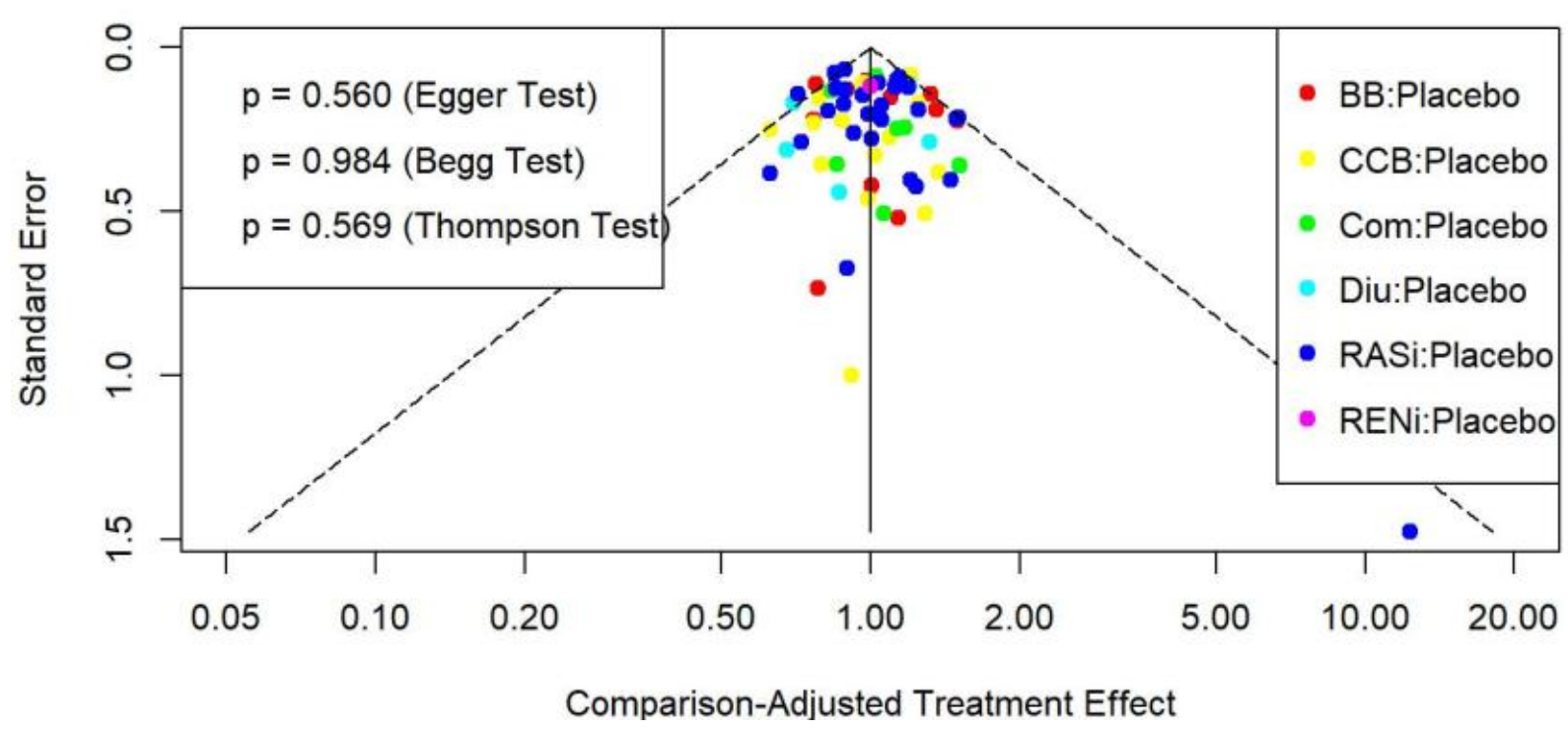

A comparison-adjusted funnel plot to evaluate potential publication bias.

Supplemental Text 3. Details of the Mendelian randomisation sensitivity analysis methods

Weighted median method : This method provides consistent estimates if at least 50% of the weight is derived from valid instrumental variables, ensuring robustness in the presence of invalid instruments[9].

MR-Pleiotropy Residual Sum and Outlier (MR-PRESSO) : to test and, if needed, to correct for any possible horizontal pleiotropic outliers in the analysiThis method is used to test for and, if necessary, correct potential horizontal pleiotropic outliers in the analysis, enhancing the reliability of the results[10].

Robust Adjusted Profile Score (RAPS) : Designed to be robust against both systematic and idiosyncratic pleiotropy, RAPS is recommended for analyzing complex traits and diseases[11]

MR-Egger regression method: The MR-Egger intercept was employed to detect potential directional pleiotropy[12]. Given MR-Egger's sensitivity to outliers[13], we calculated Cook 's distance to detect the outlier variants, and then re-ran the MR-Egger analysis after removing the outlier variants[14].

Scatter plot: Scatter plots were utilized to visually assess potential heterogeneity among the estimates of genetic variants, providing insights into the consistency of the data[15].

Funnel plots: These plots were used to test for directional pleiotropy, helping to identify any asymmetry that might indicate bias in the genetic instrument effects[15].

Steiger filtering: To mitigate potential reverse causality, Steiger filtering was applied, ensuring that the genetic instruments used were more strongly associated with the exposure than the outcome[16].

Stricter clumping criteria :To account for the potential impact of linkage disequilibrium, we also adopted stricter clumping criteria (LD $r^2 < 0.01$ and $r^2 < 0.001$).

Multivariate Mendelian randomisation(MR) analysis: Given the establish association between obesity, hypertension, and NAFLD [17,18], we performed a multivariate MR analysis, adjusting for genetically predicted BMI to avoid potential pleiotropy through BMI.

Supplemental Text 4. Details of the sensitivity analysis method for estimating bias and Type I error rate due to the use of genome-wide association study data with sample overlap

To assess the potential bias and Type I error rate resulting from sample overlap between exposure and outcome GWAS datasets, we applied the method proposed by Stephen Burgess[19].

Bias estimation

The bias induced by sample overlap was estimated as:

$$Bias = olsbias \times overlap.prop \times \frac{1}{expf}$$

Type I error rate

The Type I error rate was estimated as:

$$Type1err = 2 - \Phi\left(1.96 + \frac{bias}{\sqrt{var}}\right) - \Phi\left(1.96 - \frac{bias}{\sqrt{var}}\right)$$

where Φ denotes the cumulative distribution function of the standard normal distribution.

Parameter definitions

olsbias

Bias of the observational estimate (ordinary least squares estimate). The observational association was derived from the study by Li et al.[20], which found that each 10 mmHg increase in mean arterial blood pressure is associated with a 15% higher risk of non-alcoholic fatty liver disease (NAFLD) (odds ratio: 1.15). Consequently, a 1 standard deviation increase in systolic blood pressure (20 mmHg) is estimated to increase the risk of NAFLD, with the association coefficientβ calculated as log(1.15)×2 = 0.2795239.

overlap.prop

Proportion of sample overlap between exposure and outcome GWAS datasets. The overlap proportion is 16.0%in the primary analysis and 38.7% in the replication analysis.

expf

Expected value of the F-statistic:

$$expf = \frac{N-K-1}{K} \times \frac{R^2}{1-R^2}$$

N is the sample size. K is number of genetic variants. R^2 is proportion of variance in the exposure explained by the genetic instruments, calculated as [21]:

$$R^2 = \sum_i \frac{2{\beta_i}^2 \times MAF_i(1-MAF_i)}{2{\beta_i}^2 \times MAF_i(1-MAF_i) + se(\beta_i)^2 \times 2N \times MAF_i(1-MAF_i)}$$

Where i represent each genetic variant, β is the genetic association with the exposure measured in standard deviation units of the exposure, MAF is the minor allele frequency, se(β) is the standard error of the effect size, and N is the sample size.

var

Variance of the IV estimate:

$$var = \frac{1}{N \times R^2 \times var_x \times prop.case \times (1-prop.case)}$$

var_x is the variance of the exposure. Because one standard deviation of systolic blood pressure corresponds to 20 mmHg, the variance of the exposure was set to 400.

prop.case is the proportion of cases, which is 0.42 in the primary analysis and 0.0108 in the replication analysis.

Results

In the primary analysis, the estimated bias and Type I error rate due to GWAS sample overlap were 0.0005 and 0.05, respectively.

In the replication analysis, the corresponding values were 0.001 and 0.05.

Supplemental Text 5. Details of the post hoc analysis method using cis-eQTL data from the Adult Genotype-Tissue Expression

We utilised cis-eQTL data from the GTEx Analysis V8 to validate genetic instruments for the target genes of renin-angiotensin system (RAS) inhibitors. The eQTL data were derived from 49 non-diseased tissues in 838 donors. To ensure genetic homogeneity, we included only eQTL data from individuals of European ancestry.

We extracted SNPs within ±500 KB of each target gene and employed GTEx eQTL data to validate these genetic instruments. For eQTLs measured across multiple tissues, we selected the SNP with the lowest P-value across all tissues for analysis. Following a similar approach to the main analysis using eQTL data from eQTLGen, we conducted a two-sample Mendelian randomisation analysis using the Wald method to investigate the association between gene

expression and systolic blood pressure for each SNP. SNPs with P-values below 0.05 were retained as validated genetic instruments for subsequent analyses.

Supplemental Text 6. Details of the colocalisation analysis method for further assessing the Mendelian randomisation assumption

PPARG and JUN, target genes of renin-angiotensin (RAS), showed significant associations with NAFLD risk. Consequently, we performed colocalisation analysis on these two genes to assess whether they share the same causal variants with NAFLD.

We used approximate Bayes factor colocalisation analysis with default parameters, which provided posterior probabilities corresponding to five hypotheses:

H0: no association with either trait in the region

H1: association with trait 1 only

H2: association with trait 2 only

H3: both traits are associated but have distinct causal variants

H4: both traits are associated and share a single causal variant.

We considered a posterior probability of shared causal variants (PPH4) > 0.8 as strong evidence for colocalisation, while a high PPH3 indicated potential confounding due to linkage disequilibrium.

For colocalisation analysis, we used SNPs within two different gene region definitions: (1) the precise gene position as provided by the Ensembl genome browser, and (2) the same gene position with an additional ±500 KB window. Additionally, we conducted colocalisation analysis assuming both single[22] and multiple causal variants[23] to relax the single causal variant assumption.

PART B

Supplementary References

References

1. Rahimi K, Canoy D, Nazarzadeh M, et al. Investigating the stratified efficacy and safety of pharmacological blood pressure-lowering: an overall protocol for individual patient-level data meta-analyses of over 300 000 randomised participants in the new phase of the Blood Pressure Lowering Treatment Trialists' Collaboration (BPLTTC). BMJ Open 2019; 9: e028698.
2. Blood Pressure Lowering Treatment Trialists' Collaboration. Pharmacological blood pressure lowering for primary and secondary prevention of cardiovascular disease across different levels of blood pressure: an individual participant-level data meta-analysis. Lancet 2021; 397: 1625–36.
3. Blood Pressure Lowering Treatment Trialists' Collaboration. Age-stratified and blood-pressure-stratified effects of blood-pressure-lowering pharmacotherapy for the prevention of cardiovascular disease and death: an individual participant-level data meta-analysis. Lancet 2021; 398: 1053–64.
4. Nazarzadeh M, Bidel Z, Canoy D, et al. Blood pressure-lowering treatment for prevention of major cardiovascular diseases in people with and without type 2 diabetes: an individual participant-level data meta-analysis. Lancet Diabetes Endocrinol 2022; 10: 645–54.
5. Canoy D, Copland E, Nazarzadeh M, et al. Antihypertensive drug effects on long-term blood pressure: an individual-level data meta-analysis of randomised clinical trials. Heart 2022; 108: 1281–9.
6. Nazarzadeh M, Canoy D, Bidel Z, et al. The Blood Pressure Lowering Treatment Trialists' Collaboration: methodological clarifications of recent reports. J Hypertens 2022; 40: 847–52.
7. Ettehad D, Emdin CA, Kiran A, et al. Blood pressure lowering for prevention of cardiovascular disease and death: a systematic review and meta-analysis. The Lancet 2016; 387: 957–67.
8. Rücker G, Krahn U, König J, et al. netmeta: Network Meta-Analysis using Frequentist Methods. 2025; published online Feb 6. https://cran.r-project.org/web/packages/netmeta/index.html (accessed March 17, 2025).
9. Bowden J, Davey Smith G, Haycock PC, Burgess S. Consistent Estimation in Mendelian Randomization with Some Invalid Instruments Using a Weighted Median Estimator. Genet Epidemiol 2016; 40: 304–14.
10. Verbanck M, Chen C-Y, Neale B, Do R. Detection of widespread horizontal pleiotropy in causal relationships inferred from Mendelian randomization between complex traits and diseases. Nat Genet 2018; 50: 693–8.
11. Zhao Q, Wang J, Hemani G, Bowden J, Small DS. Statistical inference in two-sample summary-data Mendelian randomization using robust adjusted profile score. The Annals of Statistics 2020; 48: 1742–69.
12. Bowden J, Davey Smith G, Burgess S. Mendelian randomization with invalid instruments: effect estimation and bias detection through Egger regression. International Journal of Epidemiology 2015; 44: 512–25.
13. Burgess S, Thompson SG. Interpreting findings from Mendelian randomization using the MR-Egger method. Eur J Epidemiol 2017; 32: 377–89.
14. Cook RD. Detection of Influential Observation in Linear Regression. Technometrics 1977; 19: 15–8.
15. Burgess S, Bowden J, Fall T, Ingelsson E, Thompson SG. Sensitivity Analyses for Robust Causal Inference from Mendelian Randomization Analyses with Multiple Genetic Variants. Epidemiology 2017; 28: 30.
16. G H, K T, G DS. Orienting the causal relationship between imprecisely measured traits using GWAS summary data. PLoS genetics 2017; 13. DOI:10.1371/journal.pgen.1007081 .
17. Polyzos SA, Kountouras J, Mantzoros CS. Obesity and nonalcoholic fatty liver disease: From pathophysiology to therapeutics. Metabolism - Clinical and Experimental 2019; 92: 82–97.
18. Whelton PK, Carey RM, Aronow WS, et al. 2017 ACC/AHA/AAPA/ABC/ACPM/AGS/APhA/ASH/ASPC/NMA/PCNA Guideline for the Prevention, Detection, Evaluation, and Management of High Blood Pressure in Adults: A Report of the American College of Cardiology/American Heart Association Task Force on Clinical Practice Guidelines. Hypertension 2018; 71: e13–115.
19. Burgess S, Davies NM, Thompson SG. Bias due to participant overlap in two-sample Mendelian randomization. Genet Epidemiol 2016; 40: 597–608.
20. Li X, Yang H, Xie G, Kuang M, Sheng G, Zou Y. Association of mean arterial pressure with non-alcoholic fatty liver disease: results from the NAGALA study. Front Cardiovasc Med 2023; 10.
21. Yarmolinsky J, Bonilla C, Haycock PC, Langdon RJQ, Lotta LA, Langenberg C, Relton CL, Lewis SJ, Evans DM, PRACTICAL Consortium, et al. Circulating selenium and prostate cancer risk: A mendelian randomization analysis. JNCI J Natl Cancer Inst 2018; 110: 1035–1038.
22. Giambartolomei C, Vukcevic D, Schadt EE, et al. Bayesian Test for Colocalisation between Pairs of Genetic Association Studies Using Summary Statistics. PLOS Genet 2014; 10: e1004383.

23. Wallace C. A more accurate method for colocalisation analysis allowing for multiple causal variants. PLOS Genet 2021; 17: e1009440.
24. Appel LJ, Wright JT, Greene T. Intensive blood-pressure control in hypertensive chronic kidney disease. New England Journal of Medicine 2010; 363(26): 2565–6.
25. Schrier RW, Estacio RO, Jeffers B. Appropriate Blood Pressure Control in NIDDM (ABCD) Trial. Diabetologia [Internet] 1996; 39(12): 1646–54. Available from: http://cochranelibrary-wiley.com/o/cochrane/clcentral/articles/933/CN-00134933/frame.html
26. Cushman WC, Evans GW, Byington RP, et al. Effects of intensive blood-pressure control in type 2 diabetes mellitus. New England Journal of Medicine 2010; 362(17): 1575–85.
27. Yusuf S, Healey JS, Pogue J, et al. Irbesartan in patients with atrial fibrillation. New England Journal of Medicine 2011; 364(10): 928–38.
28. Patel A. Effects of a fixed combination of perindopril and indapamide on macrovascular and microvascular outcomes in patients with type 2 diabetes mellitus (the ADVANCE trial): a randomised controlled trial. Lancet 2007; 370(9590): 829–40.
29. Group TAO and C for the ACR, Coordinators TAO and, Antihypertensive T, Treatment L. Major Outcomes in High-Risk Hypertensive Patients Randomized to Angiotensin-Converting Enzyme Inhibitor or Calcium Channel Blocker vs Diuretic. JAMA: The Journal of the American Medical Association 2002; 288(23): 2981–97.
30. Doyle AE. The Australian National blood pressure study. Trends in Pharmacological Sciences 1981; 2(C): 293–6.
31. Wing LMH, Reid CM, Ryan P, et al. A comparison of outcomes with angiotensin-converting-enzyme inhibitors and diuretics for hypertension in the elderly. New England Journal of Medicine 2003; 348(7): 583–92.
32. Dahlöf B, Sever PS, Poulter NR, et al. Prevention of cardiovascular events with an antihypertensive regimen of amlodipine adding perindopril as required versus atenolol adding bendroflumethiazide as required, in the Anglo-Scandinavian Cardiac Outcomes Trial-Blood Pressure Lowering Arm (ASCOT-B. Lancet [Internet] 2005; 366(9489): 895–906.
33. Ruggenenti P, Fassi A, Ilieva AP, et al. Preventing microalbuminuria in type 2 diabetes. New England Journal of Medicine 2004; 351(19): 1941–51.
34. Park S, Yan P, Cerezo C, Jeffers BW. Effect of visit-to-visit blood pressure variability on cardiovascular events in patients with coronary artery disease and well-controlled blood pressure. Journal of the American Society of Hypertension 2016; 10(10): 799–810.
35. Hansson L, Lindholm LH, Niskanen L, et al. Effect of angiotensin-converting-enzyme inhibition compared with conventional therapy on cardiovascular morbidity and mortality in hypertension: the Captopril Prevention Project (CAPPP) randomised trial. Lancet 1999; 353(9153): 611–6.
36. Verdecchia P, Staessen JA, Angeli F, et al. Usual versus tight control of systolic blood pressure in non-diabetic patients with hypertension (Cardio-Sis): an open-label randomised trial. The Lancet 2009; 374(9689): 525–33.
37. Nakao K, Hirata M, Oba K, et al. Role of diabetes and obesity in outcomes of the candesartan antihypertensive survival evaluation in Japan (CASE-J) trial. Hypertension Research 2010; 33(6): 600–6.
38. Ogihara T, Saruta T, Rakugi H, et al. Combinations of olmesartan and a calciumchannel blocker or a diuretic inelderly hypertensive patients: A randomized, controlled trial. Journal of Hypertension 2014; 32(10): 2054–63.
39. Black HR, Elliott WJ, Grandits G, et al. Principal Results of the Controlled Onset Verapamil Investigation of Cardiovascular End Points (CONVINCE) Trial. Journal of the American Medical Association 2003; 289(16): 2073–82.
40. Matsuzaki M, Ogihara T, Umemoto S, et al. Prevention of cardiovascular events with calcium channel blocker-based combination therapies in patients with hypertension: A randomized controlled trial. Journal of Hypertension 2011; 29(8): 1649–59.
41. Marre M, Lievre M, Chatellier G, Mann JFE, Passa P, Ménard J. Effects of low dose ramipril on cardiovascular and renal outcomes in patients with type 2 diabetes and raised excretion of urinary albumin: Randomised, double blind, placebo controlled trial (the DIABHYCAR study). British Medical Journal 2004; 328(7438): 495–9.
42. Koudstaal PJ, Algra A, Pop GA, Kappelle LJ, van Latum JC, van Gijn J. Risk of cardiac events in atypical transient ischaemic attack or minor stroke. The Dutch TIA Study Group. Lancet (London, England) 1992; 340(8820): 630–3.
43. Zanchetti A, Bond MG, Hennig M, et al. Calcium antagonist lacidipine slows down progression of asymptomatic carotid atherosclerosis: Principal results of the European Lacidipine Study on Atherosclerosis (ELSA), a randomized, double-blind, long-term trial. Circulation 2002; 106(19): 2422–7.

44. Fox KM, Bertrand M, Ferrari R, et al. Efficacy of perindopril in reduction of cardiovascular events among patients with stable coronary artery disease: Randomised, double-blind, placebo-controlled, multicentre trial (the EUROPA study). Lancet [Internet] 2003; 362(9386): 782–8. Available from: https://www.ncbi.nlm.nih.gov/pubmed/13678872
45. Amery A, Brixko P, Clement D, et al. Mortality and morbidity results from the European Working Party on High Blood Pressure in the Elderly trial. The Lancet 1985; 325(8442): 1349–54.
46. Kasanuki H, Hagiwara N, Hosoda S, et al. Angiotensin II receptor blocker-based vs. non-angiotensin II receptor blocker-based therapy in patients with angiographically documented coronary artery disease and hypertension: The Heart Institute of Japan Candesartan Randomized Trial for Evaluation in. European Heart Journal 2009; 30(10): 1203–12.
47. Asayama K, Ohkubo T, Metoki H, et al. Cardiovascular outcomes in the first trial of antihypertensive therapy guided by self-measured home blood pressure [Internet]. Hypertension Research 2012; 35(11): 1102–10. Available from: http://cochranelibrary-wiley.com/o/cochrane/clcentral/articles/908/CN-00868908/frame.html
48. Sharma AM, Pischon T, Engeli S. Effect of ramipril on cardiovascular events in high-risk patients. New England Journal of Medicine 2000; 343(1).
49. Beckett NS, Peters R, Fletcher AE, et al. Treatment of hypertension in patients 80 years of age or older. New England Journal of Medicine 2008; 358(18): 1887–98.
50. Lewis EJ, Hunsicker LG, Clarke WR, et al. Renoprotective effect of the angiotensin-receptor antagonist irbesartan in patients with nephropathy due to type 2 diabetes. New England Journal of Medicine 2001; 345(12): 851–60.
51. Brown MJ, Palmer CR, Castaigne A, et al. Morbidity and mortality in patients randomised to double-blind treatment with a long-acting calcium-channel blocker or diuretic in the International Nifedipine GITS study: Intervention as a Goal in Hypertension Treatment (INSIGHT). Lancet 2000; 356(9227): 366–72.
52. Pepine CJ, Handberg EM, Cooper-DeHoff RM, et al. A Calcium Antagonist vs a Non-Calcium Antagonist Hypertension Treatment Strategy for Patients with Coronary Artery Disease the International Verapamil-Trandolapril Study (INVEST): A Randomized Controlled Trial. Journal of the American Medical Association 2003; 290(21): 2805–16.
53. Yui Y, Sumiyoshi T, Kodama K, et al. Comparison of nifedipine retard with angiotensin converting enzyme inhibitors in Japanese hypertensive patients with coronary artery disease: The Japan Multicenter Investigation for Cardiovascular Diseases-B (JMIC-B) randomized trial. Hypertension Research 2004; 27(3): 181–91.
54. Lindholm LH, Ibsen H, Dahlöf B, et al. Cardiovascular morbidity and mortality in patients with diabetes in the Losartan Intervention For Endpoint reduction in hypertension study (LIFE): A randomised trial against atenolol. Lancet 2002; 359(9311): 1004–10.
55. Schrader J, Lüders S, Kulschewski A, et al. Morbidity and mortality after stroke, eprosartan compared with nitrendipine for secondary prevention: Principal results of a prospective randomized controlled study (MOSES). Stroke 2005; 36(6): 1218–24.
56. Randomized double-blind comparison of a calcium antagonist and a diuretic in elderly hypertensives. National Intervention Cooperative Study in Elderly Hypertensives Study Group. Hypertension 1999; 34(5): 1129–33.
57. Hansson L, Hedner T, Lund-Johansen P, et al. Randomised trial of effects of calcium antagonists compared with diuretics and β-blockers on cardiovascular morbidity and mortality in hypertension: The Nordic Diltiazem (NORDIL) study. Lancet 2000; 356(9227): 359–65.
58. Yusuf S, Teo KK, Pogue J, et al. Telmisartan, ramipril, or both in patients at high risk for vascular events. New England Journal of Medicine 2008; 358(15): 1547–59.
59. MacMahon S, Sharpe N, Gamble G, et al. Randomized, placebo-controlled trial of the angiotensin-converting enzyme inhibitor, ramipril, in patients with coronary or other occlusive arterial disease. Journal of the American College of Cardiology 2000; 36(2): 438–43.
60. Braunwald E, Domanski MJ, Fowler SE, et al. Angiotensin-converting-enzyme inhibition in stable coronary artery disease. New England Journal of Medicine 2004; 351(20).
61. Asselbergs FW, Diercks GFH, Hillege HL, et al. Effects of fosinopril and pravastatin on cardiovascular events in subjects with microalbuminuria. Circulation 2004; 110(18): 2809–16.
62. Eleuteri E. Effect of amlodipine on the progression of atherosclerosis and the occurrence of clinical events. Italian heart journal Supplement 2001; 2(1): 85–6.
63. Sacco RL, Diener HC, Yusuf S, et al. Aspirin and extended-release dipyridamole versus clopidogrel for recurrent stroke. New England Journal of Medicine 2008; 359(12): 1238–51.

64. Randomised trial of a perindopril-based blood-pressure-lowering regimen among 6,105 individuals with previous stroke or transient ischaemic attack. Lancet [Internet] 2001; 358(9287): 1033–41. Available from: http://cochranelibrary-wiley.com/o/cochrane/clcentral/articles/696/CN-00356696/frame.html
65. Ogihara T, Nakao K, Fukui T, et al. Effects of candesartan compared with amlodipine in hypertensive patients with high cardiovascular risks: Candesartan antihypertensive survival evaluation in Japan trial. Hypertension 2008; 51(2): 393–8.
66. Wright JT, Williamson JD, Whelton PK, et al. A randomized trial of intensive versus standard blood-pressure control. New England Journal of Medicine 2015; 373(22): 2103–16.
67. Hansson L, Lindholm LH, Ekbom T, et al. Randomised trial of old and new antihypertensive drugs in elderly patients: Cardiovascular mortality and morbidity the Swedish trial in old patients with hypertension-2 study. Lancet 1999; 354(9192): 1751–6.
68. Staessen J, Fagard R, Thijs L, et al. Randomised double-blind comparison of placebo and active treatment for older patients with isolated systolic hypertension. The Systolic Hypertension in Europe (Syst-Eur) Trial Investigators [Internet]. Lancet 1997; 350(9080): 757–64.
69. Telmisartan T, Assessment R. Effects of the angiotensin-receptor blocker telmisartan on cardiovascular events in high-risk patients intolerant to angiotensin-converting enzyme inhibitors: a randomised controlled trial. The Lancet 2008; 372(9644): 1174–83.
70. Tight blood pressure control and risk of macrovascular and microvascular complications in type 2 diabetes: UKPDS 38. UK Prospective Diabetes Study Group. BMJ (Clinical research ed) [Internet] 1998; 317(7160): 703–13. Available from: http://www.ncbi.nlm.nih.gov/pubmed/9732337
71. Ogihara T, Saruta T, Rakugi H, et al. Target blood pressure for treatment of isolated systolic hypertension in the elderly: Valsartan in elderly isolated systolic hypertension study. Hypertension 2010; 56(2): 196–202.
72. Julius S, Kjeldsen SE, Weber M, et al. Outcomes in hypertensive patients at high cardiovascular risk treated with regimens based on valsartan or amlodipine: The VALUE randomised trial. Lancet 2004; 363(9426): 2022–31.
73. Zanchetti A, Agabiti Rosei E, Dal Palù C, Leonetti G, Magnani B, Pessina A. The Verapamil in Hypertension and Atherosclerosis Study (VHAS): Results of long-term randomized treatment with either verapamil or chlorthalidone on carotid intima-media thickness. Journal of Hypertension 1998;16(11):1667–76.
74. Langford HG, Stamier J, Wassertheil-Smoller S, Prineas RJ. All-cause mortality in the hypertension detection and follow-up program: Findings for the whole cohort and for persons with less severe hypertension, with and without other traits related to risk of mortality. Progress in Cardiovascular Diseases 1986;29(3 SUPPL. 1):29–54.

PART C

Supplementary Tables

Table S1. Details of the genome-wide association studies from which summary-level data were used for Mendelian randomisation analyses

Trait	Data sourses	Sample size a	Population	Pubmed ID b
Systolic blood pressure	UK Biobank, ICBP, Million Veteran Program, and BioVU	1,028,980	European	38689001
NAFLD used for primary analysis	Million Veteran Program	68,725 / 95,472	European	35654975
NAFLD used for replication analysis	UK Biobank, Estonian Biobank, eMERGE, and FinnGen	8,434 / 770,180	European	34841290
Alanine aminotransferase (ALT) as a proxy for NAFLD	UK Biobank	389,733	European	34017140
Aspartate aminotransferase (AST) as a proxy for NAFLD	UK Biobank	388,490	European	34017140
Coronary heart disease	DeCODE, EPIC-CVD, GerMIFS5, GerMIFS6, GerMIFS7, Greek Coronary Disease cohort, HUNT, Mass General Brigham Biobank, TIMI, UK Biobank	181,522 / 984,168	Predominantly European ancestry (>95%)	36474045
BMI	UK Biobank	461,460	European	ukb-b-19953

a. For continuous variables, the sample size refers to the sample size of the corresponding GWAS data. For binary variables, the sample size is represented as "number of cases/number of controls”.

b. The PubMed ID is used to identify the original publication that introduces the GWAS dataset, which can be referred to for accessing the GWAS data. The GWAS data for body mass index (BMI) that we used has not yet been published in an introductory paper but is available in the IEU Open GWAS project under the GWAS ID "ukb-b-19953."

Table S2. Details of 51 randomised trials from the Blood Pressure Lowering Treatment Trialists' Collaboration (BPLTTC) used to investigate the effects of pharmacological blood pressure-lowering treatments on the risk of coronary heart disease using an individual participant data meta-analysis design

Number	Trial name	Type of trial	Inclusion criteria	Exclusion criteria	Age (years), mean (SD)	Mean of follow-up duration (year)	Intervention (n)	Comparator (n)	SBP difference (mmHg) excluding first 12 months *	DBP difference (mmHg) excluding first 12 months*
1.	AASK[24]	Intensive	Age 18-70 years, African American, hypertension, renal disease (GFR=20-65 ml/min per 1.73m2)	DBP <95 mmHg, diabetes, urine protein:creatinine ratio >25, recent malignant hypertension, secondary hypertension, non-blood pressure-related CKD, serious systemic disease, HF	54 (11)	4.8	More intensive (540)	Less intensive (554)	13	7.9
2.	ABCD[25]	Intensive	Age 40-74 years, type 2 diabetes mellitus, DBP ≥80 mmHg, not on antihypertensive treatment	Recent CAD or CeVD, heart failure, renal disease	58 (8)	4.7	More intensive (474)	Less intensive (476)	7.7	6.9
3.	ACCORD[26]	Intensive	Age ≥40 years with CVD or ≥50 years with substantial atherosclerosis, diagnosis/history of type 2 diabetes mellitus, HbA1c ≥7.5%, albuminuria, LVH or ≥2 CVD risk factors (dyslipidaemia, hypertension, smoking, obesity); SBP 130-180 mmHg and taking ≤ 3 antihypertensive drugs, 24-hour protein excretion rate <1g	Body mass index ≥45 kg/m2, serum creatinine ≥ 132.6 µmol/l and other serious illness	63 (7)	4.7	More intensive (2362)	Less intensive (2371)	13.9	1.8
4.	ACTIVE I[27]	Placebo-controlled	Atrial fibrillation, ≥1 risk factor (age ≥75 years, on antihypertensive treatment, history of stroke, TIA or non-CNS embolism, LVEF <45%, PVD, or age 55-74 years with either CAD or diabetes)	Use of anticoagulant, peptic ulcer disease in past 6 months, history of intracerebral haemorrhage, thrombocytopaenia or mitral stenosis	70 (10)	4.1	ARB (3058)	Placebo (3076)	2.6	1.4
5.	ADVANCE[28]	Placebo-controlled	Age ≥55 years, type 2 diabetes mellitus (diagnosed aged ≥30 years), ≥1 major CVD or ≥1 CVD risk factor (microvascular disease, smoking, dyslipidaemia, microalbuminuria, Diagnosis of type 2 diabetes mellitus for ≥10 years, age ≥65 years)	Definite indication for, or contraindication to, any of the study treatments or the HbA1c target ≤6·5%, indication for long-term insulin therapy at baseline	66 (6)	4.2	ACEI and Diuretic (5569)	Placebo (5571)	5.4	2.1
6.	ALLHAT[29]	Drug classes comparison	Age ≥55 years, stage 1 or 2 hypertension plus ≥1 risk factor (MI or stroke >6 months, LVH, diagnosis/history of type 2 diabetes mellitus, smoking, HDL <0.91 mmol/l), other atherosclerotic CVD	Symptomatic or hospitalisation for HF, LVEF <35%	67 (8)	4.8	Diuretic (15255)	ACEI, CCB and Alpha-blockers (27163)	2	0.1
7.	ANBP[30]	Placebo-controlled	Age 30-69 years with mild hypertension (DBP 95-110 mmHg and SBP <200	Antihypertensive treatment in past 3 months, recent angina or MI, stroke,	50 (9)	3.6	Diuretic (1721)	Placebo (1706)	7.5	3.8

Number	Trial name	Type of trial	Inclusion criteria	Exclusion criteria	Age (years), mean (SD)	Mean of follow-up duration (year)	Intervention (n)	Comparator (n)	SBP difference (mmHg) excluding first 12 months *	DBP difference (mmHg) excluding first 12 months*
			mmHg)	hormone therapy, asthma, diabetes, gout, serious disease, tricyclic antidepressant use						
8.	ANBP2[31]	Drug classes comparison	Age 65-84 years, SBP ≥160 mmHg or DBP ≥90 mmHg (if SBP ≥140 mmHg), no recent CVD	Serious illness, plasma creatinine >221 µmol/l, malignant hypertension, dementia	73 (5)	4.1	Diuretic (3039)	ACEI (3044)	0.9	0
9.	ASCOT-BPLA[32]	Drug classes comparison	Age 40-79 years, untreated (SBP ≥160 or DBP ≥100 mmHg) or treated hypertension (SBP ≥140 or DBP ≥90 mmHg), ≥3 CVD risk factors (documented LVH, abnormal ECG, type 2 diabetes mellitus, PAD, previous stroke or TIA, male sex, age ≥55 years, microalbuminuria or proteinuria, smoking, TC:HDL ≥6, family history of premature coronary heart disease	Previous MI, current treatment for angina, recent CeVD, fasting triglycerides >4.5 mmol/l, heart failure, arrhythmia, haematological or biochemical abnormality at screening	63 (9)	5.3	CCB-based (9639)	Beta-blocker based (9618)	2.2	2
10.	BENEDICT[33]	Placebo-controlled	Age ≥40 years, untreated SBP ≥130 / DBP ≥85 mmHg or needing treatment to attain below these levels, type 2 diabetes mellitus for <25 years, urinary albumin excretion rate <20 mcg/min, serum creatinine ≤133 µ mol/l	HbA1c ≥11%, nondiabetic renal disease	62 (8)	3.1	ACEI, CCB and ACEI/CCB (907)	Placebo (302)	2	1.3
11.	CAMELOT[34]	Placebo-controlled	Age 30-79 years, coronary artery stenosis >20% by angiography, DBP <100 mmHg	Left middle coronary artery obstruction >50%, LVEF <40%, heart failure	58 (10)	1.6	CCB and ACEI (1340)	Placebo (657)	5.3	3.3
12.	CAPPP[35]	Drug classes comparison	Age 25-66 years, DBP ≥100 mmHg on two occasions	Secondary hypertension, serum creatinine >150 µ mol/l, condition requiring beta-blocker treatment	52 (8)	5.8	Beta-blocker and/or Diuretic (5493)	ACEI (5492)	2.2	1.3
13.	CARDIO-SIS[36]	Intensive	Age ≥55 years, SBP ≥150 mmHg, taking antihypertensive drug ≥12 weeks, ≥1 CV risk factor (smoking, dyslipidaemia, family history of premature CVD, prior TIA or stroke, established CAD or PAD	Fasting blood glucose ≥126 mg/dL (≥7 mmol/l), known diabetes, serious conditions, renal disease, valvular heart disease, left ventricular hypertrophy, atrial fibrillation, substance misuse	67 (7)	4.7	More intensive (558)	Less intensive (553)	3.8	1.5
14.	CASE-J[37]	Drug classes comparison	Age 20-85 years, ≥1 high-risk factor: SBP ≥180 or DBP ≥110 mmHg, type 2 diabetes mellitus, history of angina pectoris, MI, stroke, TIA >6 months	BP ≥200/120 mmHg, type 1 diabetes, HF, LEF <40%, atrial fibrillation, cancer	64 (11)	3.1	CCB (2349)	ARB (2354)	1.7	0.9
15.	COLM[38]	Drug classes comparison	Age 65-84 years, hypertension (treated: blood pressure ≥140/90 mmHg; untreated: blood pressure ≥160/100 mmHg), CVD history or CVD risk factors including diabetes and dyslipidaemia	Secondary/malignant hypertension, recent major CVD, revascularisation, angina pectoris hospitalisation or severe heart failure, atrial fibrillation, hepatic or renal dysfunction	74 (5)	3	ARB and Diuretic (2573)	ARB and CCB (2568)	0.3	0.4
16.	CONVINCE[39]	Drug classes comparison	Age ≥55 years, hypertension, ≥1 CVD risk factor (e.g., diabetes, smoking)	HF, dysrhythmia, secondary hypertension, recent MI or stroke, renal disease, other serious disease, BP ≥190/110 mmHg	66 (7)	2.8	CCB (8179)	Beta-blocker or Diuretic (8297)	0	0.7

Number	Trial name	Type of trial	Inclusion criteria	Exclusion criteria	Age (years), mean (SD)	Mean of follow-up duration (year)	Intervention (n)	Comparator (n)	SBP difference (mmHg) excluding first 12 months *	DBP difference (mmHg) excluding first 12 months*
				without treatment						
17.	COPE[40]	Drug classes comparison	Age 40-85 years, blood pressure ≥140/90 mmHg	SBP ≥200 or DBP ≥120 mmHg, secondary hypertension, type 1 diabetes or type 2 diabetes requiring insulin treatment, recent CVD or revascularisation, heart failure, atrial fibrillation/flutter, hepatic or renal dysfunction, congenital or rheumatic heart disease, cancer	64 (11)	3.6	CCB/Diuretic and CCB/ Beta-blocker (2183)	CCB and ARB (1110)	0.4	0.4
18.	DIABHYCAR[41]	Placebo-controlled	Age ≥50 years, type 2 diabetes mellitus, urinary albumin excretion ≥20 mg/l in two consecutive urine samples	Serum creatinine >150 micro mol/l, use of insulin, ACEI or ARB, heart failure, recent MI, urinary tract infection	65 (8)	3.9	ACEI (2443)	Placebo (2469)	0.9	0.4
19.	Dutch TIA Trial[42]	Placebo-controlled	TIA or non-disabling ischaemic stroke (Rankin Scale ≤3) in past 3 months	Cerebral ischaemia from identifiable causes other than arterial thrombosis or embolism	64 (10)	2.3	Beta-blocker (732)	Placebo (741)	3.1	2
20.	ELSA[43]	Drug classes comparison	Age 45-79 years, blood pressure 150-210/95-115 mmHg	Recent MI or stroke, and type 2 diabetes mellitus	57 (7)	3.4	CCB (1177)	Beta-blocker (1157)	0.8	0.4
21.	EUROPA[44]	Placebo-controlled	Age ≥18 years, documented MI >3 months before screening, revascularisation >6 months before screening, >70% coronary obstruction	HF, hypotension, uncontrolled hypertension, renal insufficiency, serum potassium >5.5 mmol/L	61 (9)	4.2	ACEI (6110)	Placebo (6108)	4.6	2.2
22.	EWPHE[45]	Placebo-controlled	Age ≥60 years, blood pressure 160-239/90-119 mmHg	Curable causes of high BP, retinopathy, heart failure, stroke history, hepatitis/cirrhosis, gout, malignancy, diabetes requiring insulin treatment	71 (8)	4.6	Diuretic (416)	Placebo (424)	22.4	9.5
23.	HIJ-CREATE[46]	Drug classes comparison	Age 20-80 years, CAD hospitalisation and hypertension (blood pressure ≥140/90 mmHg or antihypertensive treatment use)	Secondary hypertension, recent AMI or CeVD, severe aortic valve stenosis, cardiomyopathy, serum creatinine >2 mg/dl, serum potassium >5 mmol/l, hepatic dysfunction, malignancy	65 (9)	4	ARB (1024)	non-ARB (1025)	0.4	0.5
24.	HOMED-BP[47]	Intensive	Self-measured SBP 135-179 mmHg or DBP 85-119 mmHg, but not if DBP <65 mmHg or SBP <110 mmHg (clinic SBP <220 mmHg and DBP <125 mmHg)	None specified	60 (10)	4.9	More intensive (1759)	Less intensive (1759)	2	0.9
25.	HOPE[48]	Placebo-controlled	Age ≥55 years, CAD, stroke, PVD or diabetes, plus ≥1 risk factor (hypertension, dyslipidaemia, smoking, or documented microalbuminuria)	HF, LEF <40%, using ACEI or vitamin E, uncontrolled hypertension, nephropathy, or recent MI or stroke	66 (7)	4.5	ACEI (4645)	Placebo (4652)	3	1.4
26.	HYVET[49]	Placebo-controlled	Age ≥80y years, sustained SBP ≥160 mmHg	Accelerated or secondary hypertension, recent haemorrhagic stroke, HF, serum creatinine >150 micro mol/l, serum potassium <3.5 or >5.5 mmol/l, gout, and dementia	84 (3)	2.1	Diuretic (1933)	Placebo (1912)	13.1	5.1
27.	IDNT[50]	Placebo-controlled	Age 30-70 years, type 2 diabetes,	Age of onset type 2 diabetes mellitus <20	59 (8)	2.6	ARB and CCB (1143)	Placebo (568)	2.8	2.8

Number	Trial name	Type of trial	Inclusion criteria	Exclusion criteria	Age (years), mean (SD)	Mean of follow-up duration (year)	Intervention (n)	Comparator (n)	SBP difference (mmHg) excluding first 12 months *	DBP difference (mmHg) excluding first 12 months*
			hypertension (blood pressure ≥135/85 mmHg or taking anti-hypertensive drug), proteinuria, serum creatinine (µmol/l): 88 to 265 (women) or 106 to 265 (men)	years, type 1 diabetes mellitus, absolute requirement for an ACEI, ARB or CCB, cardiovascular disease (unstable angina, myocardial infarction, coronary artery bypass graft surgery or percutaneous transluminal coronary angioplasty within 3 months of study entry, New York Heart Association class III or IV HF, transient ischaemic attack within 6 months of study entry, stroke within 3 months of study entry), and serum potassium outside of normal range						
28.	INSIGHT[51]	Drug classes comparison	Age 55-80 years, hypertensive (SBP ≥150 or DBP ≥95 mmHg, or SBP ≥160 mmHg), ≥1 other risk factor (TC ≥6.43 mmol/l, smoking, family history of premature MI, CAD, other CVD	None specified	65 (6)	2.8	Diuretic (3164)	CCB (3157)	1.1	0.9
29.	INVEST[52]	Drug classes comparison	Age ≥50 years, documented CAD, essential hypertension requiring drug therapy, heart failure Class I-III	Patients taking beta-blocker within two weeks of randomisation or for recent MI	66 (10)	2.8	CCB (10648)	non-CCB (10672)	0.1	0.2
30.	JMIC-B[53]	Drug classes comparison	Age <75 years, hypertension (blood pressure ≥160/≥95 mmHg or both SBP ≥ 150 and DBP ≥90 mmHg, or antihypertensive treatment), CAD or meeting both criteria: history of >2 anginal attacks per week with stable frequency and ST-segment depression of ≥1 mm on stress test (or detection of MI with myocardial scintigraphy)	MI, unstable angina, DBP ≥120 mmHg, secondary hypertension, symptomatic CeVD, HF, atrial fibrillation/arrhythmias, renal or hepatic dysfunction, uncontrollable diabetes and familial hypercholesterolaemia	65 (85)	2.3	CCB (828)	ACEI (822)	2	1.5
31.	LIFE[54]	Drug classes comparison	Age 55-80 years, hypertension (SBP 160-200 mmHg; DBP 95-115 mmHg), electrocardiogram signs of LVH	Secondary hypertension, recent MI or stroke, angina pectoris requiring treatment, HF or LEF ≤ 40%	67 (7)	4.9	ARB (4605)	Beta-blocker (4588)	1.2	0.5
32.	MOSES[55]	Drug classes comparison	Hypertension requiring treatment, documented TIA, ischaemic stroke or cerebral haemorrhage	Internal carotid artery occlusion or stenosis >70%, HF, age >85 years, on anticoagulant for cardiac arrhythmia, high-grade aortic or mitral valve stenosis, unstable angina	68 (10)	3.3	CCB (671)	ARB (681)	1.5	0.5
33.	NICS-EH[56]	Drug classes comparison	Age ≥60 years, SBP 160-220 mmHg and DBP <115 mmHg and no cardiovascular complications	None specified	70 (7)	3.2	Diuretic (214)	CCB (215)	0.3	0.7
34.	NORDIL[57]	Drug classes comparison	Age 50-74 years, untreated hypertension (DBP ≥100 mmHg on two occasions); if previously treated, DBP ≥100 mmHg on two consecutive visits at one week apart during run-in period and no treatment was	Age <50 or ≥70y, bradycardia, secondary hypertension, atrial fibrillation, recent CeVD or MI, HF	60 (7)	4.2	Beta-blocker and/or Diuretic (5471)	CCB (5410)	3.3	0.1

Number	Trial name	Type of trial	Inclusion criteria	Exclusion criteria	Age (years), mean (SD)	Mean of follow-up duration (year)	Intervention (n)	Comparator (n)	SBP difference (mmHg) excluding first 12 months *	DBP difference (mmHg) excluding first 12 months*
			given							
35.	ONTARGET[58]	Drug classes comparison	CAD, PAD, CeVD or diabetes with end-organ damage	HF, pericarditis, CHD, unexplained syncope, planned revascularisation <3 months of consent, uncontrolled hypertension, heart transplant, subarachnoid haemorrhage, renal artery disease, proteinuria, hepatic dysfunction, volume or sodium depletion, primary hyper-aldosteronism, hereditary fructose intolerance, other serious conditions	67 (7)	4.8	ARB/ACEI (8502)	ACEI and ARB (17118)	1.9	1
36.	PART 2[59]	Placebo-controlled	Age ≤75 years, diagnosis (in past 5 year) of MI, documented CAD, TIA or intermittent claudication	HF, serious nonvascular disease, SBP >160 mmHg, DBP >100 mm Hg, DBP <100 mmHg during pre-randomisation run-in period	60 (8)	4.6	ACEI (308)	Placebo (309)	6.5	3.6
37.	PEACE[60]	Placebo-controlled	Age ≥50 years, documented CAD	Unstable angina, severe valvular heart disease, recent revascularisation, planned elective revascularisation, limited 5-year survival, serum creatinine >177 micro mol/l, serum potassium >5.5 mmol/l	64 (8)	4.7	ACEI (4158)	Placebo (4132)	3	1.5
38.	PREVEND IT[61]	Placebo-controlled	Microalbuminuria, SBP <160/100 mmHg (no previous antihypertension treatment)	Creatinine clearance <60% of normal age-adjusted value	51 (12)	3.8	ACEI (431)	Placebo (433)	5.6	2.8
39.	PREVENT[62]	Placebo-controlled	Age 30-80 years, documented CAD, DBP <95 mmHg, cholesterol <325 mg/dl, fasting blood glucose <200 mg/dl	Contraindication for dihydropyridines, uncontrolled hypertension, diabetes and other major illness	57 (10)	3	CCB (417)	Placebo (408)	6.1	3.3
40.	PROFESS[63]	Placebo-controlled	Age ≥55 years with ischaemic stroke <90 days before randomisation (later modified to include age 50 to 54 years or had stroke 90 to 120 days before randomisation if with ≥2 additional risk factors: diabetes, hypertension, smoker, obesity previous CVD, end-organ damage or hyperlipidaemia) and remained stablea	Haemorrhagic stroke, severe disability after the qualifying stroke, contraindication to treatments	66 (8)	2.5	ARB (9873)	Placebo (9925)	3.4	3.4
41.	PROGRESS[64]	Placebo-controlled	Stroke or TIA in past 5 years	Indication or contraindication for ACEI	64 (10)	3.9	ACEI and/or Diuretic (3051)	Placebo (3054)	9.2	4
42.	SHEP[65]	Placebo-controlled	Age ≥60 years, isolated systolic hypertension (BP 160-219/<90 mmHg, not on treatment)	Major CVD, cancer, alcoholic liver disease, renal dysfunction, competing risk of SHEP primary endpoint or presence of medical management exclusions	72 (7)	5	Beta-blocker and Diuretic (2365)	Placebo (2371)	12.8	4.2
43.	SPRINT[66]	Intensive	Age ≥50 y years, SBP 130-180 mmHg, increased CVD risk (clinical/subclinical CVD other than stroke, CKD excluding polycystic kidney disease and with eGFR of 20-60 ml/min/1.73m2 body surface area, 10-year Framingham CVD risk	Prior stroke	68 (9)	3	More intensive (4678)	Less intensive (4683)	14.9	7.7

Number	Trial name	Type of trial	Inclusion criteria	Exclusion criteria	Age (years), mean (SD)	Mean of follow-up duration (year)	Intervention (n)	Comparator (n)	SBP difference (mmHg) excluding first 12 months *	DBP difference (mmHg) excluding first 12 months*
			≥15%, age ≥75y)							
44.	STOP Hypertension-2[67]	Drug classes comparison	Aged 70-84 years, SBP ≥180 mmHg and/or DBP ≥105 mmHg	Not specified	76 (4)	4.5	Beta-blocker and/or Diuretic (2213)	ACEI and CCB (4401)	2.1	0.3
45.	SYST-EUR[68]	Placebo-controlled	Age ≥60 years, sitting SBP 160-219 mmHg, sitting DBP <95 mmHg, and standing SBP ≥140 mmHg	Secondary hypertension, retinal haemorrhage/papilloedema, HF, dissecting aortic aneurysm, serum creatinine ≥180 micro mol/l, recent severe nosebleeds, stroke or MI, dementia, disorders prohibiting standing position, severe CVD/non-CVD	70 (7)	2.6	CCB (2398)	Placebo (2297)	10.1	4
46.	TRANSCEND[69]	Placebo-controlled	Intolerant to ACEI and with established CAD, PVD, CeVD or diabetes with end-organ damage	HF, valvular/cardiac outflow tract obstruction, pericarditis, congenital heart disease, unexplained syncope, recent revascularisation, SBP >160 mmHg, heart transplantation, subarachnoid haemorrhage, significant renal stenosis, renal or hepatic dysfunction	68 (7)	4.9	ARB (2954)	Placebo (2972)	4.5	2.2
47.	UKPDS[70]	Intensive	Age 25-65 years, newly-diagnosed diabetes, and hypertension (untreated: SBP ≥160 mmHg and/or DBP ≥90 mmHg; treated: SBP ≥150 mmHg and/or DBP ≥85 mmHg)	Ketonuria, recent MI, angina, HF, >1 major vascular episode, serum creatinine >15 micro mol/, retinopathy, malignant hypertension, uncorrected endocrine abnormality, severe concurrent illness	56 (8)	7.9	More intensive (758)	Less intensive (390)	11.2	1.2
48.	VALISH[71]	Intensive	Age ≥70 to <85 years, isolated hypertension (SBP >160 mmHg and DBP <90 mmHg)	Secondary or malignant hypertension, BP ≥200/≥ 90 mmHg, recent CeVD or MI, recent/planned revascularisation, HF, aortic stenosis, valvular heart disease, atrial fibrillation/flutter, serious arrhythmia, renal/liver dysfunction	76 (4)	2.6	More intensive (1545)	Less intensive (1534)	5	1.8
49.	VALUE[72]	Drug classes comparison	Age ≥50 years, hypertension, CVD, CVD risk factors (male sex, age >50 years, diabetes, current smoking, high cholesterol, LVH, proteinuria, serum creatinine 150 to 265 micro mol/l)	Renal artery stenosis, recent CAD or CeVD, severe hepatic disease or chronic renal failure, HF, on monotherapy with beta-blocker for CAD and hypertension	67 (8)	4.2	CCB-based (7596)	ARB-based (7649)	1.6	1.3
50.	VHAS[73]	Drug classes comparison	Age 40-65 years, BP ≥160/95 mmHg	Secondary hypertension, recent stroke or TIA, CAD, PAD, bradycardia, arrhythmias, HF, renal or hepatic dysfunction, hyperuricaemia, hypokalemia, type 1 diabetes mellitus, familial dyslipidemia, serious concomitant disease	54 (7)	1.7	Diuretic (707)	CCB (707)	1.7	1.3
51.	HDFP[74]	Intensive	Ages 30-69 years, hypertension, DBP home readings and clinic readings ≥ 95 mmHg and 90 mmHg, respectively	Bedfast or institutionalized persons	50.8	7.2	More intensive (5553)	Less intensive (5387)	9.9	4.9

* Estimated through a one-stage individual patient data mta-analysis approach, and applied linear mixed models to estimate the effect of treatment on blood pressure between comparison arms, SD: standard deviation, SBP: systolic blood pressure, DBP: diastolic blood pressure, eGFR: estimated glomerular filtration rate, CVD: cardiovascular diseases, LVH: left ventricular hypertrophy, TIA: transient ischaemic attack, CNS: central nervous system, LVEF: left ventricular ejection fraction, PVD: peripheral vascular disease, CAD: coronary artery disease, MI: myocardial infarction, HDL: high-density lipoprotein, ECG: electrocardiography, TC: total cholesterol, CeVD: cerebrovascular disease, CHD: coronary artery disease, CKD: coronary artery disease, ACEI: Angiotensin-converting enzyme inhibitors, ARB: angiotensin receptor blockers, CCB: calcium channel blocker, AASK: African American Study of Kidney Disease and Hypertension; ABCD: Appropriate Blood Pressure Control in Diabetes, ACCORD: Action to Control Cardiovascular Risk in Diabetes blood pressure trial; ACTIVE I: Atrial Fibrillation Clopidogrel Trial with Irbesartan for Prevention of Vascular Events, ADVANCE: Action in Diabetes and Vascular disease: preterAx and diamicroN-MR Controlled Evaluation, ALLHAT: Antihypertensive and Lipid Lowering Treatment to Prevent Heart Attack Trial, ANBP: Australian National Blood Pressure Study, ANBP2: Second Australian National Blood Pressure Study, ASCOT-BPLA: Anglo-Scandinavian Cardiac Outcomes Trial-Blood Pressure Lowering Arm, BENEDICT: BErgamo NEphrologic DIabetes Complications Trial, CAMELOT: Comparison of Amlodipine vs Enalapril to Limit Occurrences of Thrombosis, CAPPP: Captopril Prevention Project, Cardio-Sis: Studio Italiano Sugli Effetti Cardiovascolari del Controllo della Pressione Arteriosa Sistolica, CASE-J: Candesartan Antihypertensive Survival Evaluation in Japan Trial, COLM: Combination of OLMesartan study, CONVINCE: Controlled ONset Verapamil INvestigation of Cardiovascular Endpoints trial, COPE: Combination Therapy of Hypertension to Prevent Cardiovascular Events, DIABHYCAR: Noninsulin-dependent diabetes, hypertension, microalbuminuria or proteinuria, cardiovascular events, and ramipril, Dutch TIA Trial: Dutch Transient Ischemic Attack Trial, E-COST: Efficacy of Candesartan on Outcome in Saitama Trial, ELSA: European Lacidipine Study on Atherosclerosis, EUROPA: EUropean trial on Reduction Of cardiac events with Perindopril in patients with stable coronary Artery disease, EWPHE: European Working Party on High Blood Pressure in the Elderly, HIJ-CREATE: Heart Institute of Japan Candesartan Randomized Trial for Evaluation in Coronary Artery Disease, HOMED-BP: Hypertension Objective Treatment Based on Measurement by Electrical Devices of Blood Pressure, HOPE: Heart Outcomes Prevention Evaluation, HYVET: Hypertension in the Very Elderly Trial, IDNT: Irbesartan Diabetic Nephropathy Trial, INSIGHT: International Nifedipine GITS study: Intervention as a Goal in Hypertension Treatment, INVEST: International Verapamil-Trandolapril Study, JMIC-B: Japan Multicenter Investigation for Cardiovascular Diseases- B, LIFE: Losartan Intervention For Endpoint reduction, MOSES: Morbidity and Mortality After Stroke, Eprosartan Compared With Nitrendipine for Secondary Prevention, NICS-EH: National Intervention Cooperative Study in Elderly Hypertensives, NORDIL: Nordic Diltiazem Study, ONTARGET: Ongoing Telmisartan Alone and in Combination with Ramipril Global Endpoint Trial, PART 2: Prevention of Atherosclerosis with Ramipril Trial, PEACE: Prevention of Events with Angiotensin Converting Enzyme Inhibition, PREVEND IT: Prevention of Renal and Vascular Endstage Disease Intervention Trial, PREVENT: Prospective Randomized Evaluation of the Vascular Effects of Norvasc Trial, PROFESS: Prevention Regimen for Effectively Avoiding Second Strokes, PROGRESS: Perindopril Protection Against Recurrent Stroke Study, SHEP: Systolic Hypertension in the Elderly Program, SPRINT: Systolic Blood Pressure Intervention Trial, STOP: Hypertension-2 Swedish Trial in Old

Table S3. Characteristics of randomised controlled trials included in the network meta-analysis to investigate the effect of each antihypertensive drug class versus placebo on the risk of coronary heart

Study Name	Journal	Year of Publication	Characteristics of Patients	Intervention_drug	Comparator_drug	Num in Treat	Num in Control	Duration	CHD in Treat	CHD in Control
AASK	JAMA	2002	HTN Renal dx(African-Americans,with DBP >94mmHg + GFR between 20 and 65 ml/min per 1.73m2)	ACEi	BB	436	441	4.1	19	18
AASK	JAMA	2002	HTN Renal dx(African-Americans,with DBP >94mmHg + GFR between 20 and 65 ml/min per 1.73m2)	CCB	BB	217	441	3	5	18
AASK	JAMA	2002	HTN Renal dx (African-Americans with DBP >94mmHg + GFR between 20 and 65 ml/min per 1.73m2)	ACEi	CCB	436	217	3	19	5
ABCD-H	NEJM	1998	HTN DM2 (DBP≥90mmHg)	CCB	ACEi	235	235	5	25	5
ABCD-N 1	Kidney Int	2002	DM2 Normotensive (<140/90mmHg)	Com	Placebo	237	243	5.3	19	15
ABCD-N 2	Kidney Int	2002	DM 2 normotensive (<140/90mmHg)	CCB	ACEi	234	246	5.3	18	16
ACCOMPLISH	NEJM	2008	HTN + high risk of CVD events	CCB	Diu	5744	5762	3	125	159
ACTION	Lancet	2004	HTN + angina + hx MI/proven CAD	CCB	Placebo	3825	3840	4.9	320	296
ACTIVE I	NEJM	2011	AF + Hx of risk factors for stroke + SBP≥110	ARB	Placebo	4518	4498	4.1	143	135
ADVANCE	Lancet	2007	DM 2 + hx of major CVD disease or ≥ risk factor for CVD	Com	Placebo	5569	5571	4.3	265	294
AIRE	Lancet	1993	AMI+HF	ACEi	Placebo	1004	982	1.25	68	71
ALLHAT	JAMA	2002	HTN +≥1 CHD risk factor+ ≥55yrs	CCB	Diu	9048	15255	4.9	798	1362
ALLHAT	JAMA	2002	HTN +≥1 CHD risk factor+ ≥55yrs	ACEi	Diu	9054	15255	4.9	796	1362
ALLHAT	JAMA	2002	HTN +≥1 CHD risk factor+ ≥55yrs	CCB	ACEi	9048	9054	4.9	798	796
ALTITUDE	NEJM	2012	DM2 + (≥35yrs and evidence of microalbuminuria, macroalbuminuria, or CVD)	RENi	Placebo	4274	4287	2.7	147	142
ANBP2	NEJM	2003	HTN Elderly (65-84 yrs, SBP ≥160 or DBP ≥90)	ACEi	Diu	3044	3039	4.1	58	82
ANBPS	Lancet	1980	mild HTN	Com	Placebo	1721	1706	4	33	33
APSIS	Eur Heart J	1996	stable angina pectoris (<70yrs)	BB	CCB	406	403	3.4	29	25
ASCOT	Lancet	2005	HTN + ≥3 other cardiovascular risk factors, but no previous history of CHD or HF	CCB	BB	9639	9618	5.5	390	444
BCAPS	Circulation	2001	plaque in the right carotid artery but with no symptoms of carotid artery disease	BB	Placebo	396	397	3	3	5
BEST	NEJM	2001	HF (NYHA III or IV) + LVEF≤35%	BB	Placebo	1354	1354	2	10	13
BHAT	JAMA	1982 & 1983	AMI (hospitalised)	BB	Placebo	1916	1921	2.1	192	249
CAMELOT	JAMA	2004	CAD (>20% stenosis by coronary angiography) + DBP< 100 mm Hg	CCB	ACEi	663	673	2	14	11

Study Name	Journal	Year of Publication	Characteristics of Patients	Intervention_drug	Comparator_drug	Num in Treat	Num in Control	Duration	CHD in Treat	CHD in Control
CAMELOT	JAMA	2004	CAD (>20% stenosis by coronary angiography) + DBP< 100 mm Hg	CCB	Placebo	663	655	2	14	19
CAMELOT	JAMA	2004	CAD (>20% stenosis by coronary angiography) + DBP< 100 mm Hg	ACEi	Placebo	673	655	2	11	19
CAPPP	Lancet	1999	DBP ≥ 100mmHg	ACEi	Com	5492	5493	6.1	162	161
Capricorn	Lancet	2001	LVD post AMI	BB	Placebo	975	984	1.3	34	57
CASE-J	Hypertension Research	2008	HTN + ≥1 risk factor	ARB	CCB	2354	2349	3.2	17	18
CHARM	Lancet	2003	CHF (all LVEF subgroups)	ARB	Placebo	3803	3796	3.14	176	190
CIBIS-2	Lancet/Eur J Heart Failure	1999/2001	CHF (NYHA III & IV) with EF ≤35% on standard treatment with ACE-inhibitors and diuretics	BB	Placebo	1327	1320	1.3	7	8
CONSENSUS II	NEJM	1992	AMI + BP>100/60	ACEi	Placebo	3044	3046	0.5	271	268
CONVINCE	JAMA	2003	HTN + ≥1 risk factor	CCB	Com	8179	8297	3	133	166
CRIS	AJC	1996	AMI	CCB	Placebo	531	542	1.96	39	49
DAVIT 2	AJC	1990	AMI (<76yrs old)	CCB	Placebo	878	897	1.33	137	170
DIABHYCAR	BMJ	2004	DM2 + raised excretion of urinary albumin (microalbuminuria/proteinuria) + >50yrs	ACEi	Placebo	2443	2469	3.92	61	78
DIRECT-PROTECT 2	Journal of Hypertension	2011	DM2 normo/hypertensive + normoalbuminuric + mild-moderate retinopathy	ARB	Placebo	951	954	4.7	28	34
DREAM	NEJM/Diabetes Care	2006/2008	non-diabetic + impaired fasting glucose levels or impaired glucose tolerance + no CVD	ACEi	Placebo	2623	2646	3	14	11
Dutch TIA	Stroke	1993	TIA/ischemic stroke (non-disabling)	BB	Placebo	732	741	2.67	45	40
E-COST	Hypertension Research	2005	HTN	ARB	Com	1053	995	3.1	10	23
ELITE II	Lancet	2000	HF (NYHA II-IV) + EF≤40% + ≥60yrs	ARB	ACEi	1578	1574	1.5	31	28
ELSA	Circulation	2002	HTN (SBP:150 to 210 mm Hg and DBP: 95-115 mm Hg)	BB	CCB	1157	1177	3.75	17	18
EUROPA	Lancet	2003	stable coronary artery dx + no HF	ACEi	Placebo	6110	6108	4.2	320	418
EWPHE	Lancet	1985	HTN elderly (>60yrs) (DBP 90-119 & SBP 160-239)	Diu	Placebo	416	424	4.69	17	29
FEVER	Journal of Hypertension	2005	HTN+ ≥1CVD event (140–180 mmHg (systolic) or 90–100 mmHg (diastolic))	CCB	Placebo	4841	4870	3.33	71	99
HAPPHY	Journal of Hypertension	1987	HTN men	Diu	BB	3272	3297	3.76	116	132
HEP	BMJ	1986	HTN elderly	Com	Placebo	419	465	4.4	35	38
HIJ-CREATE	European Heart Journal	2009	hosp w CAD (angiographically documented) + HTN	ARB	Com	1024	1025	4.2	29	26
HOMED-BP	Hypertension Research	2012	HTN + >40yrs	ACEi	ARB	1172	1175	5.31	22	19
HOMED-BP	Hypertension Research	2012	HTN + >40yrs	CCB	ACEi	1171	1172	5.31	12	22
HOMED-BP	Hypertension Research	2012	HTN + >40yrs	CCB	ARB	1171	1175	5.31	12	19
HOPE	NEJM	2000	CVD, CHD or DM2+RF, 1 cardiovascular risk factor (eg. Smoking, hypertension, lipid abnormality), no LVD, no HF.	ACEi	Placebo	4645	4652	4.5	459	570

Study Name	Journal	Year of Publication	Characteristics of Patients	Intervention_drug	Comparator_drug	Num in Treat	Num in Control	Duration	CHD in Treat	CHD in Control
HYVET	NEJM	2008	elderly HTN (SBP≥160)	Diu	Placebo	1933	1912	2.1	9	12
I-Preserve	NEJM	2008	HF (NYHA II-IV) + LVEF ≥45% + ≥60yrs	ARB	Placebo	2067	2061	4.13	60	54
IDNT	NEJM	2001	nephropathy due to DM2 + HTN + proteinuria	ARB	Placebo	579	569	2.6	44	46
IDNT	NEJM	2001	nephropathy due to DM2 + HTN + proteinuria	CCB	Placebo	567	569	2.6	27	46
IDNT	NEJM	2001	nephropathy due to DM2 + HTN + proteinuria	ARB	CCB	579	567	2.6	44	27
INSIGHT	Lancet	2000	HTN (BP ≥150/95 or ≥160 mm Hg) + ≥1 cardiovascular risk factor	CCB	Diu	3157	3164	4	94	84
INVEST	JAMA	2003	HTN + CAD	CCB	BB	11267	11309	2.7	452	441
IPPSH	Journal of Hypertension	1985	HTN (DBP 100-125)	BB	Com	3185	3172	4	98	107
JAMP	AHJ	2004	AMI	ACEi	Com	422	466	5.8	14	11
JMIC-B	Hypertension Research	2003	HTN + CAD	CCB	ACEi	828	822	2.98	16	13
Kanamasa	Cancer	1999	AMI	CCB	Placebo	566	488	2.19	30	26
LIFE	Lancet	2002	LVH + HTN (160-200/95-115) (LVH ascertained by ECG)	ARB	BB	4605	4588	4.8	198	188
LIT	European Heart Journal	1987	AMI	BB	Placebo	1195	1200	1.5	59	57
MACH-1	Circulation	1999	CHF (II-IV) LVF<35%	CCB	Placebo	1295	1295	1.59	9	10
MDPIT	NEJM	1988	AMI	CCB	Placebo	1232	1234	2.08	202	226
MERIT-HF	Lancet/2000	1999/2000	CHF (II-IV) + EF≤0.4	BB	Placebo	1990	2001	1	139	225
MIDAS	JAMA	1996	DBP: 95-115	CCB	Diu	442	441	3	6	5
MRC-1	BMJ	1985	HTN (90-109)	Diu	Placebo	4297	8654	5.5	119	234
MRC-1	BMJ	1985	HTN (90-109)	BB	Placebo	4403	8654	5.5	103	234
MRC-1	BMJ	1985	HTN (90-109)	Diu	BB	4297	4403	5.5	119	103
MRC-2	BMJ	1992	HTN elderly (SBP: 160-209, DBP<115)	Diu	Placebo	1081	2213	5.8	48	159
MRC-2	BMJ	1992	HTN elderly (SBP: 160-209, DBP<115)	BB	Placebo	1102	2213	5.8	80	159
MRC-2	BMJ	1992	HTN elderly (SBP: 160-209, DBP<115)	Diu	BB	1081	1102	5.8	48	80
MultiCentre Int	BMJ	1975	AMI (in hospital 7-28 days after AMI)	BB	Placebo	1524	1514	1.18	116	162
NAVIGATOR	NEJM	2010	IGT + CVD/≥1 CVD risk factors	ARB	Placebo	4631	4675	6.5	138	140
NICOLE	Heart/AJC	2003/2001	PTCA (Percutaneous Transluminal Coronary Angioplasty)	CCB	Placebo	408	411	3	16	13
NORDIL	Lancet	2000	HTN (DBP≥100)	CCB	Com	5410	5471	4.5	183	157
Norwegian Multicentre Study Group	NEJM	1981	AMI	BB	Placebo	945	939	1.42	136	189
ONTARGET	NEJM	2008	vasc dx/ DM & high risk	ARB	ACEi	8542	8576	4.67	440	413
OPTIMAAL	Lancet	2002	AMI+ HF	ARB	ACEi	2744	2733	2.7	384	379
OSCAR	AJM/Hypertension Research	2012/2009	elderly+ HTN+ high risk (DM2/CVD)	Com	ARB	586	578	3	7	6
Oslo	AJM	1980	HTN (SBP: 150-179 DBP<110)	Com	Placebo	406	379	5.5	8	8
PART-2	JACC	2000	CHD or CVD	ACEi	Placebo	308	309	4.7	22	33

Study Name	Journal	Year of Publication	Characteristics of Patients	Intervention_drug	Comparator_drug	Num in Treat	Num in Control	Duration	CHD in Treat	CHD in Control
PATE	American Journal Hypertension	2000	HTN + >60yrs	ACEi	CCB	699	1049	2.37	3	6
PATS	Hypertension Research	2009	History of TIA or Stroke	Diu	Placebo	2840	2825	2	26	23
PEACE	NEJM	2004	CAD	ACEi	Placebo	4158	4132	4.8	222	220
PHARAO	Hypertension	2008	high-normal BP	ACEi	Placebo	505	503	3	4	5
PRAISE 2	JACC	2013	Severe CHF (NYHA: III or IV) + EF<30%	CCB	Placebo	827	827	2.75	81	72
PREVEND IT	Circulation	2004	microalbuminuria	ACEi	Placebo	431	433	3.83	12	11
PREVENT	Circulation	2000	CAD	CCB	Placebo	417	408	3	19	20
PRoFESS	NEJM	2008	stroke (ischaemic)	ARB	Placebo	10146	10186	2.5	168	169
PROGRESS	Lancet		no DM + hx stroke	ACEi	Placebo	2657	2686	3.9	85	133
QUIET	AJC	2001	IHD & preserved LVF	ACEi	Placebo	878	872	2.25	48	54
RENAAL	NEJM	2001	DM2 + nephropathy	ARB	Placebo	751	762	3.4	50	68
ROADMAP	NEJM	2011	DM2 + normoalbuminuria + 1 additional CV risk factor	ARB	Placebo	2232	2215	3.2	5	0
SAVE	NEJM	1992	LVD after MI (EF≤40%)	ACEi	Placebo	1115	1116	3.5	133	170
SHELL	Blood Pressure	2003	elderly ISH (≥60yrs, SBP≥160 DBP≤95)	CCB	Diu	942	940	2.67	12	14
SHEP	JAMA	1991/1997	elderly ISH (SBP 160-219, DBP<90)	Com	Placebo	2365	2371	4.5	104	141
SOLVD- Prevention	NEJM	1992	EF≤35% + LVD	ACEi	Placebo	2111	2117	3.12	46	52
SOLVD-Treatment	NEJM	1991	CHF + EF≤35%	ACEi	Placebo	1285	1284	3.45	53	40
STONE	Hypertension	1996	elderly HTN (60-79yrs, SBP≥160 DBP≥96)	CCB	Placebo	891	741	2.5	2	2
STOP	Lancet	1991	elderly HTN (70-84yrs) SBP: 180-230, DBP≥90 (or DBP 105-120 irrespective of SBP)	ACEi	Placebo	812	815	2.08	25	28
STOP-2	Lancet	1999	elderly HTN (70-84yrs)	ACEi	CCB	2205	2196	4	139	179
SUPPORT	European Heart Journal	2015	CHF+ HTN	ARB	Placebo	578	569	4.4	13	12
Syst-China	Journal of Hypertension/Arch Intern Med	1998/2000	ISH elderly (≥60yrs SBP 160-219 DBP<95)	CCB	Placebo	1253	1141	3	9	7
Syst-Eur	Lancet	1997	ISH elderly (≥60yrs SBP: 160-219 DBP<95)	CCB	Placebo	2398	2297	2	33	45
Taylor	NEJM	1982	AMI	BB	Placebo	632	471	4	119	102
TOMHS	JAMA	1993	HTN	Com	Placebo	668	234	4.4	26	12
TRACE	NEJM/AJC	1995/1996	AMI + LVD (EF<35%)	ACEi	Placebo	876	873	2.17	99	113
TRANSCEND	Lancet	2008	ACEi intolerant + CVD/DM	ARB	Placebo	2954	2972	4.67	116	147
UKPDS	BMJ	1998	DM2 HTN	ACEi	BB	400	358	8.4	61	46
VA NEPHRON-D	NEJM	2013	DM2+ Nephropathy	ACEi	Placebo	724	724	2.2	52	40
VAL-HEFT	NEJM	2001	Heart failure (NYHA class II-IV) with EF < 40 %	ARB	Placebo	2511	2499	1.92	262	258
VALIANT	NEJM/JACC	2003/2006	AMI+HF/LVD	ARB	ACEi	4909	4909	2.06	1102	1132
VALUE	Lancet	2004	HTN + risk factor (SBP: 160-210, DBP<115)	ARB	CCB	7649	7596	4.2	369	313
VART	Hypertension Research	2011	HTN (SBP≥140 DBP≥90)	ARB	CCB	510	511	3.4	2	1
VHAS	Journal of Hypertension	1997	HTN (SBP≥160 DBP≥95)	Diu	CCB	707	707	2	5	5

HTN: Hypertension; DBP: Diastolic Blood Pressure; SBP: Systolic Blood Pressure; GFR: Glomerular Filtration Rate; DM2: Type 2 Diabetes Mellitus; CVD: Cardiovascular Disease; CHD: Coronary Heart Disease; MI: Myocardial Infarction; hx: History; CAD: Coronary Artery Disease; AF: Atrial Fibrillation; HF: Heart Failure; NYHA: New York Heart Association; LVEF: Left Ventricular Ejection Fraction; AMI: Acute Myocardial Infarction; LVD: Left Ventricular Dysfunction; CHF: Congestive Heart Failure; ACEi: Angiotensin-Converting Enzyme Inhibitor; ISH: Isolated Systolic Hypertension; RF: Risk Factor; LVH: Left Ventricular Hypertrophy; ECG: Electrocardiogram; IGT: Impaired Glucose Tolerance; PTCA: Percutaneous Transluminal Coronary Angioplasty; vasc dx: Vascular Disease; EF: Ejection Fraction; microalbuminuria: Small amounts of albumin in the urine; proteinuria: Presence of excess protein in the urine; IHD: Ischemic Heart

Table S4. The diagnostic criteria in each cohort included in the genome-wide association study of non-alcoholic fatty liver disease (NAFLD)

Use in this study	Cohort Name	Number of case	Number of control	Diagnostic criteria/code	Exclusion criteria
Primary analysis	Million Veteran Program	68,725	95,472	Elevated alanine aminotransferase (ALT) levels of higher than 40 U/L for men or 30 U/L for women on at least two occasions, separated by a minimum of six months, within a two-year timeframe prior to enrolment	Other causes of liver disease, chronic liver conditions, systemic diseases, or alcohol use disorders
Replication analysis	Electronic Medical Records and Genomics network (eMERGE)	1106	8571	ICD9: 571.5, 571.8, 571.9, ICD10: K75.81, K76.0, K76.9	Including but not limited to alcohol dependence, alcoholic liver disease, alpha-1 antitrypsin deficiency, Alagille syndrome, liver transplant, cystic fibrosis, hepatitis, abetalipoproteinemia, LCAT deficiency, lipodystrophy, disorders of copper metabolism Reye’s syndrome, inborn errors of metabolism, HELLP syndrome, starvation and acute fatty liver
	UK Biobank	2558	395,241	ICD10: K74.0, K74.2, K75.8, K76.0, K76.9	Same as above
	Estonian Biobank	4119	190,120	ICD10: K74.0, K74.2, K75.8, K76.0, K76.9	Same as above
	FinnGen	651	176,248	ICD10: K76.0	NA

We conducted the primary analysis using NAFLD GWAS data from the Million Veteran Program. We further conducted a replication analysis using NAFLD GWAS data from four additional cohorts.

Table S5. Biological mechanisms of the refined final-stage genes involved in blood pressure regulation

Gene	Related antihypertensive drug class	Function & biological role	Role in blood pressure regulation	Possible pharmacological mechanisms of action related to blood pressure regulation
ACE	RAS inhibitors	Membrane-bound enzyme that converts angiotensin I to angiotensin II (potent vasoconstrictor) and degrades bradykinin (vasodilator). Central to the renin-angiotensin system (RAS), regulating vascular tone and fluid balance.	ACE activity elevates blood pressure via angiotensin II (vasoconstriction, aldosterone-driven sodium retention) and reduced bradykinin (less vasodilation)	ACE inhibitors block Ang II synthesis and increase bradykinin, lowering BP. First-line therapy for hypertension and heart failure.
JUN	RAS inhibitors	Encodes c-Jun, a component of the AP-1 transcription factor complex. Regulates genes involved in cell proliferation, stress responses, and apoptosis. Expressed in vascular smooth muscle and brain cardiovascular centers.	AP-1 (c-Jun) mediates Ang II-induced vascular smooth muscle hypertrophy and arterial stiffening. Excessive AP-1 activity in the brain increases sympathetic nervous system activation, elevating BP. Drives vascular smooth muscle cell (VSMC) hypertrophy and endothelial inflammation, contributing to arterial stiffness.	ACE inhibitors/ARBs reduce Ang II-driven AP-1 activation. Blocking the renin-angiotensin system (with ACE inhibitors or ARBs) lowers Ang II levels and thus may indirectly dampen c-Jun/AP-1 activity and the pro-hypertensive gene programs it drives.
LTA4H	RAS inhibitors	Bifunctional enzyme: converts LTA4 to pro-inflammatory LTB4 and degrades neutrophil chemoattractants. Key player in inflammation and immune cell recruitment.	Chronic vascular inflammation driven by LTB4 contributes to hypertension. Elevated LTB4 in brain cardiovascular centers (e.g., NTS) promotes sympathetic overactivity and BP elevation.	LTA4H inhibitors (e.g., bestatin) reduce inflammation but are not yet clinically approved. Experimental LTB4 receptor antagonists reduce BP in animal models.
MMP9	RAS inhibitors	Protease degrading extracellular matrix (collagen, elastin) to facilitate tissue remodeling and immune cell infiltration. Activity regulated by TIMP-1.	Acute hypertension upregulates MMP-9 to preserve vascular compliance, but chronic elevation drives arterial stiffness and organ damage. High MMP-9 levels correlate with hypertensive vascular remodeling.	RAS blockers (e.g., ACE inhibitors, ARBs) suppress Ang II-induced MMP-9 activation. Broad-spectrum MMP inhibitors (e.g., doxycycline) reduce remodeling in animal models.
PPARG	RAS inhibitors	Nuclear receptor regulating adipogenesis, lipid metabolism, and insulin sensitivity. Modulates vascular inflammation, endothelial function, and sodium handling.	PPARγ activation reduces vasoconstriction (via angiotensin II receptor suppression) and enhances nitric oxide bioavailability. Loss-of-function mutations cause severe insulin resistance and early-onset hypertension.	Thiazolidinediones (e.g., pioglitazone) lower BP but cause fluid retention. Dual PPARG agonists (e.g., saroglitazar) in development.
ADRA2A	Beta blockers	Encodes the α2A-adrenergic receptor, a G protein-coupled receptor that inhibits neurotransmitter release (e.g., noradrenaline) in sympathetic nerves.	Activation of ADRA2A reduces sympathetic outflow, lowering heart rate and vascular resistance. Polymorphisms in ADRA2A are linked to altered autonomic control of blood pressure (BP) and hypertension risk.	Centrally acting α2-agonists (e.g., clonidine, moxonidine) target ADRA2A to reduce sympathetic activity. Used in resistant hypertension.
ADRB1	Beta blockers	Encodes the β1-adrenergic receptor, primarily in cardiac tissue. Mediates catecholamine-induced increases in heart rate and contractility.	Overactivation of ADRB1 increases cardiac output and renin release, elevating BP. Genetic variants (e.g., Arg389Gly) influence β-blocker efficacy and hypertension severity.	Beta-blockers (e.g., atenolol, bisoprolol) antagonise ADRB1, reducing heart rate and renin secretion. First-line therapy for hypertension with comorbid arrhythmias.
ADRB2	Beta blockers	Encodes the β2-adrenergic receptor, found in vascular and bronchial smooth muscle. Mediates vasodilation and bronchodilation.	ADRB2 activation causes vasodilation, lowering BP. Genetic variants (e.g., Arg16Gly) alter receptor desensitisation, affecting vascular tone and hypertension risk.	β2-agonists (e.g., salbutamol) are not antihypertensives but may indirectly influence BP. Non-selective β-blockers (e.g., propranolol) inhibit ADRB2, potentially worsening vasoconstriction in some cases.
HIF1A	Beta blockers	Encodes hypoxia-inducible factor 1-alpha, a transcription factor activated under low oxygen. Regulates genes involved in angiogenesis and erythropoiesis.	Chronic hypoxia (e.g., sleep apnoea) activates HIF1A, increasing erythropoietin and red blood cell production, which can raise BP. HIF1A also promotes vascular remodelling, contributing to pulmonary hypertension.	Roxadustat (HIF-prolyl hydroxylase inhibitor) increases erythropoietin but may exacerbate hypertension.
KCNH2	Beta blockers	Encodes the hERG potassium channel, critical for cardiac repolarisation. Mutations cause long QT syndrome.	Indirectly influences BP via cardiac rhythm stability. Arrhythmias (e.g., long QT syndrome) can reduce cardiac output, though KCNH2 is not a direct BP regulator.	Some research indicates that KCNH2 genetic variations might influence how effectively beta-blockers lower BP. Genetic testing for KCNH2 mutations might help identify individuals at higher risk for LQTS or those who may not respond well to certain beta-blockers.
KCNJ2	Beta blockers	Encodes the Kir2.1 inward-rectifier potassium channel, maintaining resting membrane potential in cardiac and vascular smooth muscle cells.	Kir2.1 dysfunction alters vascular tone and cardiac excitability. In hypertension, reduced Kir2.1 activity in resistance arteries may impair vasodilation, contributing to elevated BP.	Experimental Kir2.1 activators (e.g., ML133) show potential for vasodilation in preclinical studies.
NDUFC2	Beta blockers	Encodes a subunit of mitochondrial Complex I (NADH	Mitochondrial dysfunction (e.g., oxidative stress) in vascular	Antioxidants (e.g., coenzyme Q10) or mitochondrial

Gene	Related antihypertensive drug class	Function & biological role	Role in blood pressure regulation	Possible pharmacological mechanisms of action related to blood pressure regulation
		dehydrogenase). Involved in oxidative phosphorylation and cellular energy production.	endothelium contributes to hypertension. NDUFC2 variants may impair endothelial nitric oxide (NO) synthesis, promoting vasoconstriction.	protectants may improve endothelial function in hypertension.
VCAM1	Beta blockers	Encodes vascular cell adhesion molecule-1, mediating leukocyte adhesion to endothelial cells during inflammation.	Chronic vascular inflammation (via VCAM1) promotes endothelial dysfunction and arterial stiffness, contributing to hypertension. Elevated VCAM1 levels correlate with hypertension severity.	Statins and anti-inflammatory agents (e.g., canakinumab) reduce VCAM1 expression, improving endothelial health.
VEGFA	Beta blockers	Encodes vascular endothelial growth factor A, critical for angiogenesis and vascular permeability.	VEGFA promotes nitric oxide (NO) production and vascular repair. However, excess VEGFA (e.g., in tumours) increases vascular permeability, causing oedema and secondary hypertension.	Anti-VEGF therapies (e.g., bevacizumab) used in cancer cause hypertension as a side effect. Management requires concomitant antihypertensives (e.g., ACE inhibitors).
ABCB1	Calcium channel blockers	Encodes P-glycoprotein, a drug efflux transporter in the blood-brain barrier and kidneys. Regulates absorption and excretion of xenobiotics.	ABCB1 polymorphisms influence bioavailability of antihypertensive drugs (e.g., digoxin). Reduced renal excretion of sodium due to altered transporter activity may indirectly affect blood pressure (BP).	ABCB1 variants affect responses to calcium channel blockers (e.g., verapamil) and β-blockers (e.g., carvedilol). Pharmacogenomic considerations are emerging.
AHR	Calcium channel blockers	Aryl hydrocarbon receptor; regulates detoxification enzymes (e.g., CYP1A1) and immune responses. Activated by environmental toxins (e.g., dioxins).	Chronic AHR activation by pollutants induces oxidative stress and endothelial dysfunction, contributing to hypertension. AHR-knockout mice show reduced BP under toxin exposure.	Antioxidants (e.g., N-acetylcysteine) may mitigate AHR-driven oxidative damage in hypertension.
CACNA1C	Calcium channel blockers	Encodes the α1C subunit of L-type calcium channels in cardiac/smooth muscle. Mediates calcium influx for muscle contraction and pacemaker activity.	Overactivity of CACNA1C increases vascular tone and cardiac output, elevating BP. Gain-of-function mutations are linked to hypertension and arrhythmias.	Calcium channel blockers (e.g., amlodipine, nifedipine) inhibit CACNA1C, causing vasodilation and BP reduction. First-line therapy for hypertension.
CACNA1D	Calcium channel blockers	Encodes the α1D subunit of L-type calcium channels, predominantly in vascular smooth muscle and adrenal glands.	CACNA1D mutations increase aldosterone secretion (via adrenal gland activity) and vascular resistance, driving hypertension. Elevated expression is observed in resistant hypertension.	Calcium channel blockers (e.g., diltiazem) target CACNA1D in vascular smooth muscle. Aldosterone synthase inhibitors (e.g., LCZ696) may complement therapy.
CACNA1H	Calcium channel blockers	Encodes the α1H subunit of T-type calcium channels, critical in renal and cardiac pacemaker cells.	T-type channels regulate renal blood flow and glomerular filtration. Overactivity in renal vasculature may promote sodium retention and hypertension.	T-type calcium channel blockers (e.g., mibefradil) reduce BP but are limited by side effects. Experimental use in resistant hypertension.
CACNA1I	Calcium channel blockers	Encodes the α1I subunit of T-type calcium channels, expressed in neurons and vascular smooth muscle.	Neuronal T-type channels modulate sympathetic outflow. Overactivation increases sympathetic drive, elevating BP. CACNA1I polymorphisms are linked to autonomic dysregulation in hypertension.	No approved T-type-specific blockers. Gabapentinoids (e.g., gabapentin) indirectly modulate neuronal calcium channels.
CACNA2D2	Calcium channel blockers	Encodes the α2δ-2 auxiliary subunit of voltage-gated calcium channels. Modulates channel trafficking and stability.	CACNA2D2 dysfunction alters calcium channel density in vascular smooth muscle, affecting contractility and BP. Rare mutations are associated with hypertension in animal models.	Gabapentin (binds α2δ subunits) is used for neuropathic pain but not hypertension. Potential off-target effects on BP regulation.
CACNA2D3	Calcium channel blockers	Encodes the α2δ-3 auxiliary subunit, modulating calcium channel function in neurons and cardiomyocytes.	Altered CACNA2D3 expression may disrupt calcium signalling in baroreceptor neurons, impairing BP homeostasis. Linked to orthostatic hypotension in genetic studies.	Experimental modulation of α2δ subunits could influence autonomic BP control.
CACNB2	Calcium channel blockers	Encodes the β2 subunit of L-type calcium channels, regulating channel inactivation and expression.	CACNB2 mutations alter vascular smooth muscle calcium handling, increasing peripheral resistance. Polymorphisms are linked to hypertension and response to calcium channel blockers.	Calcium channel blockers (e.g., verapamil) efficacy may vary with CACNB2 genotype. Personalised dosing based on β subunit variants is under research.
CACNB3	Calcium channel blockers	Encodes the β3 subunit of L-type calcium channels, primarily in cardiac tissue. Modulates channel kinetics and adrenergic sensitivity.	CACNB3 variants affect cardiac contractility and arrhythmia risk. Indirectly influences BP via cardiac output regulation.	In mouse models, its knockout leads to increased BP on a high-salt diet
CACNB4	Calcium channel blockers	Encodes the β4 subunit of calcium channels, critical for neuronal and endocrine cell function.	Altered β4 subunit activity disrupts calcium-dependent hormone release (e.g., aldosterone, adrenaline), indirectly affecting BP.	Experimental β subunit inhibitors are in preclinical stages.

Gene	Related antihypertensive drug class	Function & biological role	Role in blood pressure regulation	Possible pharmacological mechanisms of action related to blood pressure regulation
CALM1	Calcium channel blockers	Encodes calmodulin, a calcium-sensing protein that regulates ion channels (e.g., L-type calcium channels) and enzymes (e.g., nitric oxide synthase).	Calmodulin dysfunction impairs vascular smooth muscle relaxation and nitric oxide production, promoting vasoconstriction and hypertension. Rare CALM1 mutations cause severe paediatric hypertension.	Calcium channel blockers and NO enhancers (e.g., nitrates) indirectly modulate calmodulin pathways.
KCNH2	Calcium channel blockers	Encodes the hERG potassium channel, critical for cardiac repolarisation. Mutations cause long QT syndrome.	Indirectly influences BP via cardiac rhythm stability. Arrhythmias (e.g., long QT syndrome) can reduce cardiac output, though KCNH2 is not a direct BP regulator.	Antihypertensives (e.g., sotalol) with KCNH2-blocking activity risk arrhythmias. Avoided in hypertension with QT prolongation.
KCNJ11	Calcium channel blockers	Encodes the Kir6.2 subunit of ATP-sensitive potassium (KATP) channels. Regulates insulin secretion in pancreatic β-cells and vascular tone.	KCNJ11 mutations impair insulin secretion (leading to diabetes) and vascular KATP function, altering peripheral resistance. Hyperinsulinaemia from β-cell dysfunction may drive hypertension.	Sulphonylureas (e.g., glimepiride) close KATP channels to stimulate insulin secretion but may worsen vascular tone.
NR3C2	Calcium channel blockers	Encodes the mineralocorticoid receptor (MR), which binds aldosterone to regulate sodium reabsorption in the kidneys.	MR overactivation (e.g., aldosterone excess) increases sodium retention and blood volume, elevating BP. Genetic variants in NR3C2 are linked to salt-sensitive hypertension.	Mineralocorticoid receptor antagonists (e.g., spironolactone, eplerenone) block NR3C2, reducing sodium retention. Used in resistant hypertension and heart failure.
PDE1B	Calcium channel blockers	Encodes phosphodiesterase 1B, which hydrolyses cAMP/cGMP in vascular smooth muscle. Regulates cyclic nucleotide signalling and vasodilation.	PDE1B overexpression reduces cAMP/cGMP levels, promoting vasoconstriction. Inhibition enhances nitric oxide-mediated relaxation, lowering BP.	Experimental PDE1 inhibitors (e.g., vinpocetine) show vasodilatory effects in preclinical studies.
SMPD1	Calcium channel blockers	Encodes acid sphingomyelinase, which produces ceramide. Ceramide regulates endothelial apoptosis, inflammation, and vascular stiffness.	SMPD1 overactivity increases ceramide, promoting endothelial dysfunction and arterial stiffening. Elevated ceramide levels correlate with hypertension severity.	Acid sphingomyelinase inhibitors (e.g., amitriptyline) reduce ceramide and improve vascular function in animal models.
TNNC1	Calcium channel blockers	Encodes cardiac troponin C, a calcium-binding protein regulating cardiac muscle contraction.	TNNC1 mutations alter calcium sensitivity in cardiomyocytes, affecting cardiac output. Indirectly influences BP in heart failure-associated hypertension.	Inotropes (e.g., dobutamine) modulate cardiac troponin function but are not used for hypertension.
TNNC2	Calcium channel blockers	Encodes fast skeletal troponin C (fsTnC), regulating skeletal muscle contraction.	Skeletal muscle perfusion may indirectly affect systemic vascular resistance.	While fsTnC itself is not typically linked to BP control, its interaction with muscle physiology, calcium signaling, and vascular function may have indirect effects.
ABP1	Diuretics	Encodes actin-binding protein 1, involved in cytoskeletal organisation and cell motility.	Hypothetically, cytoskeletal changes in vascular smooth muscle could influence contractility, but evidence is lacking.	ABP1 influences BP regulation by remodeling the actin cytoskeleton, which affects vascular smooth muscle contractility, endothelial function, nitric oxide-mediated vasodilation, vascular remodeling, RhoA/ROCK signaling, and inflammatory responses, collectively contributing to arterial stiffness, vascular resistance, and hypertension.
ASIC1	Diuretics	Acid-sensing ion channel 1 (ASIC1) detects extracellular acidosis and regulates neuronal and vascular responses to pH changes.	ASIC1 activation in vascular smooth muscle promotes vasoconstriction under acidic conditions (e.g., ischaemia). Chronic acidosis in hypertension may exacerbate ASIC1-mediated vasoreactivity.	Experimental ASIC1 inhibitors (e.g., amiloride derivatives) show vasodilatory effects in preclinical models. Not yet clinically used.
ATP1A1	Diuretics	Encodes the α1 subunit of Na^+/K^+-ATPase, critical for maintaining ion gradients and cellular osmotic balance. Highly expressed in renal tubules.	ATP1A1 dysfunction impairs renal sodium reabsorption, altering fluid balance and BP. Rare mutations cause familial hyperkalaemia and hypertension.	Digoxin (Na^+/K^+-ATPase inhibitor) is used for heart failure but exacerbates hypertension.
CA2	Diuretics	Carbonic anhydrase II (CA2) catalyses CO_2 hydration, regulating pH and bicarbonate reabsorption in kidneys and erythrocytes.	CA2 activity influences renal acid-base balance and sodium retention. CA2 inhibitors (e.g., acetazolamide) induce diuresis, lowering BP in oedematous states.	Carbonic anhydrase inhibitors (e.g., acetazolamide) are diuretics used for glaucoma and altitude sickness.
CA4	Diuretics	Carbonic anhydrase IV (CA4), membrane-bound isoform in renal tubules and vascular endothelium. Modulates pH and ion transport.	CA4 regulates renal bicarbonate reabsorption and vascular tone. CA4 inhibition may reduce sodium retention and improve endothelial function.	Experimental CA4 inhibitors are under study for renal hypertension.
CACNA1C	Diuretics	Encodes the α1C subunit of L-type calcium channels in cardiac/smooth muscle. Mediates calcium influx for contraction and pacemaker activity.	Overactivity increases vascular tone and cardiac output, elevating BP. Gain-of-function mutations linked to hypertension.	Shared target with calcium channel blockers. Calcium channel blockers (e.g., amlodipine) inhibit CACNA1C, causing vasodilation. First-line therapy for hypertension.

Gene	Related antihypertensive drug class	Function & biological role	Role in blood pressure regulation	Possible pharmacological mechanisms of action related to blood pressure regulation
CACNA1D	Diuretics	Encodes the α1D subunit of L-type calcium channels, predominantly in vascular smooth muscle and adrenal glands.	Mutations increase aldosterone secretion and vascular resistance, driving hypertension. Elevated expression in resistant hypertension.	Shared target with calcium channel blockers. Calcium channel blockers (e.g., diltiazem) target CACNA1D. Aldosterone synthase inhibitors (e.g., LCZ696) complement therapy.
CACNB2	Diuretics	Encodes the β2 subunit of L-type calcium channels, regulating channel inactivation and expression.	CACNB2 variants alter vascular smooth muscle calcium handling, increasing peripheral resistance. Polymorphisms influence response to calcium channel blockers.	Shared target with calcium channel blockers. Calcium channel blockers (e.g., verapamil) efficacy varies with CACNB2 genotype. Personalised dosing under research.
CACNB3	Diuretics	Encodes the β3 subunit of L-type calcium channels, modulating channel kinetics in cardiac tissue.	Indirectly influences BP via cardiac output regulation. No direct association with hypertension reported.	β3 subunit modulation could theoretically fine-tune calcium channel blocker specificity.
CACNB4	Diuretics	Encodes the β4 subunit of calcium channels, critical for neuronal and endocrine cell function.	Altered β4 subunit activity disrupts calcium-dependent hormone release (e.g., aldosterone), indirectly affecting BP.	Experimental β subunit inhibitors in preclinical stages.
ESR1	Diuretics	Oestrogen receptor alpha (ERα), mediates oestrogen effects on vascular tone, inflammation, and lipid metabolism.	Oestrogen via ESR1 promotes vasodilation (via NO synthesis) and reduces vascular inflammation. Postmenopausal loss of ESR1 activity contributes to hypertension.	Hormone replacement therapy (HRT) may lower BP in postmenopausal women but is not a primary antihypertensive.
ESR2	Diuretics	Oestrogen receptor beta (ERβ), modulates vascular and renal responses to oestrogen. Shares some functions with ESR1 but with distinct tissue distribution.	ERβ activation in kidneys reduces sodium reabsorption, lowering BP. Polymorphisms linked to salt-sensitive hypertension.	Selective ERβ agonists (e.g., ERB-041) show preclinical BP-lowering effects but are not yet approved.
GPR35	Diuretics	G protein-coupled receptor 35, activated by kynurenic acid. Involved in immune regulation and vascular tone.	GPR35 activation in vascular endothelium promotes vasodilation. Polymorphisms may influence BP variability.	Experimental agonists (e.g., zaprinast) show vasodilatory potential.
KCNMA1	Diuretics	Encodes the BK potassium channel (Ca^{2+}-activated), critical for vascular smooth muscle relaxation.	BK channel activation hyperpolarises smooth muscle cells, inducing vasodilation. Loss-of-function mutations increase vascular resistance and BP.	BK channel openers (e.g., NS1619) reduce BP in animal models.
NR3C2	Diuretics	Mineralocorticoid receptor (MR), binds aldosterone to regulate renal sodium reabsorption.	MR overactivation increases sodium retention and blood volume, elevating BP. Genetic variants linked to salt-sensitive hypertension.	Mineralocorticoid receptor antagonists (e.g., spironolactone) block MR, reducing sodium retention. Used in resistant hypertension.
PLAU	Diuretics	Encodes urokinase (uPA), a serine protease involved in fibrinolysis and tissue remodelling.	uPA contributes to vascular remodelling and inflammation. Elevated PLAU expression in hypertension correlates with arterial stiffness.	ACE inhibitors may reduce uPA-mediated remodelling.
SCNN1A	Diuretics	Encodes the α subunit of the epithelial sodium channel (ENaC), critical for renal sodium reabsorption.	Gain-of-function mutations (e.g., Liddle syndrome) cause excessive sodium retention and severe hypertension.	ENaC inhibitors (e.g., amiloride) block sodium reabsorption, lowering BP. Used in Liddle syndrome and resistant hypertension.
SCNN1D	Diuretics	Encodes the δ subunit of ENaC, with limited expression in humans. Role in sodium transport is poorly characterised.	Hypothetically, SCNN1D variants could modulate ENaC activity in rare cases.	SCNN1D, as part of the ENaC sodium transport system, may influence BP regulation by affecting renal sodium reabsorption, RAAS interactions, vascular tone, and genetic susceptibility to hypertension, though its precise role remains less understood compared to other ENaC subunits.
SLC12A1	Diuretics	Encodes the $Na^+/K^+/2Cl^-$ cotransporter (NKCC2) in the kidney thick ascending limb. Critical for sodium reabsorption.	NKCC2 inhibition reduces sodium retention, lowering BP. Mutations cause Bartter syndrome (hypotension).	Loop diuretics (e.g., furosemide) target NKCC2, used for oedema and hypertension with renal impairment.
SLC12A2	Diuretics	Encodes the $Na^+/K^+/2Cl^-$ cotransporter (NKCC1), expressed in vascular smooth muscle and secretory epithelia.	NKCC1 regulates vascular tone via intracellular Cl^- accumulation. Overactivity increases vasoconstriction.	Loop diuretics (e.g., bumetanide) inhibit NKCC1 but are less effective for hypertension. Experimental NKCC1 blockers in development.
SLC12A3	Diuretics	Encodes the Na^+/Cl^- cotransporter (NCC) in the kidney distal convoluted tubule. Key player in sodium reabsorption.	NCC inhibition reduces sodium retention, lowering BP. Mutations cause Gitelman syndrome (hypotension).	Thiazide diuretics (e.g., hydrochlorothiazide) target NCC. First-line therapy for hypertension.
SLC12A4	Diuretics	Encodes the K^+/Cl^- cotransporter (KCC1), involved in cell volume regulation. Limited role in BP regulation.	KCC1 dysfunction may alter vascular smooth muscle cell volume.	SLC12A4 (KCC1) may influence blood pressure regulation by modulating vascular tone, electrolyte homeostasis, renal sodium handling, and neuronal control of BP, though its role in hypertension remains less defined compared to other ion transporters like NKCCs or ENaCs.
SLC9A1	Diuretics	Encodes the Na^+/H^+ exchanger 1 (NHE1), regulating	NHE1 overactivity in vascular smooth muscle increases	Experimental NHE1 inhibitors (e.g., cariporide) reduce BP in

Gene	Related antihypertensive drug class	Function & biological role	Role in blood pressure regulation	Possible pharmacological mechanisms of action related to blood pressure regulation
		intracellular pH and sodium homeostasis in multiple tissues.	intracellular Na^+, promoting vasoconstriction. Linked to salt-sensitive hypertension.	animal models.

Table S6. Characteristics of the genetic variants included in the main Mendelian randomisation analysis

SNP	effect_allele.exposure	other_allele.exposure	effect_allele.outcome	other_allele.outcome	eaf.exposure	eaf.outcome	beta.exposure	se.exposure	pval.exposure	beta.outcome	se.outcome	pval.outcome
rs10015278	C	T	C	T	0.3634	0.3663	0.02948	0.005	3.60E-09	-0.0116	0.0075	0.1235
rs10028284	T	A	T	A	0.1803	0.1783	0.05106	0.00638	1.09E-15	-0.0227	0.0097	0.01941
rs10052777	C	T	C	T	0.3961	0.4036	-0.05682	0.00496	2.73E-30	0.013	0.0076	0.08773
rs10066799	T	G	T	G	0.2808	0.284	-0.03682	0.00538	7.96E-12	0.0251	0.0083	0.002408
rs10069690	T	C	T	C	0.2585	0.2582	-0.05524	0.00572	4.43E-22	0.0135	0.0083	0.1038
rs10072115	C	A	C	A	0.648	0.6474	-0.02756	0.00502	4.01E-08	0.0046	0.0076	0.5492
rs1010064	C	A	C	A	0.1848	0.1886	0.06464	0.0062	1.97E-25	-0.0027	0.0095	0.7767
rs1010069	A	G	A	G	0.4553	0.455	-0.0325	0.00486	2.36E-11	0.011	0.0074	0.1374
rs10134573	T	C	T	C	0.0766	0.0802	0.05144	0.0092	2.19E-08	-0.0114	0.0142	0.4211
rs10149239	A	G	A	G	0.7386	0.7432	-0.03306	0.0055	1.88E-09	-0.0038	0.0083	0.6446
rs10159340	T	C	T	C	0.7313	0.7339	0.03062	0.00546	1.96E-08	0.0107	0.0084	0.2041
rs10171459	C	A	C	A	0.4746	0.4721	-0.02674	0.0048	2.57E-08	0.0046	0.0073	0.5267
rs10224210	C	T	C	T	0.2805	0.2871	-0.0706	0.00538	3.16E-39	-0.0095	0.0081	0.2395
rs1044822	T	C	T	C	0.1472	0.1458	0.04582	0.00686	2.29E-11	-0.0095	0.0106	0.3735
rs10459965	T	A	T	A	0.268	0.2625	-0.03878	0.00638	1.25E-09	0.0138	0.0083	0.09675
rs10468291	A	C	A	C	0.5695	0.5687	0.0328	0.0049	2.31E-11	-0.0073	0.0076	0.3379
rs10501082	T	C	T	C	0.405	0.4061	0.02846	0.0049	6.64E-09	-0.0032	0.0075	0.6659
rs10501410	A	G	A	G	0.0703	0.0724	-0.05426	0.00972	2.44E-08	0.0144	0.0151	0.3378
rs10512183	G	T	G	T	0.2277	0.2177	0.03236	0.00578	2.15E-08	8.00E-04	0.0092	0.9309
rs1057040	A	G	A	G	0.528	0.5296	0.04096	0.00478	1.23E-17	-0.0098	0.0073	0.1768
rs10759426	C	T	C	T	0.1275	0.1278	-0.06892	0.00724	1.69E-21	1.00E-04	0.0111	0.991
rs10766318	T	C	T	C	0.7951	0.7969	0.0845	0.00596	1.32E-45	0.0074	0.0091	0.4179
rs10776752	T	G	T	G	0.078	0.0715	-0.14502	0.0092	7.28E-56	0.0045	0.0142	0.7481
rs10777213	A	G	A	G	0.5252	0.5262	0.0339	0.0048	1.59E-12	0.0057	0.0073	0.4373
rs10782230	A	G	A	G	0.4826	0.4789	-0.0384	0.00486	2.56E-15	0.0211	0.0075	0.005055
rs10845556	G	A	G	A	0.4207	0.417	0.0307	0.00486	2.73E-10	-0.0121	0.0075	0.1056
rs10863593	C	T	C	T	0.3231	0.3181	0.03782	0.00514	2.09E-13	0.0046	0.0079	0.5587
rs10876531	C	A	C	A	0.294	0.2867	0.07306	0.00536	3.59E-42	-0.0013	0.0083	0.8749
rs10941043	G	T	G	T	0.2879	0.2839	-0.0384	0.0053	4.37E-13	-0.0072	0.0081	0.3761
rs10952729	G	A	G	A	0.428	0.4369	0.03002	0.00486	6.15E-10	-0.0038	0.0073	0.6025
rs11006779	C	T	C	T	0.5279	0.5283	-0.03642	0.00482	4.14E-14	0.0036	0.0073	0.6264
rs11026590	C	A	C	A	0.0746	0.0752	-0.06772	0.00946	8.18E-13	0.0161	0.0152	0.2884
rs11031051	C	A	C	A	0.3126	0.3189	-0.03666	0.00522	2.01E-12	0.0148	0.0079	0.06149
rs11037809	G	A	G	A	0.3035	0.3012	0.03064	0.00526	5.51E-09	6.00E-04	0.008	0.9417
rs11105375	A	T	A	T	0.1679	0.1678	0.1481	0.0065	2.94E-115	-6.00E-04	0.0101	0.952

SNP	effect_allele.exposure	other_allele.exposure	effect_allele.outcome	other_allele.outcome	eaf.exposure	eaf.outcome	beta.exposure	se.exposure	pval.exposure	beta.outcome	se.outcome	pval.outcome
rs11117882	G	A	G	A	0.2356	0.2359	-0.04666	0.0057	2.44E-16	0.0056	0.0086	0.5198
rs11123059	A	G	A	G	0.5683	0.5689	-0.02684	0.00486	3.47E-08	-0.0013	0.0075	0.861
rs11125853	G	A	G	A	0.2323	0.2322	0.05466	0.00574	1.50E-21	-4.00E-04	0.0088	0.9599
rs11190709	A	G	A	G	0.8869	0.8843	-0.0986	0.00766	5.59E-38	-0.0315	0.0114	0.005852
rs11191531	C	G	C	G	0.0818	0.0817	0.20116	0.00892	1.74E-112	-0.0128	0.014	0.3614
rs11241305	A	C	A	C	0.4894	0.4928	-0.0503	0.00482	1.58E-25	9.00E-04	0.0073	0.9007
rs11252324	T	G	T	G	0.0781	0.0801	0.06484	0.00908	9.13E-13	0.0074	0.0137	0.5899
rs112684153C		T	C	T	0.0735	0.073	0.06792	0.00996	9.24E-12	0.005	0.0159	0.7529
rs112835657A		G	A	G	0.0332	0.0333	0.07782	0.01376	1.54E-08	-0.0177	0.0212	0.4045
rs1133400	G	A	G	A	0.2132	0.2109	-0.05244	0.00596	1.24E-18	0.0369	0.0089	3.69E-05
rs113393496A		G	A	G	0.1955	0.1974	0.0344	0.00616	2.47E-08	-0.0139	0.0092	0.13
rs113814035T		C	T	C	0.0983	0.0972	-0.05412	0.00816	3.25E-11	-0.0128	0.0123	0.2994
rs114165349C		G	C	G	0.0257	0.0209	-0.11518	0.01654	3.39E-12	0.1993	0.0279	9.10E-13
rs11571376	G	C	G	C	0.2951	0.3006	-0.0397	0.00534	1.10E-13	-0.003	0.0083	0.7157
rs11592107	A	G	A	G	0.3079	0.3067	-0.04768	0.00522	6.13E-20	0.0157	0.008	0.04871
rs11622562	T	C	T	C	0.3304	0.329	0.03144	0.00512	7.71E-10	-0.0075	0.0078	0.3351
rs11636952	C	T	C	T	0.6807	0.6776	0.08658	0.0052	3.11E-62	-0.0065	0.0079	0.4147
rs11646118	C	T	C	T	0.083	0.0807	0.05342	0.00892	2.17E-09	0.0064	0.0141	0.651
rs11676702	C	T	C	T	0.0813	0.0825	-0.06102	0.009	1.14E-11	0.023	0.0133	0.08418
rs116793716G		T	G	T	0.0169	0.0168	0.11742	0.01944	1.55E-09	0.0147	0.0282	0.6006
rs116839142G		T	G	T	0.029	0.0271	-0.08522	0.01556	4.40E-08	0.0831	0.0247	0.0007841
rs117037316G		C	G	C	0.0686	0.0628	-0.06828	0.01022	2.28E-11	-0.0218	0.0163	0.1808
rs117285318C		T	C	T	0.0763	0.075	0.08318	0.00944	1.25E-18	-0.0141	0.0141	0.3148
rs117472357T		C	T	C	0.0413	0.0413	-0.09222	0.01268	3.60E-13	0.0098	0.0202	0.6264
rs117539635G		A	G	A	0.0237	0.0134	0.19318	0.0223	4.47E-18	-0.034	0.05	0.4966
rs11852739	A	G	A	G	0.7387	0.7414	-0.03422	0.00554	6.99E-10	2.00E-04	0.0085	0.9828
rs11853701	C	G	C	G	0.3338	0.3334	-0.02854	0.0052	4.17E-08	0.0121	0.008	0.1309
rs11977526	A	G	A	G	0.4047	0.411	0.06588	0.00492	9.67E-41	-0.0141	0.0074	0.05654
rs11988716	G	A	G	A	0.1344	0.1383	-0.0422	0.00716	3.62E-09	0.017	0.0105	0.1062
rs12035750	C	T	C	T	0.3713	0.3669	-0.04966	0.00504	6.88E-23	-0.0309	0.0079	8.47E-05
rs12088448	C	A	C	A	0.3527	0.3457	-0.03534	0.00512	5.65E-12	-0.0105	0.0079	0.1878
rs12102426	G	T	G	T	0.428	0.4312	0.05528	0.00486	4.72E-30	-0.0048	0.0075	0.5202
rs12134698	A	C	A	C	0.1948	0.1946	0.04182	0.00604	4.56E-12	0.0194	0.0091	0.03397
rs12138150	T	C	T	C	0.3938	0.3794	0.04152	0.00494	4.50E-17	-0.009	0.0077	0.2425
rs1214761	G	A	G	A	0.6655	0.6461	0.06556	0.00514	2.18E-37	-0.0055	0.0079	0.4825
rs12149258	G	A	G	A	0.1591	0.1627	0.04702	0.00662	1.23E-12	0.0024	0.01	0.8085

SNP	effect_all ele. exposure	other_all ele. exposure	effect_all ele. outcome	other_all ele. outcome	eaf.exposure	eaf.outcome	beta.exposure	se.exposure	pval.exposure	beta.outcome	se.outcome	pval.outcome
rs12150907	A	G	A	G	0.1937	0.1909	-0.04062	0.0062	6.05E-11	0.0167	0.0097	0.08342
rs12153395	A	G	A	G	0.1097	0.0986	0.0576	0.00796	4.79E-13	-0.0111	0.0128	0.3837
rs12192157	T	C	T	C	0.5487	0.5504	-0.03544	0.00496	9.23E-13	0.0056	0.0073	0.4442
rs12195276	T	C	T	C	0.723	0.7238	0.03202	0.00546	4.45E-09	2.00E-04	0.0084	0.9841
rs12199746	A	G	A	G	0.595	0.5908	0.03056	0.00494	6.16E-10	-0.0145	0.0074	0.05166
rs12378485	T	G	T	G	0.6519	0.6526	0.037	0.00524	1.79E-12	-0.0023	0.0079	0.7723
rs12419342	T	C	T	C	0.7029	0.7086	-0.03818	0.00532	7.23E-13	0.0184	0.0082	0.02466
rs12494446	A	G	A	G	0.6288	0.6235	-0.04332	0.00498	3.15E-18	0.0035	0.0075	0.6437
rs12509595	C	T	C	T	0.2915	0.2905	-0.14082	0.00532	5.97E-155	-0.0108	0.008	0.1789
rs12511987	G	T	G	T	0.178	0.1797	-0.0414	0.00642	1.08E-10	0.0041	0.0099	0.6809
rs12638862	G	A	G	A	0.263	0.2626	0.05614	0.0055	1.73E-24	0.0035	0.0083	0.6725
rs12639622	C	T	C	T	0.1853	0.1746	0.03836	0.00632	1.34E-09	0.0043	0.0102	0.6761
rs12752228	T	C	T	C	0.1804	0.1786	-0.03598	0.00628	9.68E-09	-0.0064	0.0096	0.5069
rs1275980	T	C	T	C	0.6114	0.6122	0.10328	0.00492	3.31E-98	0.0082	0.0074	0.2707
rs1290933	A	C	A	C	0.6902	0.6894	0.0474	0.00522	1.20E-19	-0.0117	0.008	0.1427
rs1292053	G	A	G	A	0.4399	0.4412	-0.03374	0.00484	3.01E-12	0.0019	0.0073	0.7988
rs12926550	A	G	A	G	0.3171	0.3216	0.03996	0.0052	1.66E-14	-0.0096	0.008	0.2345
rs13107325	T	C	T	C	0.0764	0.0833	0.14912	0.00922	6.00E-59	0.0526	0.0132	6.36E-05
rs13118687	A	G	A	G	0.4636	0.4528	0.05268	0.00488	4.13E-27	-0.0022	0.0074	0.7666
rs13156073	G	A	G	A	0.0751	0.0726	-0.07194	0.00916	4.33E-15	-0.0087	0.0141	0.5402
rs13203975	A	G	A	G	0.1164	0.1171	-0.05742	0.00762	4.83E-14	0.0362	0.0117	0.001983
rs13234407	A	G	A	G	0.4867	0.4827	0.04564	0.00478	1.39E-21	-0.0128	0.0072	0.07809
rs1327235	G	A	G	A	0.4689	0.4657	-0.07464	0.00478	5.73E-55	0.0036	0.0073	0.6243
rs1408945	T	G	T	G	0.4228	0.4238	0.05636	0.00486	4.16E-31	-0.0071	0.0074	0.3359
rs141958336A		G	A	G	0.0419	0.04	-0.14784	0.01266	1.77E-31	-0.0086	0.02	0.6677
rs143167197G		A	G	A	0.072	0.0736	-0.07862	0.00984	1.43E-15	0.0753	0.0148	3.90E-07
rs1436138	G	A	G	A	0.3669	0.3765	0.04956	0.00502	6.39E-23	-0.0238	0.0077	0.001902
rs1474698	T	C	T	C	0.5503	0.5454	0.03868	0.00486	1.59E-15	0.0063	0.0074	0.3946
rs150857355C		G	C	G	0.0217	0.0209	-0.16602	0.0179	1.71E-20	-0.0084	0.0275	0.7604
rs1527575	G	A	G	A	0.6414	0.6403	0.0307	0.00506	1.38E-09	-0.0045	0.0079	0.5639
rs1621	A	G	A	G	0.6685	0.6653	0.03392	0.00514	4.36E-11	-7.00E-04	0.0079	0.9283
rs1623112	C	T	C	T	0.6372	0.6305	0.0527	0.005	5.23E-26	-0.0148	0.0076	0.05173
rs1630736	T	C	T	C	0.4622	0.4579	0.03328	0.00492	1.39E-11	-0.0103	0.0075	0.1682
rs167479	T	G	T	G	0.4766	0.4826	0.0988	0.00516	5.50E-82	-0.0109	0.0073	0.1365
rs16849728	G	A	G	A	0.0604	0.06	0.06948	0.01032	1.71E-11	-0.0488	0.0165	0.00316
rs16931177	G	C	G	C	0.6049	0.6171	0.03818	0.00494	1.22E-14	-0.006	0.0075	0.4238
rs16939357	C	T	C	T	0.158	0.1664	0.05434	0.00668	4.44E-16	-0.0105	0.0102	0.2994

SNP	effect_all ele. exposure	other_all ele. exposure	effect_all ele. outcome	other_all ele. outcome	eaf.exposure	eaf.outcome	beta.exposure	se.exposure	pval.exposure	beta.outcome	se.outcome	pval.outcome
rs1694068	A	T	A	T	0.6161	0.6196	-0.04926	0.00496	3.28E-23	0.0236	0.0076	0.00177
rs17030997	T	A	T	A	0.0913	0.0944	0.0467	0.00846	3.34E-08	9.00E-04	0.0127	0.9461
rs17035181	G	T	G	T	0.1456	0.1463	0.05898	0.00684	7.22E-18	-0.0223	0.0105	0.03293
rs17171688	A	G	A	G	0.0461	0.047	0.08548	0.01238	5.23E-12	-0.018	0.0204	0.3775
rs17189980	C	T	C	T	0.0371	0.0367	-0.08272	0.01326	4.37E-10	-0.0072	0.0193	0.7081
rs17400834	C	T	C	T	0.1428	0.1438	-0.0381	0.00694	3.83E-08	-0.0019	0.0107	0.8597
rs17474056	T	C	T	C	0.5627	0.5688	0.04304	0.00486	9.23E-19	-0.0071	0.0075	0.3471
rs17562391	T	C	T	C	0.418	0.4191	-0.04134	0.00488	2.31E-17	0.0092	0.0074	0.2133
rs17574500	T	C	T	C	0.1366	0.1344	-0.03996	0.00722	3.22E-08	0.0146	0.0114	0.2019
rs17608766	C	T	C	T	0.1403	0.1323	-0.11446	0.00696	7.46E-61	0.0184	0.0107	0.08522
rs17609994	G	A	G	A	0.2002	0.1941	-0.06138	0.00622	5.78E-23	-0.0152	0.0098	0.1214
rs17653824	A	T	A	T	0.1207	0.1176	-0.04438	0.00744	2.56E-09	0.0108	0.0117	0.3552
rs17677603	G	A	G	A	0.383	0.3802	-0.06256	0.00496	2.36E-36	-0.0075	0.0076	0.3235
rs17679259	G	A	G	A	0.1331	0.1378	0.07166	0.00712	7.17E-24	-0.025	0.0108	0.02093
rs17737180	T	C	T	C	0.5571	0.5563	-0.02848	0.00488	5.08E-09	-0.0052	0.0074	0.4805
rs1779240	A	G	A	G	0.7662	0.7706	0.07722	0.0057	7.24E-42	0.0071	0.0088	0.4176
rs17797222	G	A	G	A	0.2225	0.2204	0.0364	0.00594	9.13E-10	-0.005	0.0091	0.5862
rs17812022	T	C	T	C	0.0954	0.094	0.06306	0.0086	2.26E-13	-0.032	0.0142	0.02438
rs1786345	C	A	C	A	0.4352	0.4361	0.03842	0.00488	3.77E-15	-0.0026	0.0074	0.7262
rs1852922	A	G	A	G	0.6845	0.6796	-0.03822	0.00524	3.31E-13	0.0045	0.0081	0.5764
rs1863798	G	A	G	A	0.3791	0.3808	-0.04146	0.00502	1.36E-16	0.002	0.0076	0.7921
rs1886220	G	A	G	A	0.7556	0.7648	-0.0408	0.00564	4.88E-13	0.0063	0.0087	0.4718
rs1894774	G	A	G	A	0.5092	0.5043	0.04438	0.00484	5.08E-20	-0.0043	0.0075	0.5642
rs1898165	A	G	A	G	0.5247	0.524	0.03878	0.00482	8.22E-16	0.0019	0.0074	0.8017
rs190533862A		T	A	T	0.0641	0.0642	-0.0575	0.01034	2.75E-08	-0.0027	0.0153	0.8608
rs1906672	A	G	A	G	0.2323	0.2319	-0.0503	0.00572	1.44E-18	-0.0051	0.0087	0.5571
rs1962412	C	T	C	T	0.6975	0.6936	-0.04006	0.0053	4.40E-14	0.0112	0.0082	0.1742
rs1962648	T	C	T	C	0.4198	0.4209	-0.03718	0.00488	2.51E-14	-0.0029	0.0075	0.7012
rs1976450	T	G	T	G	0.1628	0.1679	0.03882	0.00652	2.68E-09	-0.0031	0.0098	0.7528
rs1982784	G	A	G	A	0.8105	0.8111	0.03446	0.00626	3.68E-08	-0.0082	0.0097	0.3973
rs2003931	A	G	A	G	0.3493	0.3511	0.04248	0.00508	5.68E-17	-0.0103	0.0078	0.1844
rs2011603	A	G	A	G	0.7365	0.7436	-0.04928	0.0055	3.14E-19	0.0258	0.0083	0.001877
rs2023844	A	G	A	G	0.9245	0.9249	-0.16164	0.00914	7.34E-70	-8.00E-04	0.0139	0.9538
rs2042364	G	A	G	A	0.5358	0.5341	-0.03048	0.00482	2.80E-10	0.0076	0.0074	0.3053
rs2046341	A	G	A	G	0.1915	0.1892	0.0418	0.00608	6.68E-12	0.0093	0.0092	0.3122
rs2075865	A	C	A	C	0.4086	0.4135	0.02978	0.00492	1.47E-09	-0.0206	0.0076	0.006473
rs2092867	A	C	A	C	0.6472	0.6458	-0.02904	0.00502	7.40E-09	0.0088	0.0076	0.2423

SNP	effect_allele.exposure	other_allele.exposure	effect_allele.outcome	other_allele.outcome	eaf.exposure	eaf.outcome	beta.exposure	se.exposure	pval.exposure	beta.outcome	se.outcome	pval.outcome
rs211307	G	A	G	A	0.2462	0.247	-0.03564	0.0056	1.87E-10	-0.0064	0.0085	0.4536
rs2115859	C	T	C	T	0.6377	0.6449	0.0428	0.00504	2.32E-17	-0.0122	0.0078	0.1175
rs2129869	T	A	T	A	0.222	0.2203	-0.04088	0.00578	1.56E-12	0.0285	0.0089	0.001413
rs2156805	G	A	G	A	0.5117	0.5163	0.0344	0.00484	1.20E-12	0.0125	0.0074	0.09013
rs2165197	C	T	C	T	0.4895	0.4963	-0.03666	0.00482	2.99E-14	0.0013	0.0073	0.8624
rs2178895	T	C	T	C	0.6391	0.638	-0.0351	0.00504	3.65E-12	0.0194	0.0078	0.01272
rs2224858	G	A	G	A	0.815	0.8171	-0.03578	0.00618	7.37E-09	0.015	0.0094	0.1115
rs2236295	T	G	T	G	0.3942	0.3886	0.04406	0.00498	8.47E-19	-0.0073	0.0078	0.3464
rs2236796	C	G	C	G	0.1604	0.1596	0.15948	0.00654	4.29E-131	-0.0132	0.01	0.1878
rs2238787	A	G	A	G	0.2901	0.2868	-0.04762	0.00532	4.12E-19	0.0146	0.0082	0.07462
rs2239268	A	G	A	G	0.6984	0.6973	-0.03718	0.00522	1.09E-12	0.0047	0.0079	0.5542
rs2279470	G	C	G	C	0.2155	0.2086	0.03438	0.0059	5.55E-09	0.001	0.009	0.9141
rs2281226	C	A	C	A	0.608	0.6085	0.03302	0.005	3.96E-11	3.00E-04	0.0076	0.9732
rs2286472	C	A	C	A	0.3197	0.3196	-0.04704	0.00522	2.29E-19	0.0039	0.0078	0.6196
rs2291433	G	T	G	T	0.5348	0.5387	0.04654	0.00484	7.13E-22	0.0013	0.0074	0.8613
rs2297603	T	G	T	G	0.1086	0.1057	-0.04774	0.0079	1.48E-09	0.0101	0.0118	0.3899
rs2306363	T	G	T	G	0.2013	0.1961	0.08092	0.00602	4.44E-41	-0.013	0.0092	0.1581
rs2315518	C	G	C	G	0.3377	0.3428	-0.02934	0.00506	6.99E-09	-0.0041	0.0076	0.5949
rs2327429	C	T	C	T	0.2919	0.2908	0.03558	0.00534	2.61E-11	0.0025	0.008	0.7523
rs2354862	C	A	C	A	0.3576	0.3567	0.03596	0.00506	1.09E-12	-0.006	0.0077	0.439
rs2370735	G	A	G	A	0.186	0.1814	-0.03524	0.0062	1.26E-08	-0.0118	0.0095	0.215
rs2392929	G	T	G	T	0.2021	0.2024	-0.14222	0.00602	1.59E-123	-0.0016	0.009	0.8566
rs2423514	G	A	G	A	0.4572	0.4526	0.05202	0.00486	7.99E-27	-0.0035	0.0075	0.6367
rs2493296	T	C	T	C	0.1437	0.1482	-0.06572	0.00696	4.12E-21	0.0054	0.0103	0.5961
rs2494196	A	C	A	C	0.2821	0.2801	0.03346	0.00536	4.28E-10	-0.0413	0.0083	5.80E-07
rs2540949	T	A	T	A	0.3805	0.3872	0.05212	0.00496	9.71E-26	-0.031	0.0077	5.11E-05
rs2569882	C	T	C	T	0.4349	0.4357	0.03964	0.00502	2.66E-15	0.0091	0.0074	0.2231
rs2574985	G	A	G	A	0.718	0.7311	-0.02986	0.00538	2.80E-08	0.0124	0.0084	0.1415
rs2589254	G	A	G	A	0.2525	0.251	-0.03726	0.0055	1.30E-11	-0.0053	0.0084	0.5281
rs2623079	G	C	G	C	0.381	0.3671	0.03922	0.00496	2.57E-15	-0.004	0.0077	0.6008
rs263017	G	A	G	A	0.508	0.5168	0.03814	0.00482	2.60E-15	0.0029	0.0074	0.6955
rs263533	T	C	T	C	0.4202	0.4184	0.03212	0.00492	6.71E-11	-0.0325	0.0075	1.58E-05
rs2689690	T	C	T	C	0.3629	0.355	0.0417	0.00506	1.82E-16	-0.0074	0.0078	0.3434
rs2744133	G	A	G	A	0.2742	0.2731	0.05242	0.00542	3.65E-22	0.0098	0.0084	0.2421
rs2753960	T	G	T	G	0.424	0.434	-0.07302	0.0049	2.04E-50	0.0033	0.0073	0.6543
rs2815063	A	C	A	C	0.1314	0.1286	-0.0464	0.00734	2.75E-10	0.0121	0.0115	0.293
rs2833834	A	C	A	C	0.2797	0.2828	-0.03816	0.0054	1.63E-12	0.0015	0.0083	0.857

SNP	effect_allele.exposure	other_allele.exposure	effect_allele.outcome	other_allele.outcome	eaf.exposure	eaf.outcome	beta.exposure	se.exposure	pval.exposure	beta.outcome	se.outcome	pval.outcome
rs28421137	G	A	G	A	0.3776	0.3837	0.02894	0.00506	1.03E-08	0.005	0.0075	0.5052
rs28515053	A	G	A	G	0.0299	0.0309	-0.09162	0.01432	1.58E-10	0.0077	0.0216	0.723
rs28578714	C	T	C	T	0.3918	0.39	0.03688	0.0051	4.71E-13	0.0216	0.0074	0.003703
rs28601620	T	C	T	C	0.1974	0.2017	-0.05578	0.0061	5.27E-20	-0.0019	0.0094	0.8421
rs2861484	T	G	T	G	0.2012	0.2054	0.04592	0.00604	2.99E-14	-0.0124	0.0092	0.1773
rs28688791	C	T	C	T	0.2006	0.2046	-0.06828	0.00606	1.69E-29	-0.0078	0.0092	0.3996
rs2868985	A	G	A	G	0.8038	0.8038	0.03424	0.00606	1.67E-08	0.0107	0.0091	0.2412
rs2872717	C	T	C	T	0.2444	0.2473	-0.04532	0.00562	7.95E-16	0.0173	0.0085	0.04119
rs2920926	G	A	G	A	0.7306	0.7331	-0.0518	0.00552	6.36E-21	0.0112	0.0086	0.1956
rs2943646	G	A	G	A	0.6433	0.646	-0.05418	0.005	1.94E-27	0.0625	0.0076	1.70E-16
rs3015451	A	C	A	C	0.7226	0.7228	0.02972	0.0054	3.52E-08	-0.0153	0.0083	0.06556
rs34130368	T	G	T	G	0.117	0.1172	0.0503	0.00774	7.87E-11	0.0013	0.0113	0.9071
rs34487963	A	C	A	C	0.0224	0.0313	0.13832	0.02022	7.95E-12	0.0279	0.0319	0.3815
rs34869093	G	A	G	A	0.3707	0.3719	-0.03516	0.00504	2.83E-12	-0.0122	0.0078	0.1165
rs34905952	A	G	A	G	0.16	0.1602	-0.04802	0.00658	2.78E-13	0.0174	0.01	0.08105
rs35107212	G	A	G	A	0.4021	0.3974	-0.04336	0.00496	2.53E-18	0.0189	0.0075	0.01207
rs35429	G	A	G	A	0.3874	0.3902	0.07822	0.00498	2.11E-55	0.004	0.0076	0.6037
rs35479618	A	G	A	G	0.0168	0.016	-0.18488	0.02306	1.08E-15	-0.0372	0.029	0.1993
rs35680304	T	C	T	C	0.5899	0.584	-0.0478	0.00496	5.86E-22	-0.0022	0.0076	0.7743
rs35783704	A	G	A	G	0.1009	0.0936	0.0879	0.00834	6.07E-26	0.0021	0.0136	0.88
rs35973496	T	C	T	C	0.4407	0.4436	-0.08456	0.00484	2.67E-68	-0.031	0.0073	2.36E-05
rs36010659	C	T	C	T	0.1408	0.1398	0.04636	0.00694	2.42E-11	0.0127	0.0107	0.2353
rs360153	C	T	C	T	0.5848	0.5908	-0.0558	0.00488	3.49E-30	-0.0192	0.0075	0.01002
rs36213074	C	T	C	T	0.1257	0.1272	-0.05206	0.00728	8.25E-13	2.00E-04	0.011	0.9843
rs365990	G	A	G	A	0.3681	0.3722	0.03434	0.00496	4.57E-12	0.0042	0.0075	0.5792
rs3752440	A	G	A	G	0.0843	0.0855	-0.06564	0.0091	5.67E-13	0.0116	0.014	0.4092
rs3756712	C	A	C	A	0.6241	0.6183	0.03016	0.00504	2.16E-09	0.0067	0.0075	0.3777
rs37624	A	G	A	G	0.6731	0.6734	0.03008	0.00516	5.46E-09	-0.0018	0.0079	0.8245
rs378769	A	G	A	G	0.2959	0.2968	-0.04942	0.00526	5.87E-21	-0.0018	0.008	0.8167
rs3795154	T	C	T	C	0.3621	0.3634	-0.03742	0.00506	1.33E-13	-0.0145	0.0077	0.06108
rs3796584	A	G	A	G	0.3623	0.3634	0.05506	0.00498	2.28E-28	-0.0138	0.0076	0.06737
rs3802228	G	A	G	A	0.5447	0.5454	0.0491	0.00484	4.09E-24	0.0193	0.0073	0.008484
rs3807925	G	A	G	A	0.3497	0.3486	-0.0295	0.00508	6.21E-09	-0.0038	0.0077	0.6182
rs3811640	A	C	A	C	0.2783	0.2694	0.0401	0.00536	8.05E-14	-0.0048	0.0082	0.5621
rs3814500	T	C	T	C	0.7035	0.7066	0.02954	0.00532	2.81E-08	0.0065	0.0081	0.4237
rs3815460	G	C	G	C	0.1032	0.105	-0.04574	0.00792	7.51E-09	-0.0023	0.0118	0.8427
rs3819536	A	G	A	G	0.7053	0.7089	-0.0351	0.00524	2.25E-11	0.0015	0.008	0.8475

SNP	effect_allele.exposure	other_allele.exposure	effect_allele.outcome	other_allele.outcome	eaf.exposure	eaf.outcome	beta.exposure	se.exposure	pval.exposure	beta.outcome	se.outcome	pval.outcome
rs3826537	G	A	G	A	0.4186	0.417	-0.03972	0.0049	4.60E-16	0.0137	0.0074	0.06507
rs3861882	C	T	C	T	0.2809	0.2803	0.0293	0.00536	4.79E-08	8.00E-04	0.0082	0.918
rs3918226	T	C	T	C	0.0804	0.0786	-0.1131	0.00934	8.94E-34	-0.0072	0.0147	0.622
rs3935505	G	T	G	T	0.2509	0.259	-0.031	0.0056	3.19E-08	-0.0107	0.0085	0.2091
rs398512	C	T	C	T	0.8605	0.8693	0.04722	0.00696	1.20E-11	-0.0039	0.0109	0.7206
rs4120852	T	G	T	G	0.6098	0.605	0.029	0.00494	4.13E-09	-0.0112	0.0075	0.1371
rs41508849	C	T	C	T	0.2137	0.2132	0.0349	0.00592	3.58E-09	0.0025	0.0091	0.7801
rs42032	A	G	A	G	0.2658	0.2694	0.05928	0.00546	1.88E-27	-0.0132	0.0082	0.1077
rs4260863	G	C	G	C	0.39	0.4011	0.03756	0.005	5.36E-14	-0.0056	0.0076	0.4558
rs4319878	T	C	T	C	0.5615	0.5619	-0.02712	0.00492	3.61E-08	0.0166	0.0075	0.02691
rs4374967	G	A	G	A	0.59	0.5864	0.03036	0.00488	5.33E-10	-0.0015	0.0074	0.8376
rs4433986	G	A	G	A	0.6046	0.6047	0.03766	0.00492	1.97E-14	-0.0027	0.0075	0.7186
rs4506565	T	A	T	A	0.3146	0.3192	-0.04514	0.00518	3.20E-18	-0.0135	0.0078	0.08269
rs4511593	T	C	T	C	0.6518	0.6504	0.05168	0.0051	3.96E-24	-0.0066	0.0079	0.4003
rs4553000	T	C	T	C	0.5127	0.51	0.02942	0.00478	7.82E-10	-0.005	0.0073	0.494
rs4582532	A	G	A	G	0.5073	0.5088	0.03118	0.00482	9.51E-11	-0.0226	0.0073	0.001887
rs464605	T	C	T	C	0.7447	0.7447	-0.04238	0.00552	1.49E-14	0.0346	0.0083	3.34E-05
rs4666076	A	G	A	G	0.3455	0.3405	0.03044	0.00504	1.45E-09	-0.0065	0.0077	0.3991
rs4667454	G	A	G	A	0.3257	0.3187	0.04356	0.00518	3.84E-17	0.0021	0.008	0.7891
rs4691666	T	C	T	C	0.5325	0.5366	0.05686	0.00486	1.05E-31	-0.0186	0.0075	0.01292
rs4702102	T	A	T	A	0.6983	0.698	0.0361	0.00658	4.02E-08	-8.00E-04	0.0081	0.9247
rs4739832	C	A	C	A	0.4118	0.4025	0.03892	0.00494	3.04E-15	-0.0092	0.0076	0.2273
rs4746139	C	A	C	A	0.1417	0.1445	-0.08198	0.00698	7.00E-32	-0.0061	0.0104	0.5529
rs4746687	T	C	T	C	0.2184	0.2144	-0.03234	0.00584	3.07E-08	0.0024	0.0088	0.7869
rs4767332	A	C	A	C	0.5725	0.5653	-0.04368	0.00482	1.53E-19	-0.0112	0.0073	0.1286
rs4795637	C	T	C	T	0.2354	0.2377	-0.03738	0.00566	4.30E-11	0.0049	0.0086	0.5674
rs4812536	G	A	G	A	0.3422	0.3428	0.0431	0.00516	6.53E-17	0.005	0.0079	0.5247
rs4854572	A	G	A	G	0.4557	0.4504	0.03328	0.0049	1.04E-11	-0.0198	0.0076	0.009163
rs4859681	A	C	A	C	0.5296	0.5269	0.04128	0.00482	1.02E-17	-0.0298	0.0074	5.14E-05
rs4871964	G	A	G	A	0.2508	0.2499	0.06248	0.00558	3.63E-29	-0.0043	0.0085	0.6082
rs4888408	A	G	A	G	0.5851	0.5858	-0.0661	0.0049	1.70E-41	0.0146	0.0074	0.04891
rs4890500	G	A	G	A	0.2983	0.2921	-0.04766	0.00524	8.39E-20	0.0202	0.008	0.01205
rs4925159	A	G	A	G	0.4252	0.4233	-0.04368	0.00486	2.71E-19	0.0073	0.0074	0.3236
rs4938353	A	G	A	G	0.8235	0.8207	0.05728	0.00636	2.07E-19	0.0055	0.0095	0.5652
rs5030178	C	A	C	A	0.364	0.3663	-0.03606	0.00506	1.07E-12	0.0022	0.0078	0.7795
rs512730	A	T	A	T	0.194	0.1923	-0.04248	0.00608	3.01E-12	0.0189	0.0093	0.04169
rs551510	T	C	T	C	0.5037	0.4995	-0.02756	0.00488	1.65E-08	8.00E-04	0.0075	0.9124

SNP	effect_allele.exposure	other_allele.exposure	effect_allele.outcome	other_allele.outcome	eaf.exposure	eaf.outcome	beta.exposure	se.exposure	pval.exposure	beta.outcome	se.outcome	pval.outcome
rs55875326	C	G	C	G	0.2673	0.266	0.0416	0.00556	6.61E-14	-0.0211	0.0084	0.01266
rs55881012	C	A	C	A	0.4993	0.5001	-0.03878	0.00482	7.89E-16	0.0116	0.0074	0.1167
rs56249339	T	G	T	G	0.1132	0.1064	-0.0449	0.00768	4.81E-09	0.0051	0.0124	0.6824
rs56352451	T	C	T	C	0.1328	0.1345	-0.04018	0.00706	1.31E-08	0.0235	0.0106	0.02691
rs56725874	G	T	G	T	0.1194	0.1169	0.04388	0.00762	8.68E-09	-0.0047	0.0119	0.6931
rs569550	G	T	G	T	0.3964	0.3966	-0.09782	0.00506	2.01E-83	-0.004	0.0077	0.6006
rs57140819	G	C	G	C	0.173	0.1737	0.04044	0.00642	2.91E-10	-0.0107	0.0099	0.2798
rs57541197	A	G	A	G	0.14	0.1396	0.10672	0.00692	1.39E-53	-0.0217	0.0105	0.03901
rs57748895	T	A	T	A	0.0186	0.0193	-0.15862	0.01842	7.00E-18	-0.0223	0.0281	0.4283
rs58413618	C	T	C	T	0.3308	0.3219	-0.03572	0.00516	4.28E-12	-0.0115	0.0081	0.1562
rs597808	G	A	G	A	0.5094	0.5063	0.11228	0.00584	1.57E-82	-0.0343	0.0073	2.92E-06
rs5992134	T	G	T	G	0.2383	0.2405	-0.03114	0.0057	4.69E-08	0.011	0.0089	0.2161
rs5994376	C	A	C	A	0.5621	0.5676	-0.02886	0.00506	1.15E-08	0.0056	0.0076	0.4587
rs60121503	C	G	C	G	0.0843	0.0832	0.04768	0.00866	3.68E-08	0.0262	0.0132	0.04685
rs6017279	C	T	C	T	0.1345	0.1376	-0.07728	0.00702	3.85E-28	-0.0194	0.0106	0.06786
rs602662	A	G	A	G	0.5178	0.5136	-0.03336	0.00482	4.45E-12	0.0096	0.0073	0.1887
rs6026739	T	A	T	A	0.1236	0.1257	-0.1124	0.00738	2.27E-52	0.0021	0.0113	0.851
rs6062344	T	C	T	C	0.4969	0.4938	0.05262	0.0049	7.76E-27	-0.0239	0.0074	0.001194
rs6078093	A	G	A	G	0.4244	0.4208	0.03476	0.00486	8.77E-13	0.0133	0.0074	0.07202
rs6085527	G	A	G	A	0.5938	0.605	-0.039	0.00492	2.44E-15	-0.0189	0.0077	0.01378
rs6088749	G	C	G	C	0.165	0.1667	0.04752	0.00648	2.22E-13	0.0069	0.0098	0.4832
rs60909079	C	G	C	G	0.2492	0.2489	0.03996	0.00558	8.02E-13	0.0047	0.0084	0.5739
rs61559025	G	T	G	T	0.0626	0.0569	-0.06068	0.0103	3.94E-09	0.019	0.0161	0.2394
rs61772592	G	A	G	A	0.1241	0.1214	-0.05386	0.0074	3.29E-13	0.0334	0.0117	0.004324
rs61789367	A	G	A	G	0.0451	0.0461	-0.0671	0.01198	2.10E-08	-0.0091	0.0188	0.6265
rs61915422	G	C	G	C	0.129	0.1291	0.04068	0.00738	3.55E-08	-0.0108	0.0113	0.3354
rs62050280	A	G	A	G	0.048	0.0452	-0.12794	0.01466	2.53E-18	0.0157	0.0201	0.4347
rs62158170	G	A	G	A	0.2167	0.2183	0.03866	0.0059	6.11E-11	-0.0069	0.0091	0.4448
rs62271373	A	T	A	T	0.0592	0.0562	-0.08778	0.01078	4.06E-16	0.0411	0.0174	0.01835
rs62434125	C	T	C	T	0.0691	0.0675	0.1387	0.0095	2.65E-48	-0.0155	0.0146	0.2894
rs62480403	G	C	G	C	0.3177	0.3149	0.03886	0.00524	1.21E-13	0.0131	0.0081	0.1068
rs633880	C	T	C	T	0.8643	0.8697	0.05056	0.00718	1.83E-12	-0.0039	0.011	0.7272
rs640935	C	T	C	T	0.2396	0.2338	-0.03448	0.00566	1.13E-09	-0.0134	0.0087	0.1239
rs6414805	C	T	C	T	0.2573	0.2571	0.03558	0.00548	8.48E-11	0.0082	0.0083	0.3266
rs6438857	C	T	C	T	0.4252	0.4315	0.0429	0.0049	2.29E-18	0.0155	0.0076	0.04134
rs6445819	T	C	T	C	0.3127	0.3054	0.04958	0.00522	1.89E-21	0.0075	0.008	0.3481
rs6517423	A	G	A	G	0.2957	0.2922	-0.02956	0.00528	2.17E-08	0.0042	0.0082	0.6082

SNP	effect_all ele. exposure	other_all ele. exposure	effect_all ele. outcome	other_all ele. outcome	eaf.exposure	eaf.outcome	beta.exposure	se.exposure	pval.exposure	beta.outcome	se.outcome	pval.outcome
rs6543523	G	A	G	A	0.2547	0.262	-0.03294	0.0056	4.25E-09	0.0045	0.0086	0.5996
rs6723772	T	C	T	C	0.1048	0.1047	0.04544	0.00804	1.55E-08	-0.0291	0.0129	0.02391
rs6732308	G	A	G	A	0.2028	0.2023	0.05754	0.00604	1.81E-21	0.0017	0.0094	0.8598
rs6734118	A	C	A	C	0.2167	0.2205	0.06434	0.00694	1.87E-20	0.0063	0.0087	0.4694
rs67427762	T	C	T	C	0.1551	0.1581	0.0694	0.00702	4.78E-23	-0.0053	0.0103	0.6032
rs6745430	A	C	A	C	0.1761	0.1739	0.04004	0.00632	2.49E-10	0.0023	0.0097	0.8103
rs67468157	T	C	T	C	0.3119	0.3083	-0.03142	0.0052	1.51E-09	0.0069	0.008	0.3861
rs6777317	A	G	A	G	0.2955	0.3022	-0.03428	0.00536	1.71E-10	0.0076	0.008	0.3445
rs6778977	G	T	G	T	0.3421	0.3452	-0.03594	0.00512	2.25E-12	-0.0057	0.0078	0.4652
rs67909753	A	G	A	G	0.2894	0.2896	0.04502	0.00532	2.44E-17	-0.0011	0.0081	0.8903
rs682681	C	T	C	T	0.668	0.6705	-0.04434	0.00516	9.46E-18	-0.0021	0.008	0.7933
rs688540	A	G	A	G	0.8667	0.8726	-0.04498	0.0073	6.96E-10	-0.0126	0.0112	0.2616
rs6890251	T	C	T	C	0.1579	0.1574	-0.07282	0.00664	5.52E-28	0.0105	0.0102	0.3017
rs689304	T	C	T	C	0.0974	0.1	0.07922	0.00816	3.00E-22	-0.0195	0.0125	0.1193
rs6905288	A	G	A	G	0.5671	0.5665	-0.03282	0.00492	2.50E-11	0.0348	0.0073	2.00E-06
rs6910072	G	A	G	A	0.1882	0.1845	0.0376	0.00622	1.46E-09	0.0011	0.0097	0.9096
rs6921610	T	C	T	C	0.5371	0.5403	0.03232	0.00484	2.23E-11	-0.006	0.0073	0.4105
rs6926566	T	C	T	C	0.4277	0.4285	0.03108	0.00484	1.45E-10	0.0019	0.0074	0.8004
rs6961048	G	C	G	C	0.1046	0.1062	-0.10164	0.0079	7.03E-38	-0.0369	0.0119	0.002011
rs699	G	A	G	A	0.4117	0.4165	-0.0654	0.00488	8.01E-41	0.0143	0.0074	0.0529
rs7006340	G	A	G	A	0.3054	0.3107	-0.03248	0.00528	7.19E-10	0.0033	0.0081	0.6818
rs7012866	G	T	G	T	0.5004	0.494	-0.04024	0.0048	5.38E-17	0.0196	0.0073	0.007234
rs7021547	G	A	G	A	0.1964	0.1974	0.03464	0.00606	1.08E-08	0.0068	0.0094	0.4688
rs7023828	T	C	T	C	0.4165	0.4125	0.05076	0.00492	4.91E-25	-0.0075	0.0076	0.3224
rs7045409	A	T	A	T	0.3705	0.3803	0.03532	0.00504	2.25E-12	-0.0194	0.0078	0.01321
rs709668	G	A	G	A	0.7968	0.7936	-0.05168	0.00604	1.08E-17	0.0055	0.0093	0.5544
rs7099692	C	G	C	G	0.7118	0.7154	0.03592	0.00536	2.21E-11	-0.0107	0.0082	0.1918
rs7101864	G	A	G	A	0.603	0.6069	-0.02936	0.00494	2.77E-09	0.0111	0.0076	0.1433
rs7107356	G	A	G	A	0.5041	0.506	-0.07748	0.0048	2.08E-58	0.0086	0.0073	0.2393
rs7125196	C	T	C	T	0.1181	0.115	0.06834	0.00748	7.19E-20	9.00E-04	0.0113	0.9363
rs71499040	C	G	C	G	0.7075	0.7109	-0.03636	0.00536	1.13E-11	-0.006	0.0081	0.4601
rs7213273	A	G	A	G	0.6562	0.657	0.06468	0.00504	1.42E-37	0.0147	0.0077	0.05668
rs7236548	A	C	A	C	0.1856	0.1854	-0.05638	0.0062	9.74E-20	-0.003	0.0095	0.7495
rs7248104	A	G	A	G	0.4123	0.4125	0.04334	0.00486	5.24E-19	-0.0329	0.0074	7.67E-06
rs7253742	C	T	C	T	0.4077	0.4118	-0.03248	0.00486	2.52E-11	0.0043	0.0074	0.5598
rs72661946	C	G	C	G	0.0868	0.09	-0.06242	0.00912	7.89E-12	-7.00E-04	0.014	0.9587
rs72683923	C	T	C	T	0.0202	0.0187	0.19354	0.01824	2.57E-26	-0.1034	0.0302	0.000616

SNP	effect_allele.exposure	other_allele.exposure	effect_allele.outcome	other_allele.outcome	eaf.exposure	eaf.outcome	beta.exposure	se.exposure	pval.exposure	beta.outcome	se.outcome	pval.outcome
rs72719160	T	A	T	A	0.3199	0.3268	-0.04576	0.00516	8.54E-19	0.0179	0.0079	0.02234
rs7278003	C	T	C	T	0.5648	0.5682	-0.03716	0.00484	1.84E-14	0.007	0.0073	0.339
rs72792102	G	A	G	A	0.0415	0.0391	-0.08118	0.01246	7.34E-11	0.037	0.0198	0.06213
rs72844527	A	C	A	C	0.1652	0.1584	-0.04102	0.00652	2.95E-10	0.0109	0.0101	0.282
rs72976750	C	T	C	T	0.1386	0.1364	-0.09376	0.00704	1.41E-40	3.00E-04	0.0109	0.9748
rs7302981	G	A	G	A	0.623	0.6227	0.07106	0.00492	2.10E-47	-0.0189	0.0075	0.01134
rs73033340	G	A	G	A	0.0302	0.0293	0.14466	0.01794	7.56E-16	0.0038	0.0249	0.879
rs73049928	G	A	G	A	0.1913	0.1831	-0.03898	0.0063	6.08E-10	0.0033	0.0097	0.7326
rs7306710	C	T	C	T	0.5238	0.534	-0.0419	0.00486	7.20E-18	0.0189	0.0076	0.01226
rs73075656	T	G	T	G	0.1339	0.1372	0.05104	0.00714	8.78E-13	0.0063	0.011	0.5679
rs73075659	G	A	G	A	0.3315	0.3265	0.06862	0.00518	5.56E-40	-0.0044	0.0081	0.5873
rs73156495	T	C	T	C	0.1646	0.1611	-0.04166	0.00648	1.29E-10	-0.0016	0.0098	0.8728
rs7316233	G	A	G	A	0.1453	0.1452	-0.0486	0.00684	1.27E-12	-0.0015	0.0103	0.8833
rs7320104	G	A	G	A	0.1529	0.1585	-0.0761	0.00684	1.02E-28	-0.0026	0.0105	0.8076
rs73226179	A	G	A	G	0.0598	0.0575	-0.05726	0.01028	2.49E-08	0.0348	0.0158	0.0272
rs7341594	A	G	A	G	0.2197	0.2191	-0.05682	0.00582	1.55E-22	0.0153	0.0088	0.08241
rs73551577	A	G	A	G	0.1905	0.1865	0.06766	0.00618	6.84E-28	-0.0153	0.0098	0.1168
rs7395791	A	G	A	G	0.4415	0.4406	0.03844	0.00492	5.26E-15	-0.0087	0.0075	0.2435
rs7412	T	C	T	C	0.081	0.0803	0.07842	0.00904	4.03E-18	-0.0365	0.0134	0.006399
rs7427249	A	G	A	G	0.579	0.5785	0.03996	0.00488	2.62E-16	-0.003	0.0074	0.6808
rs74516251	A	G	A	G	0.079	0.082	0.09182	0.00912	7.63E-24	0.0129	0.0142	0.3629
rs74606343	C	T	C	T	0.0877	0.0868	0.05704	0.00866	4.60E-11	-0.0202	0.0134	0.1331
rs74625348	C	G	C	G	0.2311	0.2303	0.03588	0.00588	1.06E-09	-0.0255	0.009	0.00462
rs74900445	C	T	C	T	0.1196	0.1225	-0.0561	0.00744	4.58E-14	0.0094	0.0112	0.4003
rs74926649	A	G	A	G	0.0163	0.016	-0.13296	0.01944	7.81E-12	-0.0325	0.03	0.278
rs7493678	T	A	T	A	0.3454	0.3408	-0.03138	0.0051	7.70E-10	-0.0084	0.0079	0.2883
rs7514579	C	A	C	A	0.2298	0.2303	0.03834	0.00578	3.07E-11	-0.0135	0.0088	0.1269
rs7547570	A	G	A	G	0.3818	0.3862	-0.03794	0.00498	2.30E-14	0.0142	0.0076	0.06293
rs75672964	T	C	T	C	0.043	0.0456	-0.09672	0.01308	1.43E-13	-0.039	0.0189	0.03902
rs7575637	A	G	A	G	0.4581	0.4592	0.04422	0.00482	5.13E-20	-0.0036	0.0074	0.621
rs7578166	C	A	C	A	0.617	0.6251	0.02808	0.00496	1.51E-08	0.0222	0.0077	0.003974
rs7632040	G	A	G	A	0.6031	0.6005	-0.05884	0.00494	9.71E-33	0.0049	0.0076	0.52
rs76326501	C	A	C	A	0.0889	0.0862	0.11188	0.00864	2.40E-38	-0.0173	0.0135	0.2
rs76452347	T	C	T	C	0.2053	0.2063	0.04408	0.00618	9.93E-13	0.0035	0.009	0.6961
rs7677290	C	G	C	G	0.3902	0.3864	-0.0332	0.00496	2.08E-11	0.0121	0.0076	0.1111
rs7703751	T	A	T	A	0.2554	0.2534	0.05036	0.00554	1.13E-19	0.0046	0.0086	0.5937
rs770379	G	A	G	A	0.5465	0.5519	0.0282	0.00482	5.23E-09	0.0131	0.0075	0.07942

SNP	effect_allele.exposure	other_allele.exposure	effect_allele.outcome	other_allele.outcome	eaf.exposure	eaf.outcome	beta.exposure	se.exposure	pval.exposure	beta.outcome	se.outcome	pval.outcome
rs7703932	T	C	T	C	0.4776	0.4752	-0.03402	0.00482	1.62E-12	2.00E-04	0.0073	0.9823
rs77175710	G	A	G	A	0.1505	0.1512	-0.03888	0.00692	1.86E-08	-0.0145	0.0106	0.1724
rs7733331	C	T	C	T	0.5996	0.5956	-0.10986	0.0049	4.23E-111	0.0142	0.0074	0.05645
rs778124	A	G	A	G	0.3759	0.3796	-0.05026	0.00496	4.65E-24	0.0047	0.0076	0.5337
rs78140146	T	G	T	G	0.0454	0.0472	-0.07026	0.01222	8.79E-09	-0.0484	0.0182	0.007867
rs7814142	A	G	A	G	0.5865	0.5817	0.0658	0.00488	2.26E-41	-0.0329	0.0074	7.60E-06
rs78154752	G	T	G	T	0.2075	0.2123	-0.05218	0.006	3.84E-18	0.0186	0.0093	0.04621
rs7819550	A	G	A	G	0.1922	0.1898	0.0361	0.00616	4.56E-09	-0.0078	0.0095	0.4104
rs78474310	G	A	G	A	0.0435	0.0419	-0.09648	0.01186	4.18E-16	0.0155	0.0182	0.3921
rs7912283	A	G	A	G	0.6473	0.6487	0.04002	0.00512	5.88E-15	0.0026	0.0077	0.7367
rs79349366	T	C	T	C	0.0326	0.0323	0.10272	0.01428	6.21E-13	-0.0544	0.0214	0.01108
rs79376802	C	T	C	T	0.0518	0.0511	0.07802	0.0111	2.17E-12	-0.013	0.0168	0.4404
rs79523138	G	A	G	A	0.1162	0.1073	-0.05426	0.0078	3.30E-12	-0.0142	0.0129	0.2694
rs7963397	T	C	T	C	0.3951	0.4024	-0.02792	0.00492	1.41E-08	0.0029	0.0076	0.7027
rs79782817	T	G	T	G	0.1034	0.1064	-0.08542	0.00792	3.55E-27	-0.0065	0.0118	0.581
rs7979246	G	A	G	A	0.6717	0.6737	0.02836	0.00514	3.65E-08	-0.0098	0.0078	0.2099
rs7982886	A	G	A	G	0.1368	0.1336	0.04542	0.00702	9.85E-11	-0.0039	0.0108	0.7174
rs79909779	A	G	A	G	0.1382	0.1312	-0.04456	0.00722	6.93E-10	0.016	0.0112	0.1537
rs80100774	G	T	G	T	0.0771	0.0685	0.06356	0.01024	5.54E-10	-0.0138	0.0176	0.4336
rs8030856	G	C	G	C	0.4	0.4083	-0.02756	0.00496	2.66E-08	0.0077	0.0076	0.3131
rs8046196	T	G	T	G	0.5131	0.5139	-0.03	0.00484	5.95E-10	0.0096	0.0075	0.1975
rs8108717	G	A	G	A	0.6119	0.6212	0.04542	0.00496	5.39E-20	0.0028	0.0076	0.7122
rs8125763	A	C	A	C	0.4731	0.478	-0.02918	0.00484	1.54E-09	0.0053	0.0074	0.4749
rs817140	C	T	C	T	0.2758	0.2739	-0.03216	0.0054	2.52E-09	-0.0049	0.0083	0.5583
rs866573	T	C	T	C	0.1553	0.1592	-0.05736	0.00666	6.55E-18	-0.0285	0.0099	0.00405
rs871606	C	T	C	T	0.1063	0.1086	0.07144	0.00802	5.56E-19	-0.008	0.0128	0.5338
rs885769	C	T	C	T	0.6378	0.6324	-0.03274	0.00504	8.16E-11	0.0012	0.0076	0.8768
rs8904	A	G	A	G	0.3699	0.3745	-0.05638	0.00498	1.01E-29	0.0228	0.0075	0.002342
rs9122	C	T	C	T	0.507	0.5018	0.03016	0.00482	3.69E-10	-0.0288	0.0073	8.55E-05
rs9221	T	C	T	C	0.9114	0.9117	-0.0743	0.00852	2.86E-18	0.0229	0.0131	0.08001
rs924653	A	G	A	G	0.5208	0.5226	0.03102	0.0048	1.05E-10	0.0074	0.0073	0.3072
rs9368222	A	C	A	C	0.2676	0.2673	-0.04552	0.00542	4.12E-17	0.0045	0.0082	0.5813
rs9403140	A	G	A	G	0.6807	0.6895	-0.03266	0.00522	3.85E-10	0.0095	0.008	0.2312
rs9515270	T	C	T	C	0.1779	0.1768	-0.0363	0.00636	1.19E-08	0.0223	0.0097	0.02161
rs9532243	C	A	C	A	0.5185	0.5232	0.05358	0.0048	5.58E-29	0.0089	0.0073	0.2212
rs9675039	A	G	A	G	0.3673	0.3614	-0.02918	0.0051	1.07E-08	-0.0026	0.0076	0.7366
rs9809556	A	G	A	G	0.3925	0.3959	-0.05846	0.00494	2.21E-32	-0.0104	0.0076	0.1685

SNP	effect_allele.exposure	other_allele.exposure	effect_allele.outcome	other_allele.outcome	eaf.exposure	eaf.outcome	beta.exposure	se.exposure	pval.exposure	beta.outcome	se.outcome	pval.outcome
rs9833313	T	A	T	A	0.757	0.7558	-0.03158	0.0057	3.06E-08	0.0073	0.0088	0.4065
rs9842387	G	A	G	A	0.4633	0.4603	-0.02658	0.00484	4.15E-08	-0.0151	0.0074	0.04142
rs988398	T	C	T	C	0.4424	0.4474	-0.06074	0.00484	3.28E-36	0.0014	0.0074	0.8519
rs9928003	C	A	C	A	0.1788	0.1787	0.05488	0.0063	2.66E-18	0.0027	0.0096	0.7788
rs9937801	C	T	C	T	0.4295	0.4298	0.0351	0.00482	3.30E-13	-0.0028	0.0073	0.6986
rs9992763	T	G	T	G	0.5692	0.5726	0.03456	0.00488	1.35E-12	5.00E-04	0.0075	0.9478

Table S7. Effect of overall systolic blood pressure reduction and major antihypertensive drug classes on non-alcoholic fatty liver disease, replicated using genome-wide association studies that employed diagnostic codes for identifying non-alcoholic fatty liver disease

Analysis	Primary (MVP)	Replication (4 EU cohorts)	Direction Consistent?
Overall SBP → NAFLD (per 5 mmHg)	OR 0.92 (0.90–0.94)	OR 0.95 (0.92–0.98)	Yes
RAS inhibitors → NAFLD	OR 0.73 (0.62–0.85)	OR 0.58 (0.43–0.80)	Yes
Beta-blockers → NAFLD	OR 0.88 (0.67–1.16)	OR 0.76 (0.60–0.97)	Yes
Calcium channel blockers → NAFLD	OR 0.97 (0.89–1.06)	OR 0.82 (0.66–1.02)	Yes
Diuretics → NAFLD	OR 0.94 (0.87–1.02)	OR 0.97 (0.82–1.15)	Yes

Table S8. Whole-genome Mendelian randomisation estimate of systolic blood pressure reduction on the risk of NAFLD, accounting for residual linkage disequilibrium (LD-robustness analysis)

Method	Number of SNPs	OR (per 5 mmHg reduction in SBP)	95% CI
Inverse-variance weighted, random-effects (primary)	410	0.92	0.90–0.94
Inverse-variance weighted, fixed-effect	410	0.92	0.90–0.93
Weighted median	410	0.93	0.91–0.96
MR-Egger (slope)	410	1.00	0.94–1.06

Note: instruments were clumped at $r^2 < 0.001$, so the selected variants are effectively independent and residual linkage disequilibrium between instruments is negligible; the inverse-variance weighted estimate is therefore robust to residual correlation among instruments. The MR-Egger intercept was −0.005 (SE 0.002, P = 0.005), consistent with the modest directional pleiotropy reported in the main text and addressed through exclusion of outlier variants (Appendix 18). Heterogeneity: Cochran Q = 1015.6, I^2 = 60%. Abbreviations: SNP, single nucleotide polymorphism; SBP, systolic blood pressure; OR, odds ratio; CI, confidence interval; IVW, inverse-variance weighted.

Table S9. Extended multivariable Mendelian randomisation: direct effect of systolic blood pressure on NAFLD, jointly adjusted for BMI, waist-to-hip ratio, HbA1c and LDL cholesterol

All exposures European ancestry. Genetic instruments were SNPs reaching genome-wide significance ($P < 5\times10^{-8}$) for any exposure, clumped to independence ($r^2 < 0.001$, 10 Mb) using a 1000 Genomes European reference panel; all exposures were harmonised to a common rsID set by chromosome:position:alleles. The direct effect of SBP is expressed per 5 mmHg lower SBP; covariate effects are per standard-deviation higher trait (HbA1c per trait unit).

Exposure	OR on NAFLD (95% CI)	P value	Instruments (n)
Systolic blood pressure (per 5 mmHg lower)	0.92 (0.87–0.96)	3.0×10^{-4}	165
Body mass index	1.26 (1.08–1.47)	3.4×10^{-3}	101
Waist-to-hip ratio (adjusted for BMI)	1.68 (1.43–1.99)	8.6×10^{-10}	86
LDL cholesterol	1.26 (1.13–1.40)	2.4×10^{-5}	182
HbA1c	0.94 (0.68–1.31)	0.72	23

The direct effect of systolic blood pressure on NAFLD (OR 0.92, 95% CI 0.87–0.96 per 5 mmHg lower SBP) is essentially identical to the univariable whole-genome estimate (OR 0.92, 95% CI 0.90–0.94), indicating that the blood pressure–NAFLD association is not explained by adiposity, glycaemic or lipid traits. Waist-to-hip ratio, HbA1c and LDL cholesterol are correlated with adiposity and may lie on the causal pathway; this extended model is therefore a sensitivity analysis, with BMI retained as the primary multivariable adjustment. Multivariable

MR estimated with the inverse-variance-weighted method (TwoSampleMR::mv_multiple).Data sources (all European-ancestry GWAS summary statistics). Systolic blood pressure (exposure of interest; per 5 mmHg lower): Keaton et al., Nature Genetics 2024 (n = 1,028,980; GWAS Catalog GCST90310294) — the primary-analysis SBP GWAS. Body mass index: UK Biobank, MRC-IEU pipeline (IEU OpenGWAS ukb-b-19953; n ≈ 461,460). Waist-to-hip ratio adjusted for BMI: Pulit et al., Human Molecular Genetics 2019 (GIANT + UK Biobank; n ≤ 694,649). HbA1c: MAGIC, Chen et al., Nature Genetics 2021 (European ancestry). LDL cholesterol: Global Lipids Genetics Consortium, Graham et al., Nature 2021 (European ancestry). Outcome — NAFLD (unexplained chronic ALT elevation, Million Veteran Program): Vujkovic et al., Nature Genetics 2022 (European-ancestry subset, 68,725 cases / 95,472 controls) — the primary-analysis outcome GWAS. Instruments were SNPs with $P < 5\times10^{-8}$ for any exposure, clumped at $r^2 < 0.001$ (10 Mb) on a 1000 Genomes Phase 3 European reference panel; all datasets harmonised to a common variant set by chromosome:position:alleles. Direct effects estimated by inverse-variance-weighted multivariable MR (TwoSampleMR::mv_multiple). The SBP GWAS and the GLGC LDL GWAS both include Million Veteran Program participants and so partly overlap the outcome sample; given the strong instruments this has negligible influence on the SBP estimate. Effects are per 5 mmHg lower SBP, and per standard-deviation higher trait for BMI, WHRadjBMI and LDL (HbA1c per trait unit). OR, odds ratio; CI, confidence interval.

Table S10. Initially retrieved target genes for commonly prescribed antihypertensive drugs

Drug class	Drug generic name	Target gene
**Renin-angiotensin system (RAS) inhibitors**	Benazepril	ACE
	Candesartan	AGTR1
	Captopril	MMP9, BDKRB1, LTA4H, ACE, MMP2
	Enalapril	ACE
	Eprosartan	AGTR1
	Fosinopril	ACE
	Irbesartan	AGTR1, JUN
	Lisinopril	ACE, REN
	Losartan	AGTR1
	Moexipril	ACE
	Perindopril	ACE, SFRP4
	Quinapril	ACE
	Ramipril	BDKRB1, ACE
	Telmisartan	PPARG, AGTR1
	Trandolapril	ACE
	Valsartan	AGTR1
**Beta blockers (BBs)**	Acebutolol	ADRB2, ADRB1
	Atenolol	ADRB1, ADRB2
	Betaxolol	ADRB2, ADRB1
	Bisoprolol	ADRB1, ADRB2
	Carvedilol	HIF1A, NDUFC2, ADRA1A, KCNJ2, ADRA1D, VCAM1, SELE, VEGFA, GJA1, ADRA2B, ADRB2, KCNH2, ADRB1, ADRA1B, KCNJ4, NPPB, ADRA2C, ADRA2A
	Esmolol	ADRB1
	Labetalol	ADRA1A, ADRB1, ADRA1D, ADRB2, ADRA1B
	Metoprolol	ADRB2, ADRB1
	Nadolol	ADRB2, ADRB1
	Nebivolol	ADRB3, ADRB1, ADRB2
	Pindolol	ADRB3, ADRB2, HTR1B, ADRB1, HTR1A
	Propranolol	ADRB1, ADRB2, ADRB3, HTR1A, HTR1B
	Sotalol	ADRB1, ADRB2, KCNH2
	Timolol	ADRB1, ADRB2, E
**Calcium channel blocker (CCBs)**	Amlodipine	CACNB1, CACNA1C, SMPD1, CACNA2D3, CACNA1B, CACNA1I, CA1
	Diltiazem	CACNA1C, CACNG1
	Felodipine	CACNA2D2, CACNB2, CALM1, TNNC2, PDE1B, PDE1A, CACNA1S, TNNC1, CACNA2D1, CACNA1D, CACNA1H, NR3C2, CACNA1C
	Isradipine	CACNA2D2, CACNB2, CACNA2D1, CACNA1H, CACNA1C, CACNA1D, CACNA1S
	Nicardipine	CACNA2D1, CACNA1C, CACNA1H, CHRM2, PDE1A, CACNB2, CACNA1G, CACNA1I, ADRA1A, ADRA1B, CALM1, CACNA1D, PDE1B, ADRA1D, CHRM1, CHRM5, CHRM4, CHRM3
	Nifedipine	CACNA1S, CALM1, CACNA1G, CACNA1C, CACNB2, NR1I2, CACNA1D, KCND3, CACNA1I, CACNA1H
	Nimodipine	CACNA1F, CACNA1C, CACNB3, CACNB1, CACNB2, CACNA1S, CACNA1D, NR3C2, CACNB4, AHR

Drug class	Drug generic name	Target gene
	Nisoldipine	CACNA1C, CACNA2D1, CACNA1D, CACNB2, CACNA1S
	Verapamil	ABCB1, ADRA1A, CACNA1B, KCNJ11, CACNA1H, CACNA1C, CACNA1G, ADRA1B, CACNA1A, SLC6A4, ADRA1D, KCNH2
**Diuretics**	Amiloride	ASIC1, SLC9A1, PLAU, SCNN1D, SCNN1A, SCNN1B, AOC1, SCNN1G, ASIC2
	Bendroflumethiazide	CA2, CA4, CA1, KCNMA1, SLC12A3
	Bumetanide	SLC12A5, CFTR, SLC12A4, SLC12A1, SLC12A2
	Chlorthalidone	CA1, SLC12A1
	Eplerenone	NR3C2
	Furosemide	CA2, SLC12A1, GPR35
	Hydrochlorothiazide	KCNMA1, SLC12A3
	Hydroflumethiazide	CA9, ATP1A1, CA7, CA1, CA12, CA4, CA2, KCNMA1, SLC12A1
	Indapamide	SLC12A3
	Methyclothiazide	CA4, SLC12A1, CA2, CA1
	Metolazone	SLC12A3
	Polythiazide	SLC12A3
	Spironolactone	ESR1, CACNB3, CACNA1D, ESR2, CACNB4, CACNA1S, CACNA1F, CACNB1, CACNA1C, CACNA1A, CACNB2
	Triamterene	SCNN1A, SCNN1B, SCNN1D, SCNN1G
	Trichlormethiazide	CA1, CA2, SLC12A3, ATP1A1, CA4

Table S11. Positional information of target genes for commonly prescribed antihypertensive drugs

Gene name	Gene id	Chromosome	Position
ABCB1	ENSG00000085563	7	87133175-87342611
ACE	ENSG00000159640	17	61554422-61599205
ADRA1A	ENSG00000120907	8	26605667-26724790
ADRA1B	ENSG00000170214	5	159343790-159399551
ADRA1D	ENSG00000171873	20	4201329-4229721
ADRA2A	ENSG00000150594	10	112836790-112840658
ADRA2B	ENSG00000222040	2	96778707-96781984
ADRA2C	ENSG00000184160	4	3768075-3770251
ADRB1	ENSG00000043591	10	115803806-115806667
ADRB2	ENSG00000169252	5	148206156-148208196
ADRB3	ENSG00000188778	8	37820516-37824483
AGTR1	ENSG00000144891	3	148415571-148460795
AHR	ENSG00000106546	7	17338246-17385776
AOC1	ENSG00000002726	7	150521715-150558592
ASIC1	ENSG00000110881	12	50451331-50477394
ASIC2	ENSG00000108684	17	31340105-32501983
ATP1A1	ENSG00000163399	1	116915290-116952883
BDKRB1	ENSG00000100739	14	96722161-96735304
CA1	ENSG00000133742	8	86239837-86291243
CA12	ENSG00000074410	15	63613577-63674360
CA2	ENSG00000104267	8	86376081-86393722
CA4	ENSG00000167434	17	58227297-58248260
CA7	ENSG00000168748	16	66878282-66888056
CA9	ENSG00000107159	9	35673853-35681156
CACNA1A	ENSG00000141837	19	13317256-13734804
CACNA1B	ENSG00000148408	9	140772241-141019076
CACNA1C	ENSG00000151067	12	2079952-2802108
CACNA1D	ENSG00000157388	3	53528683-53847760
CACNA1F	ENSG00000102001	X	49061523-49089833
CACNA1G	ENSG00000006283	17	48638429-48704835
CACNA1H	ENSG00000196557	16	1203241-1271771
CACNA1I	ENSG00000100346	22	39966758-40085742
CACNA1S	ENSG00000081248	1	201008642-201081694
CACNA2D1	ENSG00000153956	7	81575760-82073114
CACNA2D2	ENSG00000007402	3	50400233-50541675
CACNA2D3	ENSG00000157445	3	54156574-55108584
CACNB1	ENSG00000067191	17	37329709-37353956
CACNB2	ENSG00000165995	10	18429606-18830798
CACNB3	ENSG00000167535	12	49207577-49222724
CACNB4	ENSG00000182389	2	152689290-152955593
CACNG1	ENSG00000108878	17	65040706-65052909
CALM1	ENSG00000198668	14	90862846-90874605
CFTR	ENSG00000001626	7	117105838-117356025
CHRM1	ENSG00000168539	11	62676151-62689279
CHRM2	ENSG00000181072	7	136553416-136705002
CHRM3	ENSG00000133019	1	239549865-240078750
CHRM4	ENSG00000180720	11	46406640-46408107
CHRM5	ENSG00000184984	15	34260921-34357291
ESR1	ENSG00000091831	6	151977826-152450754
ESR2	ENSG00000140009	14	64550950-64804830
GJA1	ENSG00000152661	6	121756838-121770873

Gene name	Gene id	Chromosome	Position
GPR35	ENSG00000178623	2	241544848-241570676
HIF1A	ENSG00000100644	14	62162231-62214976
HTR1A	ENSG00000178394	5	63256183-63258334
HTR1B	ENSG00000135312	6	78171948-78173490
JUN	ENSG00000177606	1	59246465-59249785
KCND3	ENSG00000171385	1	112313284-112531777
KCNH2	ENSG00000055118	7	150642049-150675403
KCNJ11	ENSG00000187486	11	17407406-17410878
KCNJ2	ENSG00000123700	17	68164814-68176189
KCNJ4	ENSG00000168135	22	38822332-38851205
KCNMA1	ENSG00000156113	10	78629359-79398353
LTA4H	ENSG00000111144	12	96394606-96437298
MMP2	ENSG00000087245	16	55423612-55540603
MMP9	ENSG00000100985	20	44637547-44645200
NDUFC2	ENSG00000151366	11	77779350-77791265
NPPB	ENSG00000120937	1	11917521-11918988
NR1I2	ENSG00000144852	3	119499331-119537332
NR3C2	ENSG00000151623	4	148999913-149365850
PDE1A	ENSG00000115252	2	183004763-183387919
PDE1B	ENSG00000123360	12	54943134-54973023
PLAU	ENSG00000122861	10	75668935-75677255
PPARG	ENSG00000132170	3	12328867-12475855
REN	ENSG00000143839	1	204123944-204135465
SCNN1A	ENSG00000111319	12	6456009-6486896
SCNN1B	ENSG00000168447	16	23289552-23392620
SCNN1D	ENSG00000162572	1	1215816-1227409
SCNN1G	ENSG00000166828	16	23194036-23228204
SELE	ENSG00000007908	1	169691781-169733846
SFRP4	ENSG00000106483	7	37945543-38065297
SLC12A1	ENSG00000074803	15	48483861-48596275
SLC12A2	ENSG00000064651	5	127419458-127525380
SLC12A3	ENSG00000070915	16	56899119-56949762
SLC12A4	ENSG00000124067	16	67977377-68003504
SLC12A5	ENSG00000124140	20	44650356-44688784
SLC6A4	ENSG00000108576	17	28521337-28563020
SLC9A1	ENSG00000090020	1	27425306-27493472
SMPD1	ENSG00000166311	11	6411655-6416228
TNNC1	ENSG00000114854	3	52485118-52488086
TNNC2	ENSG00000101470	20	44451853-44462384
VCAM1	ENSG00000162692	1	101185298-101204601
VEGFA	ENSG00000112715	6	43737921-43754224

Table S12. Genetic variants as proxies for renin-angiotensin system (RAS) inhibitors used in drug-target Mendelian randomisation

gene	SNP	effect_allele	other_allele	beta.exposure	se.exposure	pval.exposure	beta.outcome	se.outcome	pval.outcome	beta	se	pval
ACE	rs8077276	G	A	0.072625799	0.008794493	1.48E-16	0.2313	0.0251	2.82E-20	3.184819	0.345607	3.11E-20
JUN	rs113299700	A	G	-0.23748999	0.04841103	9.30E-07	0.2884	0.1417	0.04184	-1.21437	0.596657	0.041822
JUN	rs12063372	A	G	-0.036646354	0.00827736	9.54E-06	0.1615	0.0258	3.86E-10	-4.40699	0.704026	3.86E-10
JUN	rs12740054	G	A	-0.046978258	0.010898563	1.63E-05	-0.0735	0.0363	0.04268	1.564554	0.772698	0.042889
JUN	rs147625684	A	G	0.130521397	0.029059645	7.08E-06	0.2314	0.1088	0.03348	1.772889	0.83358	0.033434
JUN	rs17356414	T	C	0.038785606	0.008492764	4.95E-06	-0.0809	0.0265	0.002221	-2.08583	0.683243	0.002267
JUN	rs2492321	A	G	0.120708866	0.009771149	4.66E-35	-0.0994	0.0301	0.0009687	-0.82347	0.24936	0.000959
JUN	rs2716131	T	G	-0.129802484	0.020435865	2.13E-10	0.205	0.0777	0.008304	-1.57932	0.598602	0.008331
JUN	rs2764933	T	C	-0.205268281	0.007992535	1.83E-145	0.1112	0.0243	4.85E-06	-0.54173	0.118382	4.74E-06
JUN	rs4912387	C	T	0.034453507	0.008068548	1.95E-05	-0.0873	0.0245	0.000373	-2.53385	0.711103	0.000366
JUN	rs67430108	A	G	0.08886386	0.014292539	5.05E-10	-0.3037	0.0421	5.27E-13	-3.41759	0.473758	5.44E-13
JUN	rs822165	G	A	-0.038814219	0.007978257	1.14E-06	0.0799	0.0245	0.001106	-2.05852	0.631212	0.001109
JUN	rs835369	A	G	-0.057594963	0.009597081	1.96E-09	-0.0655	0.0297	0.02736	1.137252	0.51567	0.027427
LTA4H	rs11108491	A	C	-0.036353245	0.008271877	1.11E-05	0.1198	0.0265	5.89E-06	-3.29544	0.728958	6.16E-06
LTA4H	rs11108574	A	G	0.055641635	0.011722913	2.07E-06	0.1015	0.0345	0.003243	1.824174	0.620039	0.003261
LTA4H	rs112882964	A	G	-0.165864521	0.030436091	5.05E-08	0.1949	0.0827	0.01843	-1.17506	0.4986	0.018438
LTA4H	rs116883244	G	A	-0.174761279	0.027764565	3.09E-10	0.2736	0.1068	0.01038	-1.56556	0.611119	0.010413
LTA4H	rs12812062	G	A	0.083156217	0.013715132	1.34E-09	-0.1008	0.0408	0.01349	-1.21218	0.490643	0.013489
LTA4H	rs12820402	A	G	0.061059823	0.009408148	8.58E-11	0.0651	0.0287	0.0232	1.066168	0.470031	0.023311
LTA4H	rs1362971	G	A	-0.054494308	0.008350466	6.75E-11	-0.0554	0.0258	0.03178	1.01662	0.473444	0.03177
LTA4H	rs139457745	T	C	-0.120717113	0.025892736	3.13E-06	-0.1956	0.0683	0.0042	1.620317	0.565786	0.004185
LTA4H	rs140590723	T	C	0.289331723	0.051578879	2.03E-08	-0.2871	0.1182	0.01509	-0.99229	0.408528	0.015143
LTA4H	rs1421436	A	G	0.136720028	0.015040045	9.89E-20	0.1346	0.0443	0.00239	0.984494	0.32402	0.002379
LTA4H	rs2230885	T	G	0.177965	0.010079177	9.05E-70	0.0712	0.0315	0.02392	0.400079	0.177001	0.023802
LTA4H	rs2660863	C	T	-0.191373407	0.035888121	9.68E-08	0.2848	0.1223	0.01994	-1.48819	0.639065	0.019875
LTA4H	rs34519397	G	A	-0.07753406	0.009148346	2.34E-17	0.0602	0.027	0.02582	-0.77643	0.348234	0.025772
LTA4H	rs34602	C	T	-0.057128399	0.009167239	4.61E-10	0.1112	0.0275	5.41E-05	-1.94649	0.481372	5.26E-05
LTA4H	rs61938768	T	C	0.243592367	0.014336215	9.51E-65	-0.0876	0.0433	0.04313	-0.35962	0.177756	0.043063
LTA4H	rs6538681	A	G	0.083660871	0.00894606	8.63E-21	-0.1049	0.0268	9.13E-05	-1.25387	0.320341	9.07E-05
LTA4H	rs73220065	G	A	0.084972395	0.009891898	8.70E-18	0.0862	0.0296	0.003626	1.014447	0.348348	0.003589

gene	SNP	effect_allele	other_allele	beta.exposure	se.exposure	pval.exposure	beta.outcome	se.outcome	pval.outcome	beta	se	pval
LTA4H	rs77138128	A	G	0.060586522	0.01189487	3.52E-07	-0.0764	0.0389	0.04974	-1.26101	0.642057	0.049529
MMP9	rs112728846	A	G	0.179474284	0.037416979	1.62E-06	0.2558	0.1229	0.0373	1.425274	0.684778	0.0374
MMP9	rs117650271	T	C	-0.148864981	0.029512109	4.55E-07	0.2259	0.0863	0.008891	-1.51748	0.57972	0.008855
MMP9	rs144373758	G	A	0.293391588	0.030446814	5.63E-22	-0.2371	0.0989	0.01645	-0.80813	0.337092	0.016513
MMP9	rs4812987	C	T	-0.203072032	0.026460965	1.66E-14	0.2277	0.0799	0.004394	-1.12128	0.393456	0.004375
MMP9	rs6074044	T	C	-0.074469379	0.01199069	5.28E-10	0.1395	0.0358	9.94E-05	-1.87325	0.480735	9.75E-05
MMP9	rs811657	T	C	0.036187185	0.008311632	1.34E-05	0.1029	0.0255	5.46E-05	2.843548	0.704669	5.45E-05
PPARG	rs114975640	T	C	0.183899956	0.02830188	8.15E-11	0.2441	0.0907	0.007109	1.327352	0.493203	0.007118
PPARG	rs115113943	A	G	-0.161768649	0.026376327	8.61E-10	-0.1781	0.0877	0.04236	1.100955	0.542132	0.042277
PPARG	rs11706443	G	T	-0.078324399	0.012314576	2.01E-10	-0.0822	0.0368	0.02541	1.049481	0.469841	0.025503
PPARG	rs139628827	G	A	0.273918608	0.039480349	3.98E-12	0.266	0.0956	0.005387	0.971091	0.349009	0.005395
PPARG	rs145809586	A	G	-0.176978001	0.031619024	2.18E-08	-0.2175	0.0885	0.01394	1.228966	0.500062	0.013986
PPARG	rs1797912	C	A	-0.04016815	0.008399337	1.73E-06	-0.1658	0.0254	6.16E-11	4.127648	0.632342	6.69E-11
PPARG	rs2305398	A	G	0.044198454	0.00814643	5.78E-08	0.1047	0.0247	2.26E-05	2.368861	0.558843	2.25E-05
PPARG	rs2600228	A	C	0.043429216	0.00816308	1.04E-07	0.0769	0.0246	0.001779	1.770697	0.566439	0.001772
PPARG	rs3729931	A	G	-0.115944433	0.008333892	5.31E-44	-0.1476	0.025	3.39E-09	1.273024	0.215621	3.55E-09
PPARG	rs4135250	G	A	-0.050490851	0.011546836	1.23E-05	-0.1802	0.0364	7.29E-07	3.568963	0.720923	7.40E-07
PPARG	rs438129	T	C	-0.044985151	0.009386965	1.65E-06	0.0618	0.0296	0.03658	-1.37379	0.657995	0.036812
PPARG	rs56133301	A	G	0.157297255	0.034547288	5.29E-06	-0.3356	0.1052	0.001419	-2.13354	0.668797	0.001422
PPARG	rs73025253	C	A	-0.10972576	0.022149816	7.27E-07	0.1441	0.0687	0.0361	-1.31327	0.626106	0.035947
PPARG	rs73029213	G	A	-0.117060374	0.012776309	5.08E-20	0.1182	0.037	0.00142	-1.00974	0.316076	0.0014
PPARG	rs74406844	A	T	-0.081823961	0.013133862	4.66E-10	0.1742	0.0407	1.83E-05	-2.12896	0.497409	1.87E-05

gene	SNP	effect_allele	other_allele	beta.exposure	se.exposure	pval.exposure	beta.outcome	se.outcome	pval.outcome	beta	se	pval
PPARG	rs79310821	A	G	0.133663196	0.023053328	6.72E-09	0.1688	0.079	0.03254	1.262876	0.591038	0.032622

Note: "beta.exposure" is obtained from eQTLGen; "beta.outcome" is obtained from the systolic blood pressure (SBP) GWAS; "beta" represnet the association between gene expression and SBP, calculated using two-sample Mendelian randomisation Wald method.

Table S13. Genetic variants proxies for beta blockers (BBs) used in drug-target Mendelian randomisation

gene	SNP	effect_allele	other_allele	beta.exposure	se.exposure	pval.exposure	beta.outcome	se.outcome	pval.outcome	beta	se	pval
ADRA2A	rs10885091	C T		-0.0397568	0.008006122	6.83E-07	0.0669	0.0246	0.006612	-1.68273	0.618762	0.006538
ADRB1	rs11196549	A G		-0.095301105	0.021794069	1.23E-05	0.5354	0.0609	1.50E-18	-5.61798	0.639027	1.48E-18
ADRB1	rs117648943	G A		0.513462833	0.078349406	5.62E-11	-0.2385	0.1166	0.04074	-0.46449	0.227086	0.04081
ADRB1	rs143854972	A G		0.208491523	0.019438319	7.70E-27	0.3536	0.0537	4.62E-11	1.695992	0.257564	4.56E-11
ADRB1	rs17875473	T C		-0.092587193	0.014915375	5.38E-10	0.2699	0.0442	1.06E-09	-2.91509	0.477388	1.02E-09
ADRB1	rs1801253	G C		-0.130481564	0.009894036	1.03E-39	-0.399	0.0278	9.76E-47	3.057903	0.213057	1.03E-46
ADRB1	rs2532825	A G		-0.054328863	0.008940962	1.23E-09	0.1416	0.0254	2.57E-08	-2.60635	0.467523	2.48E-08
ADRB1	rs572208	G A		0.157209523	0.011906295	8.34E-40	-0.1958	0.0342	1.05E-08	-1.24547	0.217544	1.03E-08
ADRB1	rs75228369	T C		0.097881297	0.022565253	1.44E-05	0.261	0.0614	2.16E-05	2.666495	0.62729	2.13E-05
ADRB1	rs79352661	C T		-0.12087472	0.022118377	4.63E-08	-0.235	0.0781	0.002618	1.944162	0.646124	0.002621
ADRB2	rs11959615	T A		0.12623957	0.008028413	1.04E-55	-0.0795	0.0242	0.001023	-0.62975	0.191699	0.001019
ADRB2	rs12522527	A T		0.036801682	0.007959702	3.78E-06	-0.1047	0.0245	1.96E-05	-2.84498	0.66573	1.92E-05
ADRB2	rs34246418	A C		0.036838509	0.008391651	1.13E-05	0.1585	0.0256	6.44E-10	4.302563	0.694925	5.96E-10
ADRB2	rs4705067	C G		-0.040821387	0.009533475	1.85E-05	-0.124	0.03	3.64E-05	3.037623	0.734909	3.58E-05
HIF1A	rs11158352	A G		0.109050598	0.013810516	2.88E-15	-0.0975	0.0416	0.01906	-0.89408	0.381474	0.019091
HIF1A	rs3751293	C T		-0.058945299	0.01292065	5.06E-06	0.0838	0.0388	0.03087	-1.42166	0.658237	0.030789
HIF1A	rs710047	C G		-0.078583176	0.009930268	2.50E-15	0.097	0.0359	0.006812	-1.23436	0.456841	0.006893
HIF1A	rs710067	A G		0.046504131	0.010557843	1.06E-05	0.0874	0.0332	0.008396	1.879403	0.713915	0.008475
HIF1A	rs7159288	G A		-0.078889542	0.017545045	6.91E-06	0.1669	0.0643	0.009511	-2.11562	0.815064	0.009441
HIF1A	rs72716794	A G		-0.17679714	0.037787663	2.89E-06	0.2824	0.1124	0.01198	-1.59731	0.635757	0.011989
KCNH2	rs11546674	A C		0.052826341	0.008056972	5.51E-11	0.0956	0.0249	0.0001196	1.809703	0.471356	0.000123
KCNH2	rs116972354	A C		0.125247004	0.022100332	1.45E-08	-0.2096	0.059	0.0003844	-1.67349	0.471069	0.000382
KCNH2	rs192090795	C A		-0.188498482	0.040345558	2.98E-06	0.3438	0.1106	0.001877	-1.82389	0.586742	0.00188
KCNH2	rs2303923	G C		0.045776417	0.009899744	3.76E-06	0.0718	0.025	0.004111	1.568493	0.546133	0.004079
KCNH2	rs2566514	G C		0.042687173	0.008908008	1.65E-06	-0.2309	0.0286	6.71E-16	-5.40912	0.66999	6.84E-16
KCNH2	rs35274907	T C		-0.123300581	0.027799202	9.18E-06	-0.2592	0.0808	0.001329	2.10218	0.655309	0.001337
KCNH2	rs3807375	T C		-0.063882854	0.008245077	9.32E-15	-0.1033	0.0251	3.71E-05	1.617022	0.392907	3.86E-05
KCNH2	rs3918226	T C		0.086486986	0.015936133	5.73E-08	0.5655	0.0467	8.94E-34	6.538556	0.539966	9.44E-34

gene	SNP	effect_allele	other_allele	beta.exposure	se.exposure	pval.exposure	beta.outcome	se.outcome	pval.outcome	beta	se	pval
KCNH2	rs73169605	T C		0.153254444	0.022086592	3.95E-12	-0.1616	0.0723	0.02536	-1.05446	0.471764	0.025409
KCNH2	rs740954	C T		0.034255991	0.008031132	2.00E-05	-0.1452	0.0247	4.10E-09	-4.23867	0.721042	4.14E-09
KCNH2	rs79684285	T C		-0.169055252	0.026292868	1.28E-10	-0.2228	0.0734	0.002398	1.317912	0.434178	0.002402
KCNJ2	rs4435300	C T		0.09789962	0.008287167	3.33E-32	0.0908	0.0255	0.0003806	0.927481	0.260471	0.00037
NDUFC2	rs116896993	A C		-0.306548916	0.026065755	6.21E-32	-0.1868	0.0682	0.006204	0.609364	0.222477	0.006163
NDUFC2	rs11821643	T A		-0.055446767	0.011567797	1.64E-06	-0.0971	0.0344	0.004804	1.751229	0.620415	0.004762
NDUFC2	rs1986277	C T		-0.056329913	0.013147678	1.83E-05	0.1305	0.0402	0.001162	-2.31671	0.713653	0.001169
NDUFC2	rs4945241	T C		-0.216870919	0.009615886	1.24E-112	-0.0896	0.0295	0.002405	0.413149	0.136026	0.002387
NDUFC2	rs530588	C T		-0.080548075	0.011276031	9.11E-13	0.0864	0.0336	0.01011	-1.07265	0.417142	0.010128
NDUFC2	rs7119933	C A		-0.069600132	0.010353157	1.78E-11	-0.1639	0.0316	2.18E-07	2.354881	0.454022	2.14E-07
NDUFC2	rs76498871	A G		0.140138823	0.021316159	4.89E-11	0.1378	0.0615	0.02505	0.983311	0.438851	0.025049
NDUFC2	rs901099	T G		-0.091936506	0.008825114	2.06E-25	-0.0564	0.0269	0.03579	0.613467	0.292593	0.036024
VCAM1	rs10875321	A G		0.162698934	0.034818296	2.97E-06	0.2831	0.1133	0.01245	1.740024	0.696378	0.012466
VCAM1	rs11166440	G A		-0.072242749	0.008322418	3.94E-18	-0.0736	0.0252	0.003505	1.018787	0.348824	0.003493
VCAM1	rs11166507	C T		-0.042545655	0.009102427	2.95E-06	-0.0731	0.0279	0.008876	1.718154	0.655766	0.008791
VCAM1	rs4488055	T C		-0.227252634	0.034007637	2.35E-11	-0.3176	0.124	0.01041	1.397564	0.545648	0.010428
VCAM1	rs721385	G A		0.067452316	0.014475678	3.17E-06	0.1053	0.0433	0.01507	1.561103	0.641935	0.015021
VCAM1	rs72732105	C A		0.139538659	0.026344452	1.18E-07	-0.1693	0.0718	0.01845	-1.21328	0.514553	0.018377
VCAM1	rs77639769	G A		-0.140371804	0.021808717	1.22E-10	0.1211	0.0564	0.0317	-0.86271	0.40179	0.03178
VEGFA	rs114681547	C A		-0.049536038	0.01081691	4.66E-06	0.1185	0.0321	0.0002266	-2.3922	0.648013	0.000223
VEGFA	rs11755799	A G		-0.110636061	0.009112449	6.39E-34	0.0657	0.0291	0.02425	-0.59384	0.263025	0.023962
VEGFA	rs2270860	T C		-0.039962518	0.008631213	3.66E-06	0.3106	0.0261	1.04E-32	-7.77228	0.653112	1.18E-32
VEGFA	rs3025053	A G		-0.078905282	0.014545021	5.80E-08	-0.2112	0.0421	5.42E-07	2.676627	0.533551	5.26E-07
VEGFA	rs35410204	C T		-0.04423937	0.008859214	5.93E-07	-0.0721	0.0273	0.008118	1.62977	0.617097	0.008266
VEGFA	rs41281826	T G		0.234978307	0.035657881	4.40E-11	-0.2586	0.094	0.005932	-1.10053	0.400037	0.00594
VEGFA	rs6899540	C A		0.071668559	0.01195951	2.07E-09	-0.0828	0.0337	0.01396	-1.15532	0.47022	0.014011

gene	SNP	effect_allele	other_allele	beta.exposure	se.exposure	pval.exposure	beta.outcome	se.outcome	pval.outcome	beta	se	pval
VEGFA	rs76074477	A G		-0.159592491	0.02089071	2.18E-14	-0.3022	0.0693	1.30E-05	1.893573	0.434231	1.30E-05
VEGFA	rs79654162	T C		0.076714062	0.012640105	1.29E-09	0.1026	0.0368	0.005261	1.337434	0.479703	0.005303
VEGFA	rs9394961	C T		-0.115526744	0.008107083	4.47E-46	-0.1006	0.025	5.77E-05	0.870794	0.2164	5.72E-05

Note: "beta.exposure" is obtained from eQTLGen; "beta.outcome" is obtained from the systolic blood pressure (SBP) GWAS; "beta" represnet the association between gene expression and SBP, calculated using two-sample Mendelian randomisation Wald method.

Table S14. Genetic variants proxies for calcium channel blockers (CCBs) used in drug-target Mendelian randomisation

gene	SNP	effect_allele	other_allele	beta.exposure	se.exposure	pval.exposure	beta.outcome	se.outcome	pval.outcome	beta	se	pval
ABCB1	rs10235853	G	T	0.048037785	0.00906801	1.17E-07	0.0859	0.0277	0.001907	1.788176	0.576629	0.001928
ABCB1	rs10275831	T	C	0.118280011	0.024011858	8.40E-07	0.2066	0.0763	0.006759	1.746703	0.645079	0.006774
ABCB1	rs10808071	G	A	-0.094925537	0.01048565	1.39E-19	-0.1188	0.0306	0.0001044	1.251507	0.322358	0.000103
AHR	rs112198319	G	A	-0.179924082	0.034058469	1.27E-07	-0.2848	0.098	0.003653	1.58289	0.544674	0.003659
AHR	rs112780228	G	T	0.336881446	0.028804365	1.35E-31	-0.2299	0.0985	0.01956	-0.68244	0.292388	0.019595
AHR	rs12699826	G	A	0.087693579	0.013780715	1.97E-10	0.1165	0.0415	0.005011	1.32849	0.473239	0.004997
AHR	rs1404960	G	A	0.097367239	0.008679477	3.33E-29	-0.0846	0.0273	0.001915	-0.86888	0.280382	0.001942
AHR	rs144923433	A	G	0.182828966	0.03405651	7.94E-08	-0.2277	0.0994	0.02191	-1.24543	0.543678	0.021978
AHR	rs17704807	C	T	0.088811051	0.008137202	9.86E-28	-0.0611	0.0251	0.01511	-0.68798	0.282622	0.014922
AHR	rs55824063	C	T	0.049932006	0.009285184	7.56E-08	-0.1008	0.0283	0.0003729	-2.01875	0.566771	0.000368
AHR	rs6968865	A	T	0.049706111	0.008274974	1.89E-09	-0.0955	0.0248	0.0001174	-1.92129	0.498933	0.000118
AHR	rs73073176	T	C	0.055384579	0.012598858	1.10E-05	-0.1291	0.0371	0.0005084	-2.33097	0.669862	0.000502
AHR	rs76240850	A	G	0.105441085	0.016856279	3.97E-10	0.104	0.0524	0.04704	0.986333	0.49696	0.047174
AHR	rs9648222	A	G	-0.060012065	0.013101069	4.63E-06	-0.0943	0.0432	0.02906	1.571351	0.719855	0.029045
CACNA1C	rs10774053	A	G	-0.051099977	0.011833903	1.57E-05	-0.104	0.0316	0.001012	2.035226	0.618396	0.000998
CACNA1D	rs312476	T	G	-0.045357712	0.009086262	5.98E-07	0.1042	0.0282	0.0002206	-2.29729	0.621724	0.00022

gene	SNP	effect_allele	other_allele	beta.exposure	se.exposure	pval.exposure	beta.outcome	se.outcome	pval.outcome	beta	se	pval
CACNA1D	rs898419	A	C	0.050246662	0.01081853	3.41E-06	-0.0664	0.0317	0.03625	-1.32148	0.630888	0.036203
CACNA1D	rs9841978	A	G	-0.06373765	0.008449571	4.58E-14	0.2072	0.0261	1.95E-15	-3.25083	0.409491	2.04E-15
CACNA1H	rs117177120	G	T	0.371975144	0.024021953	4.41E-54	0.2868	0.0645	8.68E-06	0.771019	0.173399	8.73E-06
CACNA1H	rs140532679	G	A	0.266094687	0.051077758	1.89E-07	0.2568	0.1185	0.03019	0.96507	0.44533	0.030228
CACNA1H	rs2738896	C	T	-0.062606782	0.012816127	1.03E-06	-0.0983	0.0312	0.001626	1.570117	0.498349	0.001629
CACNA1H	rs3751661	A	T	0.10540603	0.010079371	1.35E-25	0.0836	0.0255	0.00106	0.793124	0.241922	0.001044
CACNA1I	rs136829	C	T	-0.130347329	0.010438559	8.77E-36	0.0808	0.0304	0.007806	-0.61988	0.233223	0.007863
CACNA1I	rs73169382	A	C	-0.039331356	0.008745354	6.87E-06	-0.0599	0.0265	0.02375	1.522958	0.673763	0.023798
CACNA2D2rs2526388		T	C	-0.043156611	0.00934328	3.86E-06	0.0815	0.0276	0.003166	-1.88847	0.639531	0.003148
CACNA2D2rs6763990		C	T	0.052318665	0.011773937	8.85E-06	0.1667	0.0358	3.26E-06	3.186243	0.684268	3.22E-06
CACNA2D3rs116147398		T	C	0.178709156	0.037252028	1.61E-06	0.2732	0.1157	0.01824	1.528741	0.647421	0.018212
CACNA2D3rs1534236		T	C	0.074199306	0.008022674	2.27E-20	0.0527	0.0246	0.0322	0.710249	0.331539	0.032171
CACNA2D3rs4077115		A	G	-0.110755788	0.014927058	1.17E-13	0.1035	0.0436	0.0177	-0.93449	0.393659	0.017604
CACNA2D3rs4955921		G	A	0.090629447	0.008255101	4.84E-28	-0.0717	0.0252	0.004447	-0.79113	0.278055	0.004438
CACNA2D3rs59719061		C	T	-0.038305816	0.008192531	2.93E-06	0.0608	0.025	0.01491	-1.58723	0.652642	0.015016
CACNA2D3rs62254535		G	C	-0.055108992	0.012080272	5.07E-06	-0.1223	0.0355	0.0005677	2.219239	0.644178	0.000571
CACNA2D3rs62254541		A	C	-0.130854808	0.02485702	1.41E-07	-0.164	0.0677	0.01547	1.253297	0.517367	0.015416
CACNB2	rs10829260	G	A	0.060328514	0.010082311	2.19E-09	0.077	0.031	0.01286	1.276345	0.513853	0.012996
CACNB2	rs11014484	C	G	0.046246041	0.008272107	2.26E-08	0.1103	0.025	1.05E-05	2.385069	0.540587	1.02E-05

gene	SNP	effect_allele	other_allele	beta.exposure	se.exposure	pval.exposure	beta.outcome	se.outcome	pval.outcome	beta	se	pval
CACNB2	rs111646368	G	A	0.098253381	0.021389655	4.36E-06	0.2197	0.0651	0.0007348	2.236055	0.662573	0.000739
CACNB2	rs117539117	T	C	-0.079186544	0.015020779	1.35E-07	-0.1168	0.0438	0.007698	1.474998	0.553124	0.007661
CACNB2	rs12219393	G	A	-0.062843704	0.013515356	3.32E-06	0.1369	0.0415	0.0009587	-2.17842	0.660368	0.000971
CACNB2	rs12258967	G	C	-0.047065455	0.008968607	1.54E-07	-0.5456	0.027	1.43E-90	11.59237	0.573669	8.43E-91
CACNB2	rs61839375	A	G	0.108990259	0.011472055	2.09E-21	0.1076	0.0368	0.003445	0.987244	0.337645	0.003457
CACNB2	rs7100884	A	G	-0.110975497	0.010120331	5.59E-28	-0.1708	0.0297	9.16E-09	1.539078	0.267627	8.88E-09
CACNB3	rs10875907	A	G	0.318758856	0.038874453	2.41E-16	-0.407	0.0963	2.40E-05	-1.27683	0.302109	2.37E-05
CACNB3	rs141479808	G	C	-0.19256038	0.044520572	1.52E-05	0.1885	0.0833	0.0236	-0.97891	0.432592	0.023642
CACNB3	rs146336654	A	G	0.3509968	0.023856885	5.37E-49	0.6034	0.0686	1.36E-18	1.719104	0.195443	1.42E-18
CACNB3	rs151022896	G	A	0.224133188	0.050467944	8.95E-06	0.4732	0.1354	0.0004738	2.111245	0.604105	0.000474
CACNB3	rs1726440	T	C	-0.056303284	0.012271323	4.47E-06	-0.0881	0.0418	0.03519	1.56474	0.742408	0.035061
CACNB3	rs2453473	C	G	-0.071748462	0.012389222	6.99E-09	-0.1506	0.0384	8.74E-05	2.099	0.535203	8.79E-05
CACNB3	rs78957863	C	A	0.255840293	0.042449028	1.67E-09	-0.2311	0.1003	0.02118	-0.9033	0.392041	0.021218
CACNB3	rs9668292	C	A	0.332396477	0.010069874	1.00E-200	0.0795	0.0304	0.008972	0.239172	0.091457	0.008919
CACNB3	rs9739878	A	G	0.103641738	0.008311286	1.09E-35	0.1012	0.0253	6.53E-05	0.976441	0.24411	6.33E-05
CACNB4	rs12622797	A	G	0.055850504	0.012275645	5.37E-06	-0.108	0.0394	0.00606	-1.93373	0.705455	0.006123
CACNB4	rs16830878	A	G	0.156663371	0.015318455	1.50E-24	0.1722	0.0466	0.0002172	1.099172	0.297453	0.00022
CACNB4	rs2139419	C	T	0.058393865	0.008229356	1.29E-12	-0.1114	0.0252	9.60E-06	-1.90773	0.431552	9.84E-06
CACNB4	rs35172004	A	T	0.077479793	0.01455976	1.03E-07	0.1455	0.0438	0.0008918	1.877909	0.565309	0.000894

gene	SNP	effect_allele	other_allele	beta.exposure	se.exposure	pval.exposure	beta.outcome	se.outcome	pval.outcome	beta	se	pval
					6							
CACNB4	rs4507102	T	A	0.040904869	0.008816845	3.49E-06	-0.0906	0.0273	0.0009096	-2.2149	0.667402	0.000904
CACNB4	rs6760139	C	G	-0.038751089	0.008422685	4.21E-06	0.0752	0.0252	0.00284	-1.94059	0.650304	0.002844
CALM1	rs61986783	C	T	0.120780603	0.015571333	8.74E-15	0.0931	0.0468	0.0466	0.770819	0.387479	0.046666
CALM1	rs74926439	T	C	0.163200646	0.03640109	7.35E-06	-0.31	0.105	0.003141	-1.8995	0.64338	0.003153
KCNH2	rs11546674	A	C	0.052826341	0.008056972	5.51E-11	0.0956	0.0249	0.0001196	1.809703	0.471356	0.000123
KCNH2	rs116972354	A	C	0.125247004	0.022100332	1.45E-08	-0.2096	0.059	0.0003844	-1.67349	0.471069	0.000382
KCNH2	rs192090795	C	A	-0.188498482	0.040345558	2.98E-06	0.3438	0.1106	0.001877	-1.82389	0.586742	0.00188
KCNH2	rs2303923	G	C	0.045776417	0.009899744	3.76E-06	0.0718	0.025	0.004111	1.568493	0.546133	0.004079
KCNH2	rs2566514	G	C	0.042687173	0.008908008	1.65E-06	-0.2309	0.0286	6.71E-16	-5.40912	0.66999	6.84E-16
KCNH2	rs35274907	T	C	-0.123300581	0.027799202	9.18E-06	-0.2592	0.0808	0.001329	2.10218	0.655309	0.001337
KCNH2	rs3807375	T	C	-0.063882854	0.008245077	9.32E-15	-0.1033	0.0251	3.71E-05	1.617022	0.392907	3.86E-05
KCNH2	rs3918226	T	C	0.086486986	0.015936133	5.73E-08	0.5655	0.0467	8.94E-34	6.538556	0.539966	9.44E-34
KCNH2	rs73169605	T	C	0.153254444	0.022086592	3.95E-12	-0.1616	0.0723	0.02536	-1.05446	0.471764	0.025409
KCNH2	rs740954	C	T	0.034255991	0.008031132	2.00E-05	-0.1452	0.0247	4.10E-09	-4.23867	0.721042	4.14E-09
KCNH2	rs79684285	T	C	-0.169055252	0.026292868	1.28E-10	-0.2228	0.0734	0.002398	1.317912	0.434178	0.002402
KCNJ11	rs10832696	C	T	0.097109716	0.022543287	1.65E-05	0.1158	0.0365	0.001491	1.192466	0.375864	0.001511
KCNJ11	rs10832778	C	G	0.136972998	0.012115286	1.23E-29	0.3181	0.0249	2.79E-37	2.322356	0.181788	2.26E-37
NR3C2	rs13109093	C	A	0.055039656	0.008214384	2.08E-11	-0.0576	0.0248	0.0203	-1.04652	0.450584	0.020202
PDE1B	rs11199580	T	C	0.307691336	0.03741246	1.97E-16	0.2718	0.094	0.003837	0.883353	0.305501	0.003834

gene	SNP	effect_allele	other_allele	beta.exposure	se.exposure	pval.exposure	beta.outcome	se.outcome	pval.outcome	beta	se	pval
	9				5							
PDE1B	rs113935627	A	C	-0.075018449	0.017569135	1.95E-05	-0.1412	0.0568	0.01286	1.882204	0.757147	0.012922
PDE1B	rs11611943	G	A	-0.051904575	0.010807824	1.57E-06	0.2117	0.0309	7.71E-12	-4.07864	0.595323	7.33E-12
PDE1B	rs138448085	G	A	0.257653363	0.044475137	6.91E-09	-0.4276	0.1284	0.0008677	-1.65959	0.498344	0.000868
PDE1B	rs139476284	A	G	-0.178575348	0.025467469	2.35E-12	0.2159	0.0796	0.006663	-1.20901	0.44575	0.006682
PDE1B	rs149643204	T	C	-0.274670982	0.026096014	6.58E-26	-0.1855	0.0923	0.04458	0.675353	0.336038	0.044458
PDE1B	rs35852203	T	C	-0.159878693	0.013937764	1.85E-30	0.1279	0.0397	0.001277	-0.79998	0.248313	0.001274
PDE1B	rs3782403	C	T	0.196082519	0.007924895	3.74E-135	-0.0951	0.0249	0.0001388	-0.485	0.126987	0.000134
PDE1B	rs4326844	A	G	0.064644506	0.008078947	1.23E-15	0.0905	0.0249	0.0002844	1.399964	0.385184	0.000278
PDE1B	rs73115303	G	A	0.099386984	0.021959607	6.02E-06	0.2428	0.0656	0.0002122	2.442976	0.660046	0.000215
PDE1B	rs74091317	A	G	0.221553543	0.04387894	4.44E-07	-0.223	0.1023	0.02925	-1.00653	0.461739	0.029268
SMPD1	rs1079199	C	T	0.056239837	0.008843298	2.02E-10	0.0733	0.0263	0.005387	1.303347	0.46764	0.005319
TNNC1	rs353547	T	C	0.045862969	0.008072193	1.33E-08	0.0935	0.0249	0.0001767	2.038682	0.542922	0.000173
TNNC1	rs55664484	C	T	0.088966557	0.019034758	2.96E-06	0.1387	0.0566	0.01423	1.559013	0.636194	0.014265
TNNC2	rs116841822	G	A	-0.099850126	0.021858609	4.92E-06	-0.1945	0.0649	0.002742	1.947919	0.649974	0.002727
TNNC2	rs145371887	T	C	-0.131836393	0.029130608	6.02E-06	-0.3137	0.0963	0.001123	2.379464	0.730451	0.001124
TNNC2	rs146153465	A	C	0.36260519	0.028045013	3.08E-38	0.2671	0.0944	0.004637	0.736614	0.260338	0.004663
TNNC2	rs2425780	G	A	0.120505113	0.014207158	2.21E-17	-0.0926	0.0394	0.01883	-0.76843	0.326957	0.018761
TNNC2	rs55943410	T	C	-0.156606058	0.017472115	3.15E-19	-0.1997	0.0482	3.38E-05	1.275174	0.307779	3.43E-05
TNNC2	rs57041960	C	A	0.074323785	0.01231239	1.58E-09	0.091	0.037	0.01387	1.224373	0.497822	0.013915

gene	SNP	effect_allele	other_allele	beta.exposure	se.exposure	pval.exposure	beta.outcome	se.outcome	pval.outcome	beta	se	pval
					7							
TNNC2	rs6017746	C	T	-0.050868364	0.009485235	8.19E-08	0.0914	0.0284	0.001289	-1.79679	0.558304	0.001289
TNNC2	rs6065826	T	C	-0.08795058	0.020518998	1.82E-05	0.1353	0.063	0.03167	-1.53836	0.716311	0.031744
TNNC2	rs6073949	G	A	0.065091566	0.008296675	4.32E-15	0.0711	0.0265	0.007399	1.092307	0.407119	0.007296
TNNC2	rs6130942	C	T	0.274136492	0.007833993	1.00E-200	0.131	0.0243	6.70E-08	0.477864	0.088642	7.01E-08
TNNC2	rs73116741	A	G	0.112088366	0.02034124	3.58E-08	0.1393	0.058	0.01623	1.242769	0.517449	0.016318

Note: "beta.exposure" is obtained from eQTLGen; "beta.outcome" is obtained from the systolic blood pressure (SBP) GWAS; "beta" represnet the association between gene expression and SBP, calculated using two-sample Mendelian randomisation Wald method.

Table S15. Genetic variants proxies for diuretics used in drug-target Mendelian randomisation

gene	SNP	effect_allele	other_allele	beta.exposure	se.exposure	pval.exposure	beta.outcome	se.outcome	pval.outcome	beta	se	pval
ABP1	rs10280499	G	C	0.411704973	0.019187	3.89E-102	0.175	0.0484	0.0003007	0.425062	0.11756	0.0003
ABP1	rs111651915	G	A	-0.091935589	0.020903	1.09E-05	-0.1974	0.0556	0.0003851	2.147155	0.604771	0.000385
ABP1	rs116972354	A	C	-0.144162535	0.02473619	5.61E-09	-0.2096	0.059	0.0003844	1.453914	0.40926	0.000382
ABP1	rs117588967	C	A	-0.13614534	0.02731045	6.19E-07	-0.2622	0.075	0.0004686	1.925883	0.550882	0.000472
ABP1	rs11762140	C	A	0.074507182	0.0088642	4.27E-17	-0.0846	0.0247	0.0006145	-1.13546	0.331512	0.000615
ABP1	rs11773687	A	G	0.082939816	0.01207996	6.61E-12	0.0848	0.0303	0.00508	1.022428	0.365325	0.005131
ABP1	rs140877522	A	G	0.156588228	0.02342067	2.30E-11	-0.1671	0.0713	0.0191	-1.06713	0.455334	0.019098
ABP1	rs17173658	T	G	0.086565852	0.01908418	5.73E-06	0.1786	0.0577	0.001967	2.063169	0.666545	0.001966
ABP1	rs17256042	A	G	-0.239590564	0.02247672	1.57E-26	-0.1674	0.0558	0.002681	0.698692	0.232897	0.0027
ABP1	rs17705456	A	C	-0.062250727	0.01371433	5.65E-06	-0.0828	0.0387	0.03263	1.330105	0.621679	0.032392
ABP1	rs1960024	A	G	0.17280312	0.00886279	1.15E-84	0.1567	0.0248	2.67E-10	0.906812	0.143516	2.64E-10
ABP1	rs2268998	T	C	0.084766949	0.00999445	2.23E-17	0.0651	0.0279	0.0196	0.767988	0.329138	0.019631
ABP1	rs2373962	G	C	-0.039905097	0.00904098	1.01E-05	0.1019	0.025	4.52E-05	-2.55356	0.626486	4.58E-05
ABP1	rs34723411	A	G	-0.164014448	0.02728118	1.83E-09	-0.3415	0.0762	7.50E-06	2.082134	0.464593	7.41E-06
ABP1	rs3800784	C	T	-0.03988143	0.00881864	6.11E-06	-0.0785	0.0244	0.001321	1.968335	0.611814	0.001294
ABP1	rs4725978	A	G	-0.043047558	0.00891365	1.37E-06	0.1084	0.0254	1.95E-05	-2.51815	0.590045	1.97E-05
ABP1	rs4725995	T	C	0.043556823	0.00945839	4.12E-06	0.1627	0.0267	1.19E-09	3.735351	0.612992	1.10E-09
ABP1	rs61649854	G	C	-0.247172585	0.02305757	8.22E-27	0.1589	0.0665	0.01686	-0.64287	0.269043	0.016873
ABP1	rs71537960	G	A	0.168969439	0.03714757	5.40E-06	-0.3142	0.0987	0.001465	-1.85951	0.584129	0.001456
ABP1	rs73163595	T	C	0.389088286	0.05180867	5.91E-14	0.2371	0.0966	0.01409	0.609373	0.248273	0.01411
ABP1	rs741066	T	C	0.079210487	0.00931921	1.90E-17	0.1602	0.0268	2.19E-09	2.022459	0.338339	2.26E-09
ABP1	rs76582590	G	A	-0.206664656	0.03836929	7.20E-08	-0.3055	0.0961	0.001479	1.47824	0.465005	0.001478
ABP1	rs76842805	T	C	-0.112759997	0.02400221	2.63E-06	0.1349	0.0659	0.0407	-1.19635	0.584427	0.040654
ABP1	rs7789585	A	G	-0.120826988	0.0114228	3.78E-26	-0.1235	0.0319	0.0001064	1.022123	0.264014	0.000108
ABP1	rs78430825	A	C	-0.14679632	0.02228982	4.52E-11	0.1717	0.0686	0.01227	-1.16965	0.467314	0.012317
ABP1	rs78533049	A	G	0.429794923	0.04467862	6.61E-22	0.3934	0.1117	0.0004303	0.91532	0.259891	0.000428
ABP1	rs79684285	T	C	-0.12803745	0.02899596	1.01E-05	-0.2228	0.0734	0.002398	1.740116	0.57327	0.002402
ABP1	rs891511	A	G	-0.043325369	0.00977646	9.35E-06	-0.2927	0.0267	4.98E-28	6.755857	0.616267	5.78E-28
ASIC1	rs11169270	A	G	-0.106779326	0.00940323	6.96E-30	-0.1276	0.0255	5.52E-07	1.194988	0.23881	5.62E-07
ASIC1	rs142203208	T	C	0.19403845	0.04479085	1.48E-05	-0.2126	0.0885	0.01631	-1.09566	0.456095	0.016294
ASIC1	rs427758	C	G	0.04935689	0.01146315	1.67E-05	-0.0938	0.0314	0.002818	-1.90044	0.636183	0.002815
ASIC1	rs73106145	C	G	0.044821838	0.00995952	6.78E-06	-0.2985	0.0277	5.10E-27	-6.6597	0.618002	4.46E-27

gene	SNP	effect_allele	other_allele	beta.exposure	se.exposure	pval.exposure	beta.outcome	se.outcome	pval.outcome	beta	se	pval
ATP1A1	rs6696907	C	G	0.040651768	0.00892053	5.19E-06	0.164	0.0279	3.96E-09	4.034265	0.686317	4.15E-09
CA2	rs11775810	T	G	0.260711127	0.01573269	1.13E-61	0.1006	0.0472	0.03292	0.385868	0.181043	0.03306
CA2	rs1534041	T	C	0.039653718	0.00897874	1.00E-05	-0.0726	0.0271	0.007309	-1.83085	0.683416	0.007385
CA2	rs188354953	A	G	0.317937555	0.03864375	1.92E-16	0.223	0.106	0.03531	0.701396	0.333399	0.035398
CA2	rs77062373	A	C	-0.516666752	0.02404846	2.18E-102	0.2584	0.0917	0.004853	-0.50013	0.177484	0.004834
CA4	rs1148559	T	C	0.048193637	0.00993642	1.23E-06	0.23	0.0306	5.53E-14	4.772414	0.634939	5.63E-14
CA4	rs1197070	A	T	-0.087827171	0.01095703	1.10E-15	0.1834	0.0351	1.78E-07	-2.08819	0.399649	1.74E-07
CA4	rs62084509	A	G	-0.094245387	0.01836784	2.88E-07	0.1663	0.0592	0.004957	-1.76454	0.628147	0.004968
CACNA1Crs10774053		A	G	-0.051099977	0.0118339	1.57E-05	-0.104	0.0316	0.001012	2.035226	0.618396	0.000998
CACNA1Drs312476		T	G	-0.045357712	0.00908626	5.98E-07	0.1042	0.0282	0.0002206	-2.29729	0.621724	0.00022
CACNA1Drs898419		A	C	0.050246662	0.01081853	3.41E-06	-0.0664	0.0317	0.03625	-1.32148	0.630888	0.036203
CACNA1Drs9841978		A	G	-0.06373765	0.00844957	4.58E-14	0.2072	0.0261	1.95E-15	-3.25083	0.409491	2.04E-15
CACNB2	rs10829260	G	A	0.060328514	0.01008231	2.19E-09	0.077	0.031	0.01286	1.276345	0.513853	0.012996
CACNB2	rs11014484	C	G	0.046246041	0.00827211	2.26E-08	0.1103	0.025	1.05E-05	2.385069	0.540587	1.02E-05
CACNB2	rs111646368	G	A	0.098253381	0.02138966	4.36E-06	0.2197	0.0651	0.0007348	2.236055	0.662573	0.000739
CACNB2	rs117539117	T	C	-0.079186544	0.01502078	1.35E-07	-0.1168	0.0438	0.007698	1.474998	0.553124	0.007661
CACNB2	rs12219393	G	A	-0.062843704	0.01351536	3.32E-06	0.1369	0.0415	0.0009587	-2.17842	0.660368	0.000971
CACNB2	rs12258967	G	C	-0.047065455	0.00896861	1.54E-07	-0.5456	0.027	1.43E-90	11.59237	0.573669	8.43E-91
CACNB2	rs61839375	A	G	0.108990259	0.01147206	2.09E-21	0.1076	0.0368	0.003445	0.987244	0.337645	0.003457
CACNB2	rs7100884	A	G	-0.110975497	0.01012033	5.59E-28	-0.1708	0.0297	9.16E-09	1.539078	0.267627	8.88E-09
CACNB3	rs10875907	A	G	0.318758856	0.03887445	2.41E-16	-0.407	0.0963	2.40E-05	-1.27683	0.302109	2.37E-05
CACNB3	rs141479808	G	C	-0.19256038	0.04452057	1.52E-05	0.1885	0.0833	0.0236	-0.97891	0.432592	0.023642
CACNB3	rs146336654	A	G	0.3509968	0.02385688	5.37E-49	0.6034	0.0686	1.36E-18	1.719104	0.195443	1.42E-18
CACNB3	rs151022896	G	A	0.224133188	0.05046794	8.95E-06	0.4732	0.1354	0.0004738	2.111245	0.604105	0.000474
CACNB3	rs1726440	T	C	-0.056303284	0.01227132	4.47E-06	-0.0881	0.0418	0.03519	1.56474	0.742408	0.035061
CACNB3	rs2453473	C	G	-0.071748462	0.01238922	6.99E-09	-0.1506	0.0384	8.74E-05	2.099	0.535203	8.79E-05
CACNB3	rs78957863	C	A	0.255840293	0.04244903	1.67E-09	-0.2311	0.1003	0.02118	-0.9033	0.392041	0.021218
CACNB3	rs9668292	C	A	0.332396477	0.01006987	1.00E-200	0.0795	0.0304	0.008972	0.239172	0.091457	0.008919
CACNB3	rs9739878	A	G	0.103641738	0.00831129	1.09E-35	0.1012	0.0253	6.53E-05	0.976441	0.24411	6.33E-05
CACNB4	rs12622797	A	G	0.055850504	0.01227565	5.37E-06	-0.108	0.0394	0.00606	-1.93373	0.705455	0.006123
CACNB4	rs16830878	A	G	0.156663371	0.01531845	1.50E-24	0.1722	0.0466	0.0002172	1.099172	0.297453	0.00022

gene	SNP	effect_allele	other_allele	beta.exposure	se.exposure	pval.exposure	beta.outcome	se.outcome	pval.outcome	beta	se	pval
CACNB4	rs2139419	C	T	0.058393865	0.00822936	1.29E-12	-0.1114	0.0252	9.60E-06	-1.90773	0.431552	9.84E-06
CACNB4	rs35172004	A	T	0.077479793	0.01455977	1.03E-07	0.1455	0.0438	0.0008918	1.877909	0.565309	0.000894
CACNB4	rs4507102	T	A	0.040904869	0.00881684	3.49E-06	-0.0906	0.0273	0.0009096	-2.2149	0.667402	0.000904
CACNB4	rs6760139	C	G	-0.038751089	0.00842269	4.21E-06	0.0752	0.0252	0.00284	-1.94059	0.650304	0.002844
ESR1	rs11752282	A	G	0.086832594	0.01558318	2.52E-08	-0.1325	0.0401	0.0009413	-1.52592	0.461808	0.000952
ESR1	rs2982565	A	G	-0.103670259	0.01111757	1.11E-20	-0.1001	0.0324	0.002027	0.965561	0.312529	0.002005
ESR1	rs852002	C	T	0.057322778	0.00869726	4.37E-11	0.1128	0.0241	2.95E-06	1.967804	0.420426	2.86E-06
ESR2	rs11158505	T	C	-0.101065425	0.01592133	2.18E-10	-0.1729	0.0589	0.003305	1.710773	0.582791	0.00333
ESR2	rs61984406	A	T	-0.059898808	0.01388926	1.61E-05	-0.0881	0.0409	0.03125	1.470814	0.682818	0.031237
ESR2	rs72720372	G	C	-0.226864209	0.0144901	2.99E-55	0.1742	0.0442	7.98E-05	-0.76786	0.19483	8.11E-05
ESR2	rs79988539	A	G	0.116338642	0.01718241	1.28E-11	-0.0984	0.0492	0.04565	-0.84581	0.422903	0.0455
GPR35	rs13019955	C	A	-0.168833121	0.00857439	2.60E-86	-0.0888	0.0266	0.0008293	0.525963	0.157552	0.000843
GPR35	rs2240542	C	T	0.042961883	0.00914024	2.60E-06	0.0587	0.0267	0.02774	1.366327	0.621481	0.027913
GPR35	rs35595549	A	G	0.104245249	0.0096577	3.67E-27	0.0749	0.0298	0.01188	0.718498	0.285864	0.011957
GPR35	rs4613	G	A	-0.071191776	0.00996484	9.04E-13	-0.0529	0.0263	0.04411	0.743063	0.369425	0.044283
GPR35	rs4676396	T	C	-0.071555228	0.01051015	9.88E-12	0.0833	0.0312	0.007521	-1.16414	0.436027	0.007588
GPR35	rs62187252	T	C	0.111944622	0.01769875	2.53E-10	-0.0895	0.0452	0.04772	-0.7995	0.403771	0.047694
GPR35	rs7600847	C	A	-0.035620601	0.00832199	1.87E-05	0.1016	0.0253	5.86E-05	-2.85228	0.710263	5.92E-05
KCNMA1	rs10740466	C	T	0.097572244	0.01506023	9.25E-11	0.1162	0.0489	0.01743	1.190912	0.501167	0.017488
KCNMA1	rs1079037	A	G	-0.222561422	0.0131119	1.27E-64	-0.1833	0.0443	3.56E-05	0.823593	0.199046	3.51E-05
KCNMA1	rs11002163	C	A	-0.125005359	0.01708891	2.57E-13	-0.1122	0.0521	0.0315	0.897562	0.416782	0.031275
KCNMA1	rs2559667	C	A	0.055928135	0.01239734	6.45E-06	-0.094	0.0257	0.0002514	-1.68073	0.459518	0.000255
KCNMA1	rs2619648	G	A	0.076696978	0.00897534	1.28E-17	0.0748	0.0273	0.006217	0.975267	0.355946	0.006145
KCNMA1	rs2720003	T	C	-0.092666368	0.00878896	5.44E-26	0.0532	0.0268	0.0468	-0.5741	0.28921	0.047136
KCNMA1	rs34500161	C	T	-0.036547309	0.00814225	7.16E-06	-0.0601	0.0245	0.01421	1.644444	0.670364	0.014165
KCNMA1	rs4979900	A	C	-0.240975192	0.00920128	3.51E-151	-0.0687	0.0281	0.0144	0.285092	0.11661	0.014492
KCNMA1	rs607483	A	G	0.258082868	0.00949763	1.35E-162	0.1009	0.0289	0.000486	0.39096	0.11198	0.000481
NR3C2	rs13109093	C	A	0.055039656	0.00821438	2.08E-11	-0.0576	0.0248	0.0203	-1.04652	0.450584	0.020202
PLAU	rs11000852	C	T	-0.050106153	0.00799856	3.74E-10	-0.1948	0.0244	1.28E-15	3.887746	0.486966	1.42E-15
PLAU	rs2227581	A	G	-0.121605738	0.02027878	2.01E-09	-0.1786	0.0609	0.003391	1.468681	0.500799	0.003361
PLAU	rs4746158	G	A	0.086478479	0.00871223	3.21E-23	0.111	0.0305	0.0002777	1.283556	0.352689	0.000273
PLAU	rs67145629	C	A	0.138387944	0.02420174	1.08E-08	0.2928	0.0845	0.0005315	2.115791	0.610602	0.00053
PLAU	rs75118385	A	G	0.470371011	0.02948092	2.63E-57	0.3342	0.1094	0.002247	0.710503	0.232582	0.002252
PLAU	rs9664186	T	C	0.105571798	0.00816721	3.20E-38	0.2674	0.026	9.11E-25	2.532873	0.246278	8.27E-25

gene	SNP	effect_allele	other_allele	beta.exposure	se.exposure	pval.exposure	beta.outcome	se.outcome	pval.outcome	beta	se	pval
SCNN1A	rs767455	C	T	-0.046877464	0.00802257	5.11E-09	0.0777	0.0246	0.001618	-1.65751	0.524772	0.001586
SCNN1D	rs12077700	T	C	-0.317243697	0.03955854	1.06E-15	-0.3116	0.1085	0.004071	0.98221	0.342008	0.00408
SCNN1D	rs1815606	T	G	-0.087180009	0.01043672	6.64E-17	-0.0855	0.0273	0.001749	0.980729	0.313145	0.001737
SCNN1D	rs307359	A	G	-0.076842646	0.01772201	1.45E-05	-0.2613	0.0507	2.53E-07	3.400456	0.65979	2.55E-07
SCNN1D	rs4590	G	C	-0.096701458	0.01193006	5.24E-16	-0.121	0.0362	0.0008459	1.251274	0.374348	0.00083
SLC12A1	rs1561484	T	C	-0.065657558	0.01312994	5.71E-07	0.0775	0.0267	0.003719	-1.18037	0.406655	0.003701
SLC12A1	rs16961033	A	G	-0.244583446	0.01671348	1.70E-48	0.1452	0.034	1.95E-05	-0.59366	0.139012	1.95E-05
SLC12A1	rs17463995	T	C	0.063615663	0.01204614	1.28E-07	-0.0774	0.0247	0.001756	-1.21668	0.388269	0.001727
SLC12A1	rs28381090	A	G	-0.434210939	0.07819253	2.80E-08	0.3144	0.1569	0.04506	-0.72407	0.361345	0.045089
SLC12A1	rs4775755	C	T	1.233427869	0.01085864	1.00E-200	-0.101	0.0313	0.001253	-0.08189	0.025376	0.001252
SLC12A1	rs62011404	C	A	-0.242058424	0.0534723	5.98E-06	0.2245	0.1052	0.03284	-0.92746	0.434606	0.03284
SLC12A1	rs689304	T	C	0.316099286	0.02080655	3.98E-52	-0.3961	0.0408	3.00E-22	-1.25309	0.129073	2.78E-22
SLC12A2	rs11948128	G	A	-0.18502626	0.02573276	6.46E-13	-0.3472	0.1078	0.00128	1.87649	0.58262	0.001278
SLC12A2	rs1235244	G	T	-0.102465019	0.0200213	3.09E-07	-0.1399	0.0587	0.01721	1.365344	0.572878	0.017158
SLC12A2	rs17606751	A	G	-0.037227348	0.00797672	3.05E-06	-0.0942	0.0246	0.0001258	2.530398	0.660805	0.000129
SLC12A2	rs27855	A	G	-0.040365339	0.00819551	8.42E-07	0.085	0.0245	0.0005306	-2.10577	0.606956	0.000522
SLC12A2	rs3101725	T	C	-0.178680133	0.00958584	1.52E-77	-0.1783	0.0286	4.57E-10	0.997873	0.160063	4.54E-10
SLC12A2	rs6860901	T	C	0.045821206	0.00869654	1.37E-07	0.3157	0.0261	1.09E-33	6.889823	0.569605	1.11E-33
SLC12A2	rs73337306	T	A	0.144875718	0.01320895	5.46E-28	0.3082	0.0382	7.74E-16	2.127341	0.263674	7.14E-16
SLC12A3	rs11864751	T	C	0.058003764	0.01307569	9.17E-06	0.1373	0.0274	5.23E-07	2.367088	0.472383	5.42E-07
SLC12A3	rs158479	G	A	-0.056103949	0.01198546	2.85E-06	-0.0567	0.0247	0.02164	1.010624	0.440254	0.021702
SLC12A3	rs2289114	T	C	0.071216645	0.0145861	1.05E-06	0.1211	0.0311	9.60E-05	1.700445	0.436696	9.86E-05
SLC12A3	rs8048885	T	A	0.058587806	0.01330422	1.06E-05	-0.1317	0.0274	1.53E-06	-2.24791	0.467674	1.54E-06
SLC12A4	rs6499167	T	A	-0.039475037	0.00884119	8.01E-06	0.071	0.0268	0.008009	-1.79861	0.67891	0.008067
SLC9A1	rs11247626	T	C	0.083138332	0.00905982	4.46E-20	-0.1406	0.0287	9.46E-07	-1.69116	0.345208	9.63E-07
SLC9A1	rs17262181	A	G	-0.070996077	0.01602729	9.43E-06	0.3157	0.0498	2.25E-10	-4.44672	0.701447	2.31E-10
SLC9A1	rs2504775	A	G	0.078552253	0.01436791	4.57E-08	-0.2913	0.0424	6.60E-12	-3.70836	0.539768	6.41E-12

Note: "beta.exposure" is obtained from eQTLGen; "beta.outcome" is obtained from the systolic blood pressure (SBP) GWAS; "beta" represnet the association between gene expression and SBP, calculated using two-sample Mendelian randomisation Wald method.

Table S16. Sensitivity analysis: exclusion of RAS-inhibitor instruments nominally associated with NAFLD

To test the exclusion-restriction assumption, the renin–angiotensin system (RAS) inhibitor drug-target analysis was repeated after removing every genetic instrument that was itself nominally associated with NAFLD ($P < 0.05$ in the Million Veteran Program NAFLD GWAS), as such variants might act on NAFLD through pathways independent of systolic blood pressure. All effects are expressed as odds ratios per 5 mmHg lower genetically predicted systolic blood pressure.

Target gene	Instruments (full), n	OR (95% CI), full	Instruments retained, n	Instruments excluded, n	OR (95% CI), after exclusion
LTA4H	14	0.93 (0.65–1.32)	13	1	0.99 (0.71–1.39)
MMP9	6	0.85 (0.57–1.26)	6	0	0.85 (0.57–1.26)
ACE	1	0.87 (0.63–1.21)	1	0	0.87 (0.63–1.21)
JUN	7	0.64 (0.45–0.91)	6	1	0.83 (0.55–1.26)
PPARG	15	0.50 (0.36–0.70)	12	3	0.72 (0.56–0.93)
Pooled RAS class	43	0.73 (0.58–0.93)	38	5	0.83 (0.72–0.96)

Heterogeneity: full RAS class $I^2 = 55\%$ (Cochran's Q $P = 0.06$; random-effects estimate shown). After exclusion, $I^2 = 0\%$ (Q $P = 0.66$); fixed- and random-effects estimates coincide at OR 0.83 (95% CI 0.72–0.96).

Excluded instruments (nominally associated with NAFLD, P < 0.05)

Target gene	SNP	NAFLD P-value	NAFLD effect (log-OR)
LTA4H	rs1362971	4.8×10^{-2}	−0.016
JUN	rs67430108	5.9×10^{-4}	−0.046
PPARG	rs3729931	1.7×10^{-5}	−0.033
PPARG	rs4135250	5.3×10^{-10}	−0.069
PPARG	rs73029213	9.1×10^{-3}	+0.029

Methods: per-gene causal estimates were obtained by inverse-variance-weighted meta-analysis of the cis instruments (Wald ratio where a single instrument was available) and pooled across target genes by fixed-or random-effects meta-analysis (random effects where Cochran's Q $P < 0.05$). Instruments were the eQTLGen cis-eQTLs for each RAS target gene that were associated with systolic blood pressure; the NAFLD outcome was the Million Veteran Program GWAS (68,725 cases, 95,472 controls). The full RAS class estimate reproduces the published primary result exactly (OR 0.73, 95% CI 0.62–0.85 fixed; 0.58–0.93 random). Computed from the harmonised drug-target × NAFLD data (dat_ACEIs_SBP_NAFLD.Rdata and dat_ARBs_SBP_NAFLD.Rdata).

Table S17. Summary of published and ongoing clinical trials and systematic reviews/meta-analyses assessing the effect of renin–angiotensin system (RAS) inhibitors on non-alcoholic fatty liver disease (NAFLD)/non-alcoholic steatohepatitis (NASH) outcomes

First author (year)	Study design	RAS inhibitor (dose)	Comparator	N	Follow-up	Hepatic outcomes / biomarkers	Key findings	Citation (with DOI)
**A. Interventional clinical trials**								
Yokohama (2004)	Open-label, single-arm pilot	Losartan 50 mg/day	None (before–after)	7	48 weeks	Liver histology; aminotransferases; TGF-β1; ferritin	Improved aminotransferases; histological necroinflammation improved in 5/7 and fibrosis in 4/7; ↓TGF-β1 and ferritin	Yokohama S, et al. Hepatology. 2004;40(5):1222–1225. https://doi.org/10.1002/hep.20420
Eriksson (2008) – MEDICA	Multicentre 3-way crossover, double-blind RCT	Candesartan 16–32 mg/day	Hydrochlorothiazide 25–50 mg; placebo	26 (22 completed)	12 weeks per period	Liver fat (^{1}H-MRS); insulin sensitivity (clamp); visceral fat	Hydrochlorothiazide raised liver fat and worsened insulin resistance, whereas candesartan was neutral on hepatic fat	Eriksson JW, et al. Hypertension. 2008;52(6):1030–1037. https://doi.org/10.1161/HYPERTENSIONAHA.108.119404
Georgescu (2009)	Single-blind RCT, head-to-head	Telmisartan 20 mg/day	Valsartan 80 mg/day	54	20 months	Liver histology (NAS); HOMA-IR; ALT/AST	ALT fell in both arms; NAFLD activity score and insulin resistance improved more with telmisartan	Georgescu EF, et al. World J Gastroenterol. 2009;15(8):942–954. https://doi.org/10.3748/wjg.15.942
Torres (2011)	Open-label RCT (3-arm)	Rosiglitazone + losartan 50 mg/day	Rosiglitazone; rosiglitazone + metformin	137 (108 completed)	48 weeks	Liver histology (steatosis, inflammation, fibrosis)	Adding losartan to rosiglitazone gave no additional histological benefit over rosiglitazone alone	Torres DM, et al. Hepatology. 2011;54(5):1631–1639. https://doi.org/10.1002/hep.24558
Fogari (2012)	Double-blind RCT	Losartan 100 mg/day (± simvastatin)	Amlodipine 10 mg/day (± simvastatin)	150	12 months	Steatosis (ultrasound); insulin sensitivity (GIR); adipose tissue	Losartan reduced steatosis and improved insulin sensitivity vs amlodipine; simvastatin add-on further reduced steatosis	Fogari R, et al. Eur J Gastroenterol Hepatol. 2012;24(2):164–171. https://doi.org/10.1097/MEG.0b013e32834ba188
Hidaka (2013)	Open-label, single-arm	Olmesartan 10–40 mg/day (median 20 mg)	None (before–after)	7	48 weeks	Liver histology; fibrosis markers (HA, type IV collagen, PIIINP); AST/ALT; ferritin	↓AST, ALT, ferritin, fasting glucose and blood pressure; histology, TGF-β1 and fibrosis markers unchanged (anti-inflammatory but not antifibrotic)	Hidaka H, et al. Open J Gastroenterol. 2013;3(2):128–133. https://doi.org/10.4236/ojgas.2013.32021
Hirata	Open-label	Telmisartan 40	Losartan 50 mg/day	19	12 months	Liver-to-spleen ratio	Telmisartan ↓free fatty	Hirata T, et al. Int J Endocrinol. 2013;2013:587140.

First author (year)	Study design	RAS inhibitor (dose)	Comparator	N	Follow-up	Hepatic outcomes / biomarkers	Key findings	Citation (with DOI)
(2013) – FANTASY	RCT, head-to-head	mg/day				(CT); serum free fatty acids; liver enzymes	acids and ↑liver-to-spleen ratio (reduced liver fat); no significant change with losartan	https://doi.org/10.1155/2013/587140
Derosa (2015)	Double-blind RCT	Perindopril 5 mg/day (ACE inhibitor; ± simvastatin)	Barnidipine 20 mg/day (± simvastatin)	149	12 months	Steatosis (ultrasound); inflammatory markers	Hepatic steatosis improved only after simvastatin add-on; perindopril alone did not significantly change steatosis	Derosa G, et al. Eur J Pharmacol. 2015;766:31–36. https://doi.org/10.1016/j.ejphar.2015.09.030
Alam (2016)	Open-label RCT	Telmisartan 40–80 mg/day + lifestyle	Lifestyle modification alone	50 (30 completed)	1 year	Liver histology (NAS, fibrosis); ALT/AST; HOMA-IR	Telmisartan significantly improved steatosis, ballooning, lobular inflammation and fibrosis vs control	Alam S, et al. Saudi J Gastroenterol. 2016;22(1):69–76. https://doi.org/10.4103/1319- 3767.173762
McPherson (2017)	Double-blind, placebo-controlled RCT	Losartan 50 mg/day	Placebo	45 (32 completed)	96 weeks	Histological fibrosis stage	Underpowered owing to recruitment difficulty (widespread baseline ACEI/ARB use); no significant fibrosis difference	McPherson S, et al. PLoS One. 2017;12(4):e0175717. https://doi.org/10.1371/journal.pone.0175717
Al-Nimer (2019)	Open-label, randomised, placebo-controlled	Telmisartan 20 mg/day	Placebo	51	8 weeks	AST; lipids; triglyceride-glucose index; IL-1β/IL-18; fibrosis assessment	Telmisartan ↓AST, waist circumference, blood pressure, triglyceride-glucose index and inflammasome markers (IL-1β, IL-18); lower fibrosis vs placebo	Al-Nimer MSM, Esmail VAW, Mohammad O. Electron J Gen Med. 2019;16(3):em142. https://doi.org/10.29333/ejgm/104568
Alam (2020)	Open-label, noninferiority RCT	Telmisartan 40 mg/day	Vitamin E 800 IU/day	40	1 year	Liver histology (NAS, fibrosis, steatosis, ballooning)	Telmisartan was noninferior to vitamin E in improving NASH histology; both improved the NAFLD activity score	Alam S, et al. JGH Open. 2020;4(4):663–669. https://doi.org/10.1002/jgh3.12315
Esmail / Al-Nimer (2022)	Open-label RCT (3-arm)	Telmisartan 20 mg/day	Orlistat 120 mg/day; placebo	74 (telmisartan n=24)	8 weeks (telmisartan arm)	Serum free fatty acids; triglyceride-glucose index; lipid profile	Telmisartan (and orlistat) significantly reduced free fatty acids and triglyceride-glucose index vs placebo	Wasta Esmail VA, Al-Nimer MSM, Mohammed MO. Turk J Gastroenterol. 2022;33(5):421–426. https://doi.org/10.5152/tjg.2020.19365
Vos (2022)	Multicentre, double-blind, placebo-controlled RCT (paediatric)	Losartan 100 mg/day	Placebo	83	24 weeks	ALT (primary); AST; GGT; alkaline phosphatase; HOMA-IR; blood pressure	Stopped early for futility; no significant ALT difference; alkaline phosphatase and systolic blood pressure fell with losartan	Vos MB, et al. Hepatology. 2022;76(2):429–444. https://doi.org/10.1002/hep.32403
**B. Systematic reviews and meta-analyses**								

First author (year)	Study design	RAS inhibitor (dose)	Comparator	N	Follow-up	Hepatic outcomes / biomarkers	Key findings	Citation (with DOI)
**First author (year)**	**Study design**	**RAS inhibitor (dose)**	**Comparator**	**N**	**Follow-up**	**Hepatic outcomes / biomarkers**	**Key findings**	**Citation (with DOI)**
Li (2018)	Systematic review & meta-analysis (4 RCTs + 8 single-arm)	Angiotensin receptor blockers (various)	Various	887	Variable	ALT; fibrosis score; NAS; LDL	ALT, fibrosis score and NAS not significantly changed overall; LDL significantly reduced	Li Y, et al. Oncotarget. 2018;9(35):24155–24167. https://doi.org/10.18632/oncotarget.23816
Meng (2023)	Systematic review & meta-analysis (6 RCTs)	Losartan (various doses)	Various	408	Variable	AST; ALT; total cholesterol; triglycerides; LDL; HDL	Losartan ↓AST overall (MD −5.34); 50 mg subgroup ↓ALT (MD −18.92); no significant lipid change	Meng C, et al. Open Life Sci. 2023;18(1):20220583. https://doi.org/10.1515/biol-2022-0583
**C. Ongoing trial**								
**First author (year)**	**Study design**	**RAS inhibitor (dose)**	**Comparator**	**N**	**Follow-up**	**Hepatic outcomes / biomarkers**	**Key findings**	**Citation (with DOI)**
RELIEF-NAFLD (2021–)	Phase 2, single-arm, open-label	Lisinopril (ACE inhibitor), once daily	None (single-arm)	45 (target)	24 weeks	PRO-C3 (fibrosis; primary); steatosis and liver stiffness (elastography, MRI-PDFF)	Active, not recruiting; results pending	ClinicalTrials.gov NCT04550481. https://clinicaltrials.gov/study/NCT04550481

Abbreviations: ACEI, angiotensin-converting enzyme inhibitor; ARB, angiotensin receptor blocker; ALT, alanine aminotransferase; AST, aspartate aminotransferase; CT, computed tomography; FFA, free fatty acids; GGT, gamma-glutamyl transferase; GIR, glucose infusion rate; HA, hyaluronic acid; HOMA-IR, homeostatic model assessment of insulin resistance; IL, interleukin; MRI-PDFF, magnetic resonance imaging proton-density fat fraction; NAS, NAFLD activity score; NASH, non-alcoholic steatohepatitis; PIIINP, procollagen III N-terminal propeptide; PRO-C3, N-terminal type III collagen propeptide; TGF-β1, transforming growth factor-β1. Studies are predominantly angiotensin receptor blocker (ARB) trials; Derosa (2015) and RELIEF-NAFLD evaluate ACE inhibitors. Several studies are small, single-arm or open-label and of limited methodological quality.

PART D

Supplementary Figures

Figure S1. Schematic overview of the Mendelian randomisation principle and its comparison to randomised controlled trials. BP: blood pressure.

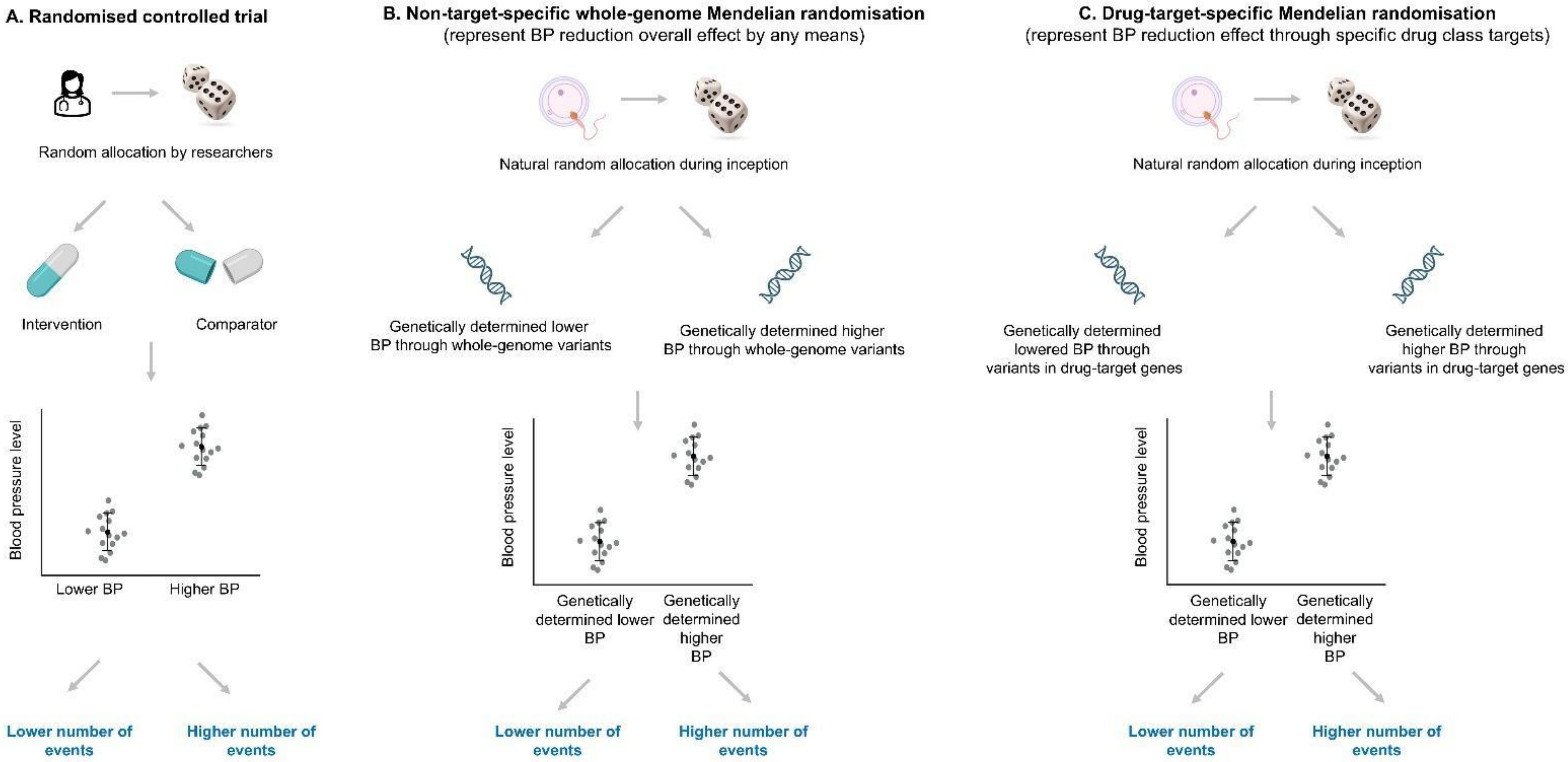

Figure S2. Flowchart of the stages of the drug-target Mendelian randomisation study for evaluating the effects of antihypertensive drug classes. MR: Mendelian randomisation; SBP: systolic blood pressure.

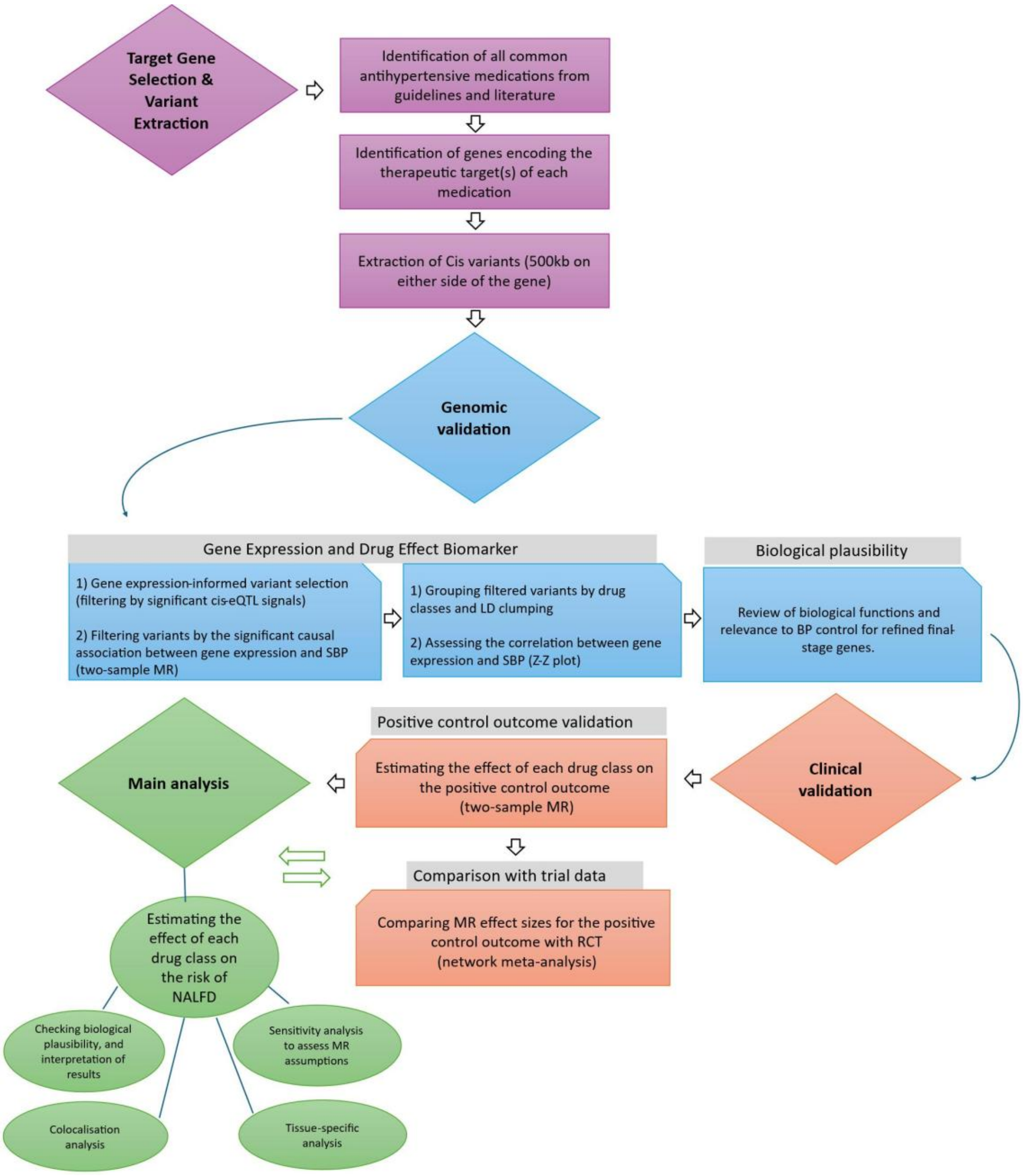

Figure S3. Results of the Mendelian randomisation analysis using inverse variance weighting, along with sensitivity analyses, to assess the association between systolic blood pressure lowering and non-alcoholic fatty liver disease

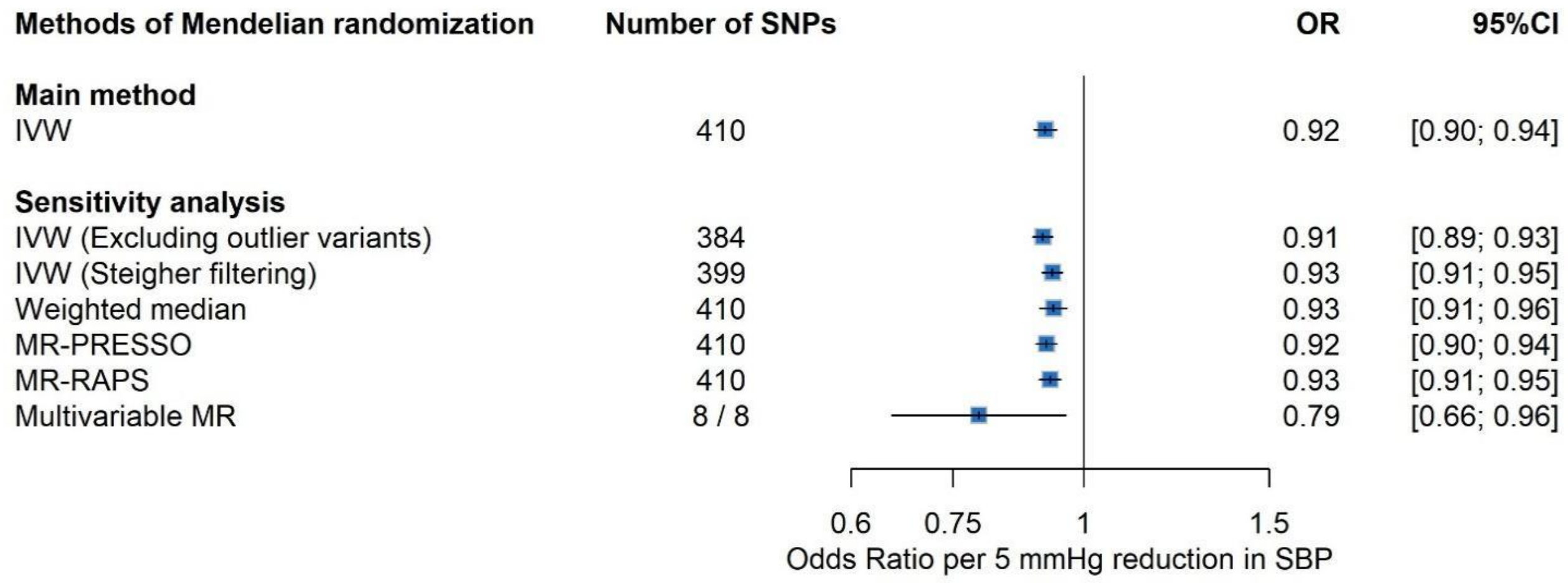

Note: the SNP number for the multivariable MR is presented as “number of SBP SNPs” / “number of BMI SNPs”. Abbreviations: IVW: inverse variance weighted method; SNPs:single nucleotide polymorphisms; SBP: systolic blood pressure; BMI: body mass index; MR: Mendelian randomisation;MR-PRESSO: MR-pleiotropy residual sum and outlier;

Figure S4. Scatter plot of genetic variant-outcome associations versus variant-exposure associations for the effect of systolic blood pressure reduction on the risk of non-alcoholic fatty liver disease (NAFLD)

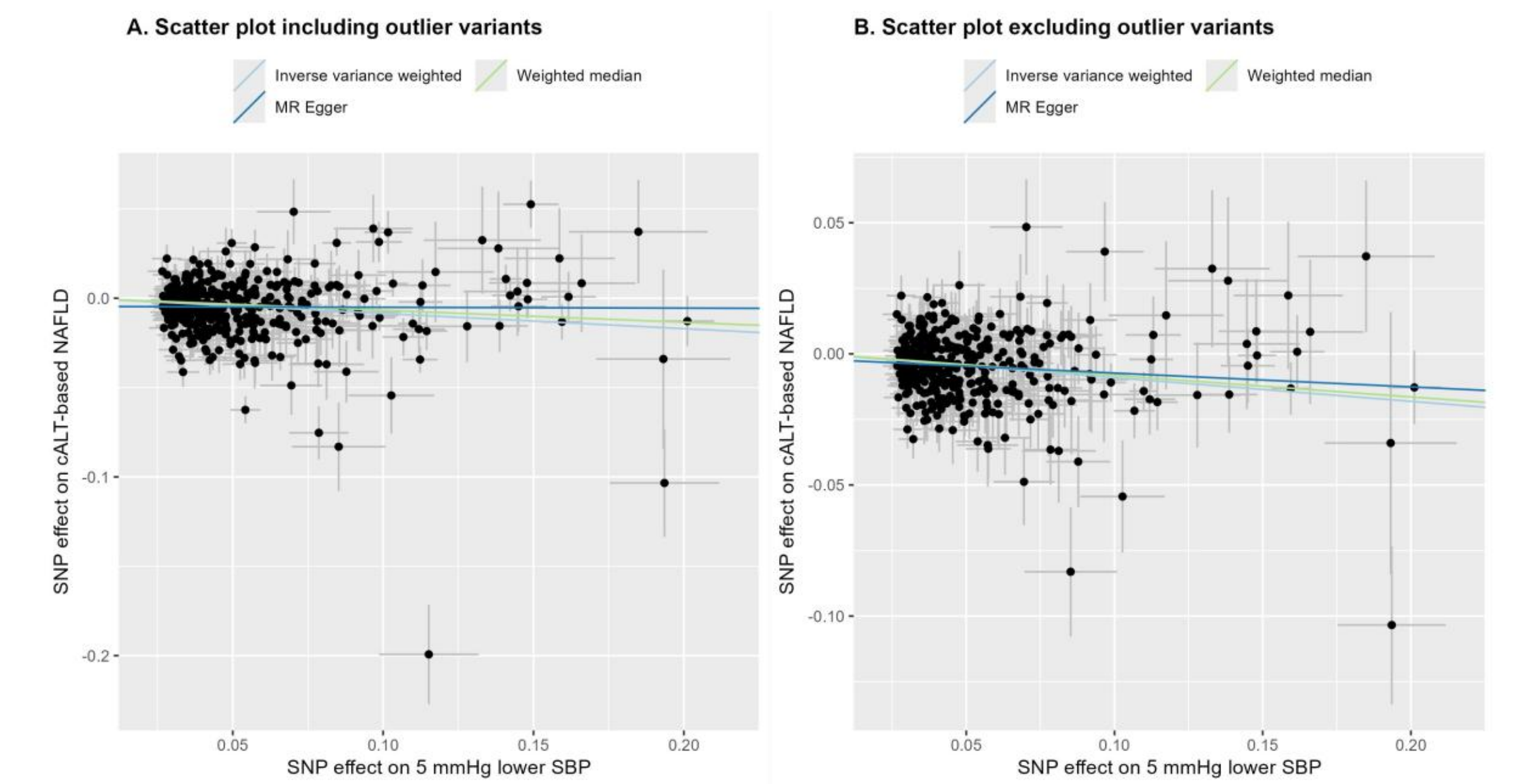

Abbreviations: SNPs: single nucleotide polymorphism; SBP: systolic blood pressure; NAFLD: non-alcoholic fatty liver disease.

Figure S5. Funnel plot of variants, displaying each variant's causal estimate against instrument strength

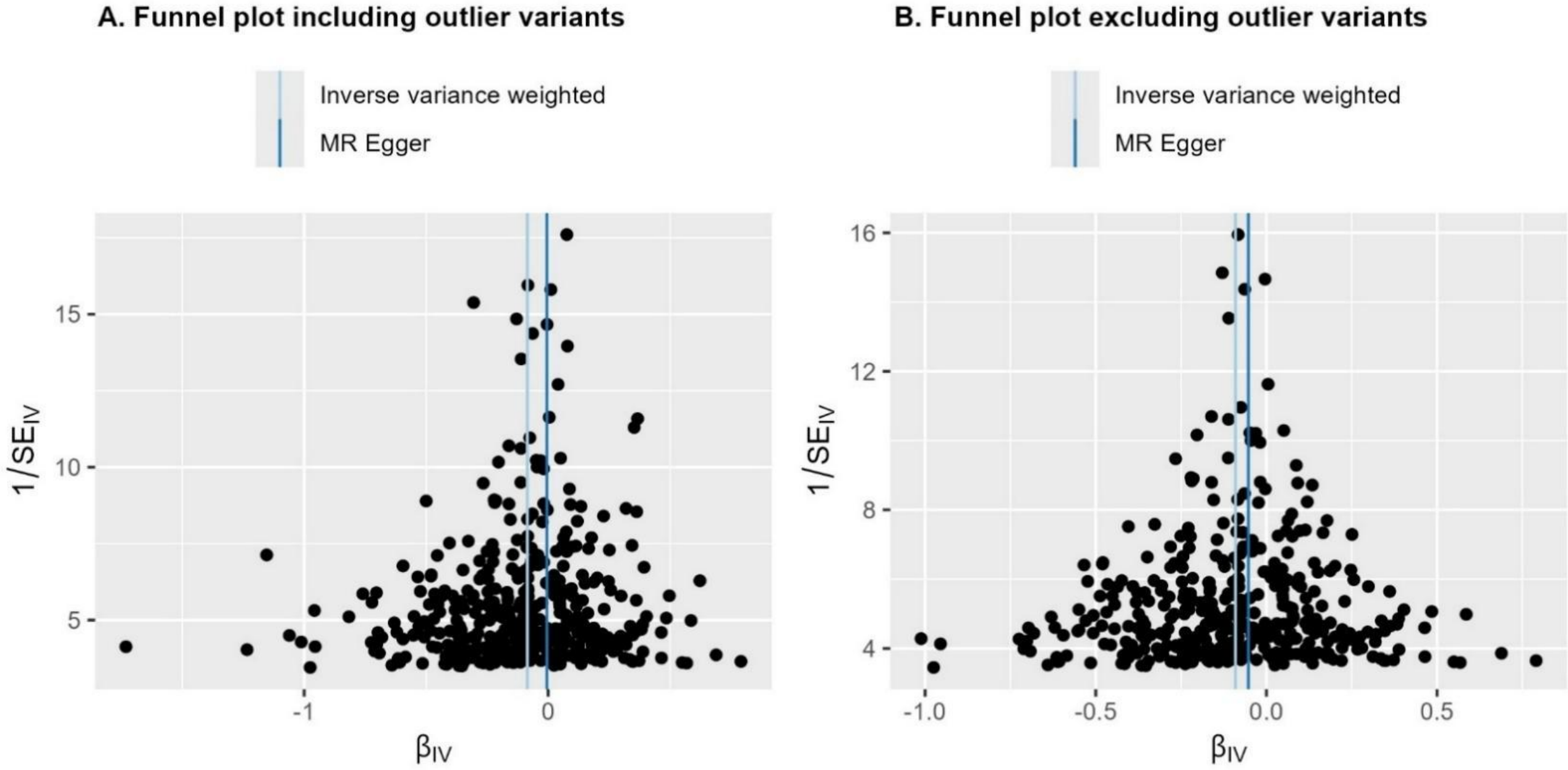

Note: β_{IV}: The causal effect of the exposure on the outcome; Abbreviation: SE: standard error.

Figure S6. Cook's Distance bar plot for identifying outlier variants to perform sensitivity analysis excluding outlier variants

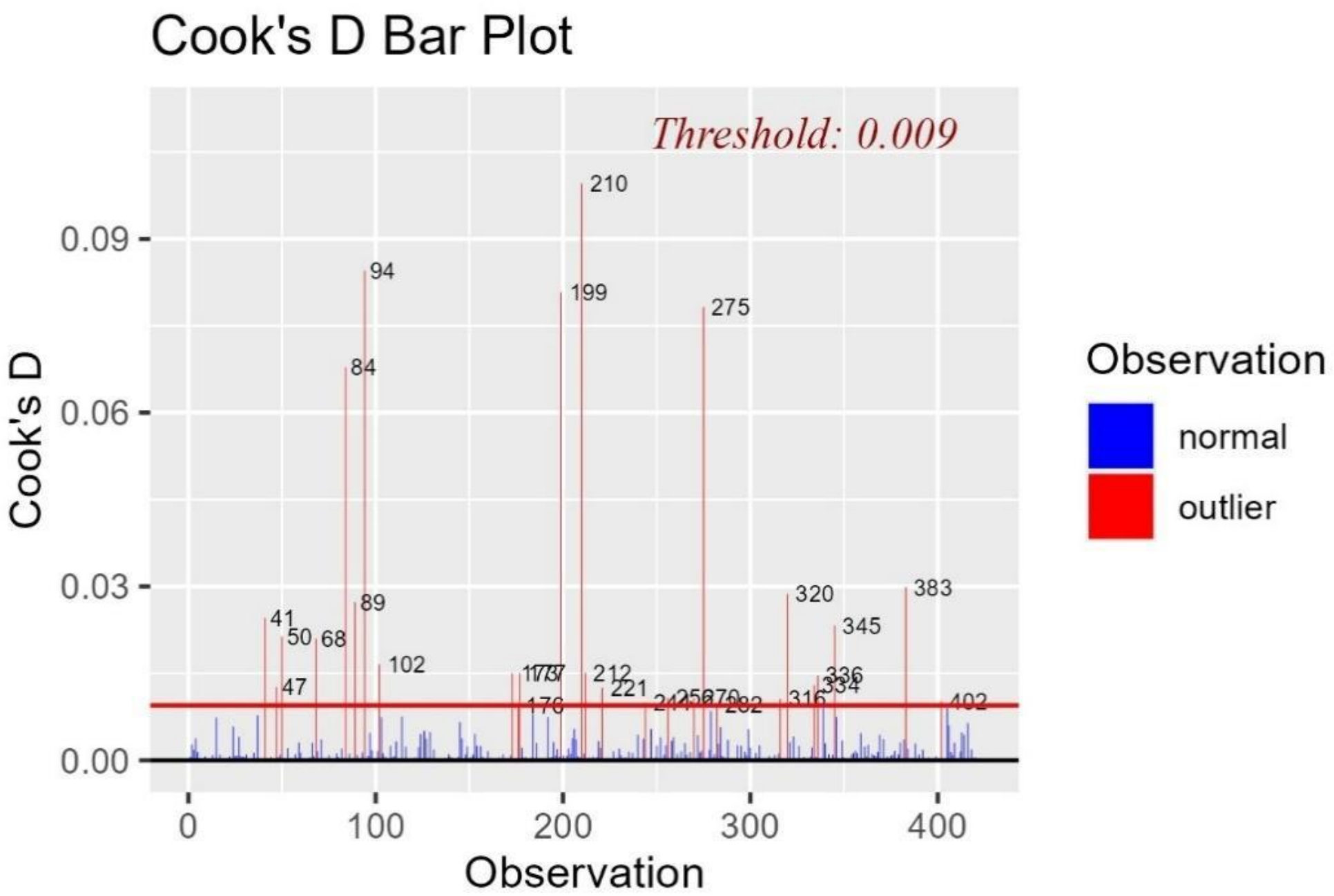

Note: The Cook's Distance threshold is calculated using 4/n, where n is the number of variants. [13-14]

Figure S7. Results of the Mendelian randomisation analysis assessing the association between systolic blood pressure lowering and non-alcoholic fatty liver disease, replicated using genome-wide association studies that employed diagnostic codes for identifying non-alcoholic fatty liver disease

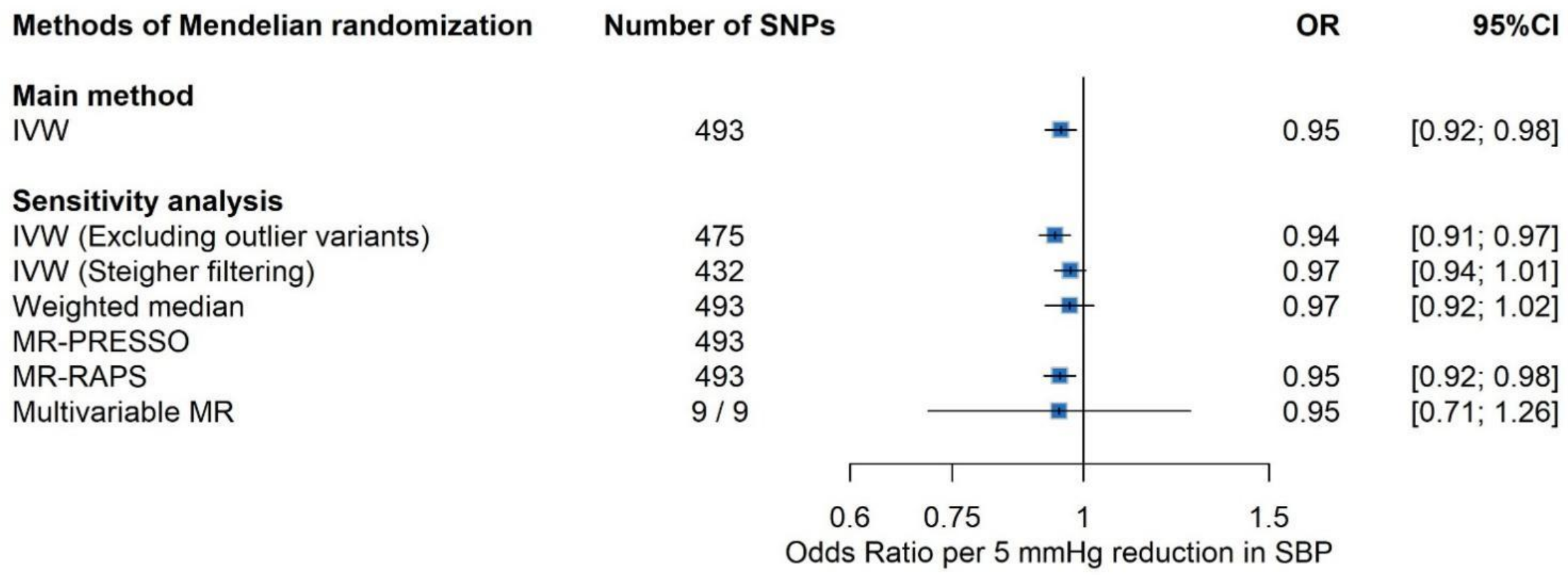

Note: the SNP number for the multivariable MR is presented as “number of SBP SNPs” / “number of BMI SNPs”. Abbreviations: IVW: inverse variance weighted method; SNPs: single nucleotide polymorphisms; SBP: systolic blood pressure; BMI: body mass index; MR: Mendelian randomisation; MR-PRESSO: MR-pleiotropy residual sum and

Figure S8. Results of the Mendelian randomisation analysis assessing the association between systolic blood pressure lowering and liver enzymes (ALT and AST) as proxies for non-alcoholic fatty liver disease

A. ALT

Methods of Mendelian randomization	Number of SNPs	Effect (SD)	95%CI
**Main method**			
IVW	458	-0.03	[-0.04; -0.02]
**Sensitivity analysis**			
IVW (Excluding outlier variants)	433	-0.02	[-0.03; -0.02]
IVW (Steigher filtering)	431	-0.03	[-0.03; -0.02]
Weighted median	458	-0.02	[-0.03; -0.01]
MR-PRESSO	458	-0.02	[-0.03; -0.02]
MR-RAPS	458	-0.02	[-0.03; -0.01]
Multivariable MR	458 / 18	-0.03	[-0.04; -0.02]

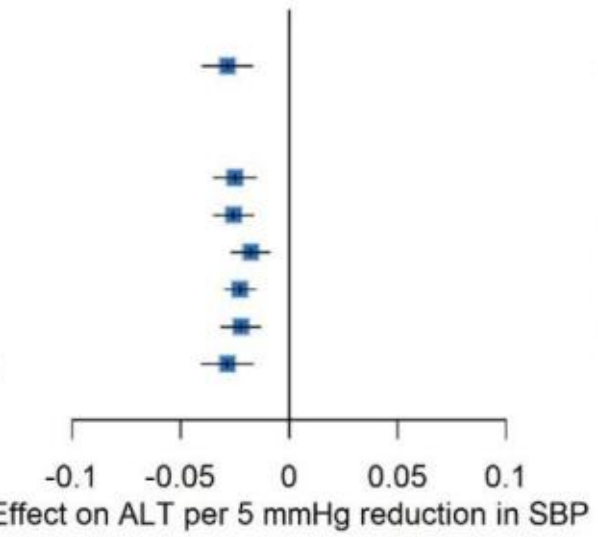

B. AST

Methods of Mendelian randomization	Number of SNPs	Effect (SD)	95%CI
**Main method**			
IVW	458	-0.03	[-0.04; -0.02]
**Sensitivity analysis**			
IVW (Excluding outlier variants)	433	-0.02	[-0.03; -0.02]
IVW (Steigher filtering)	431	-0.03	[-0.03; -0.02]
Weighted median	458	-0.02	[-0.03; -0.01]
MR-PRESSO	458	-0.02	[-0.03; -0.02]
MR-RAPS	458	-0.02	[-0.03; -0.01]
Multivariable MR	458 / 18	-0.03	[-0.04; -0.02]

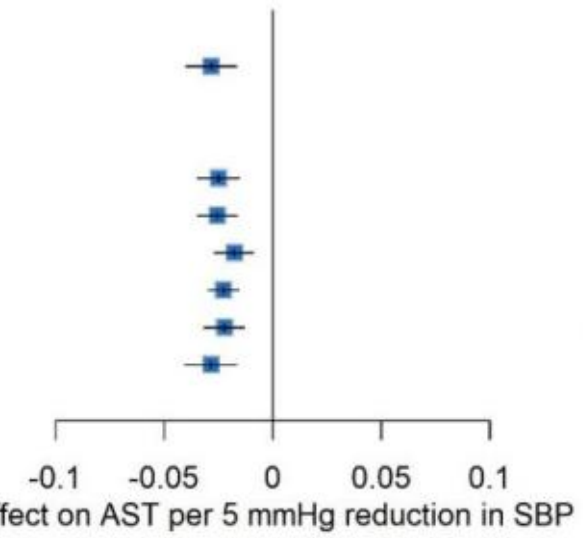

Note: the SNP number for the multivariable MR is presented as "number of SBP SNPs" / "number of BMI SNPs". Abbreviations: IVW: inverse variance weighted method; SNPs: single nucleotide polymorphisms ; SBP: systolic blood pressure; BMI: body mass index; MR: Mendelian randomisation; MR-PRESSO: MR-pleiotropy residual sum and outlier; RAPS: robust adjusted profile score; SD: standard deviation; CI: confidence interval; ALT: alanine aminotransferase; AST: aspartate aminotransferase.

Figure S9. Z-Z plot for target genes of the four major antihypertensive drug classes before and after filtering each variant by its causal association between the eQTL Z-score and the systolic blood pressure GWAS Z-score

RAS inhibitors

Before	After

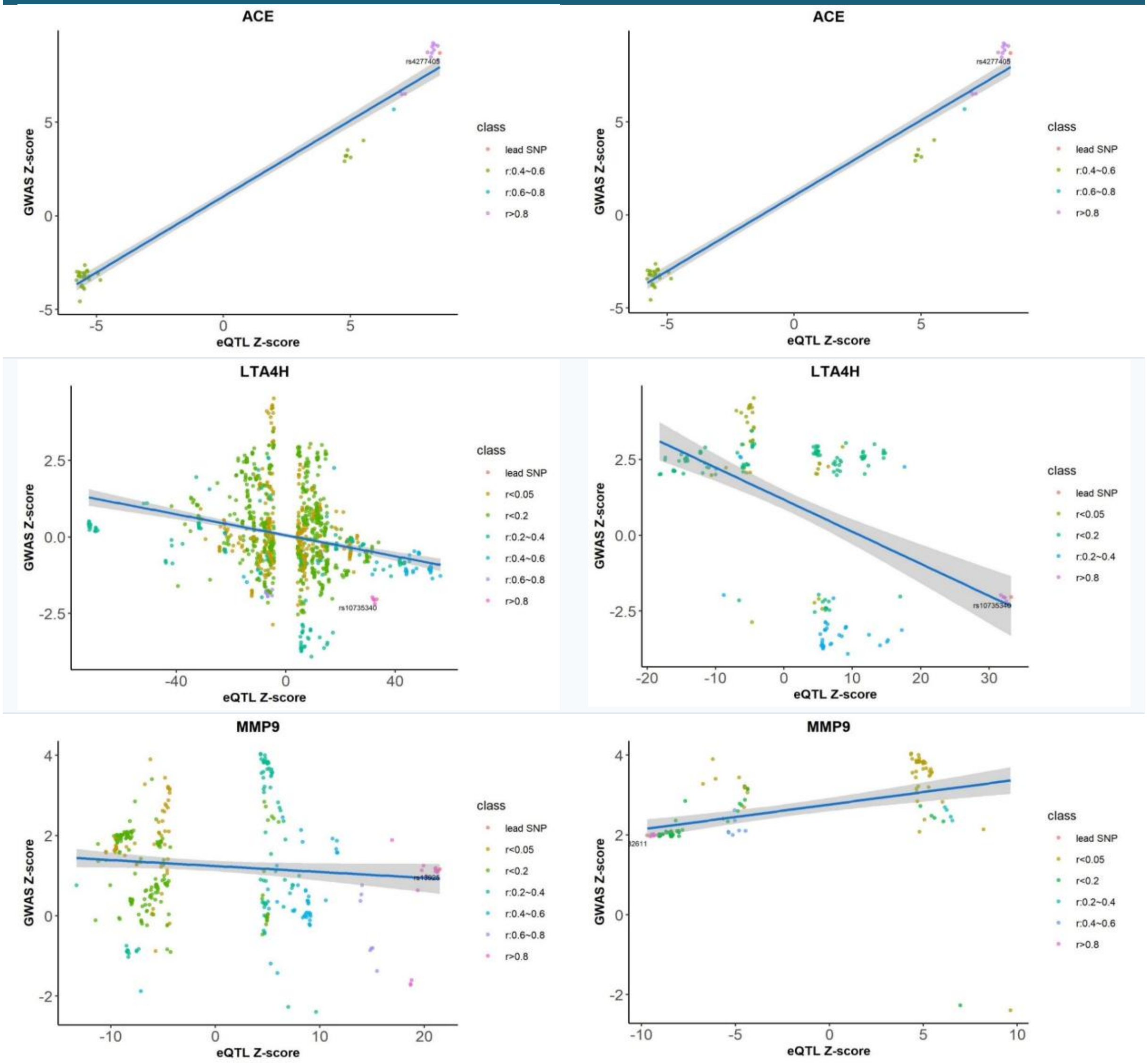

Before	After
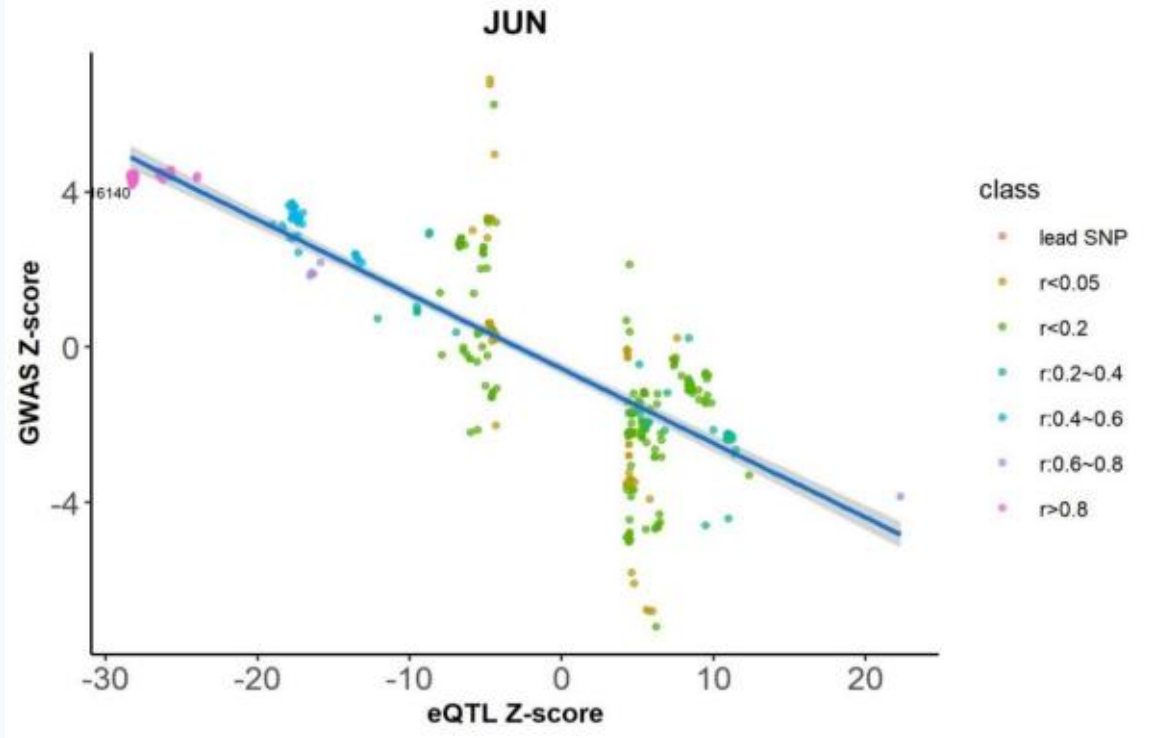	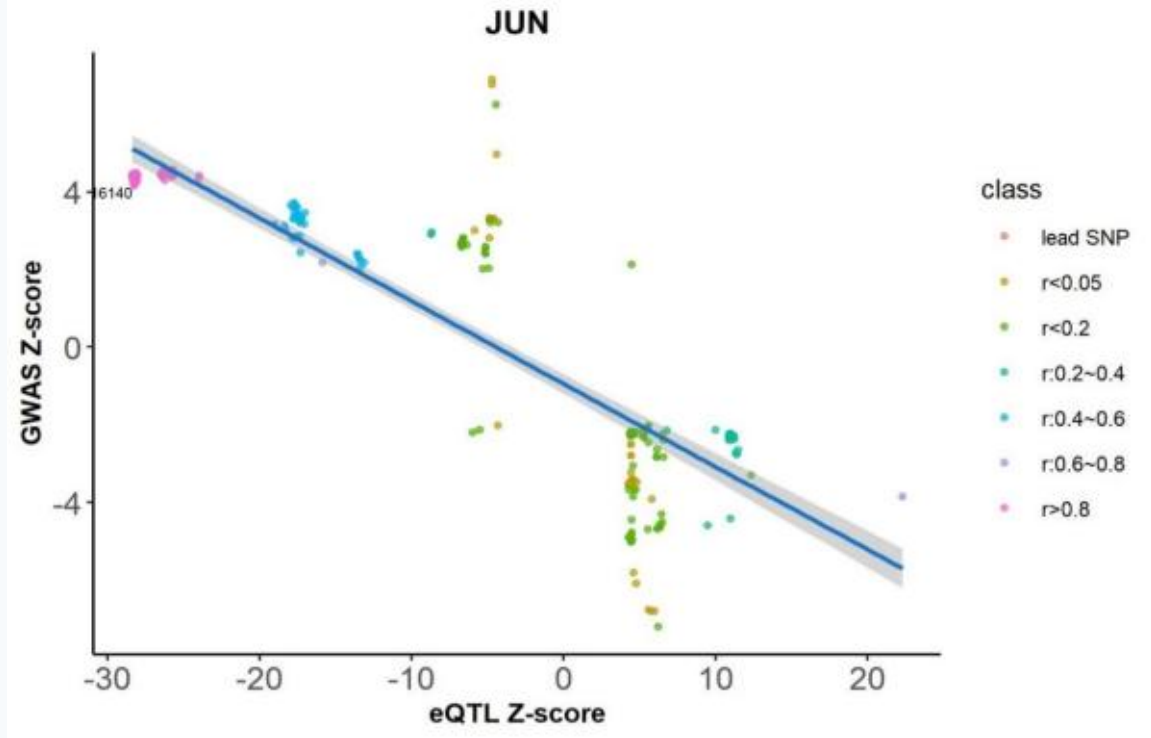
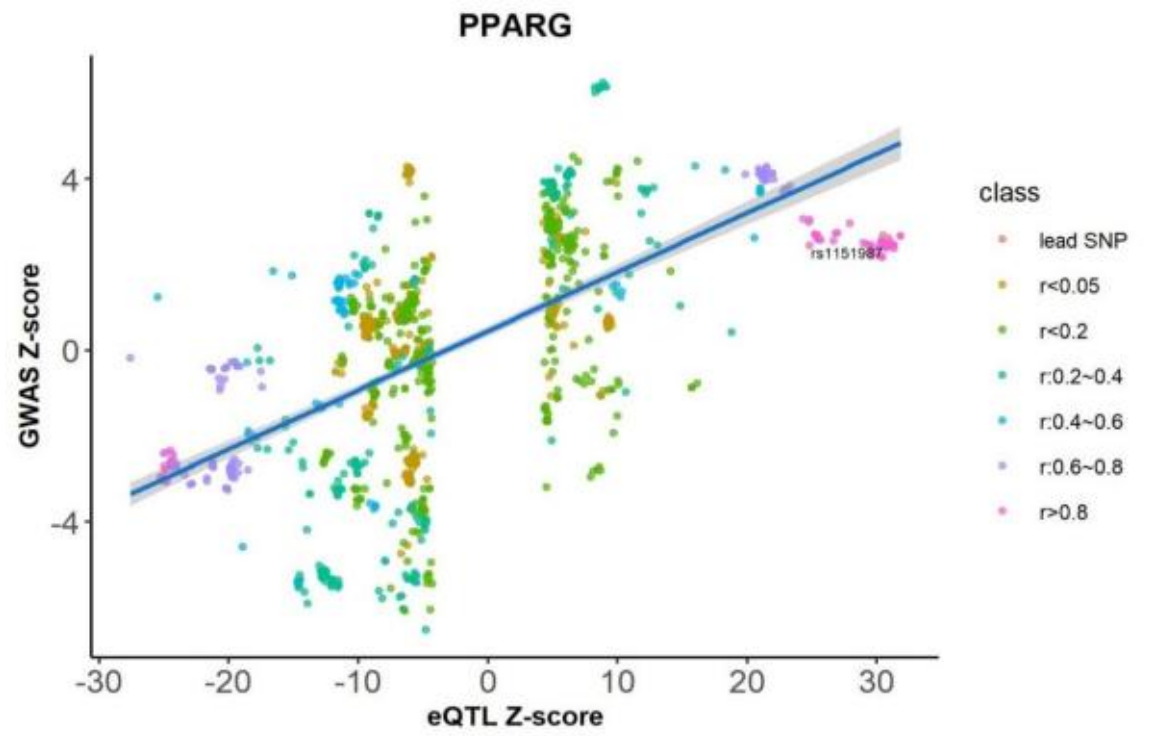	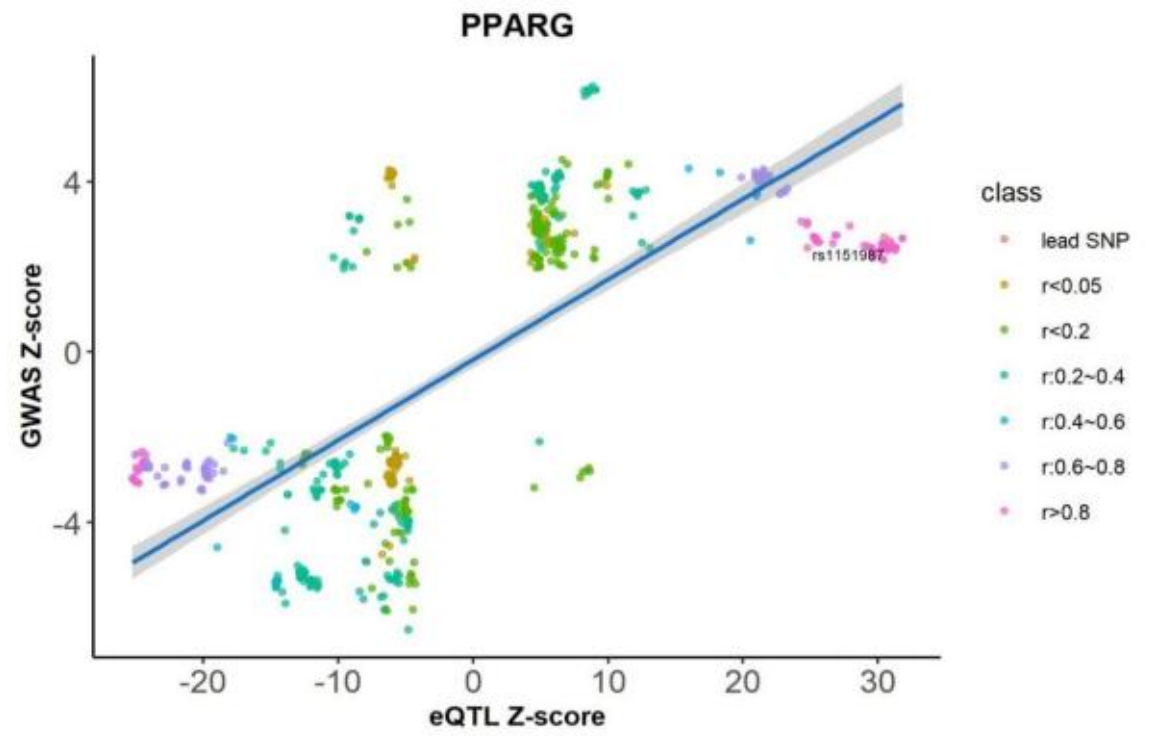

Beta blockers

Before	After
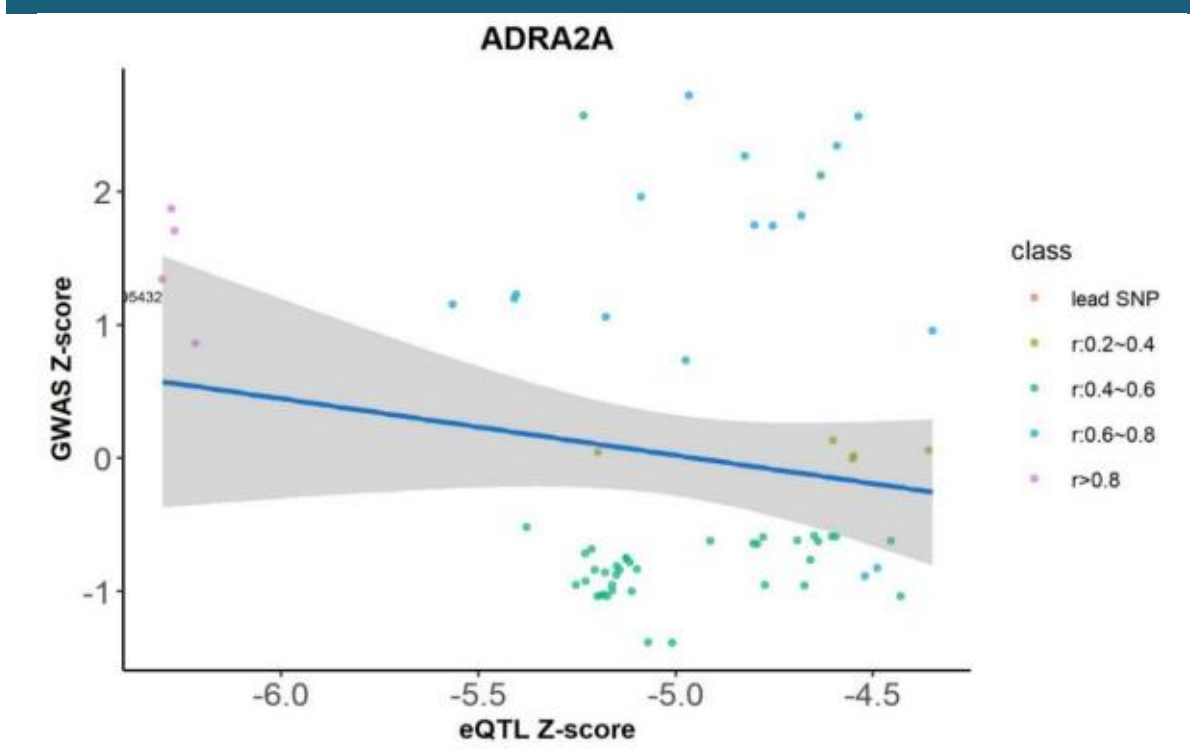	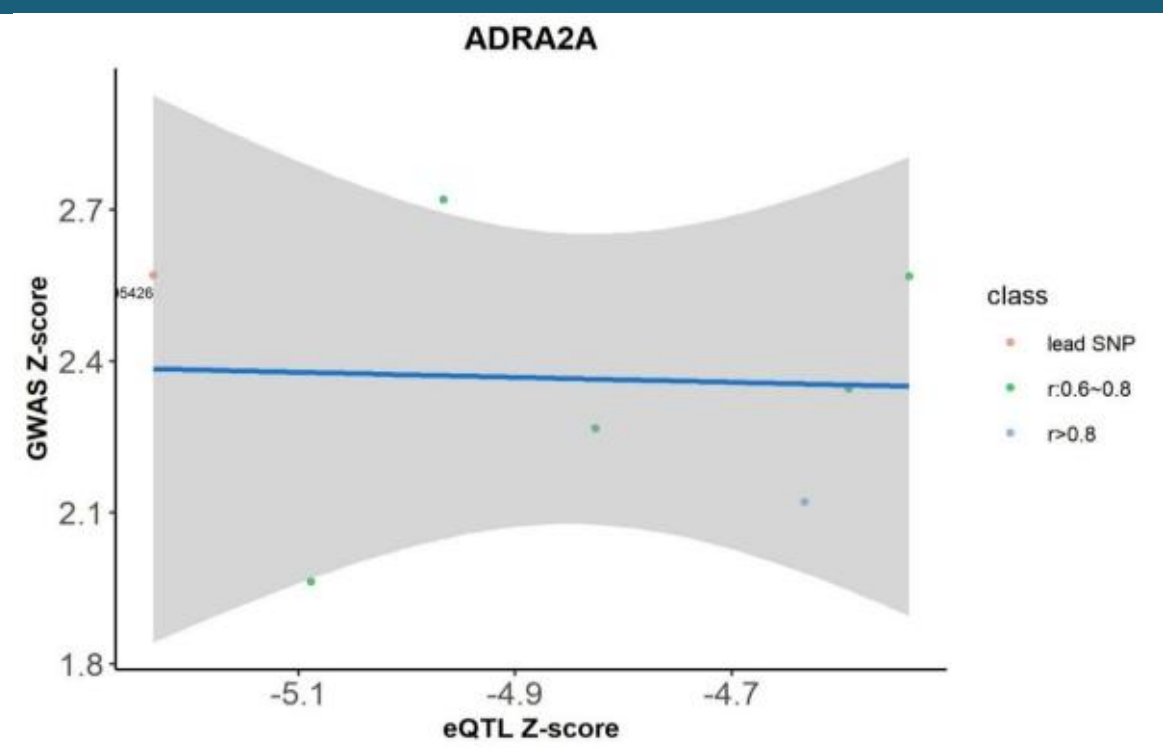
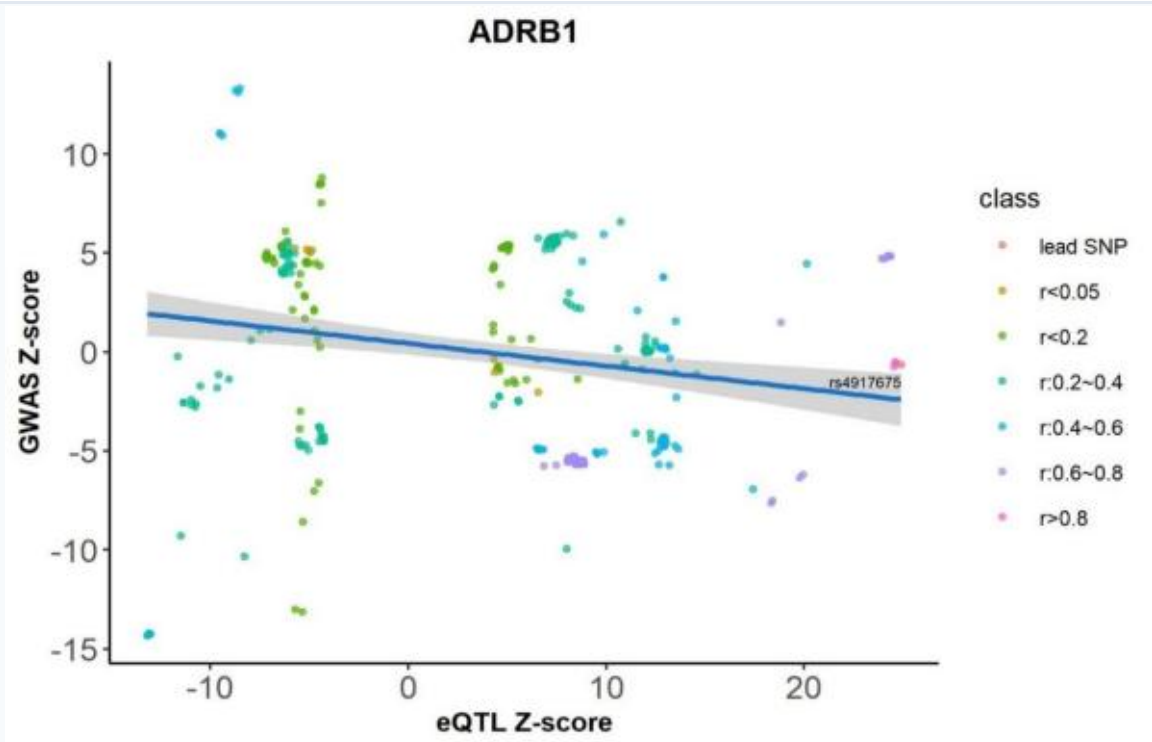	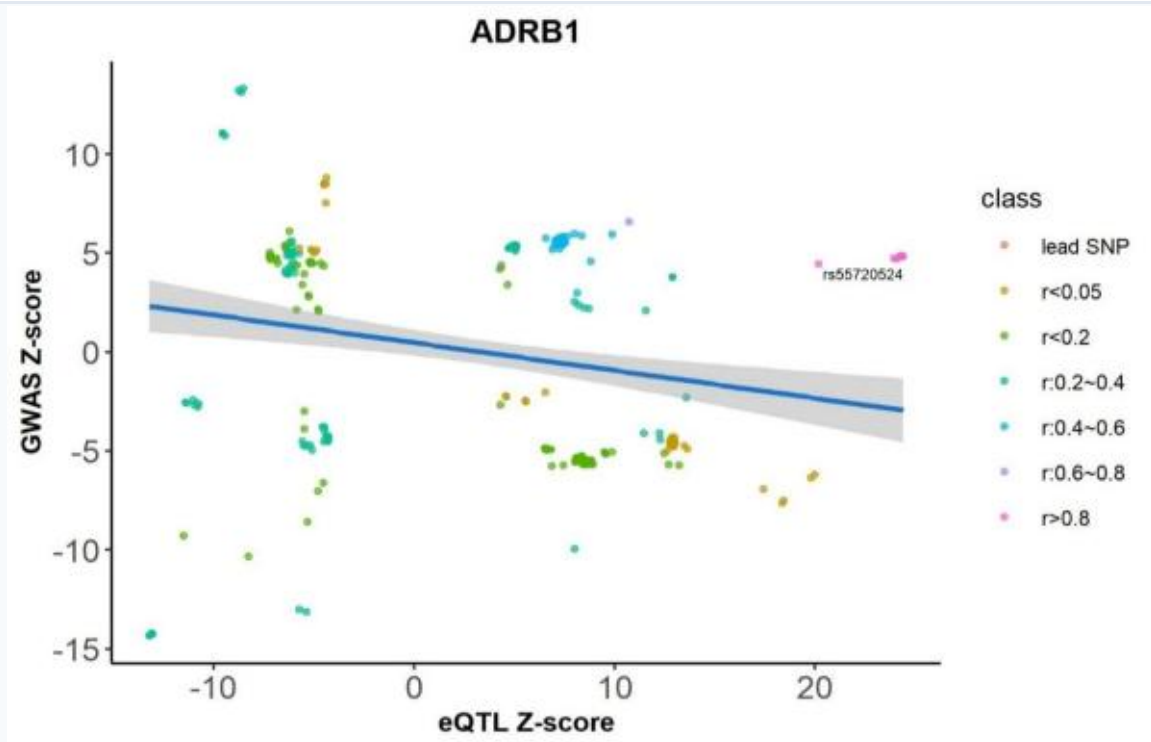

Before	After

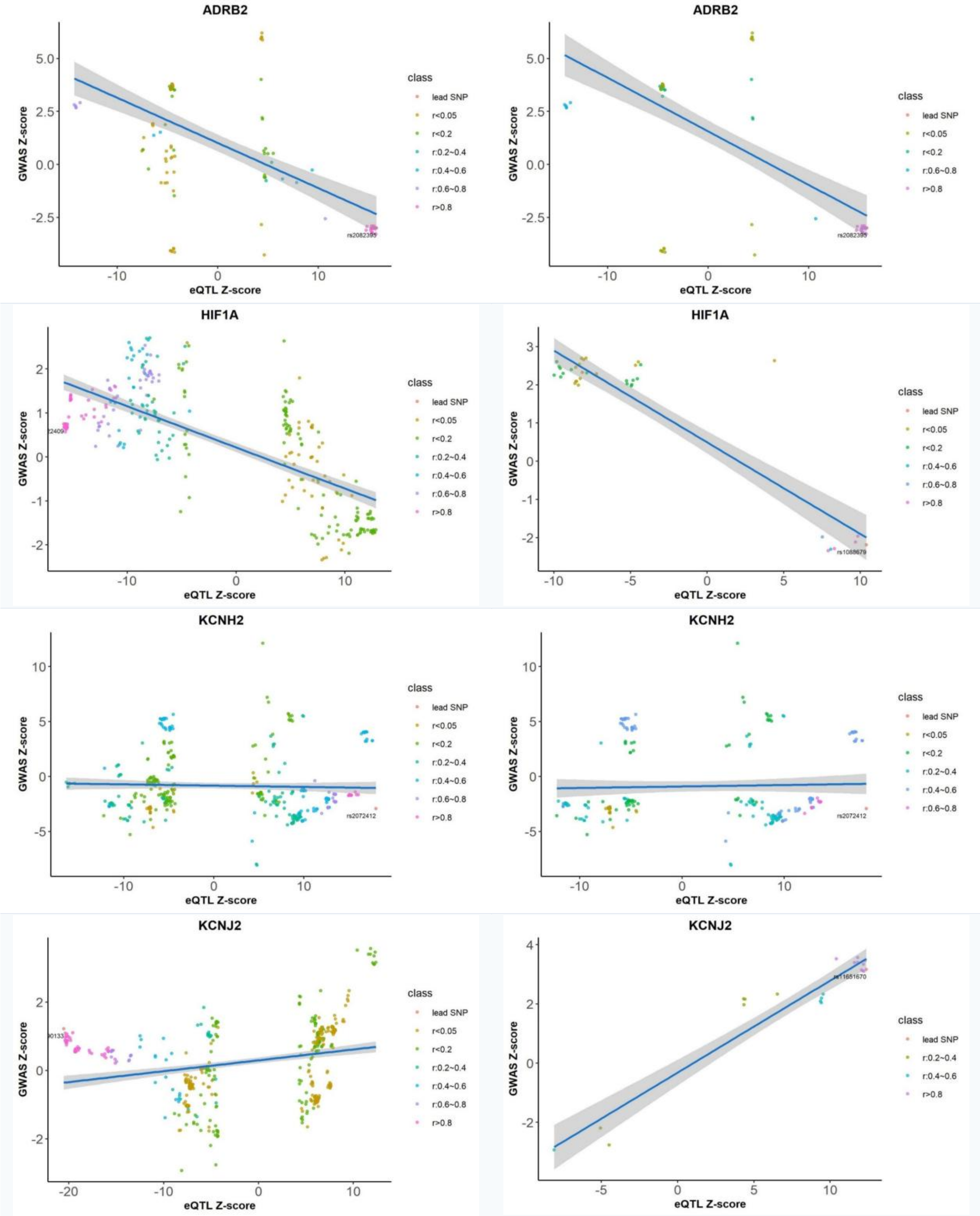

Before	After

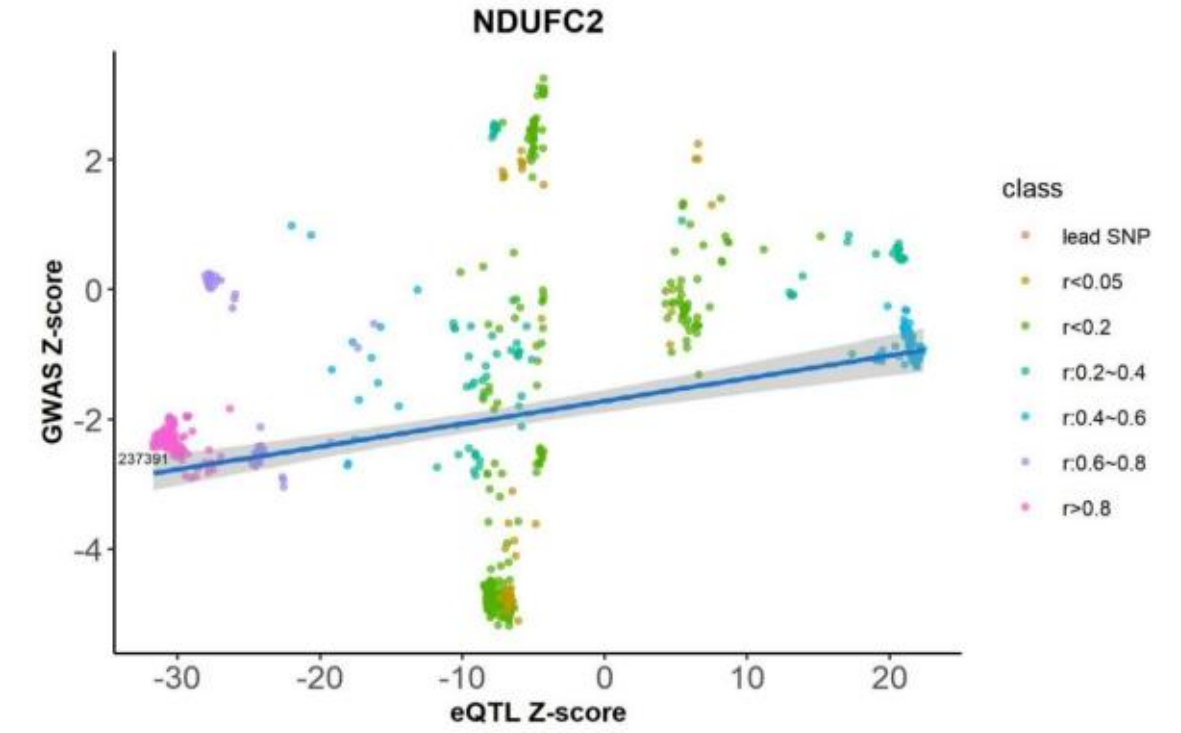

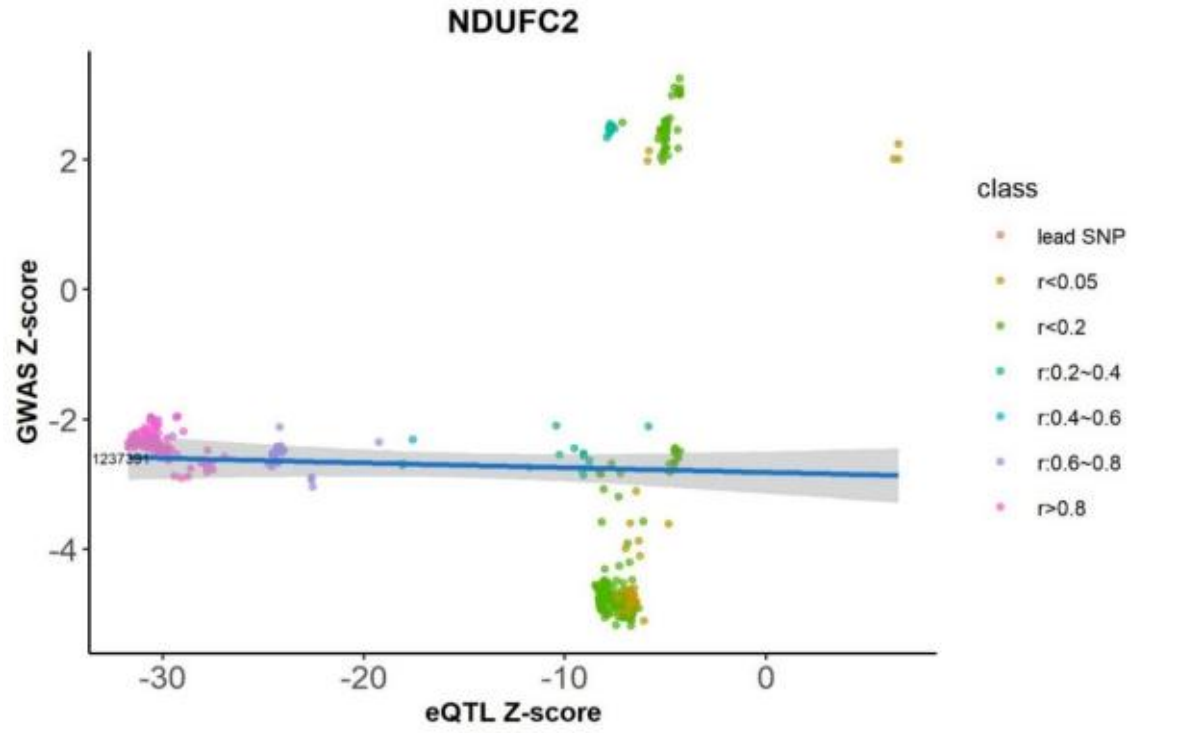

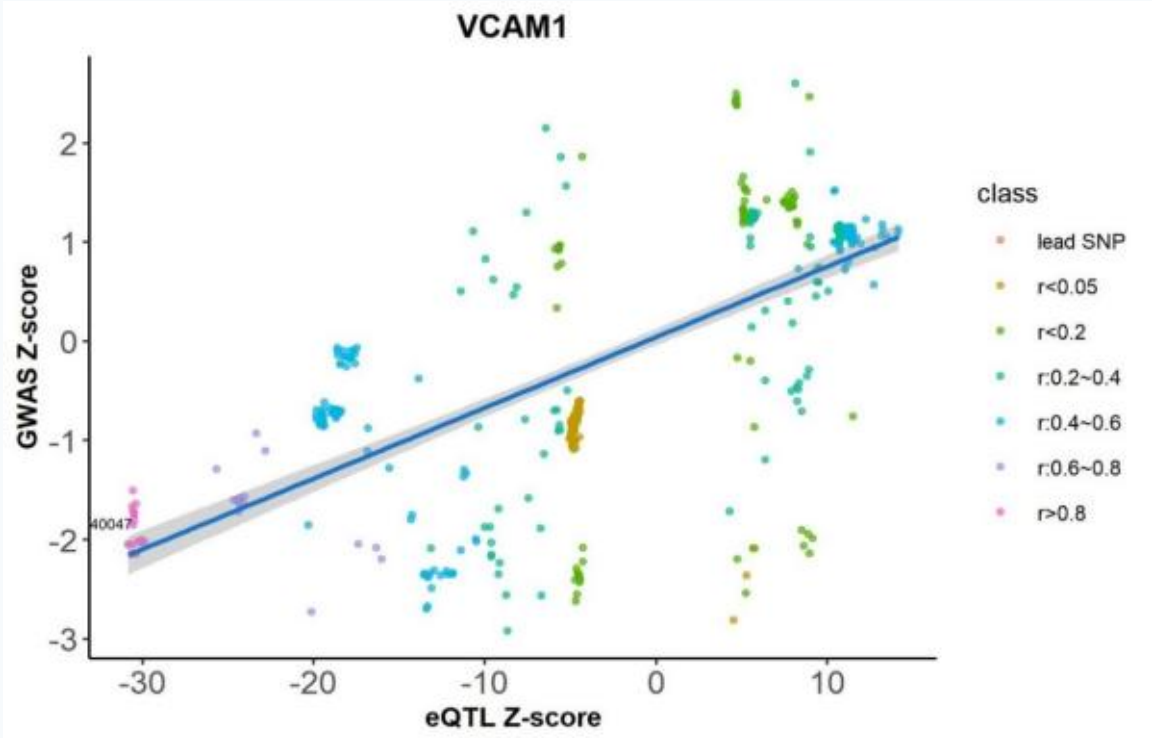

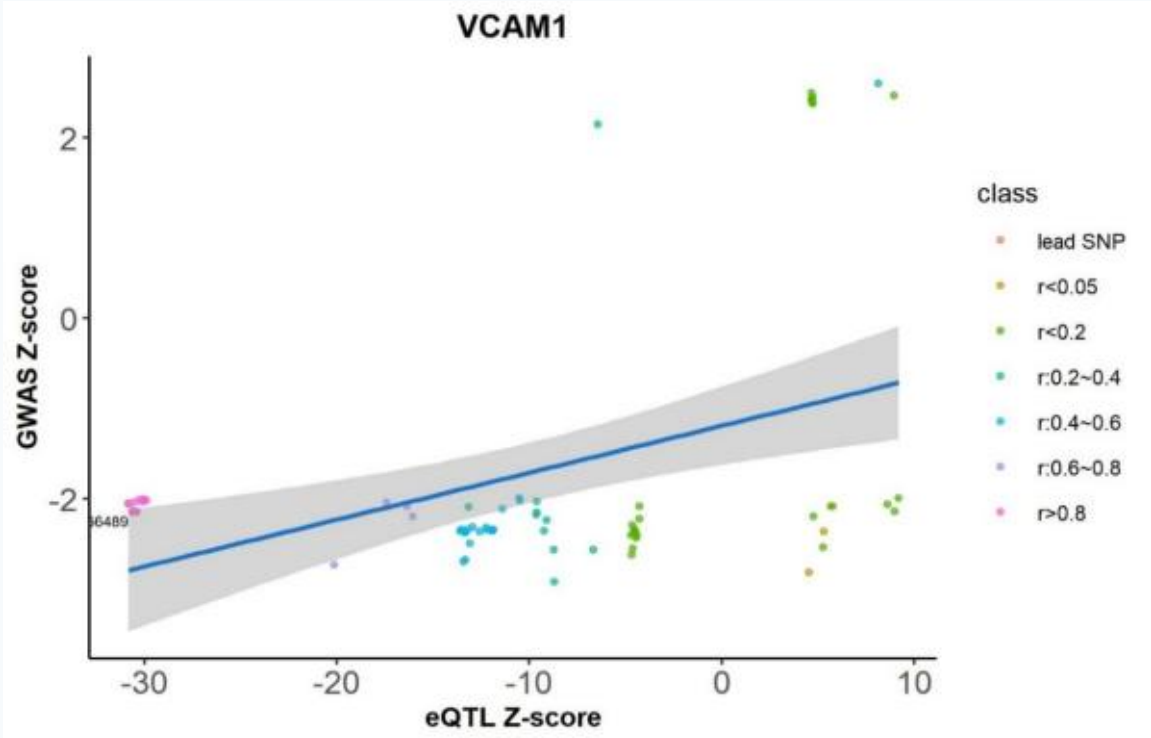

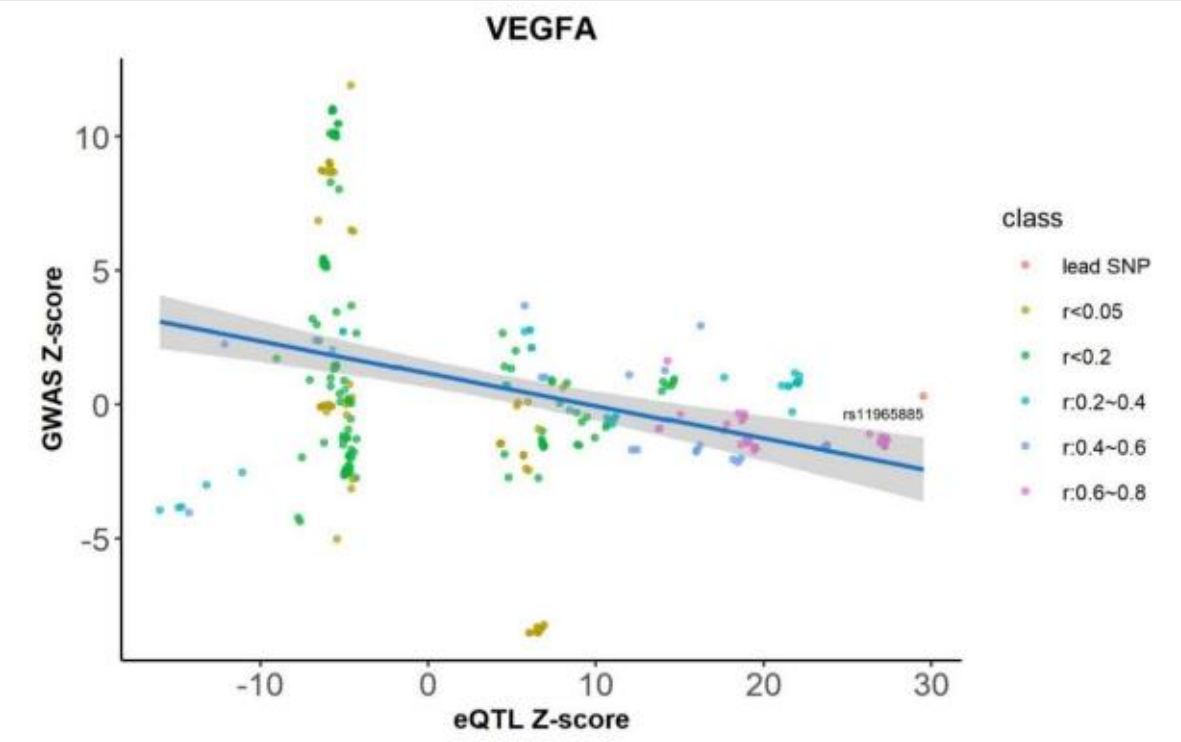

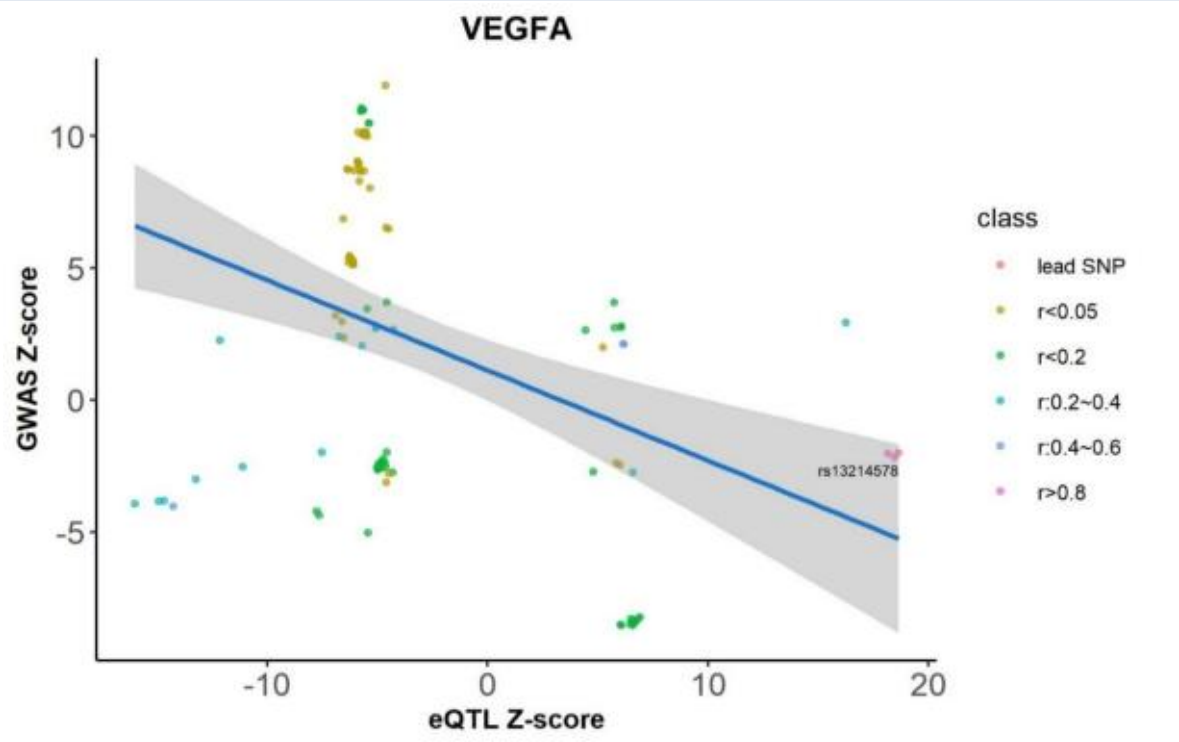

Calcium chanel blockers

Before	After

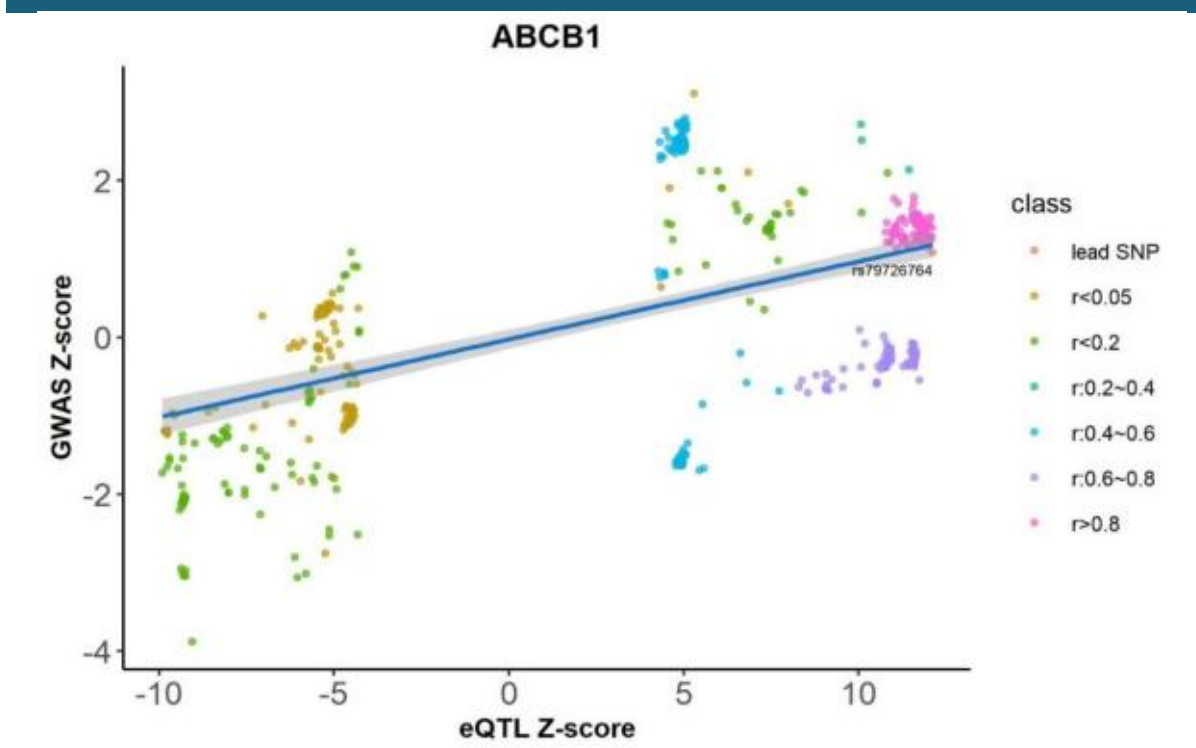

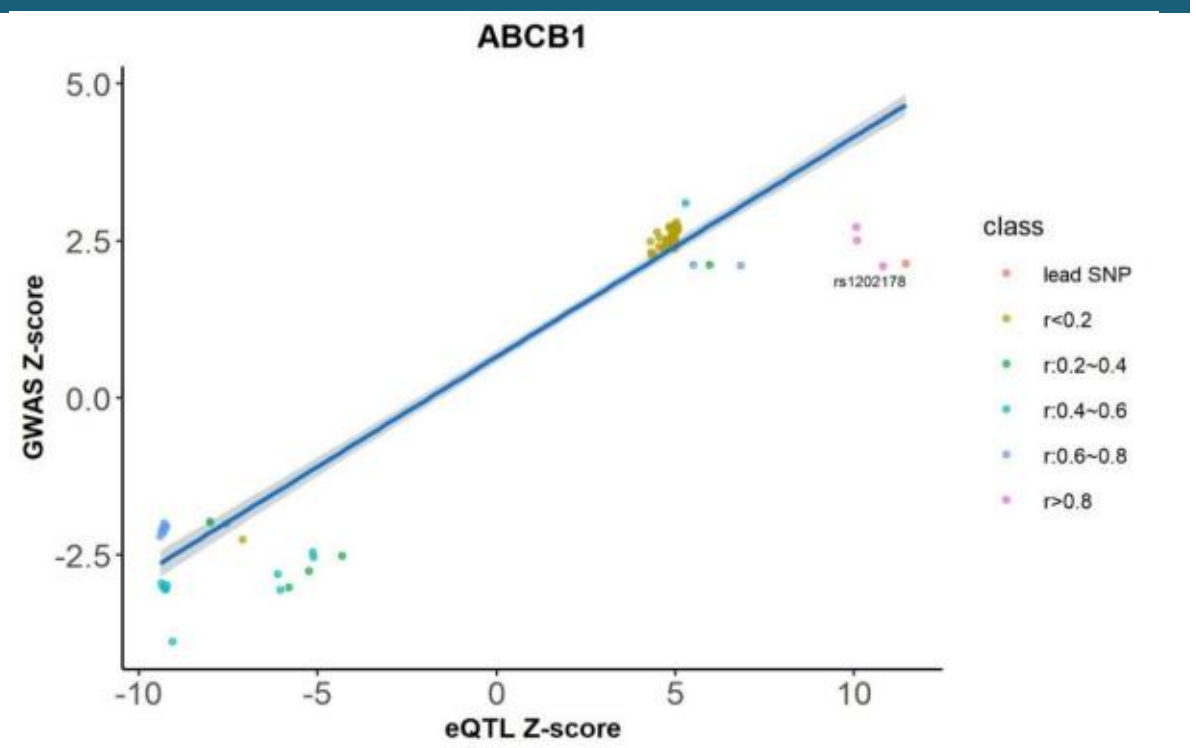

Before	After
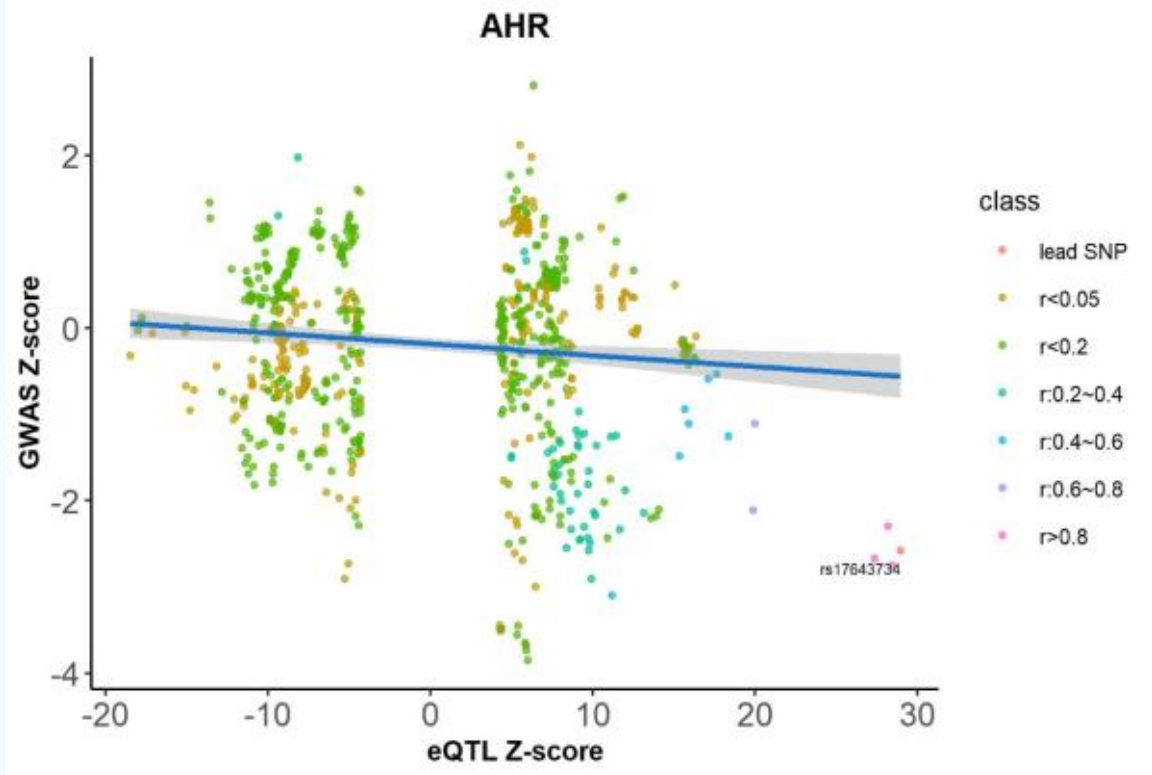	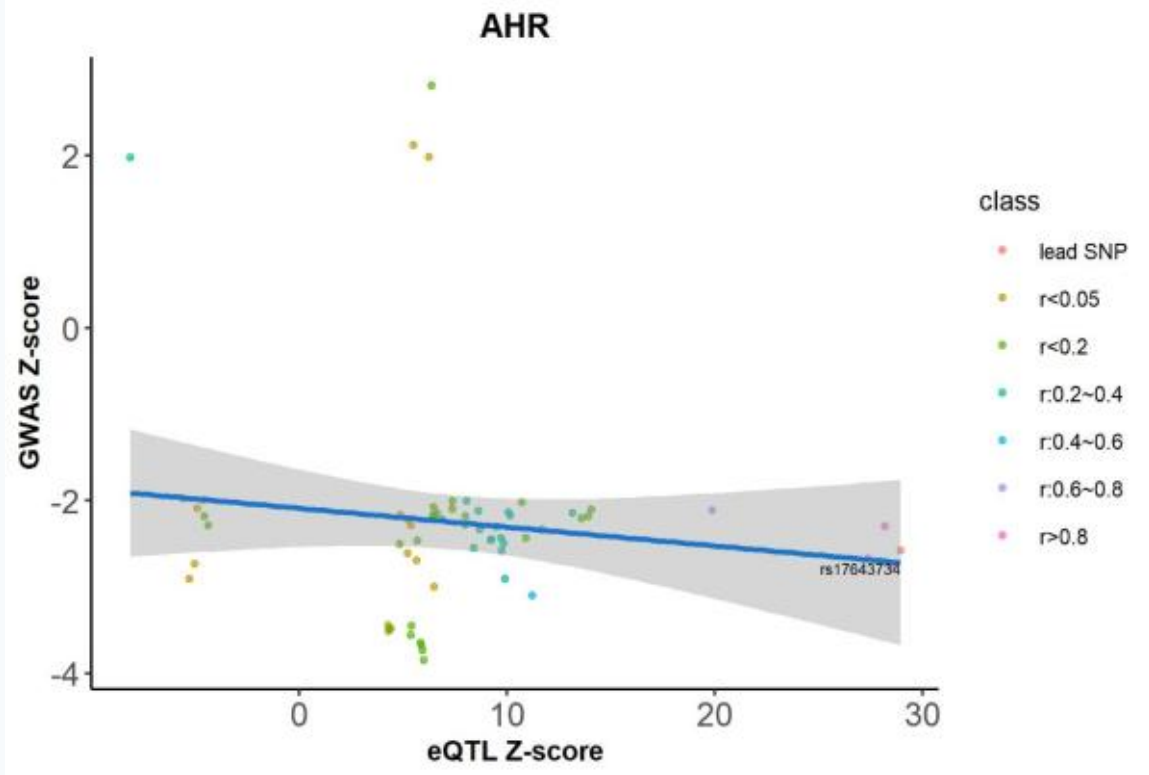
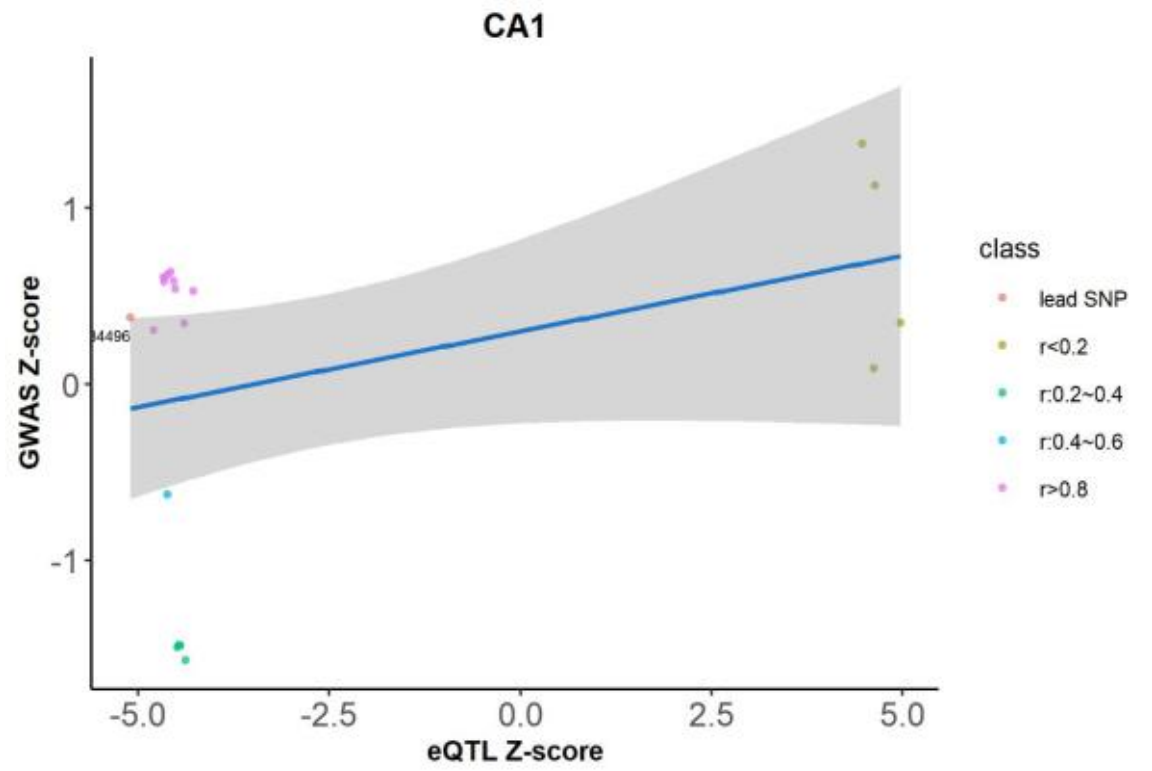	*No significant variant retrieved*
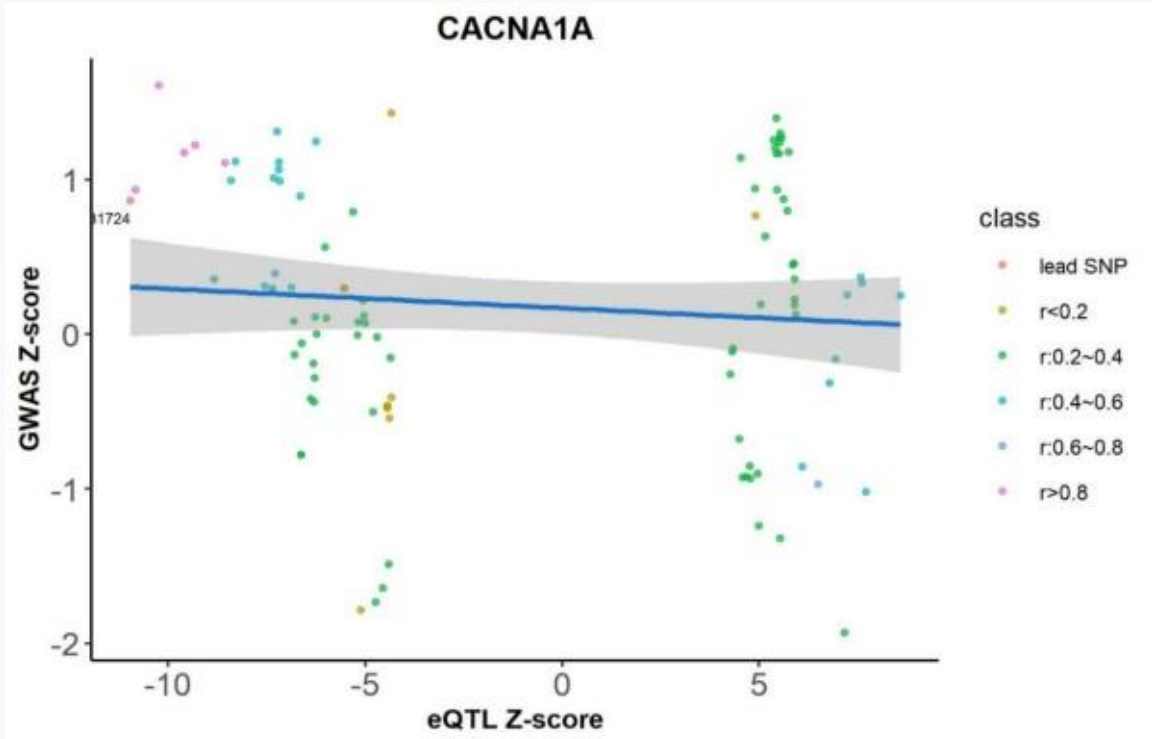	*No significant variant retrieved*
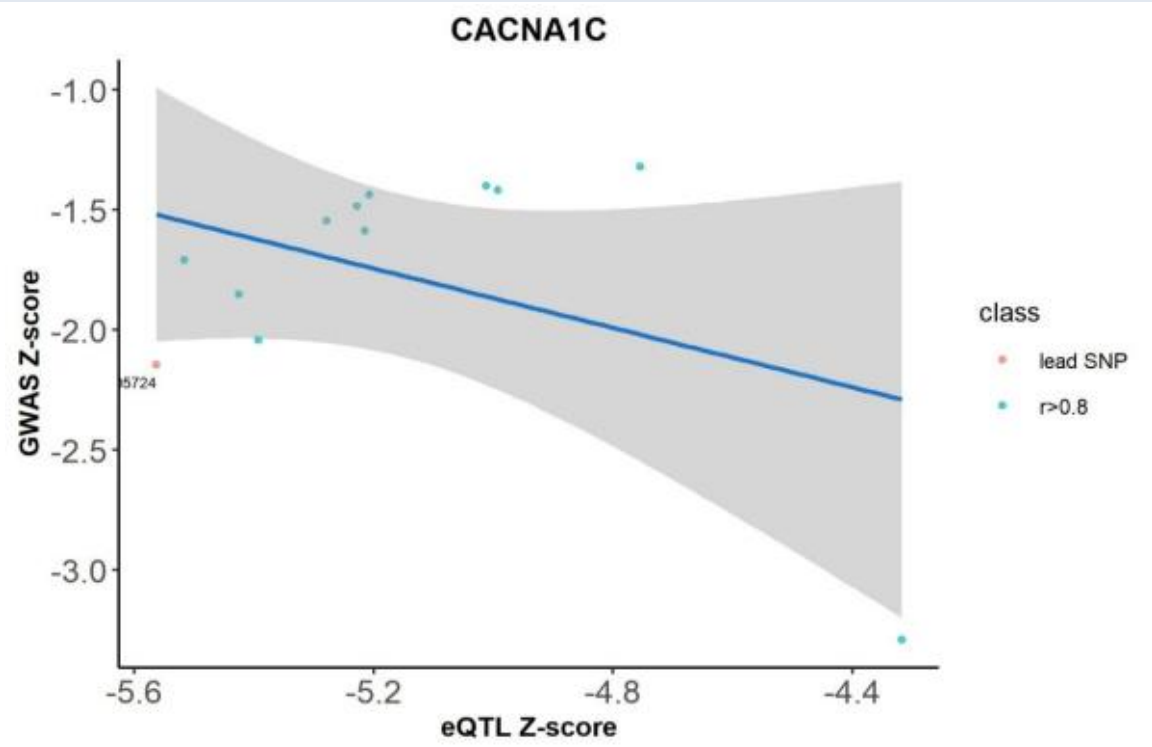	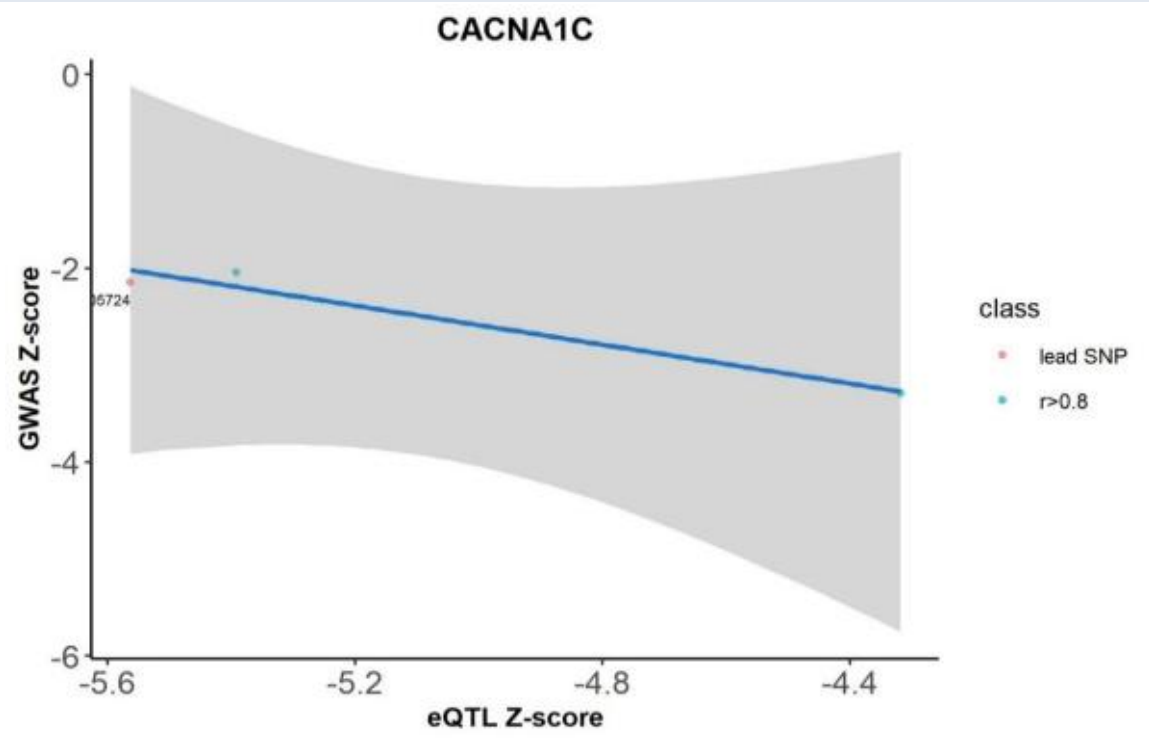

Before	After

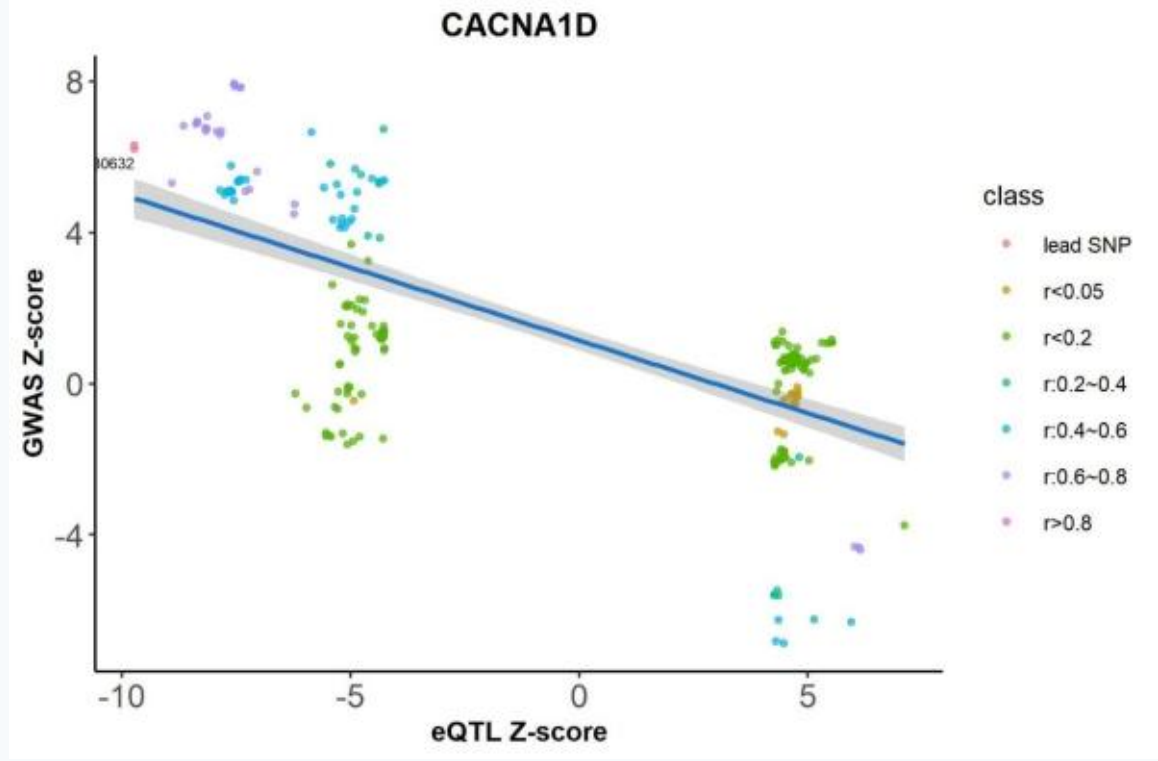

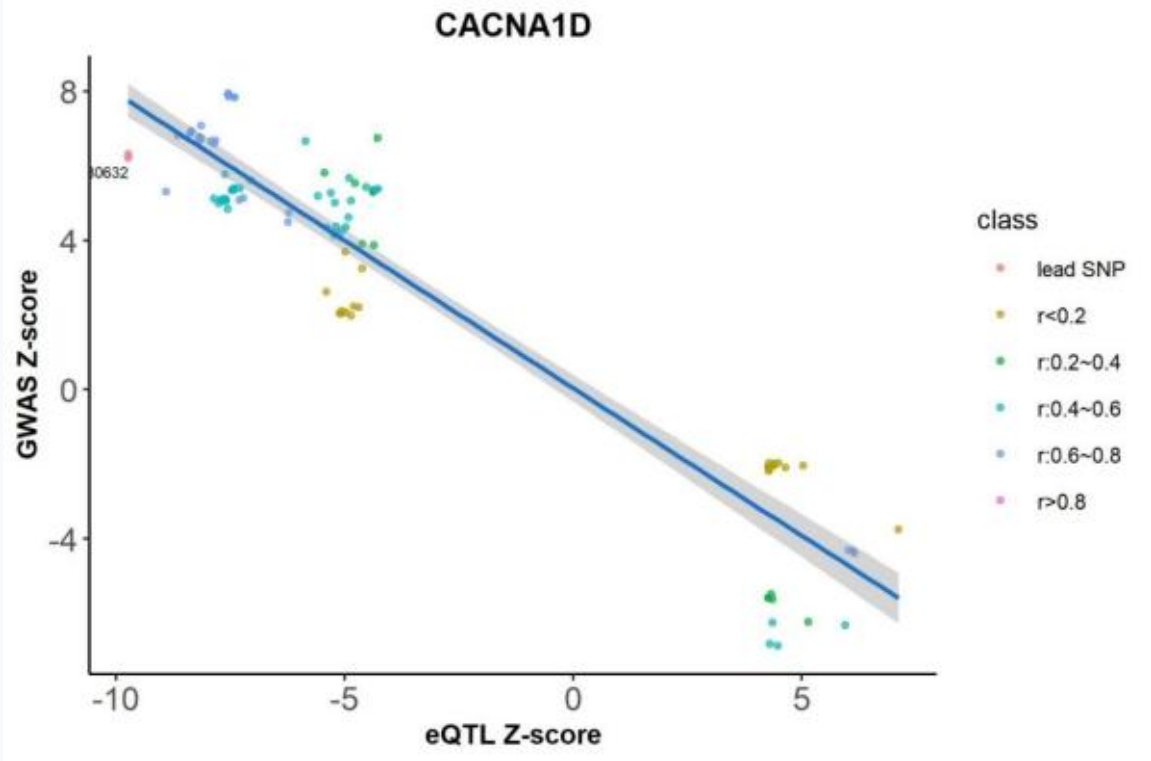

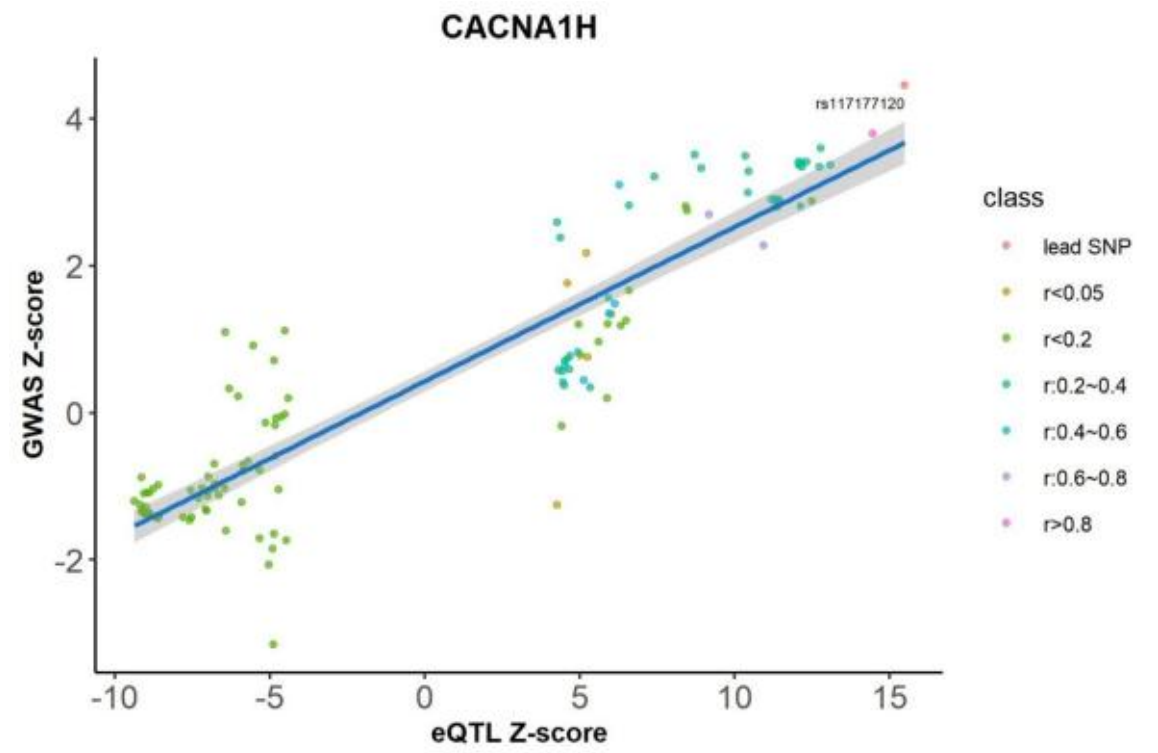

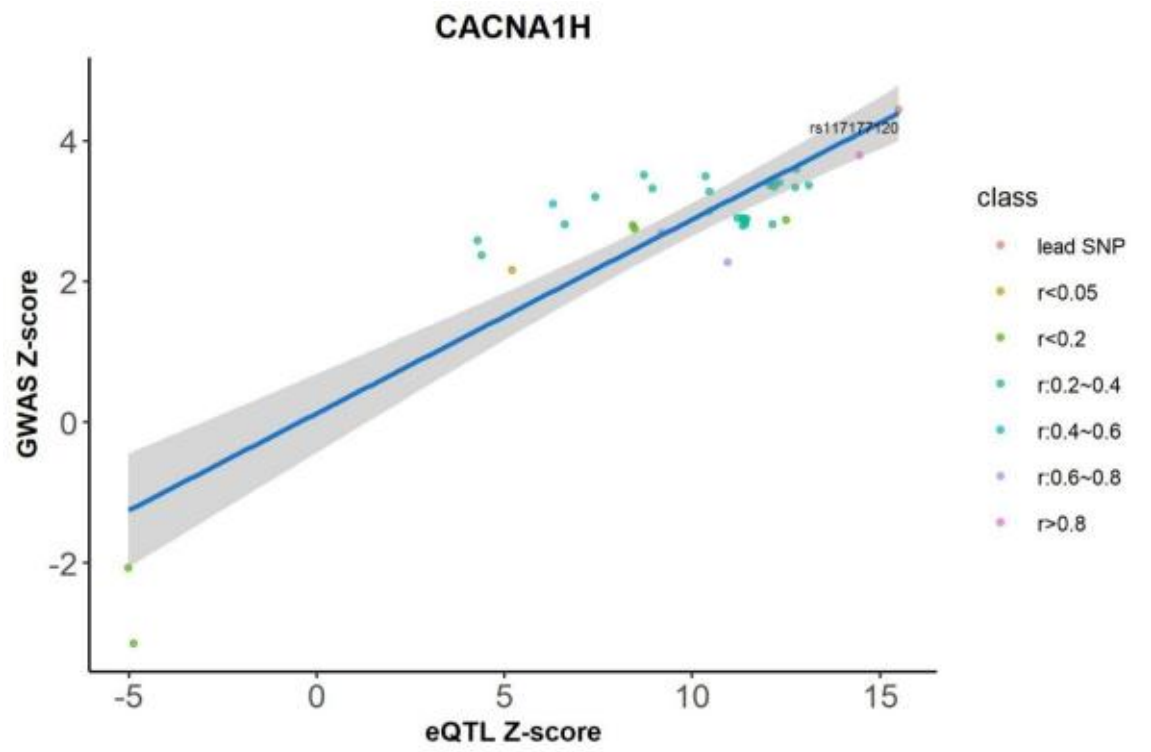

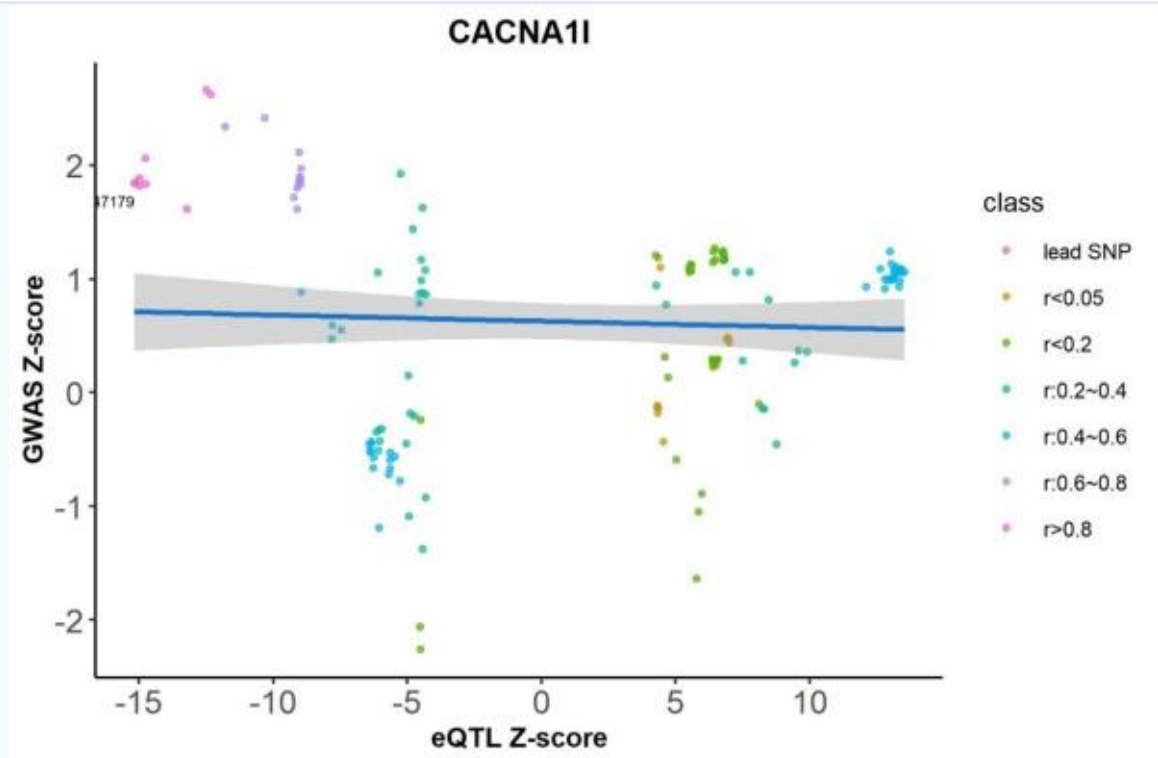

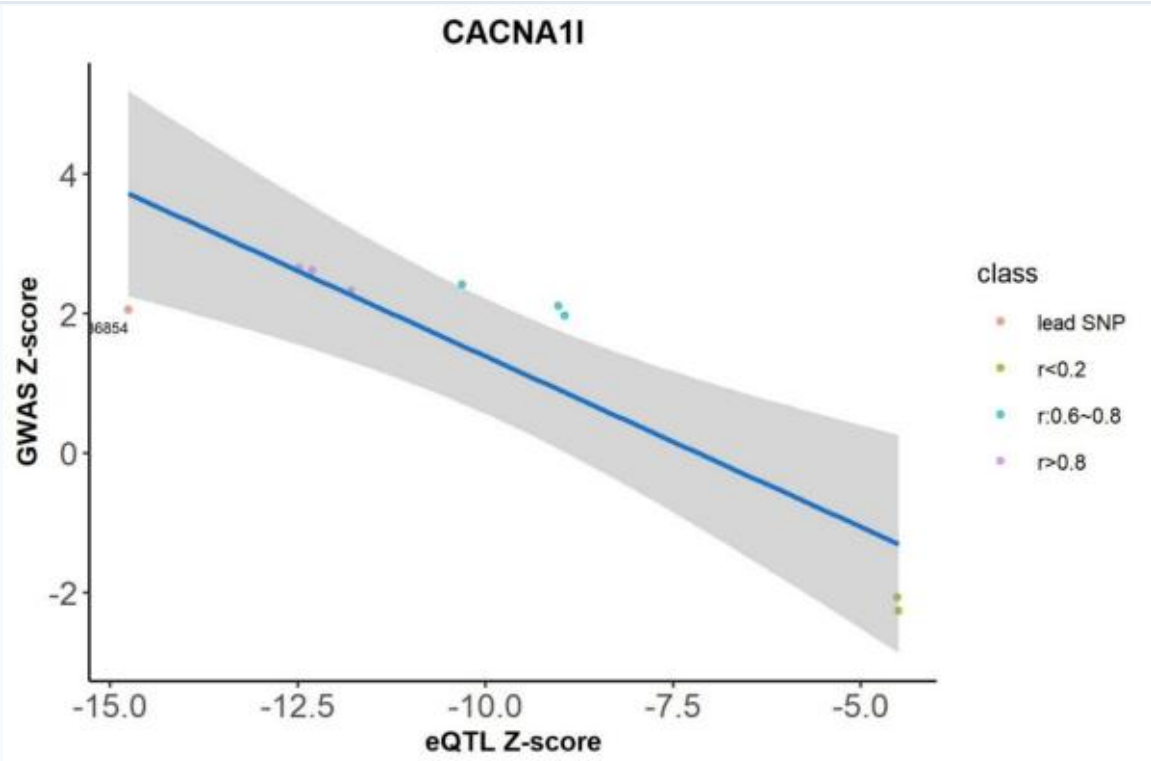

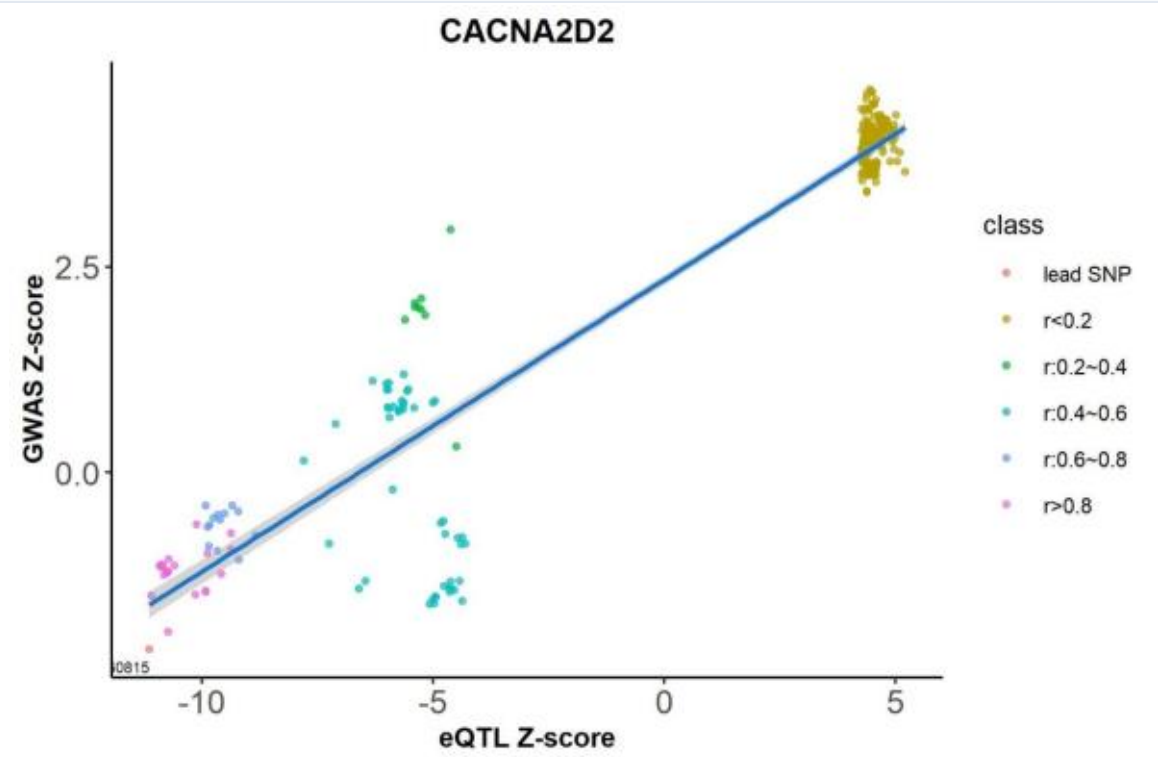

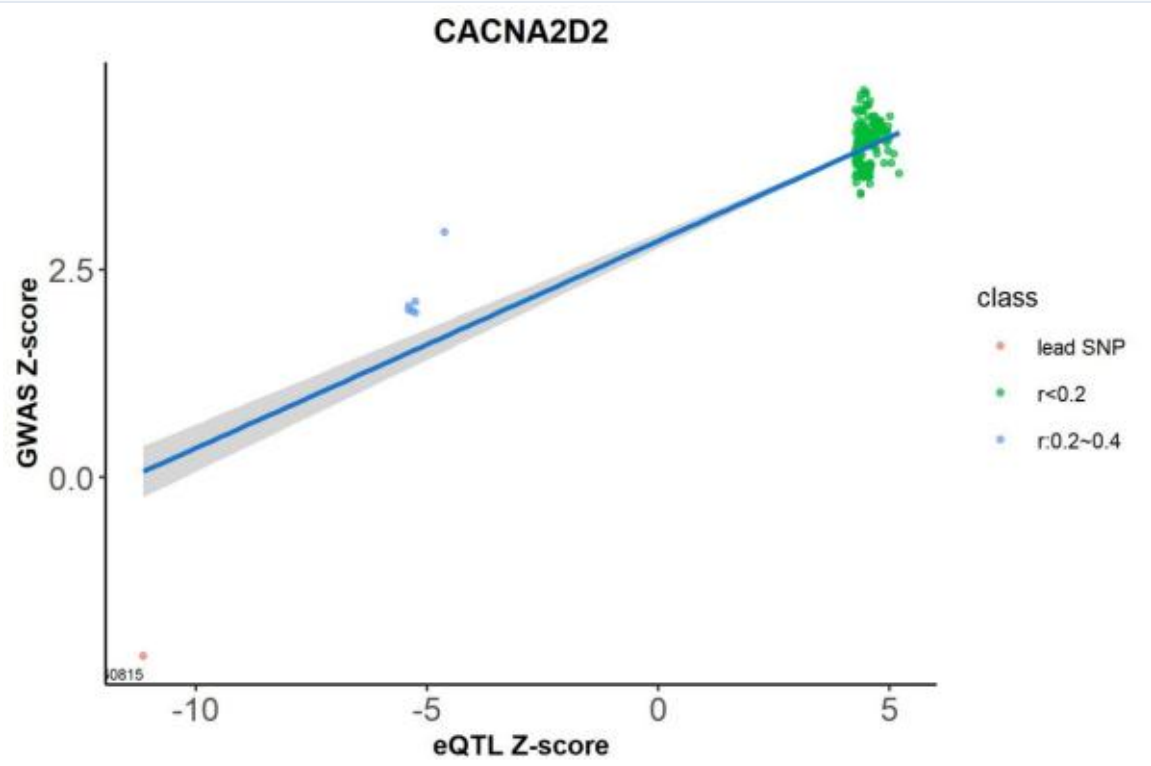

Before	After
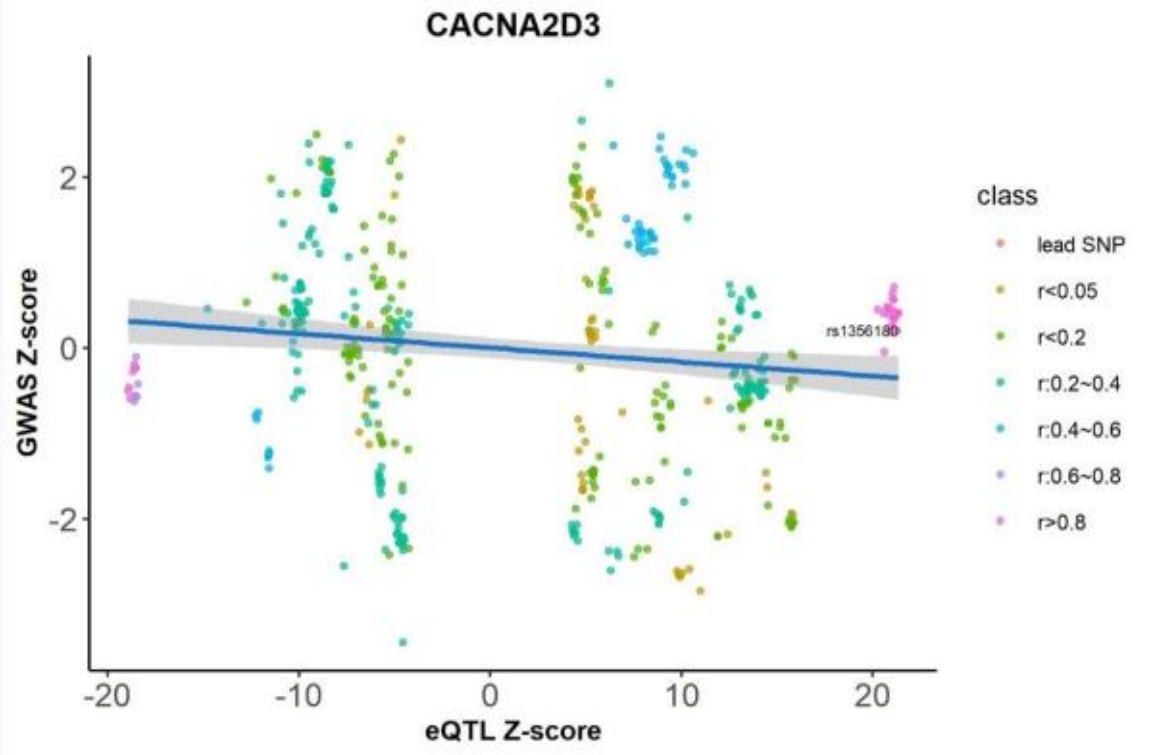	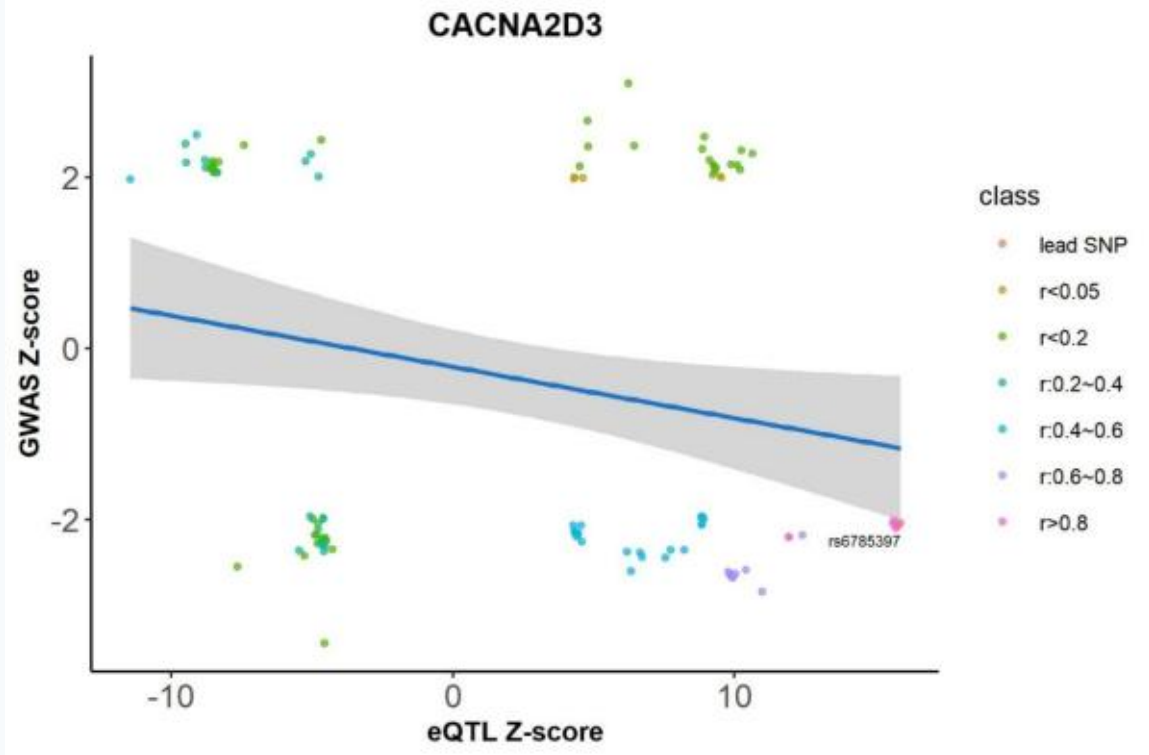
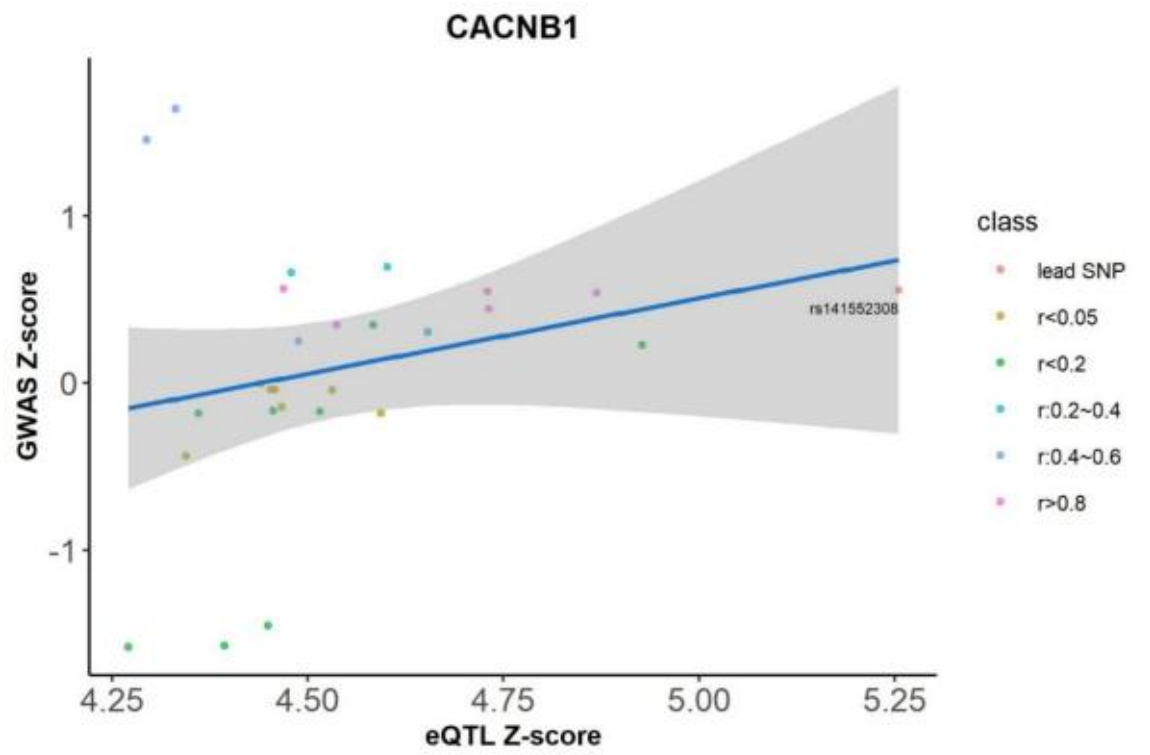	*No significant variant retrieved*
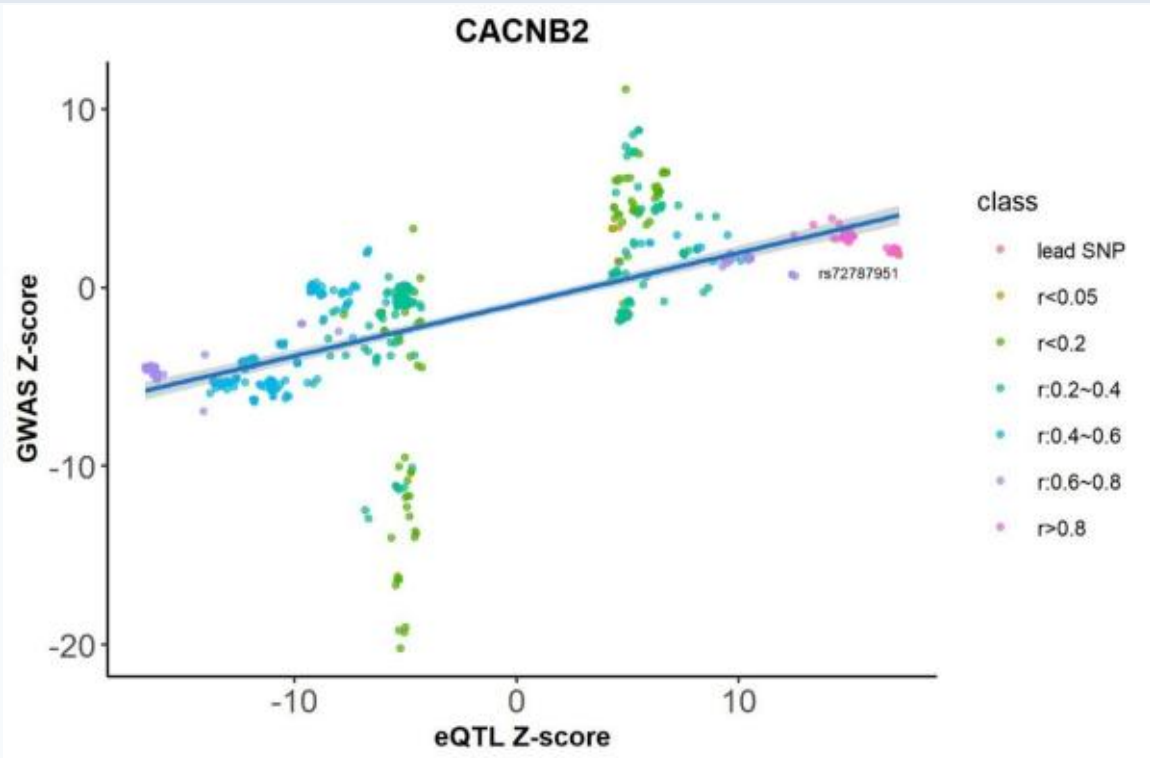	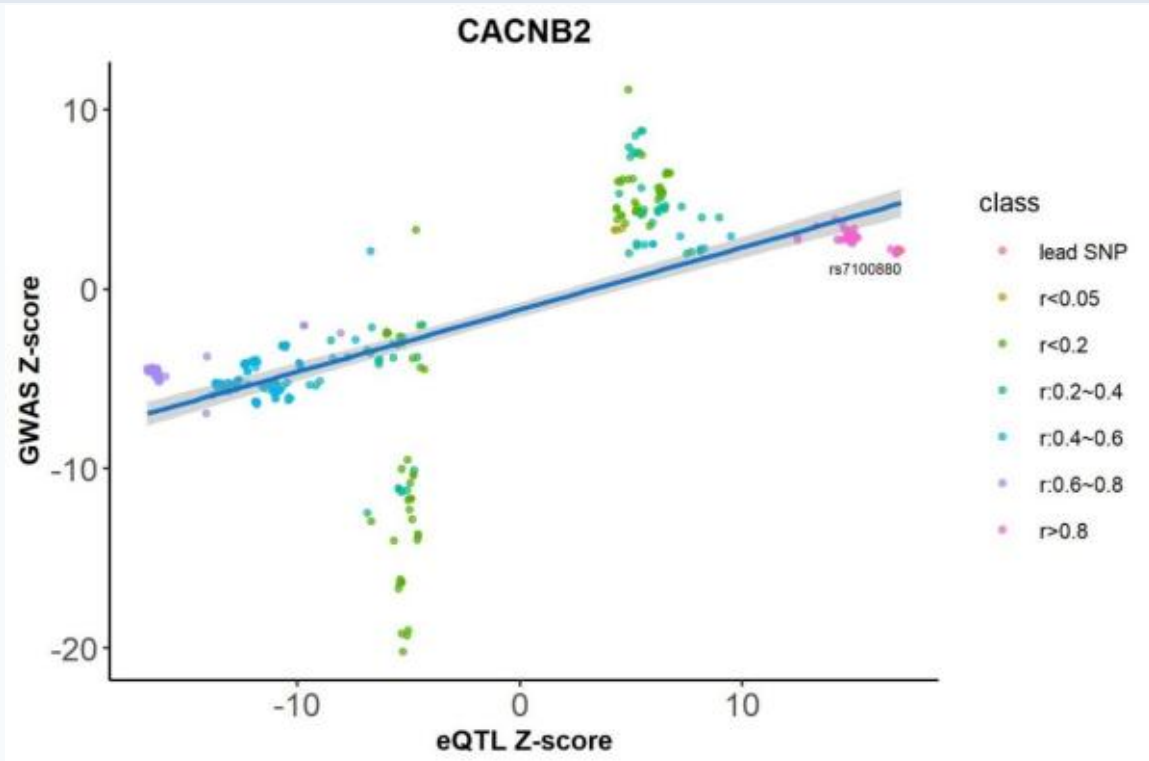
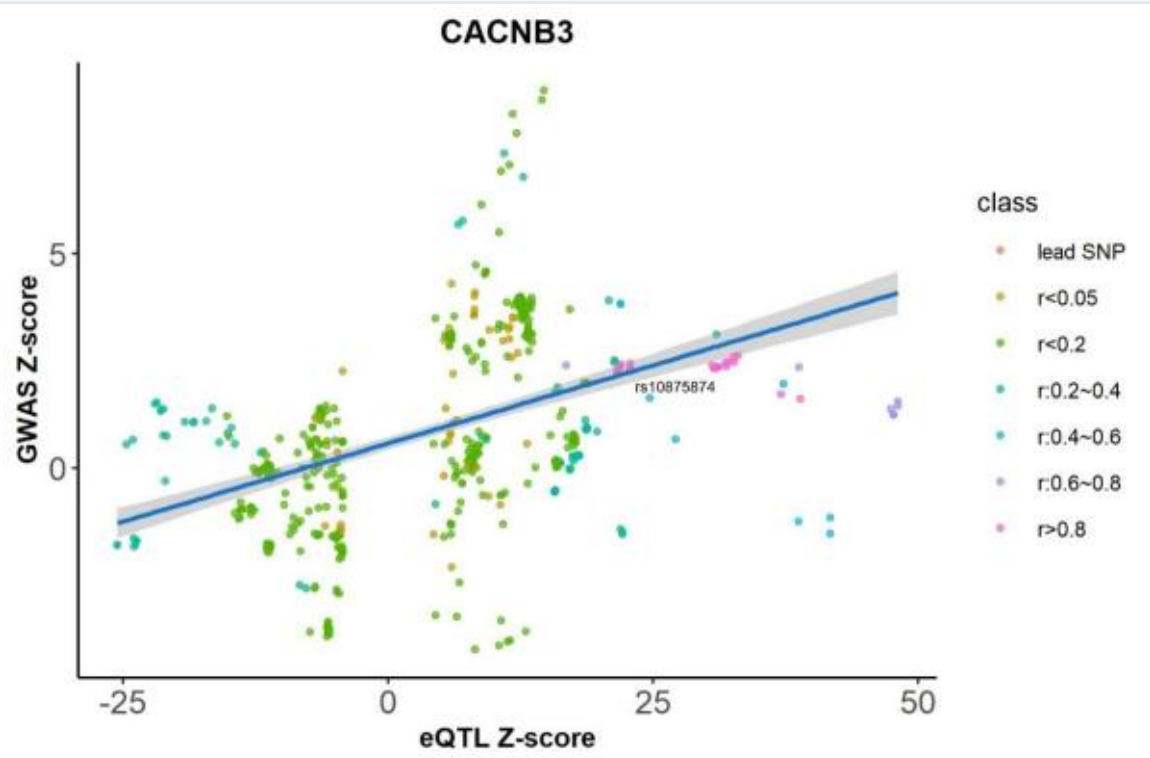	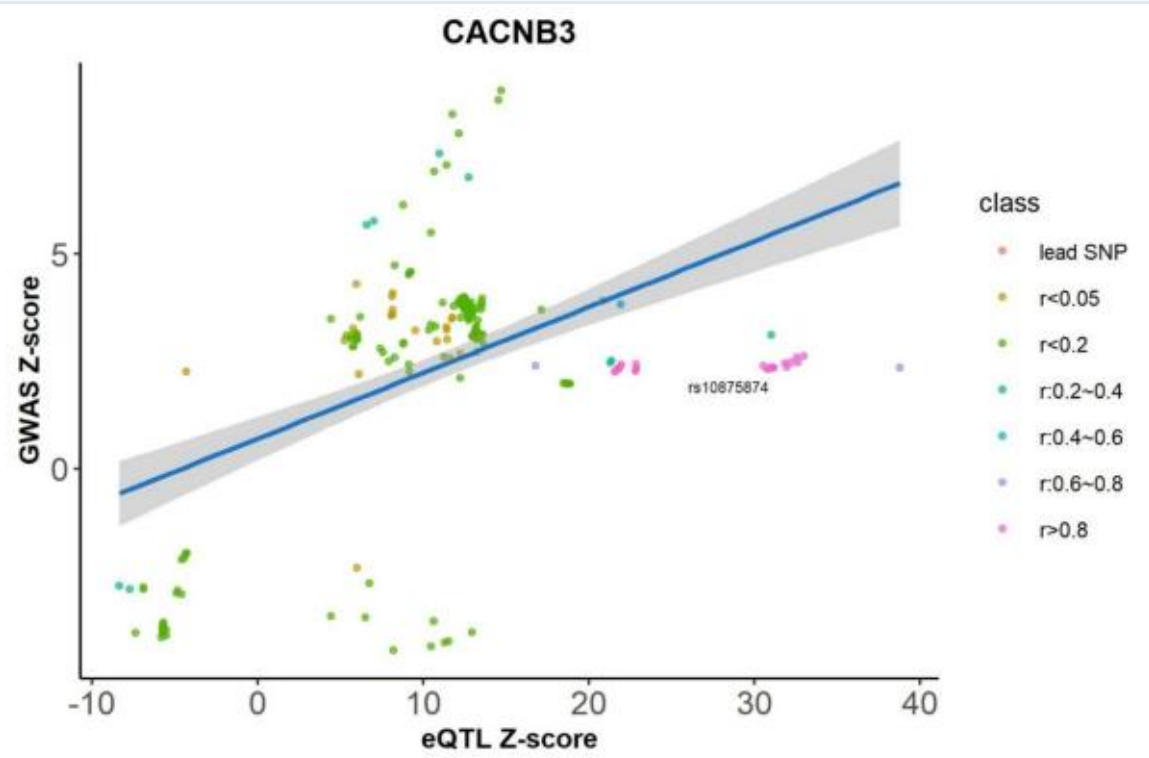

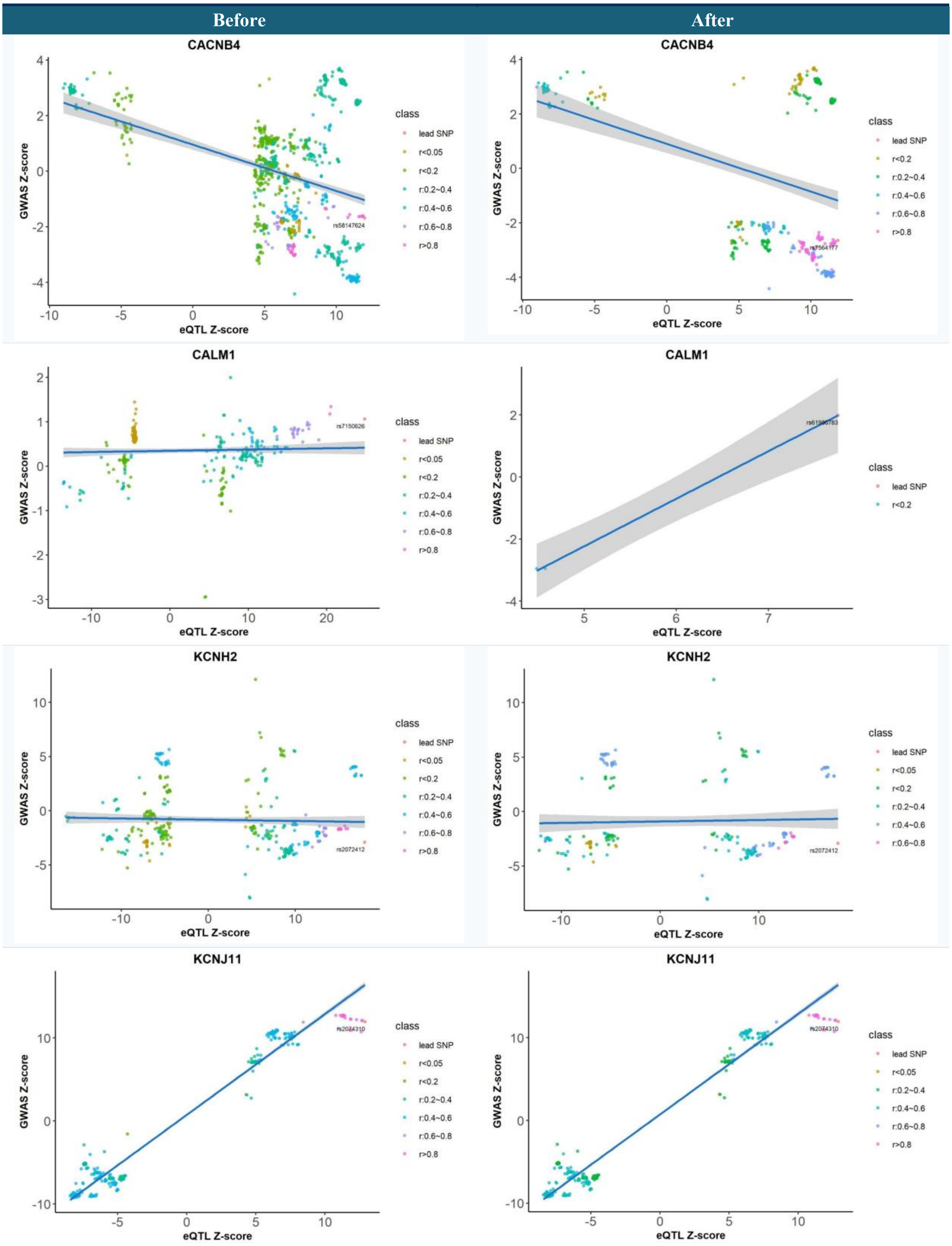
Before
After
CACNB4
CALM1
KCNH2
KCNJ11
GWAS Z-score
eQTL Z-score
class
lead SNP
r<0.05
r<0.2
r:0.2~0.4
r:0.4~0.6
r:0.6~0.8
r>0.8
rs58147624
rs7564177
rs7150626
rs2072412
rs2074310

Before	After

Before

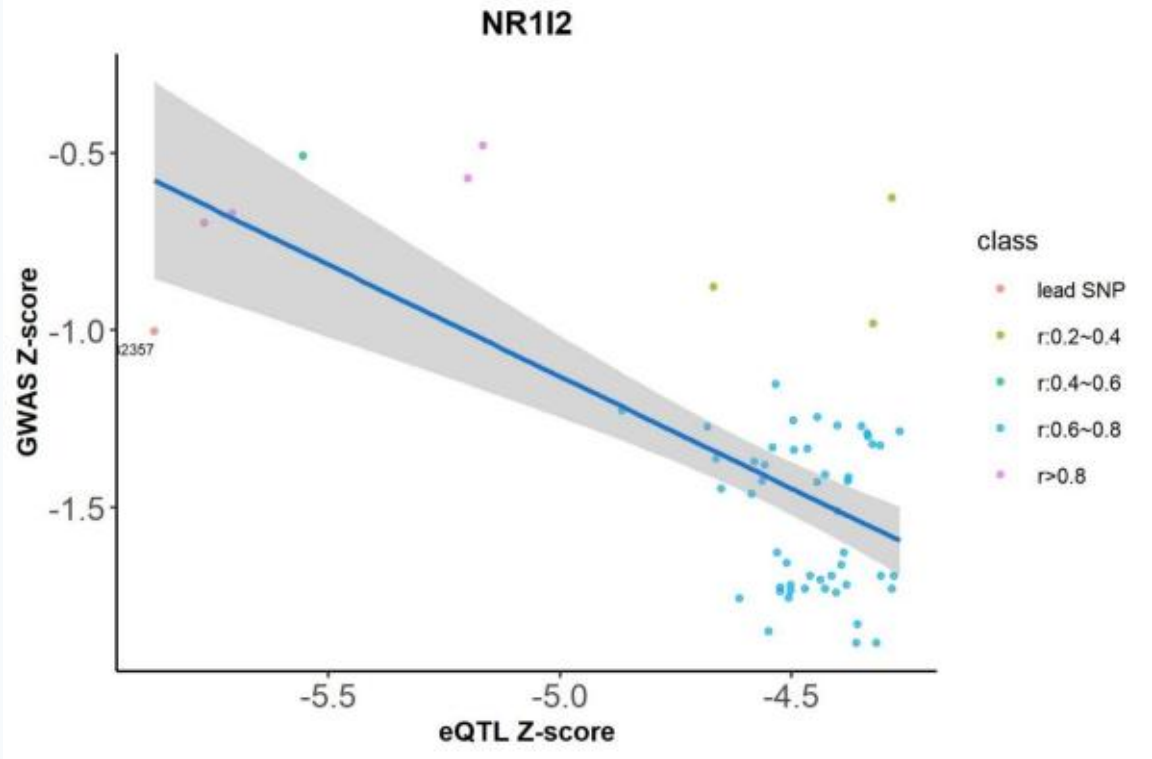

After

No significant variant retrieved

Before

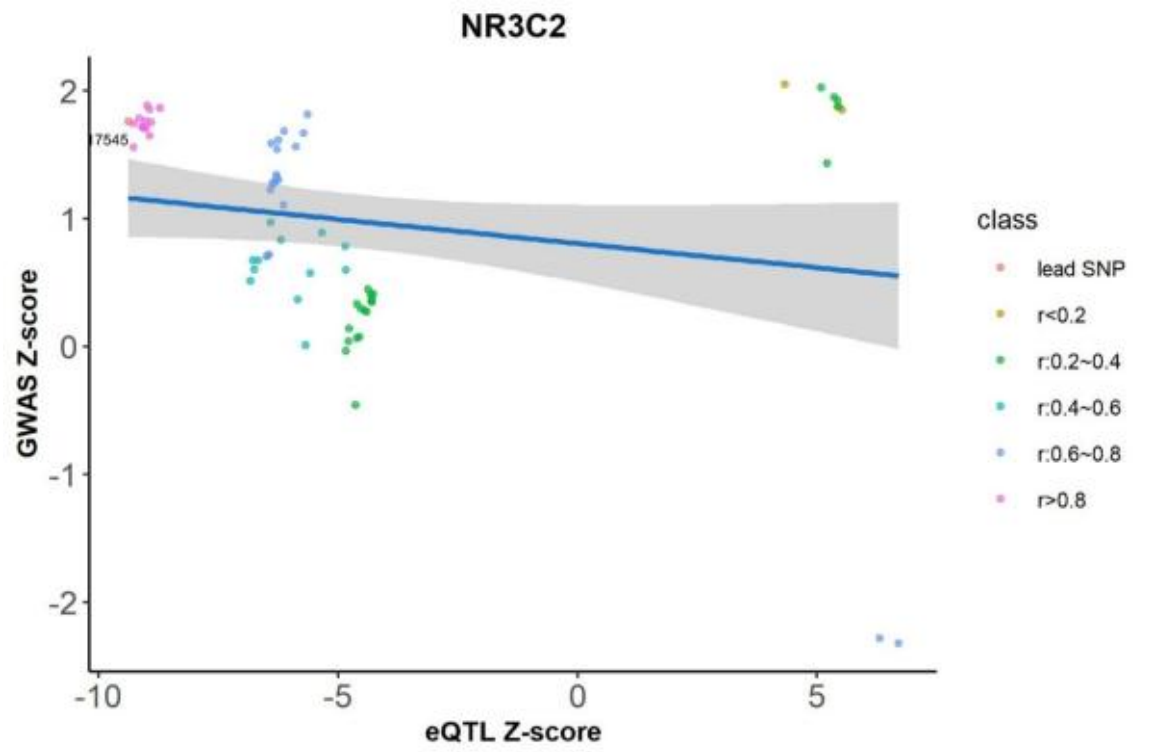

After

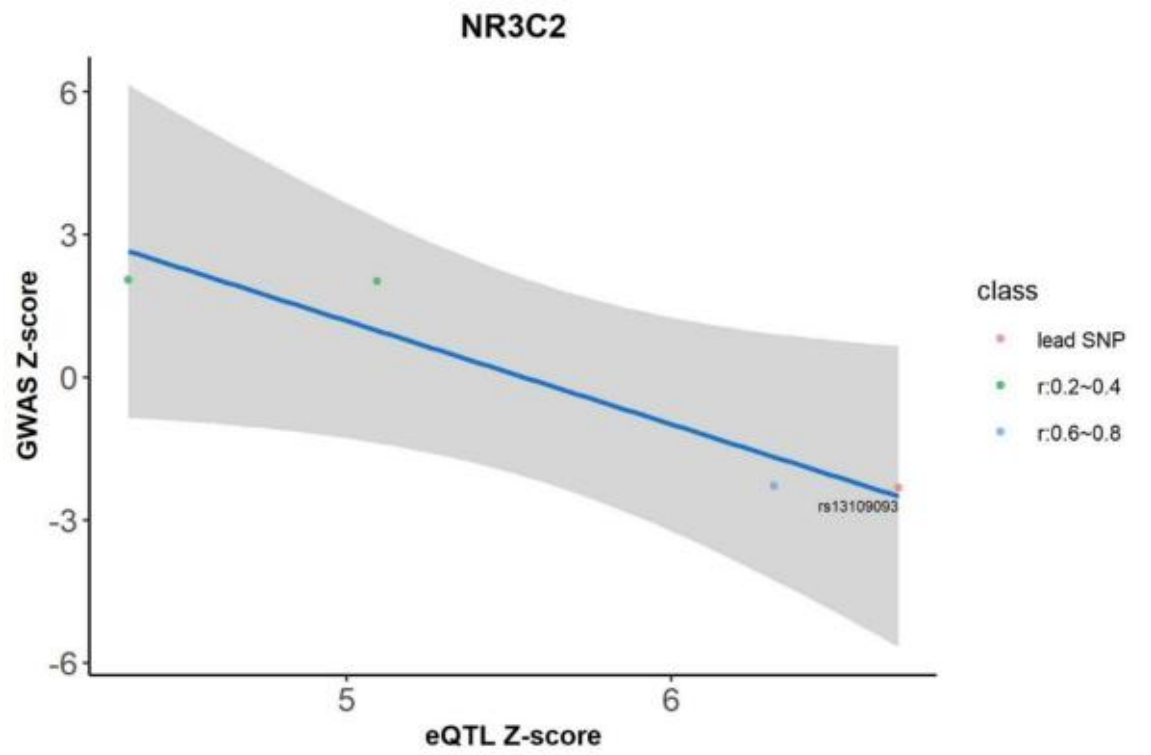

Before

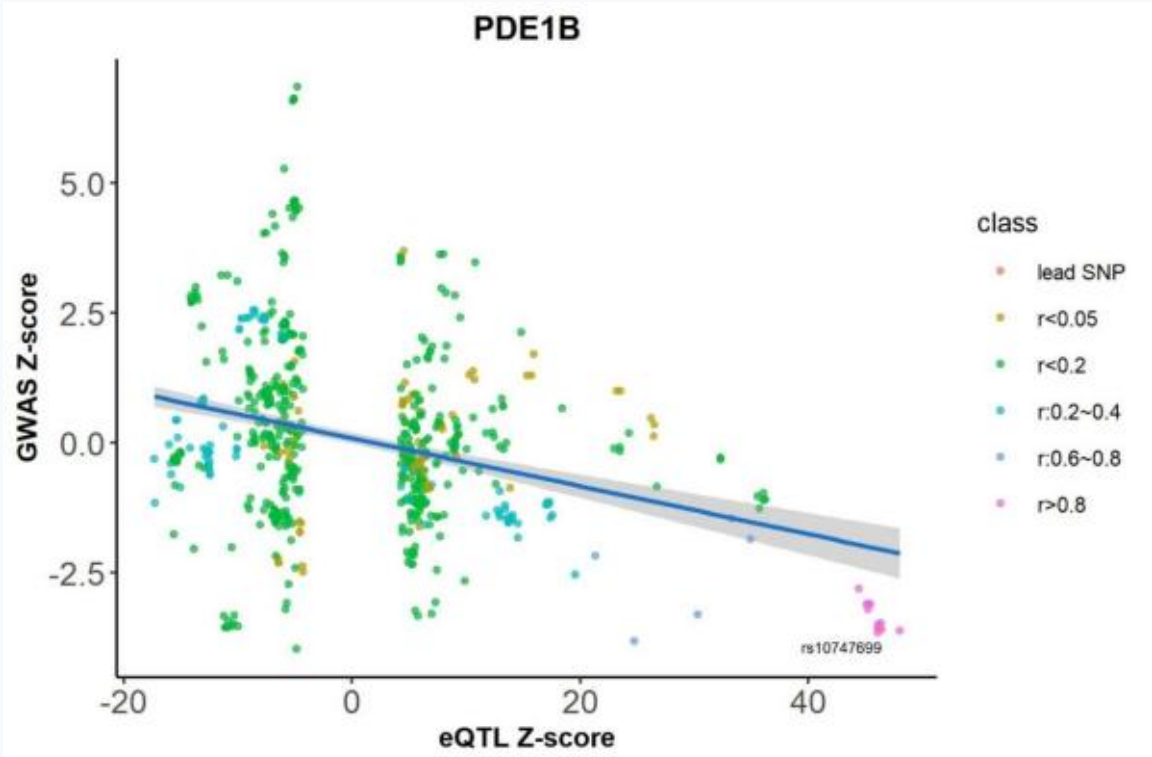

After

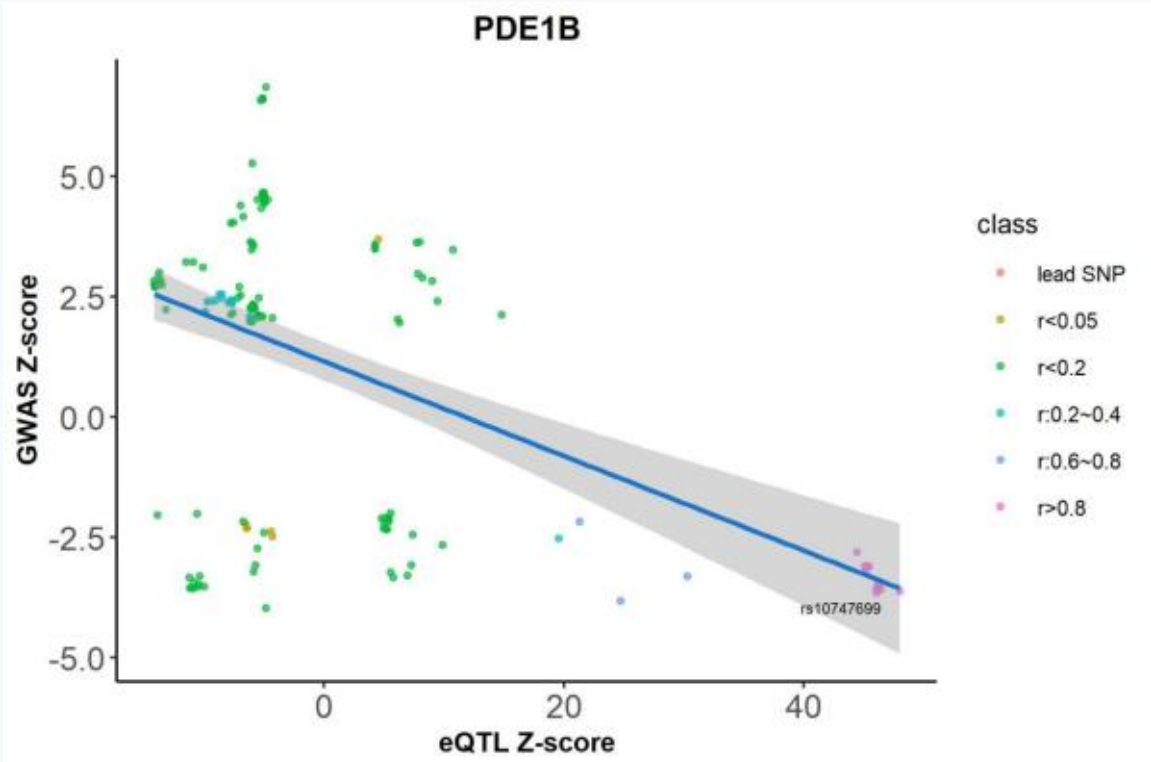

Before

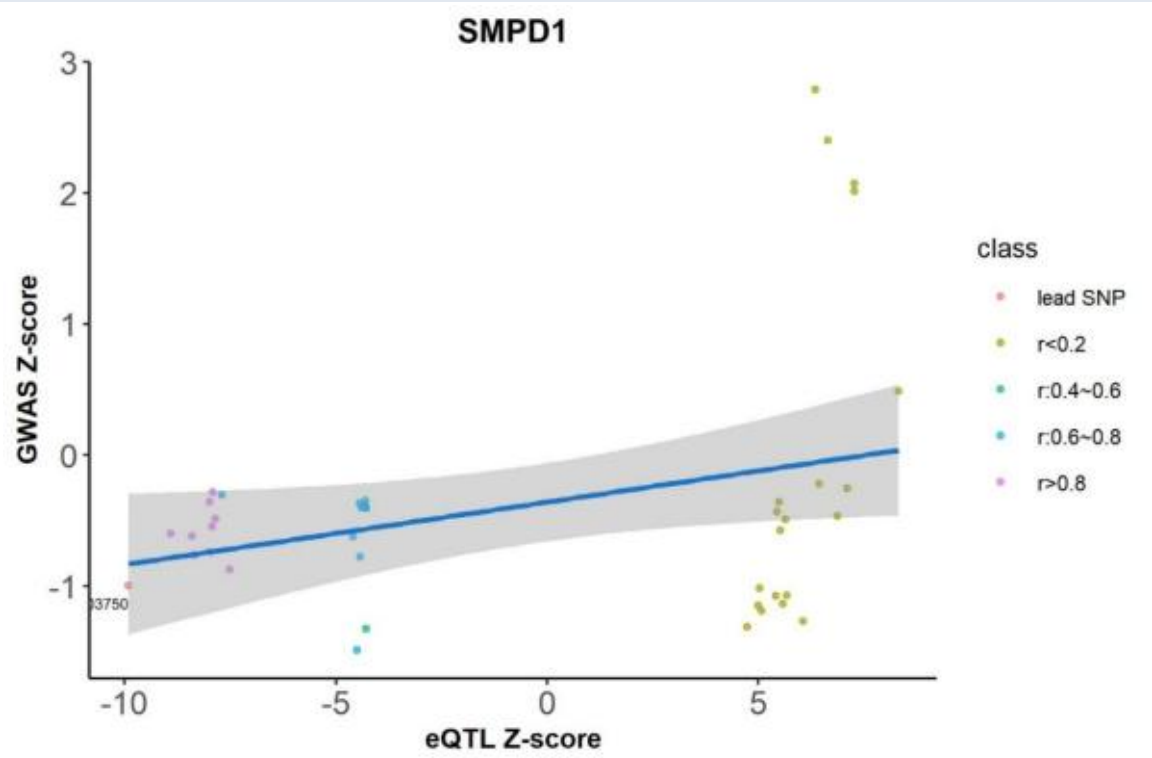

After

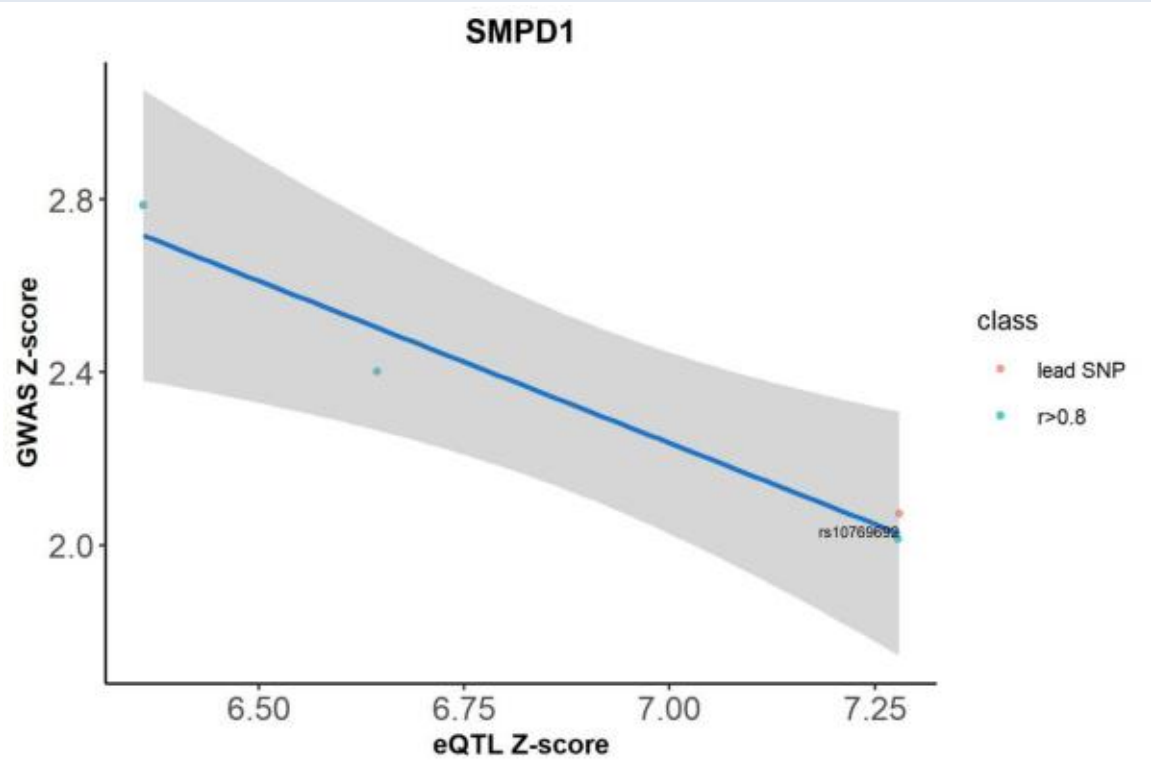

Before | **After**

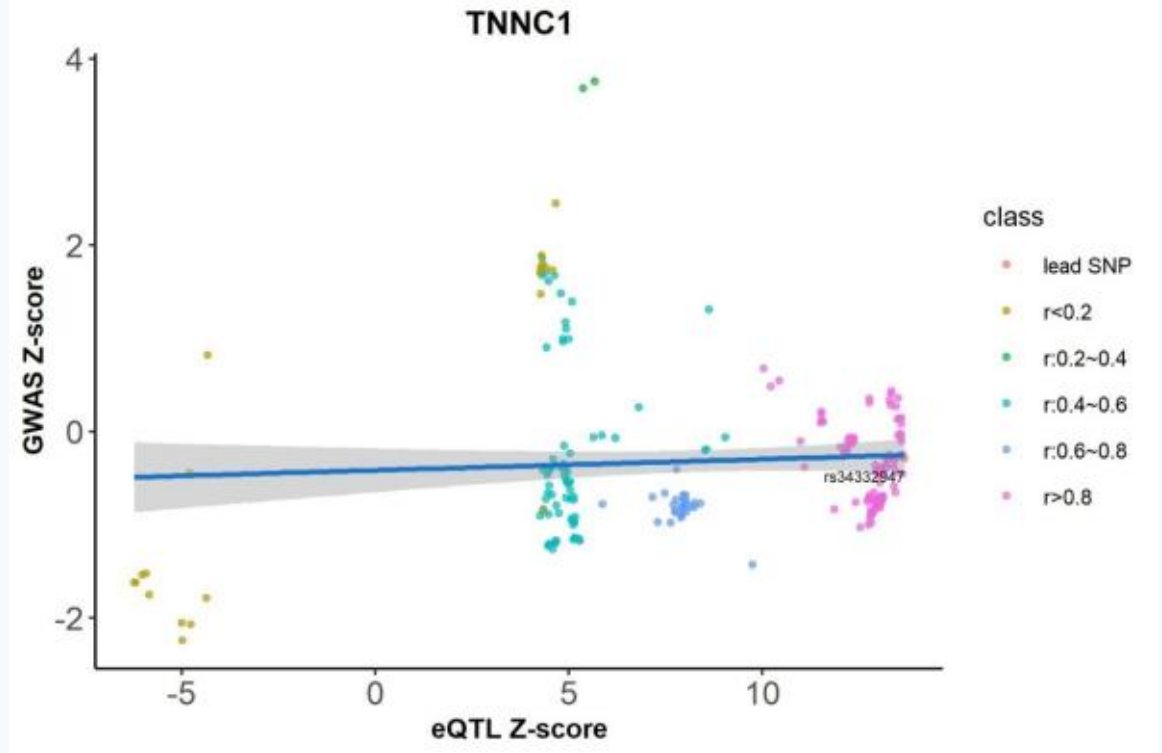

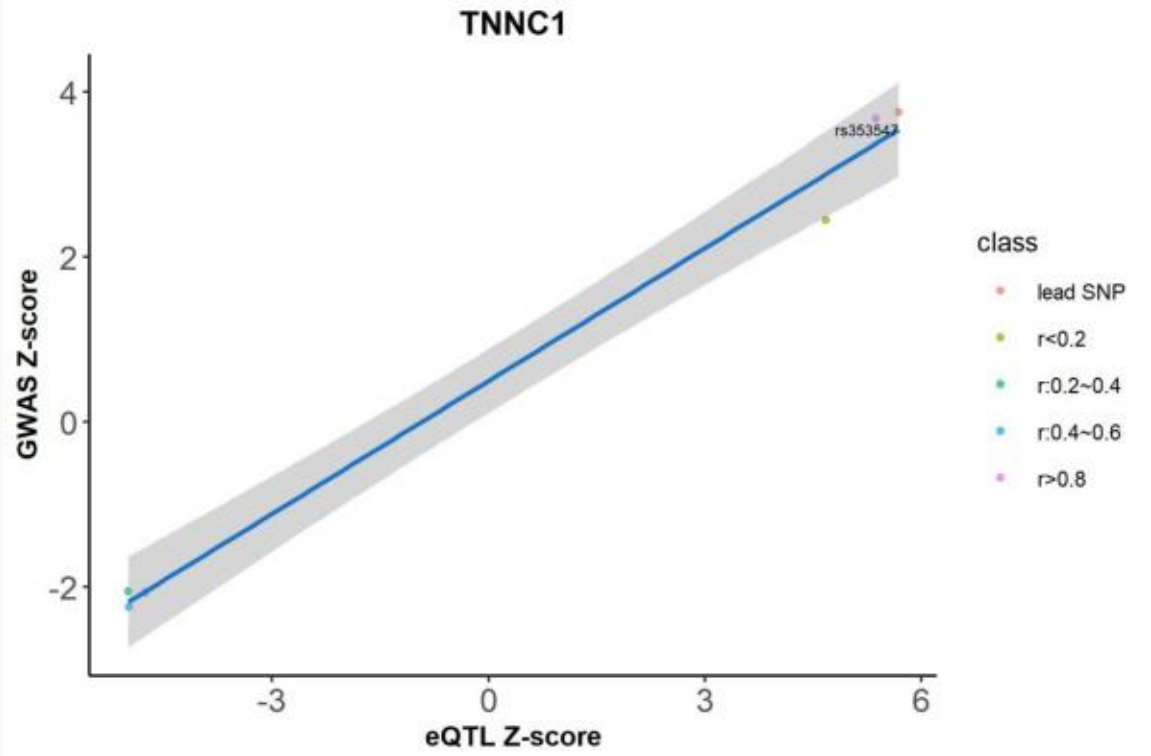

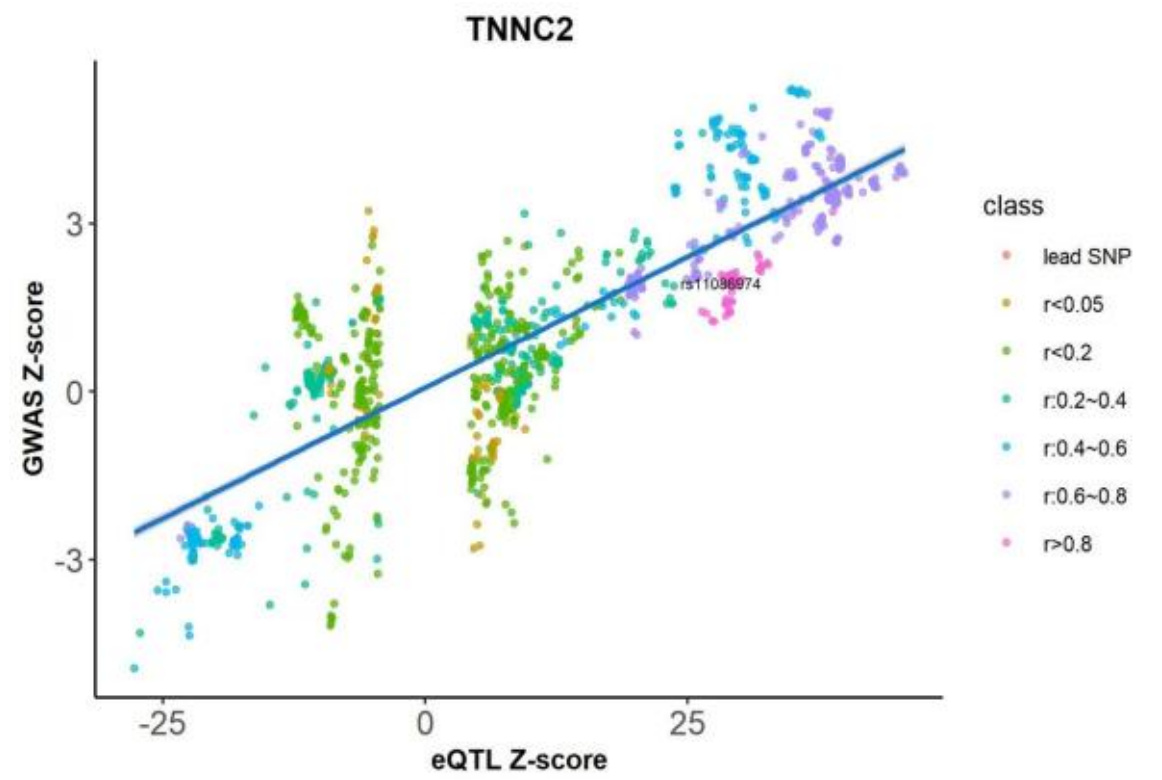

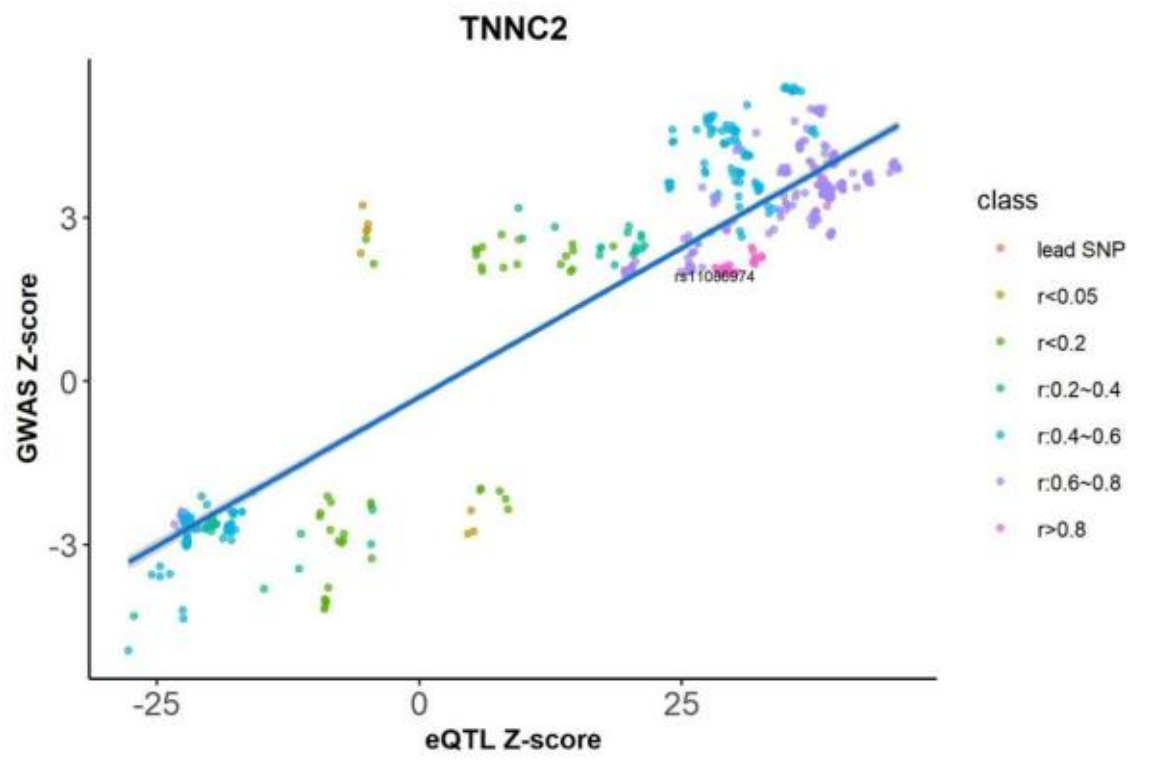

Diuretics

Before | **After**

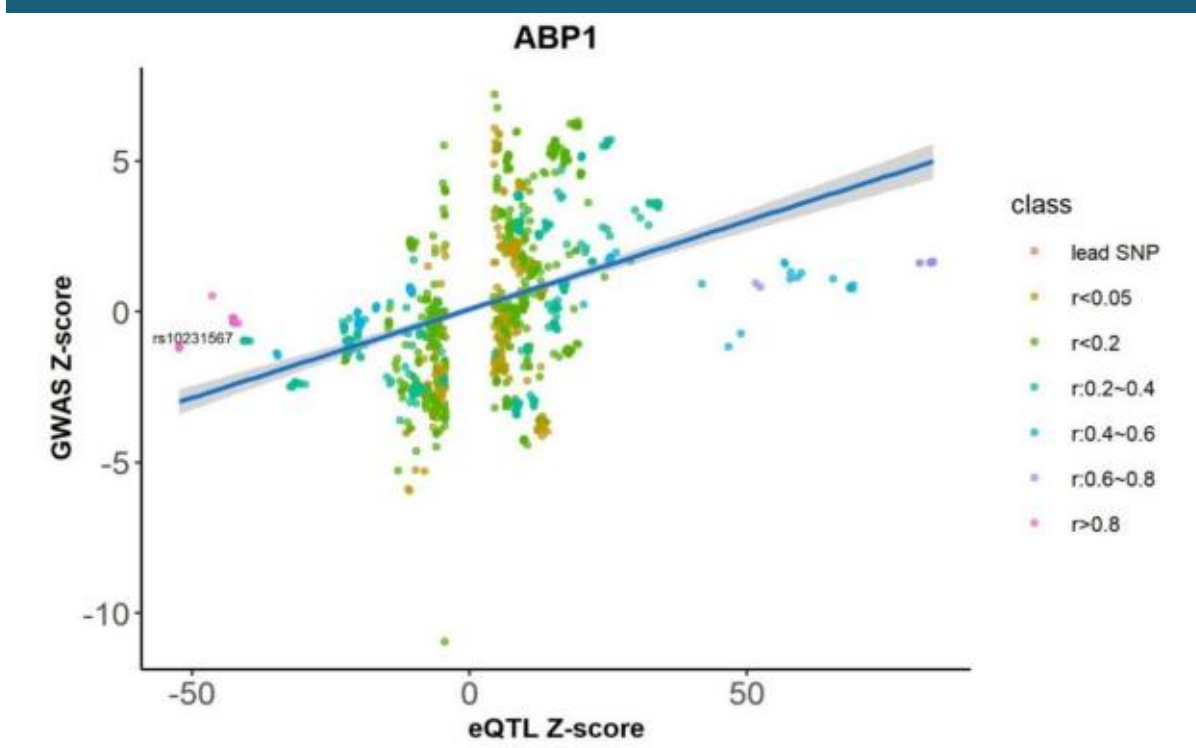

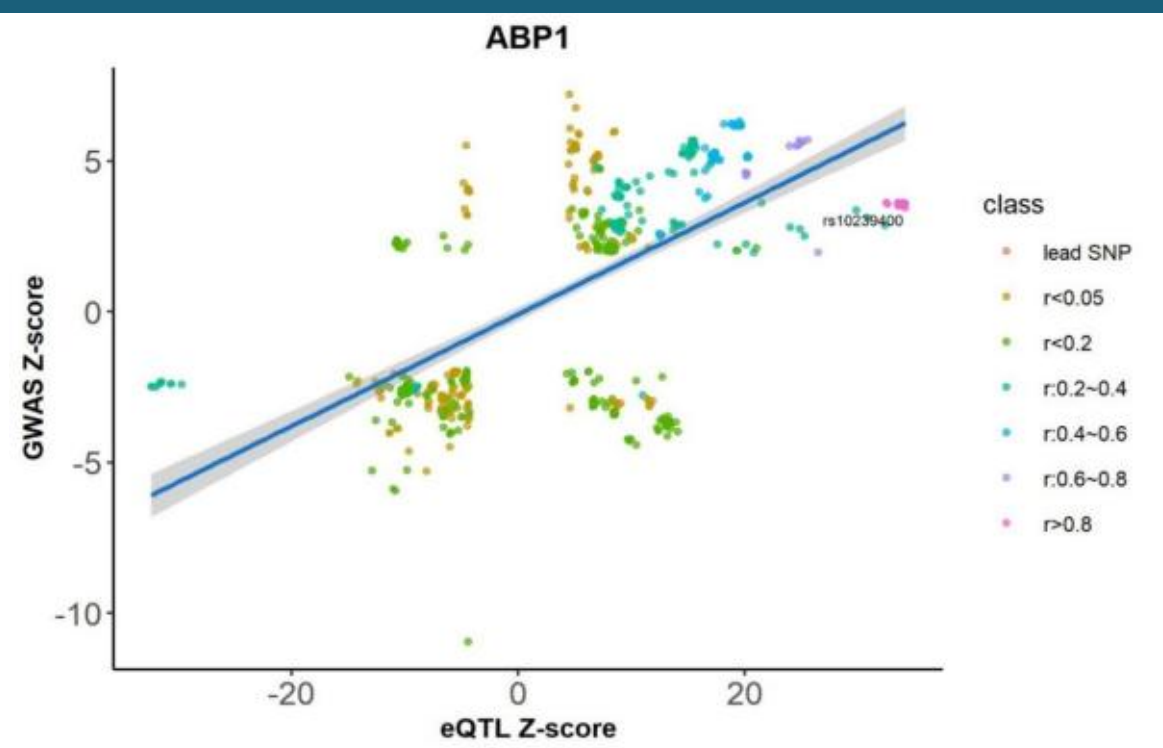

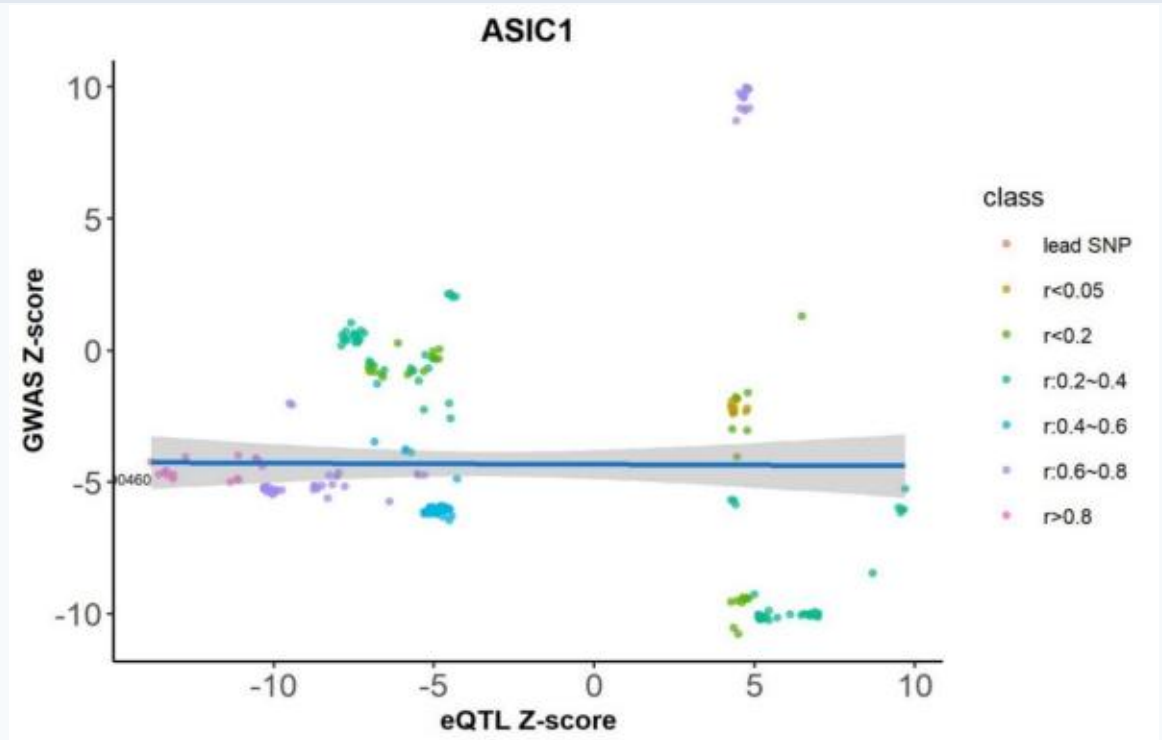

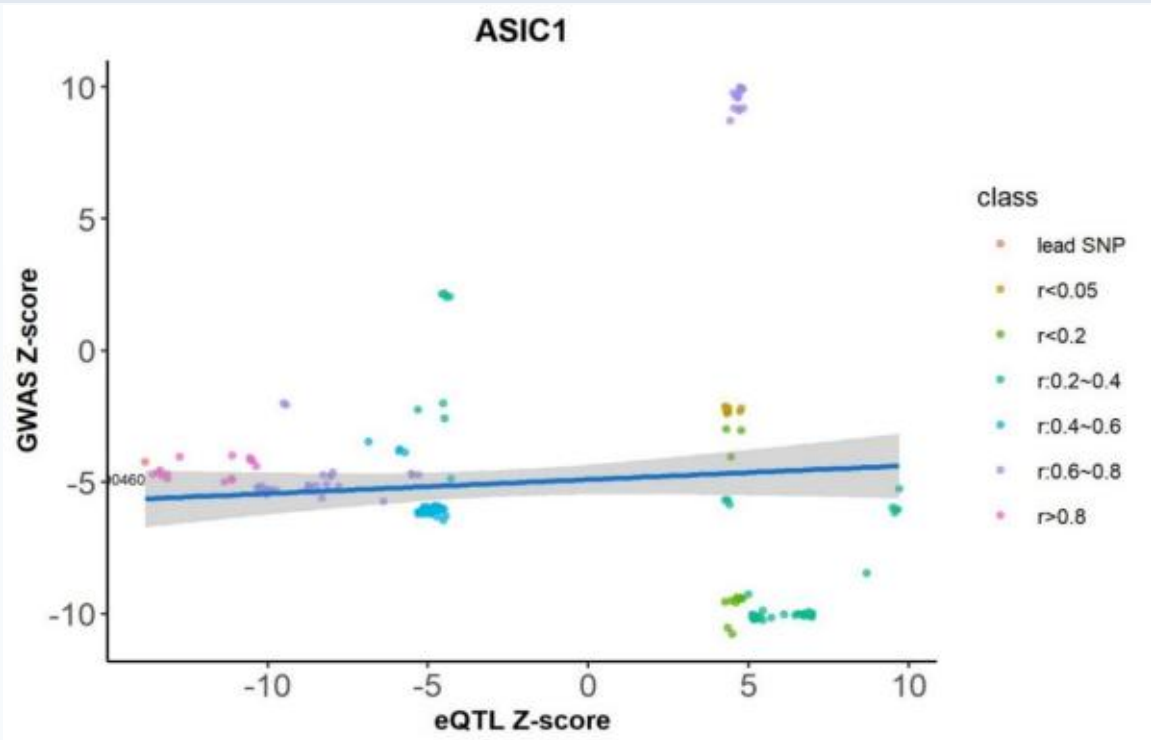

Before	After
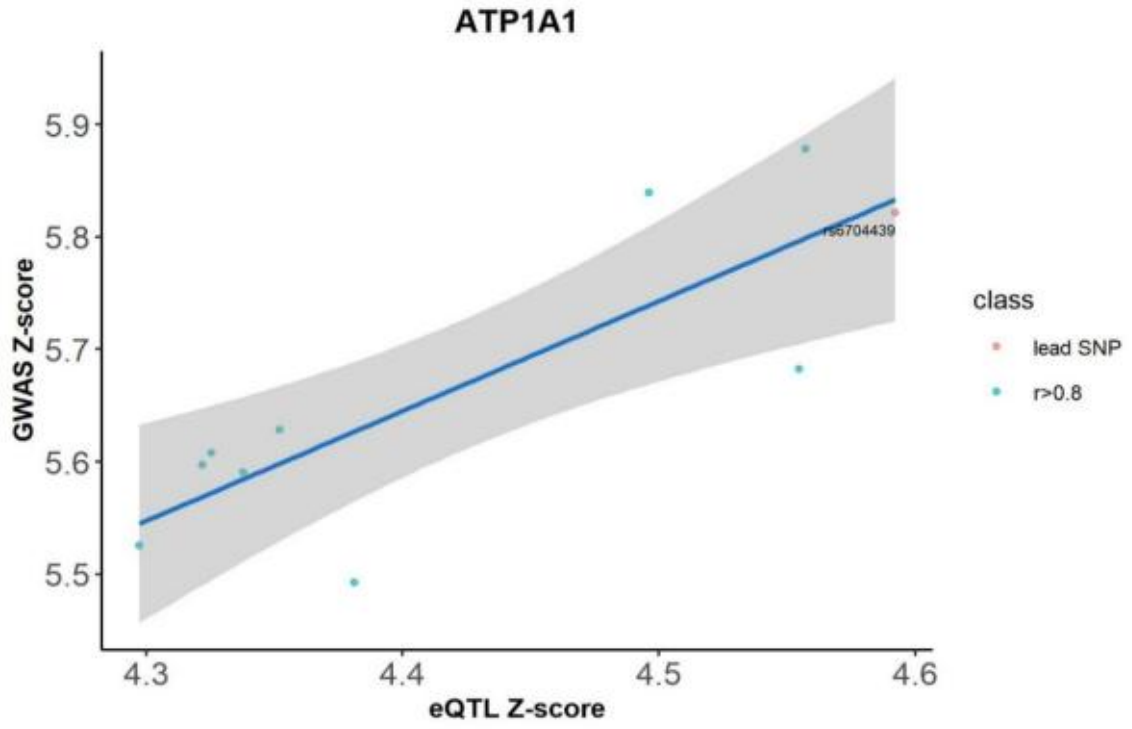	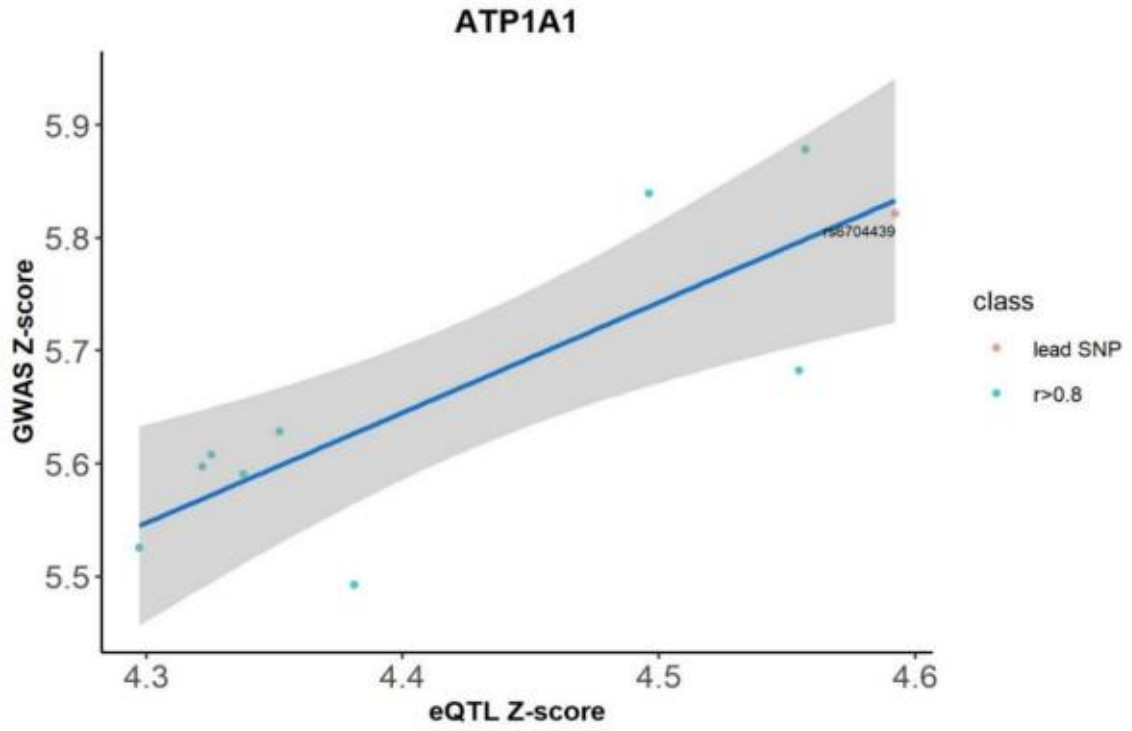
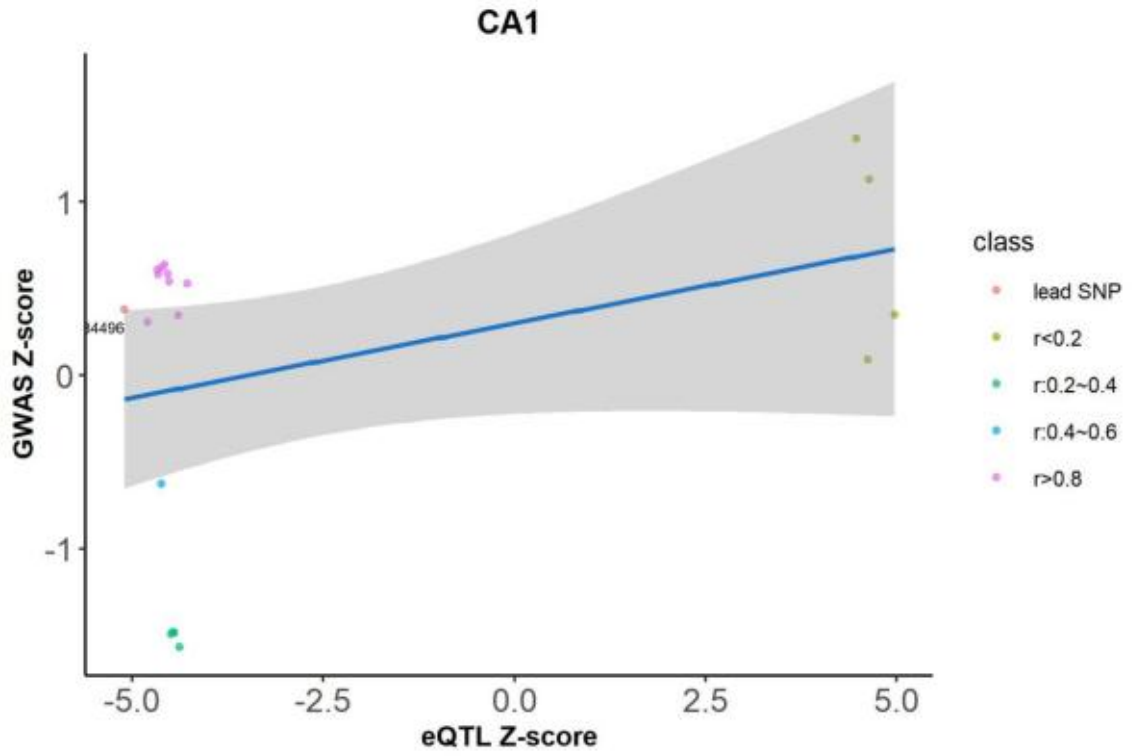	*No significant variant retrieved*
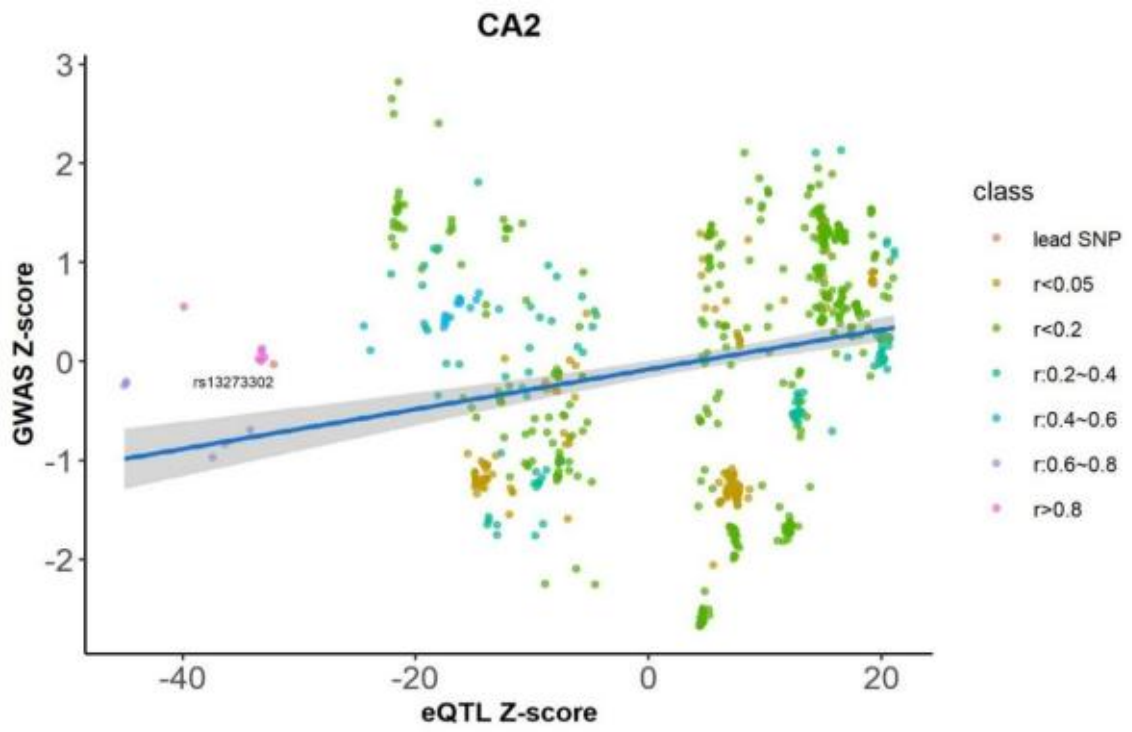	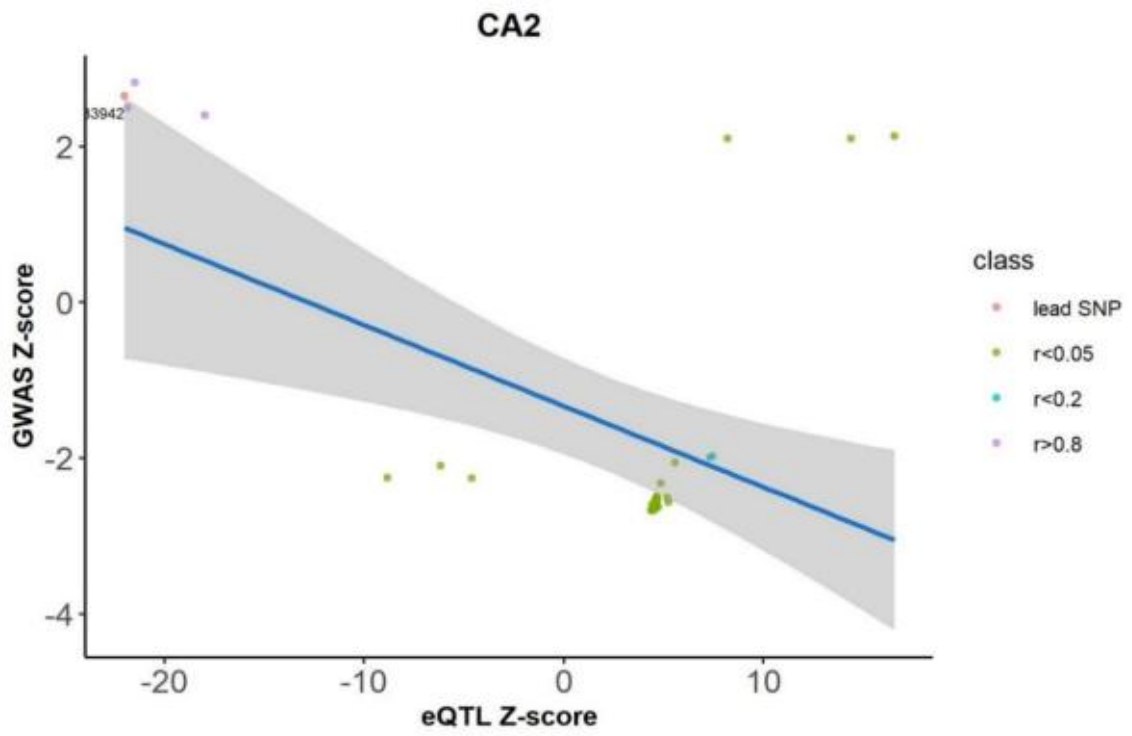
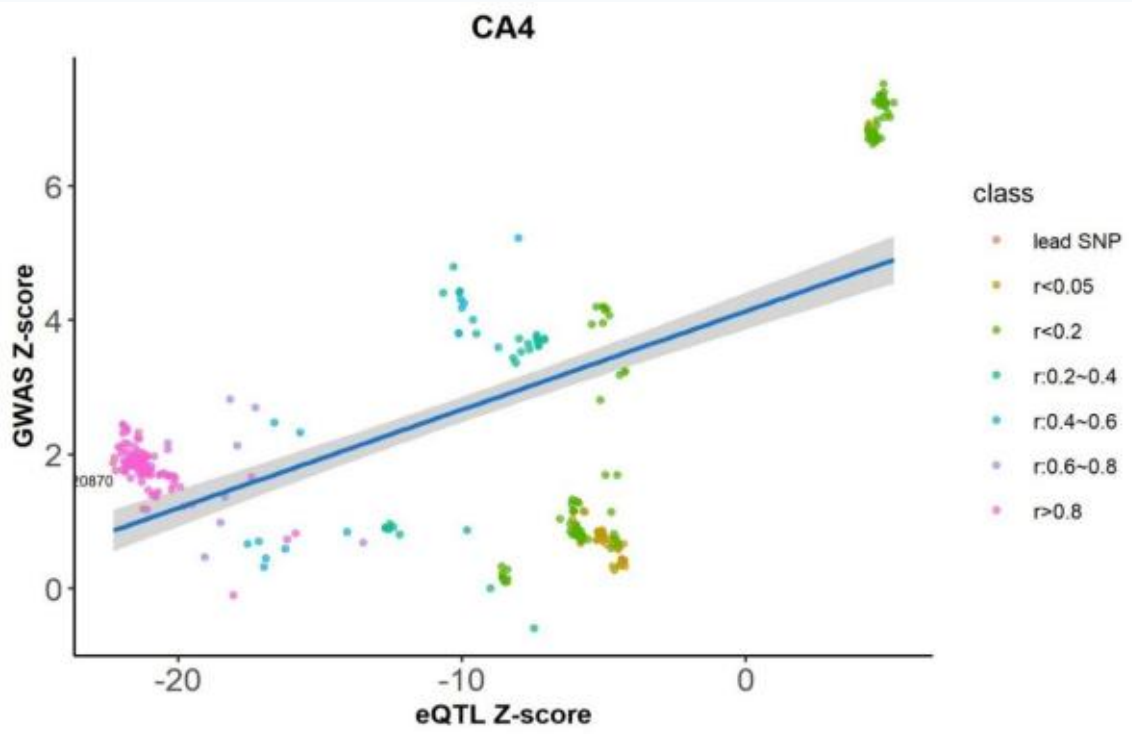	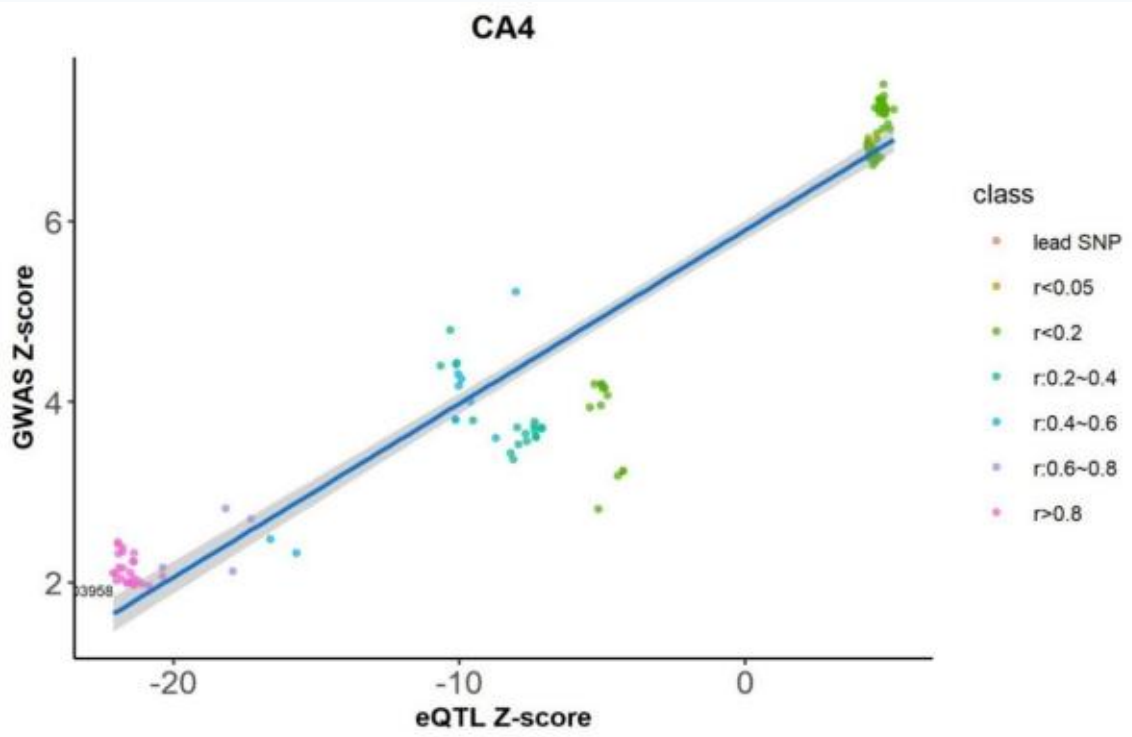

Before	After

Before

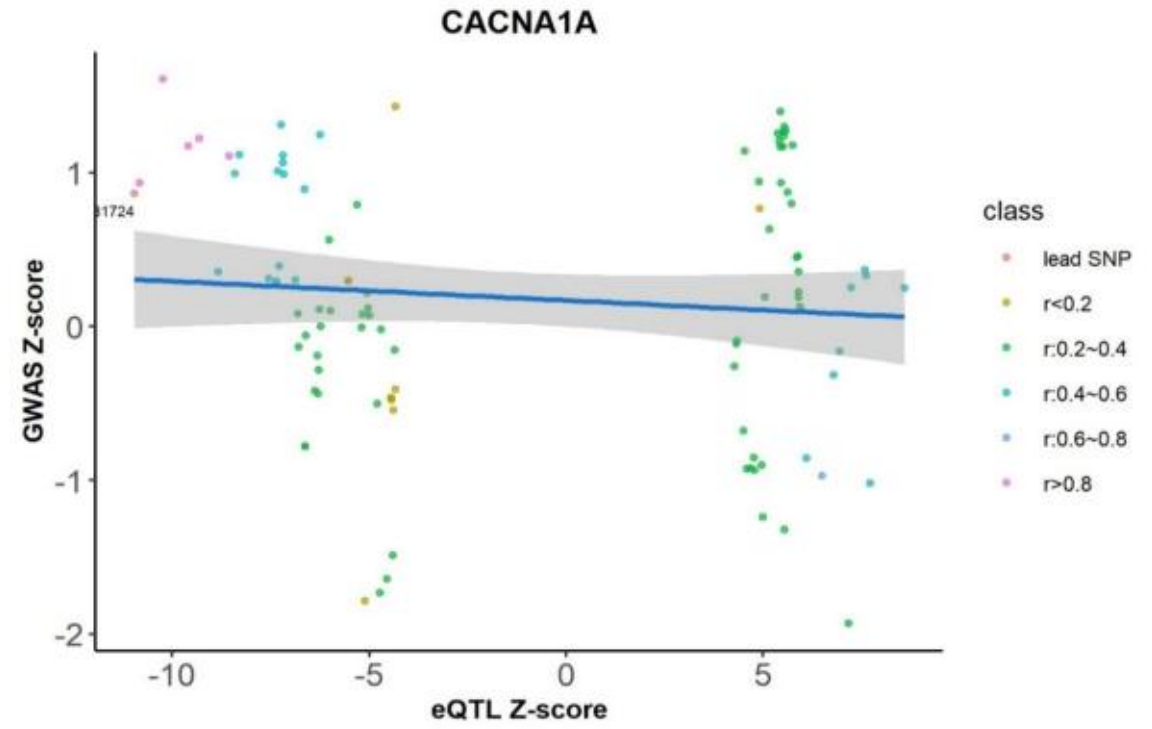

After

No significant variant retrieved

Before

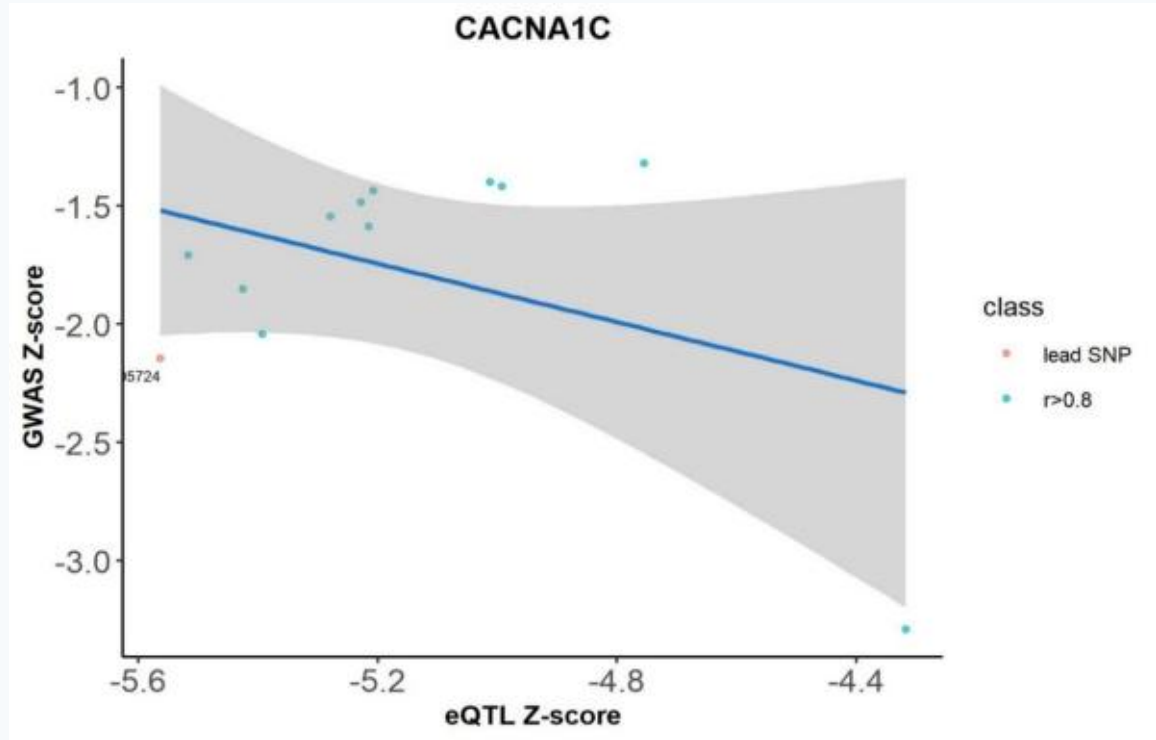

After

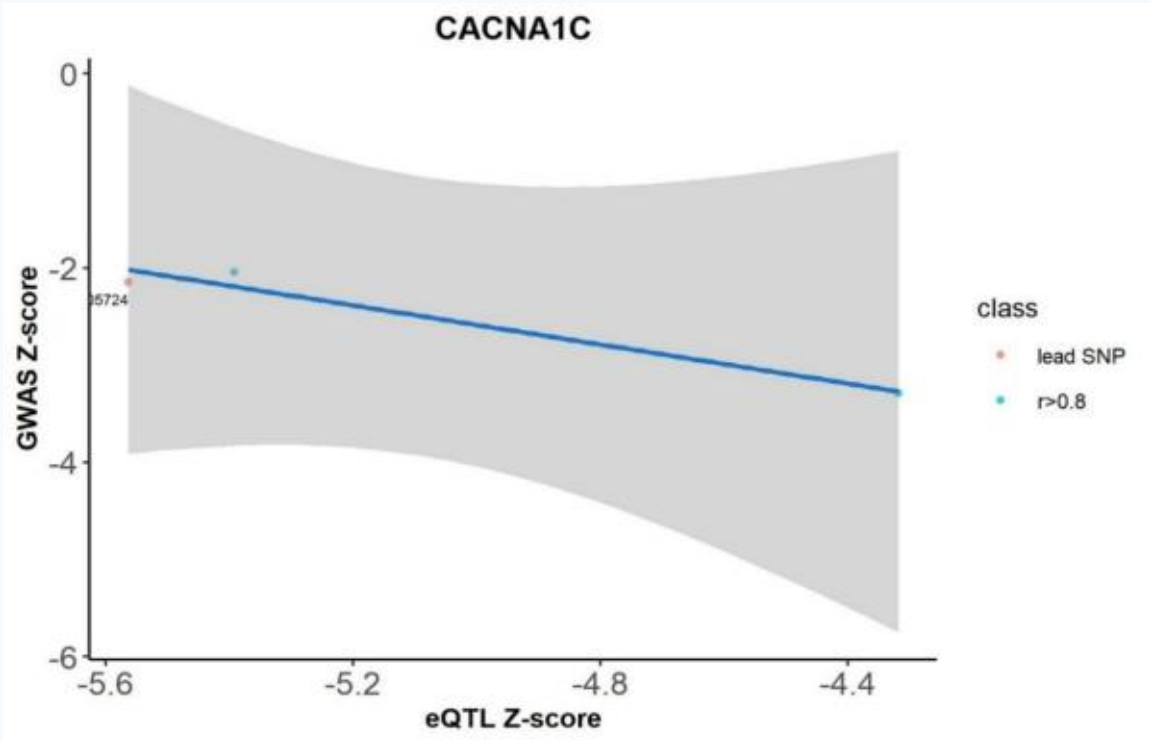

Before

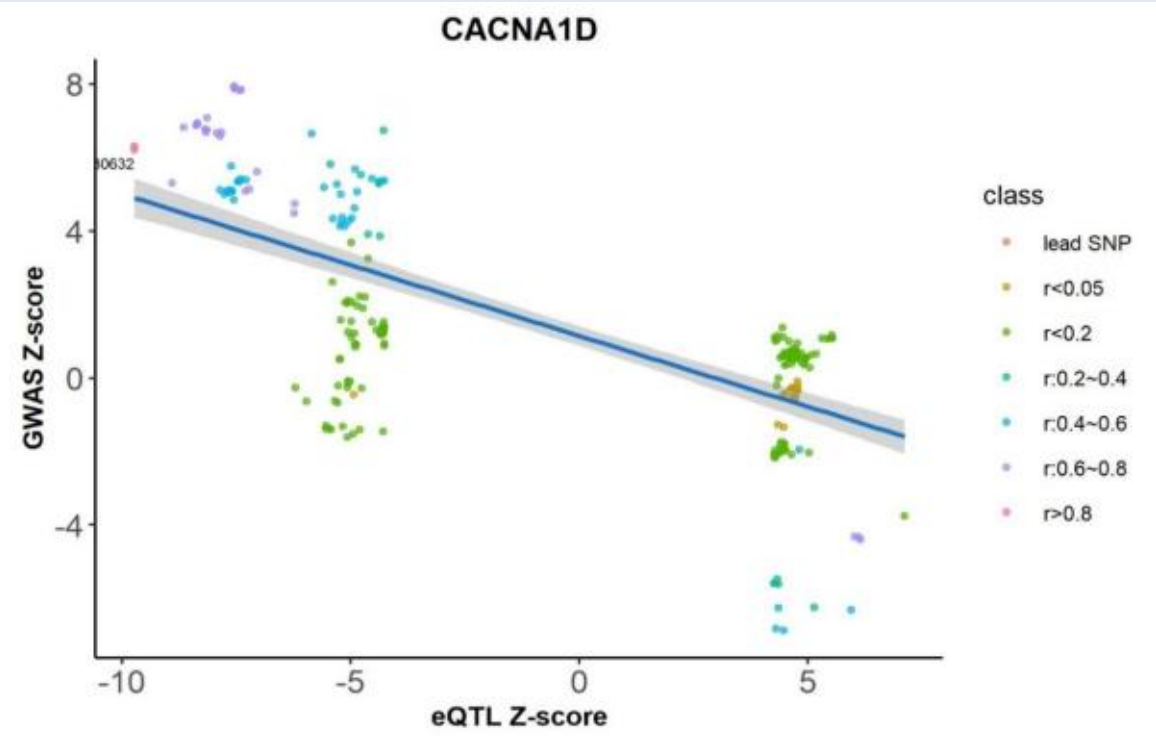

After

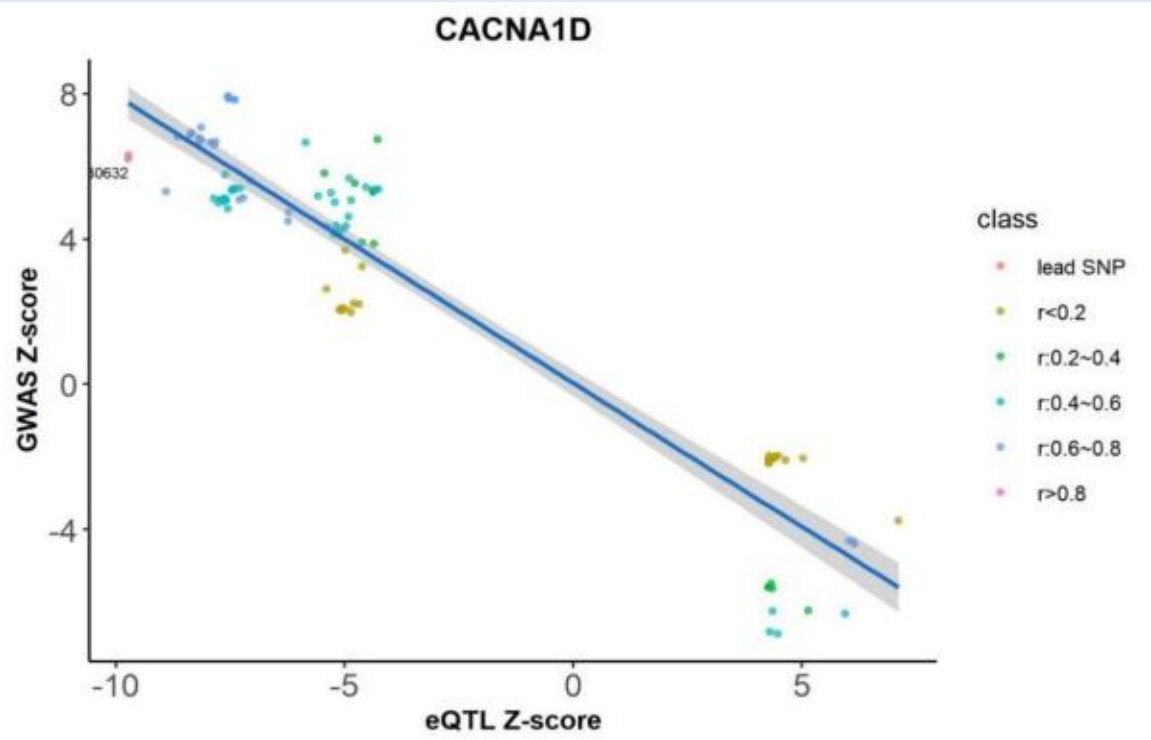

Before

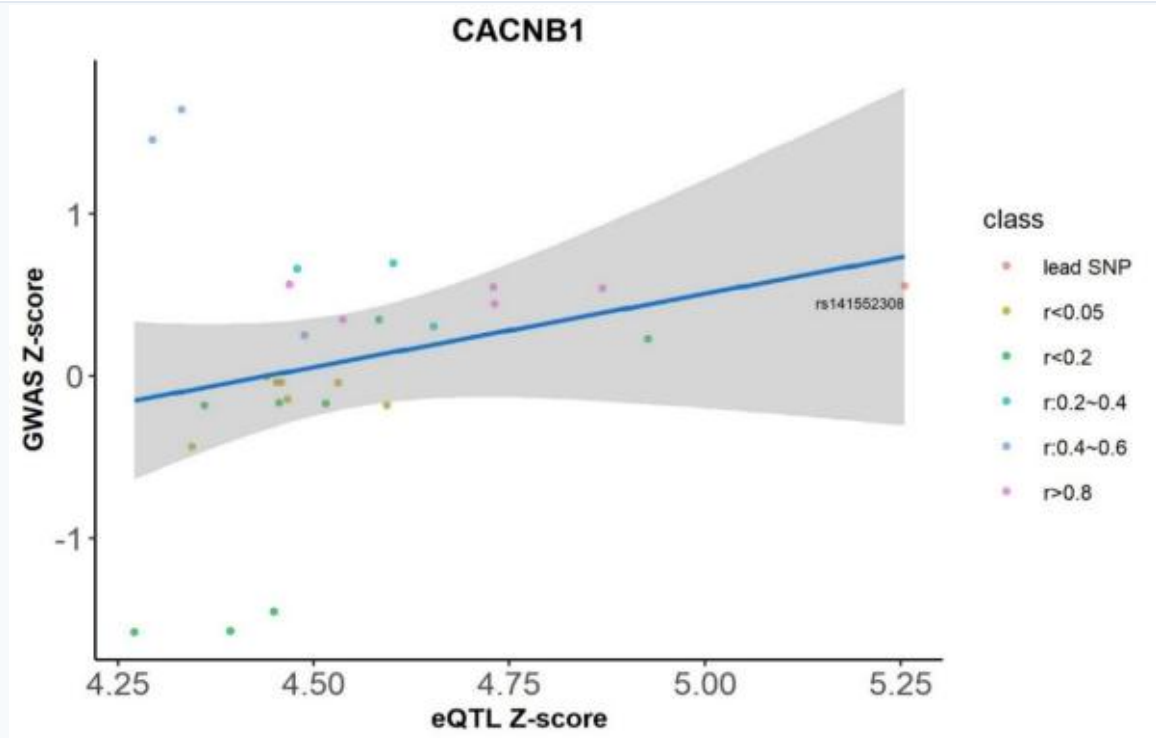

After

No significant variant retrieved

Before	After

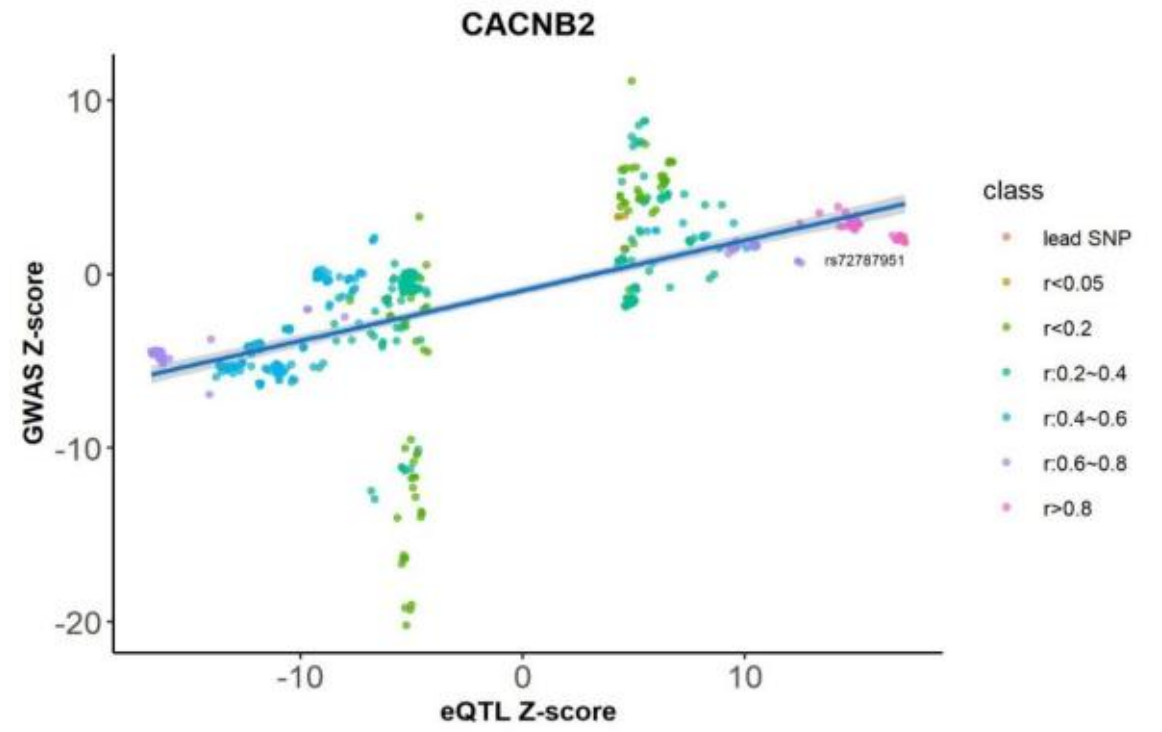

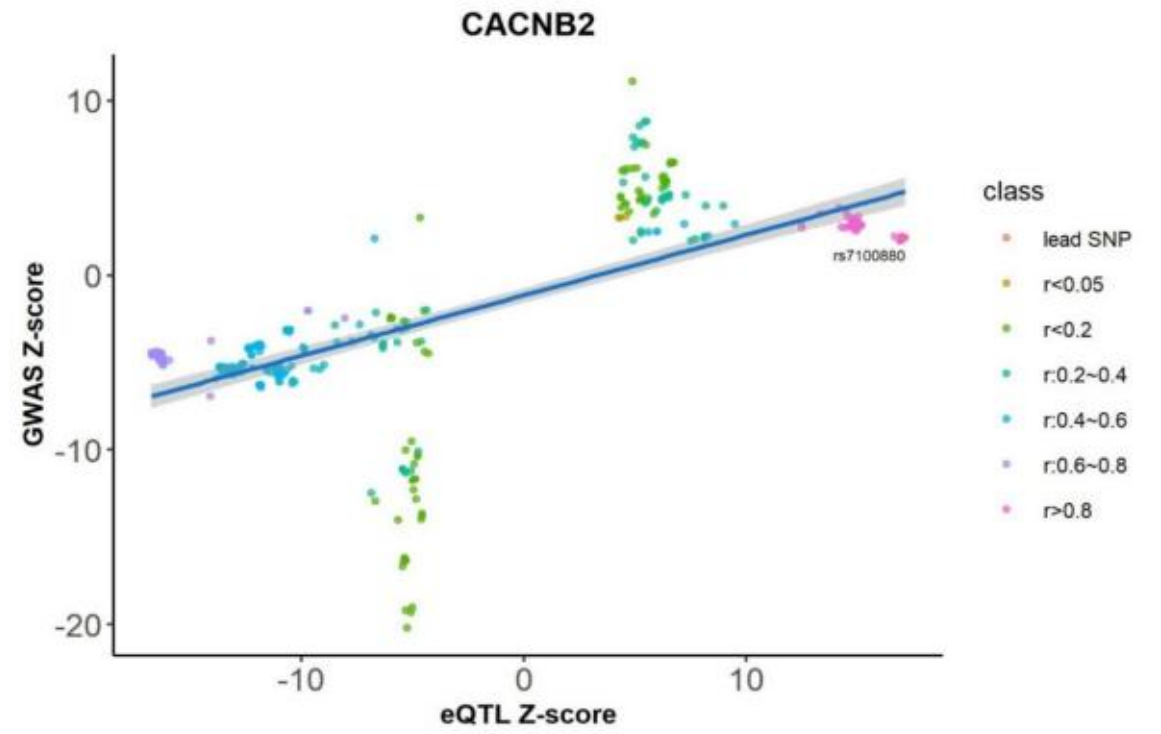

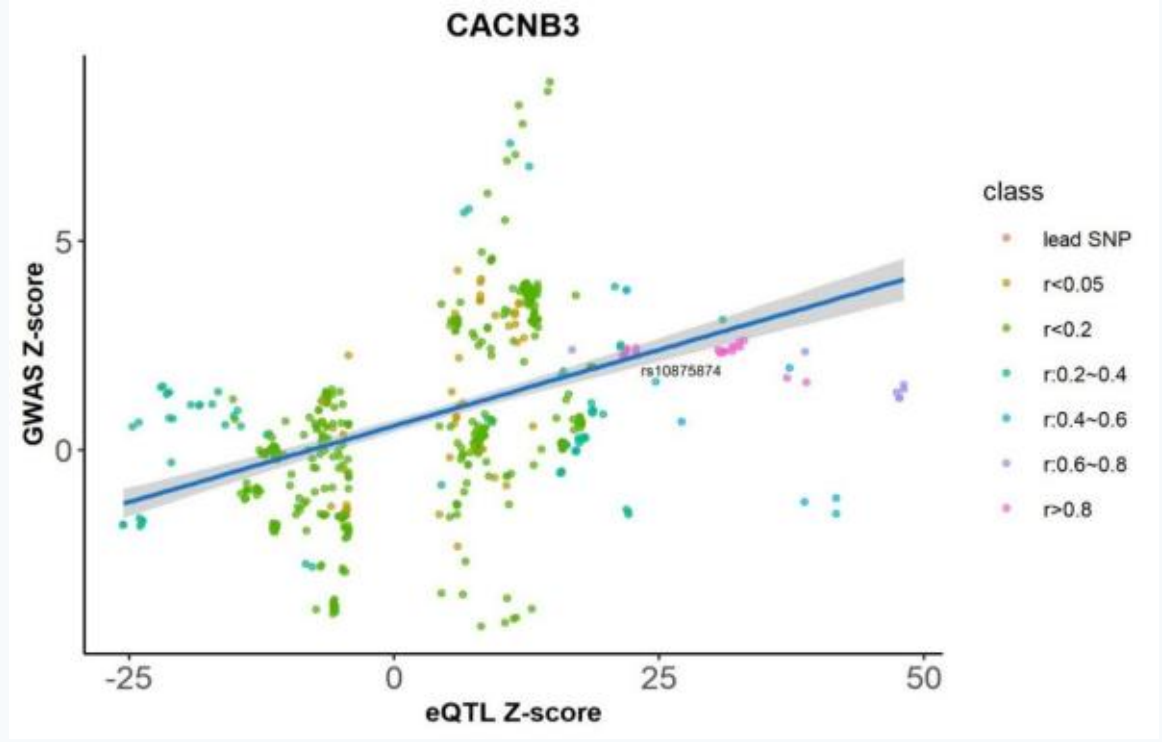

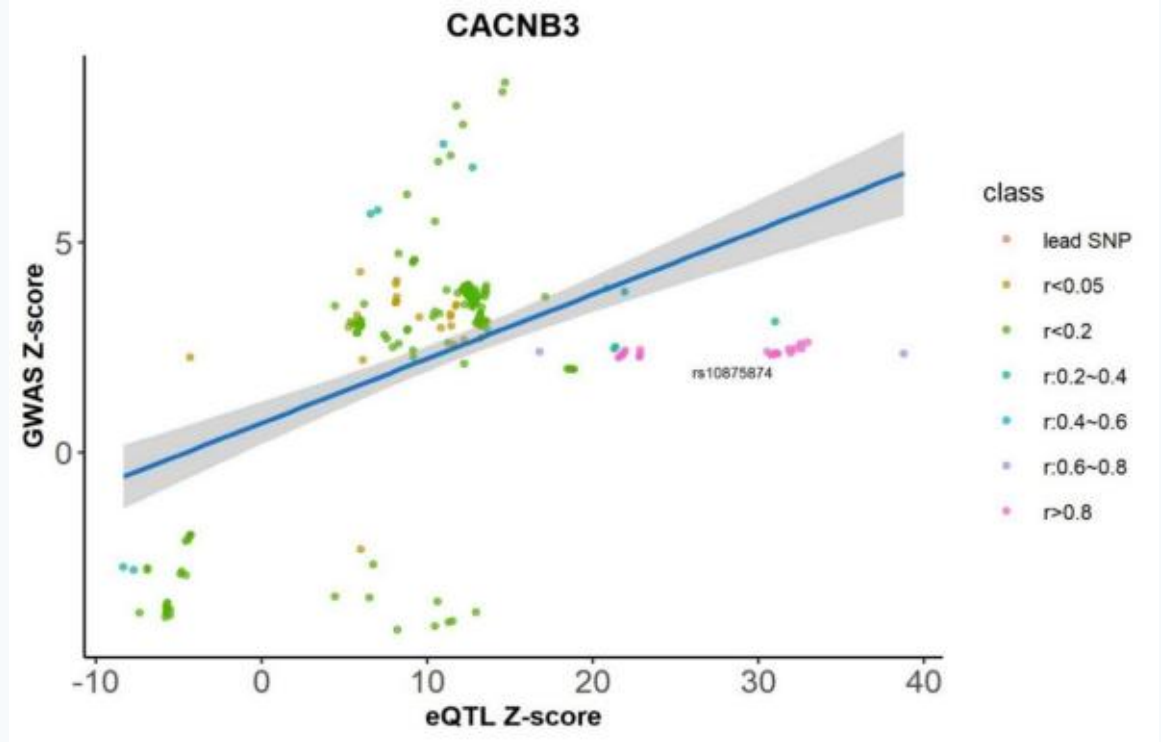

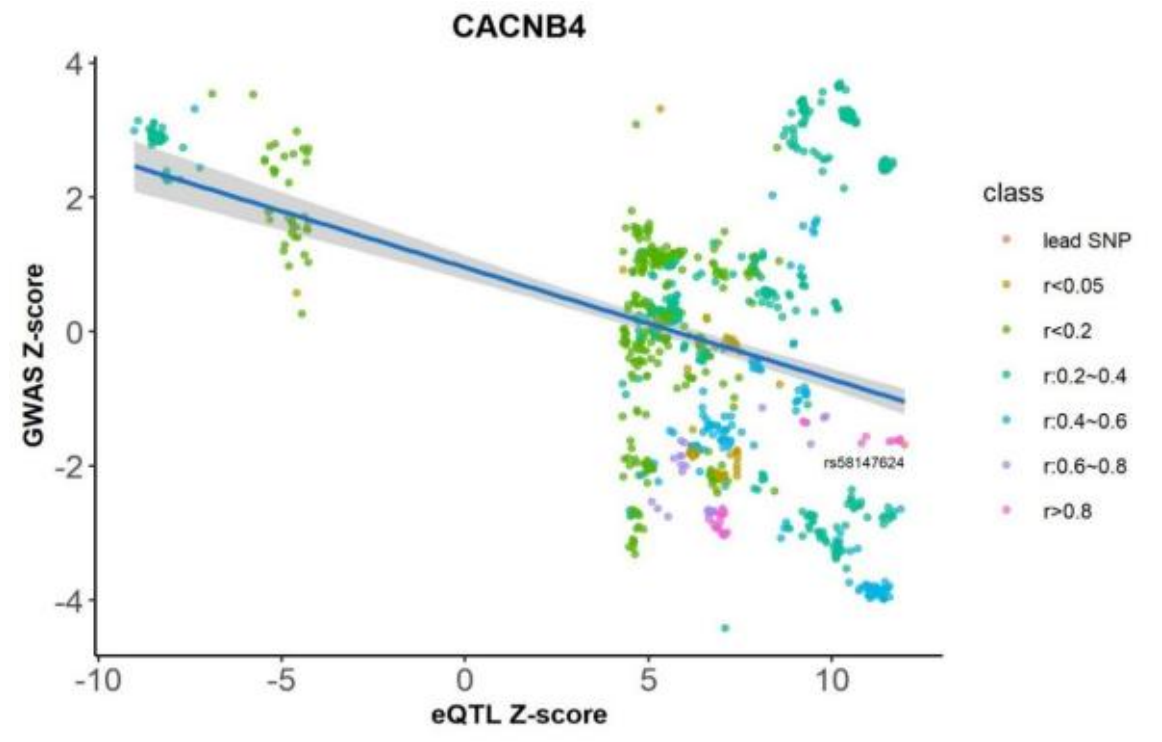

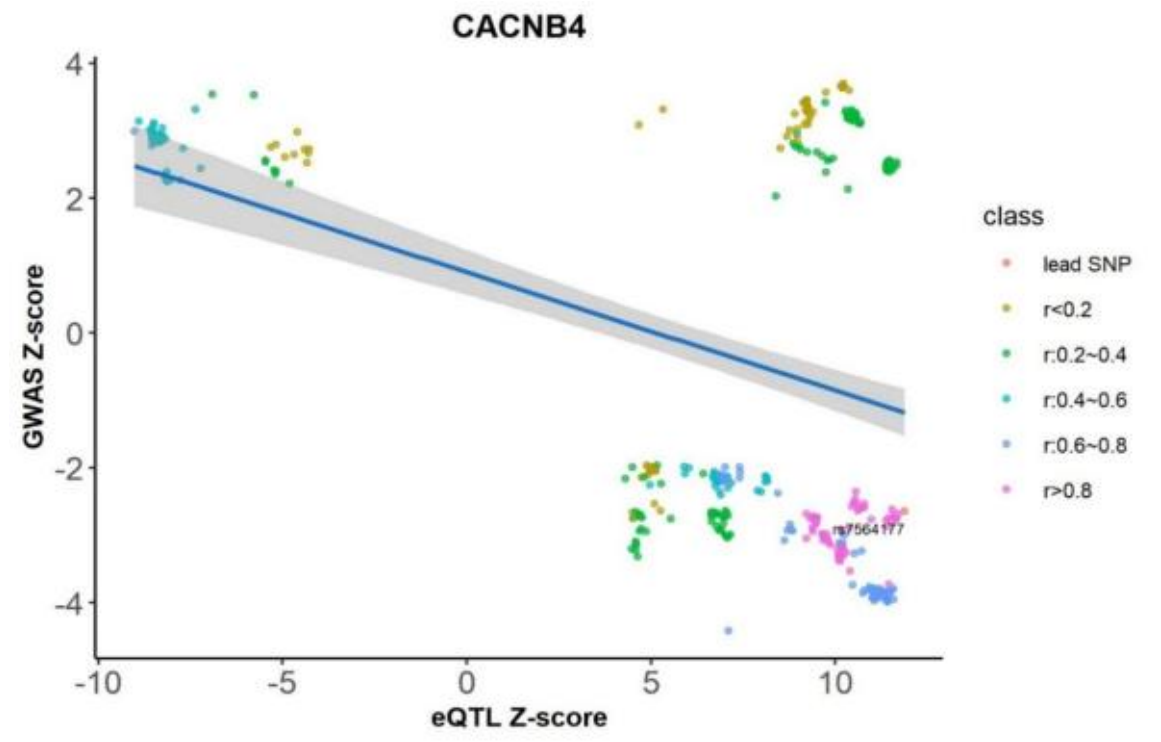

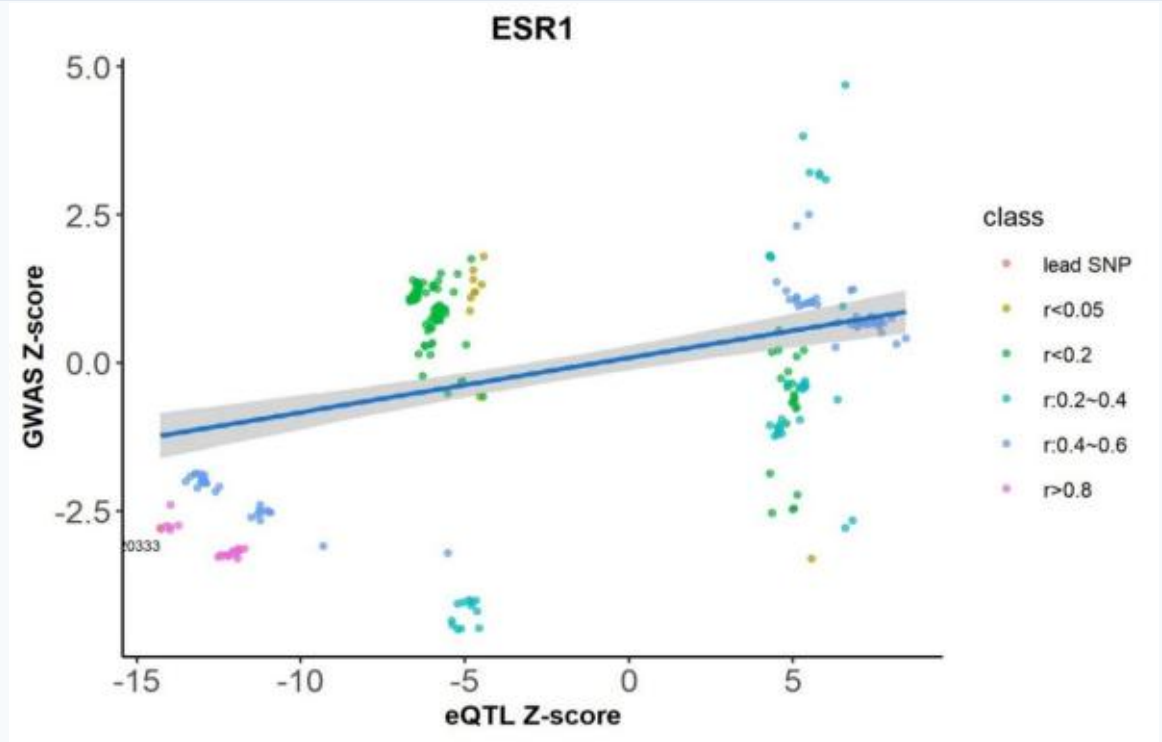

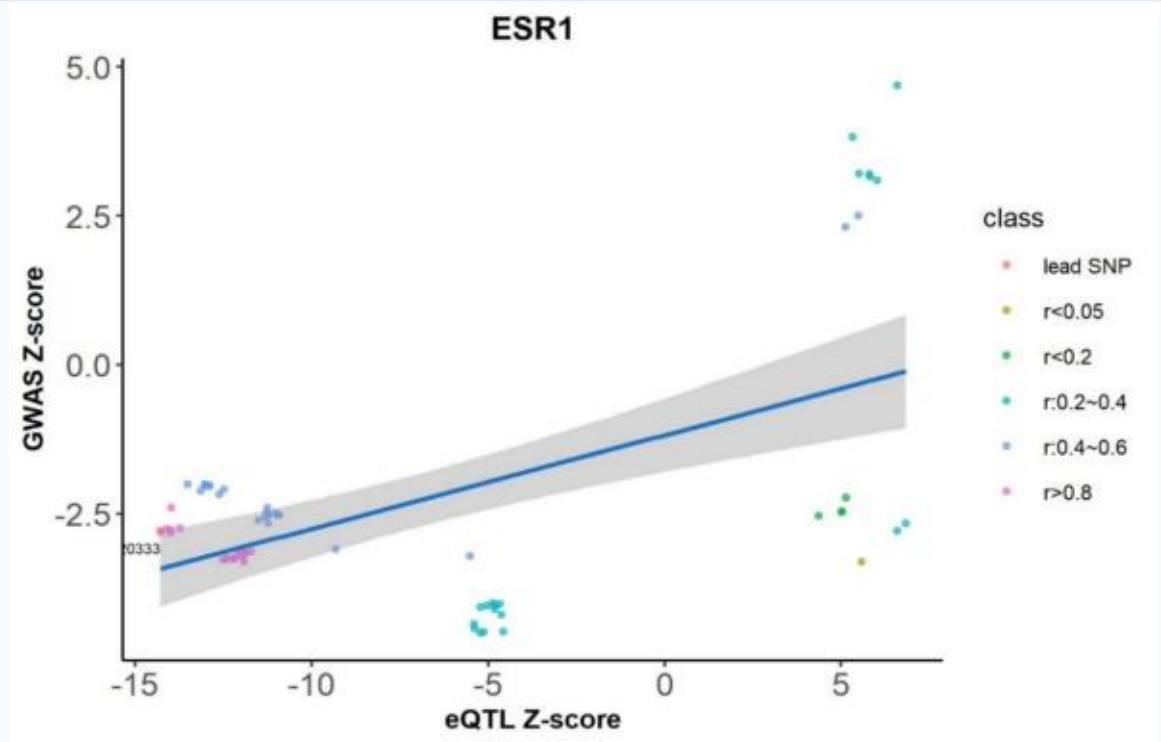

Before	After

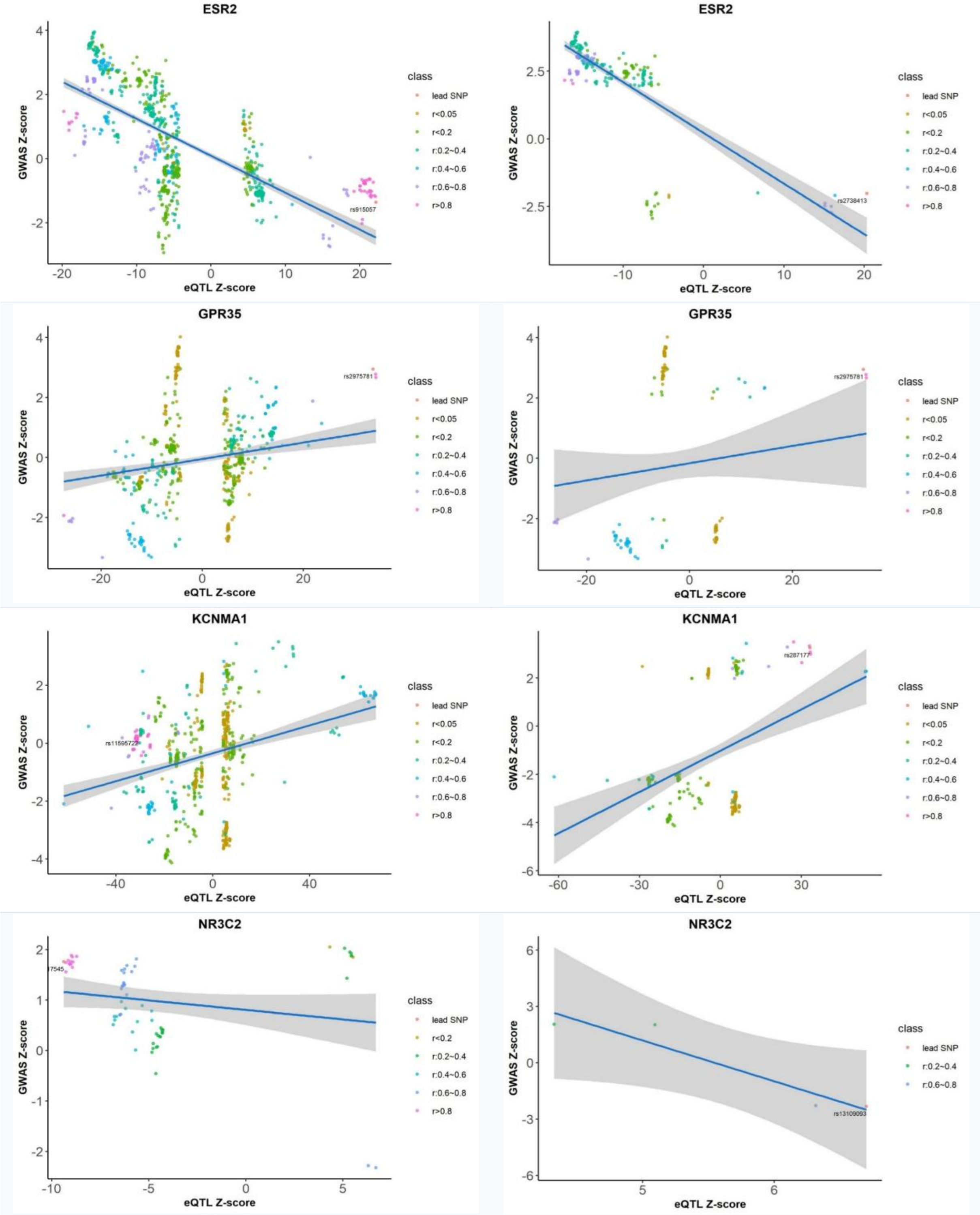

Before	After

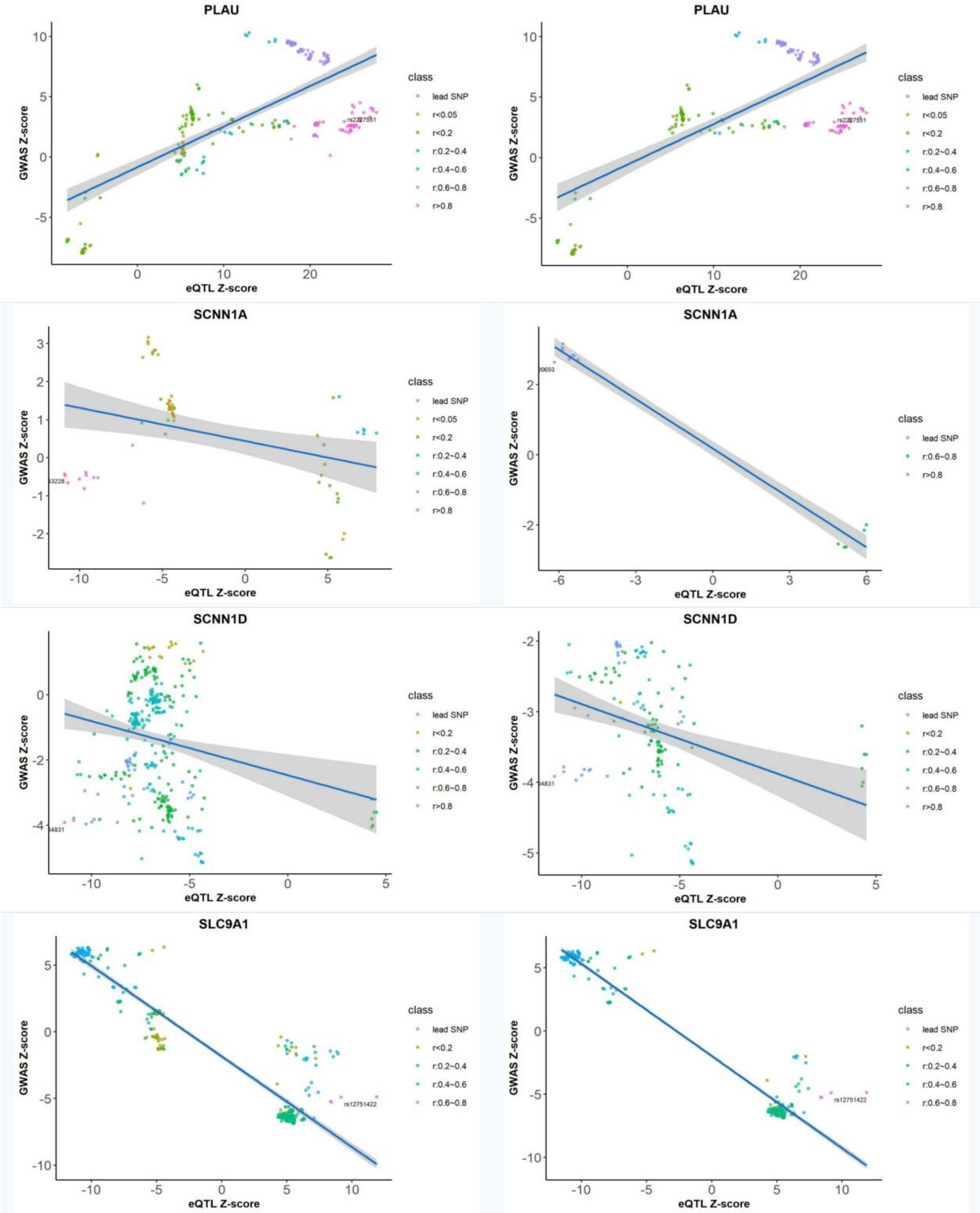

Before	After

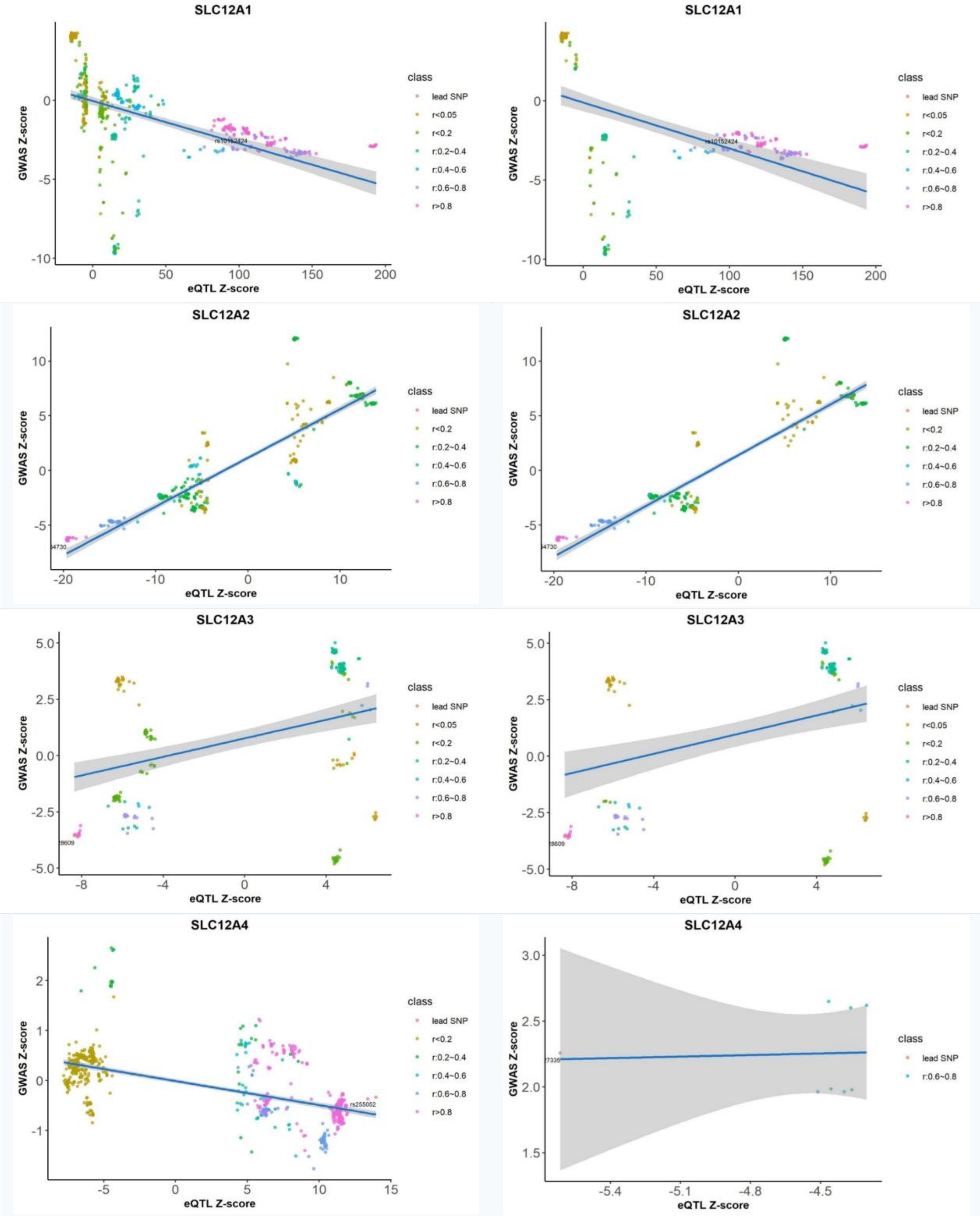

Figure S10. Sensitivity drug-target Mendelian randomisation analyses for renin–angiotensin system (RAS) inhibitor targets using GLS-IVW and GSMR with HEIDI-outlier filtering

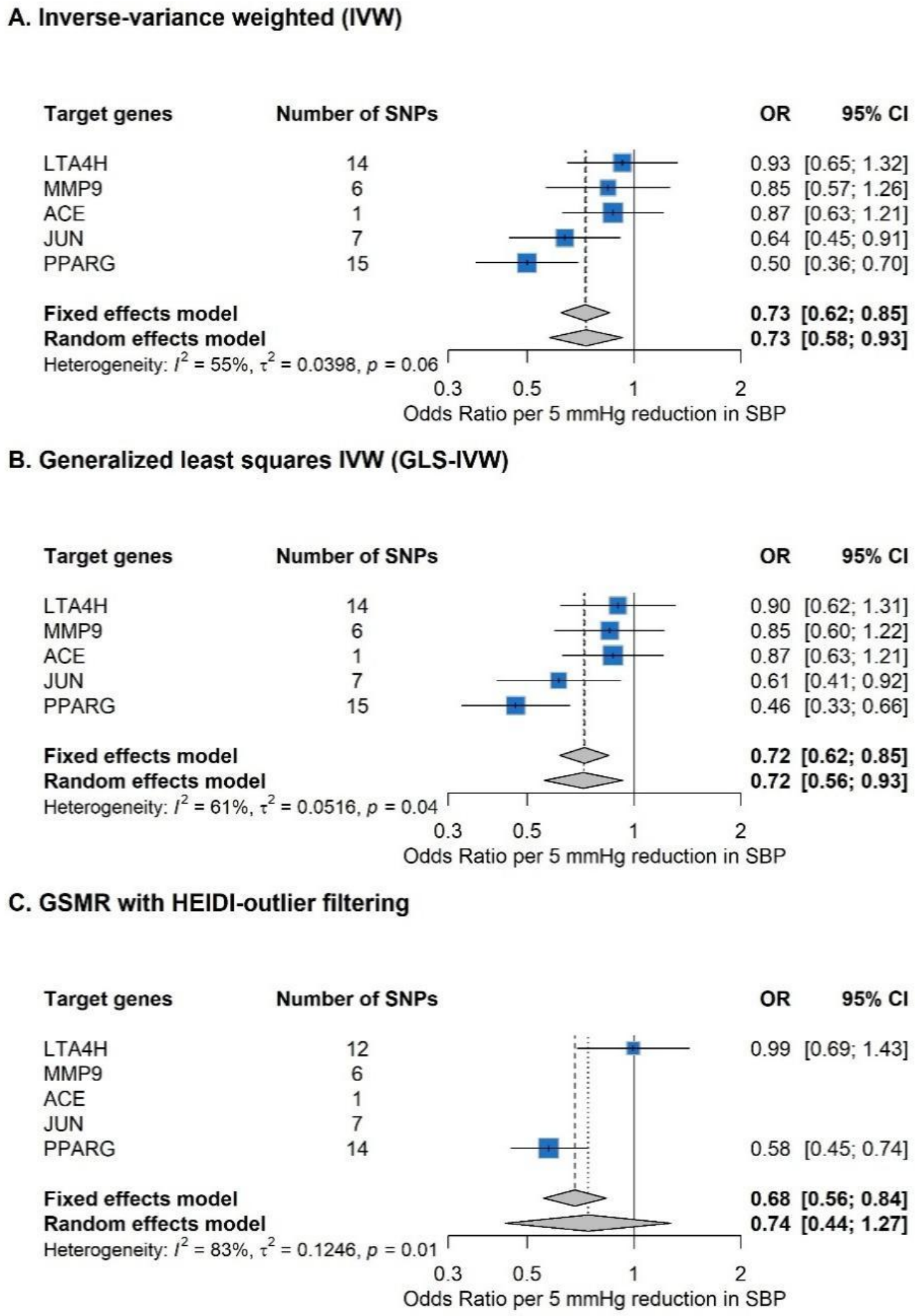

Note: In panels A and B, when only a single variant remained after LD clumping, the Wald ratio was used instead of IVW. In the GSMR analysis, HEIDI-outlier filtering removed 2 variants for LTA4H and 1 for PPARG. GSMR could not be performed for the remaining genes because fewer than 10 variants were available as instruments.

Abbreviations: IVW, inverse-variance weighted; GLS-IVW, generalized least squares inverse-variance weighted; GSMR, generalized summary-data-based Mendelian randomization; HEIDI, heterogeneity in dependent instruments; SNP, single nucleotide polymorphism; SBP, systolic blood pressure; OR, odds ratio;

Figure S11. Leave-one-out meta-analysis of the renin–angiotensin system (RAS) inhibitor target genes for the effect on non-alcoholic fatty liver disease (NAFLD). Each row shows the pooled fixed-effect odds ratio (per 5 mmHg reduction in systolic blood pressure) after excluding the indicated target gene; the pooled estimate remained statistically significant after excluding any single gene, with PPARG being the most influential.

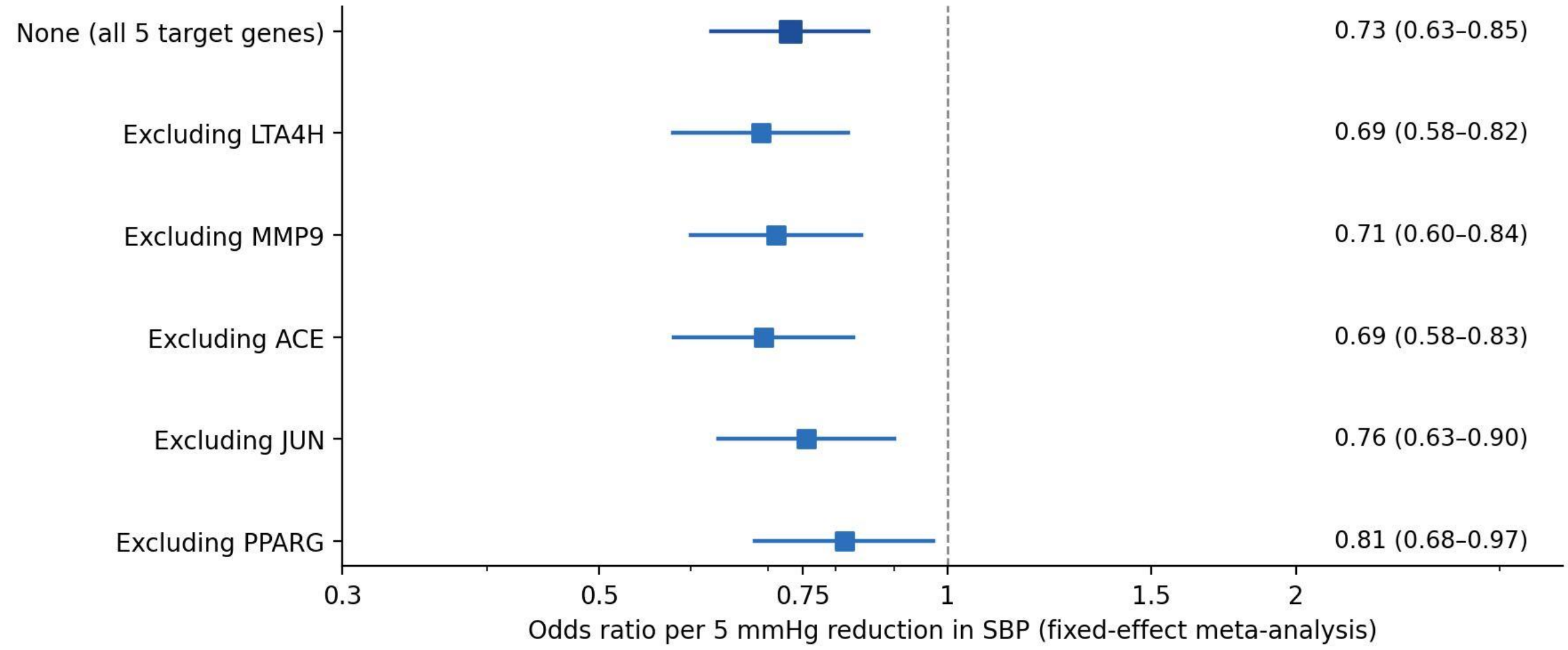

Figure S12. Results of the replication analysis for the effect of renin-angiotensin system (RAS) inhibitors on non-alcoholic fatty liver disease, using genetic instruments validated by cis-eQTL data from GTEx

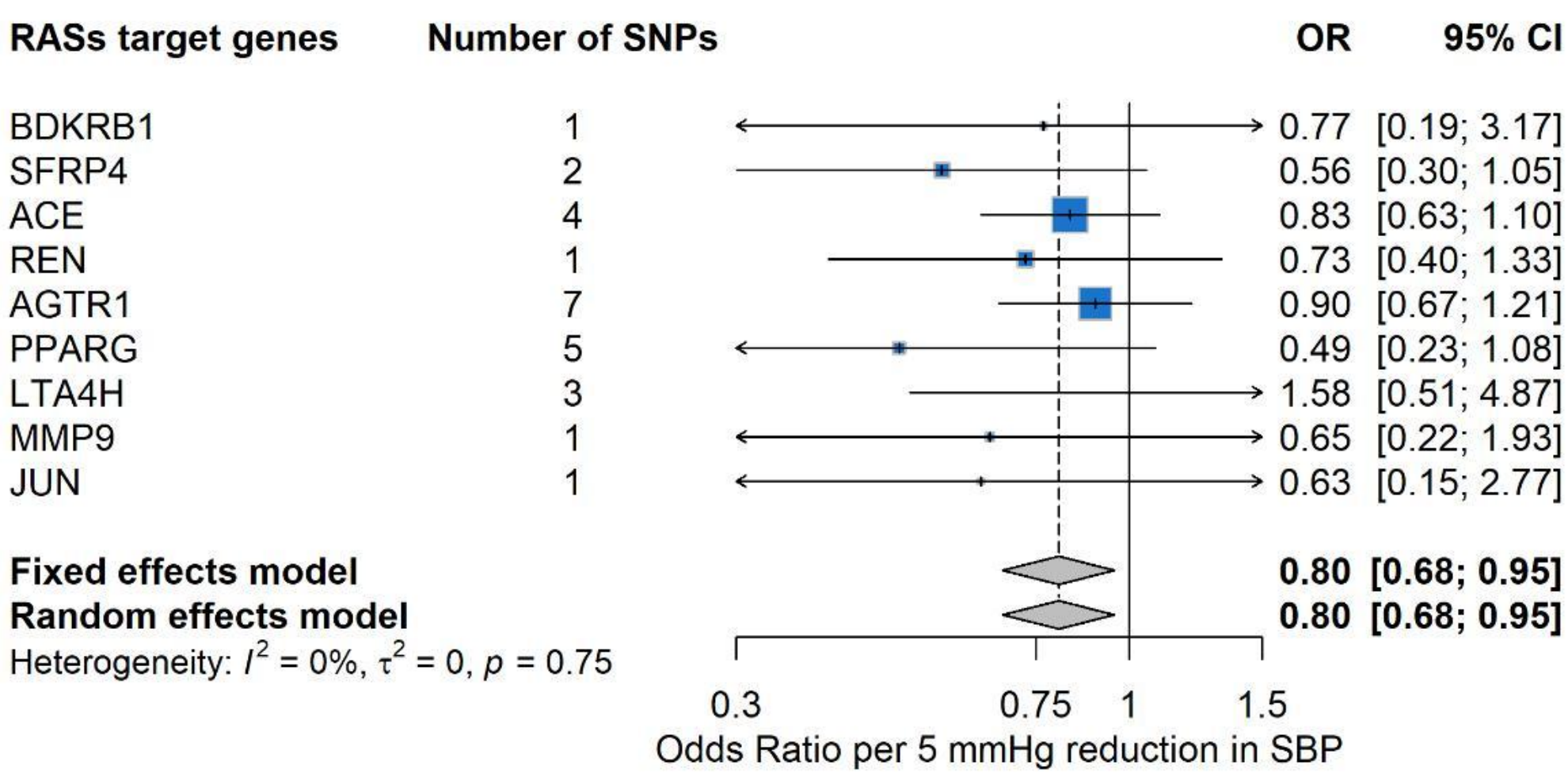

Abbreviations: SNPs: single nucleotide polymorphisms; SBP: systolic blood pressure; OR: odds ratio; CI: confidence interval.

Figure S13. Results of the colocalisation analyses and LocusZoom plots for significant genes

Gene	Single causal variant assumption		Multiple causal variants assumption	
	Precise gene position	Precise gene position ± 500 KB	Precise gene position	Precise gene position ± 500 KB
JUN	PPH3: 1.5e-08 PPH4: 2.3e-06	PPH3: 6.4e-02 PPH4: 2.5e-01	NULL	NULL
PPARG	PPH3: 8.6e-01 PPH4: 1.4e-01	PPH3: 9.1e-01 PPH4: 8.8e-02	variant 1: PPH3: 9.9e-01 PPH4: 1.7e-07 variant 2: PPH3: 0.4e-01 PPH4: 9.6e-01	variant 1: PPH3: 0.4e-01 PPH4: 9.6e-01 variant 2: PPH3: 9.9e-01 PPH4: 6.4e-06 variant 3: PPH3: 9.9e-01 PPH4: 1.8e-04 variant 4: PPH3: 0.1e-01 PPH4: 9.9e-01
VEGFA	PPH3: 6.33e-07 PPH4: 1.79e-05	PPH3: 8.84e-01 PPH4: 6.06e-04	NULL	variant 1: PPH3: 9.5e-01 PPH4: 0.4e-03 variant 2: PPH3: 0.7e-02 PPH4: 9.9e-01
HIF1A	PPH3: 5.06e-06 PPH4: 6.18e-05	PPH3: 3.79e-02 PPH4: 8.01e-03	NULL	NULL
CACNA1I	PPH3: 5.24e-06 PPH4: 3.47e-05	PPH3: 2.65e-01 PPH4: 1.89e-01	NULL	NULL
CACNA2D3	PPH3: 1.39e-03 PPH4: 7.54e-04	PPH3: 2.77e-01 PPH4: 3.93e-03	NULL	NULL
SLC9A1	PPH3: 2.20e-03 PPH4: 2.08e-01	PPH3: 2.20e-03 PPH4: 9.98e-01	NULL	variant 1: PPH3: 1.9e-01 PPH4: 0.8e+00 variant 2: PPH3: 0.6e+00 PPH4: 0.5e-02 variant 3: PPH3: 0.9e+00 PPH4: 0.2e-02
SLC12A1	PPH3: 1.05e-06 PPH4: 7.61e-06	PPH3: 9.1e-01 PPH4: 2.64e-02	NULL	NULL

Note: "NULL" indicates that there are insufficient variants to perform colocalization under the assumption of multiple causal variants.

Figure S13 (continued) — LocusZoom plots for significant genes

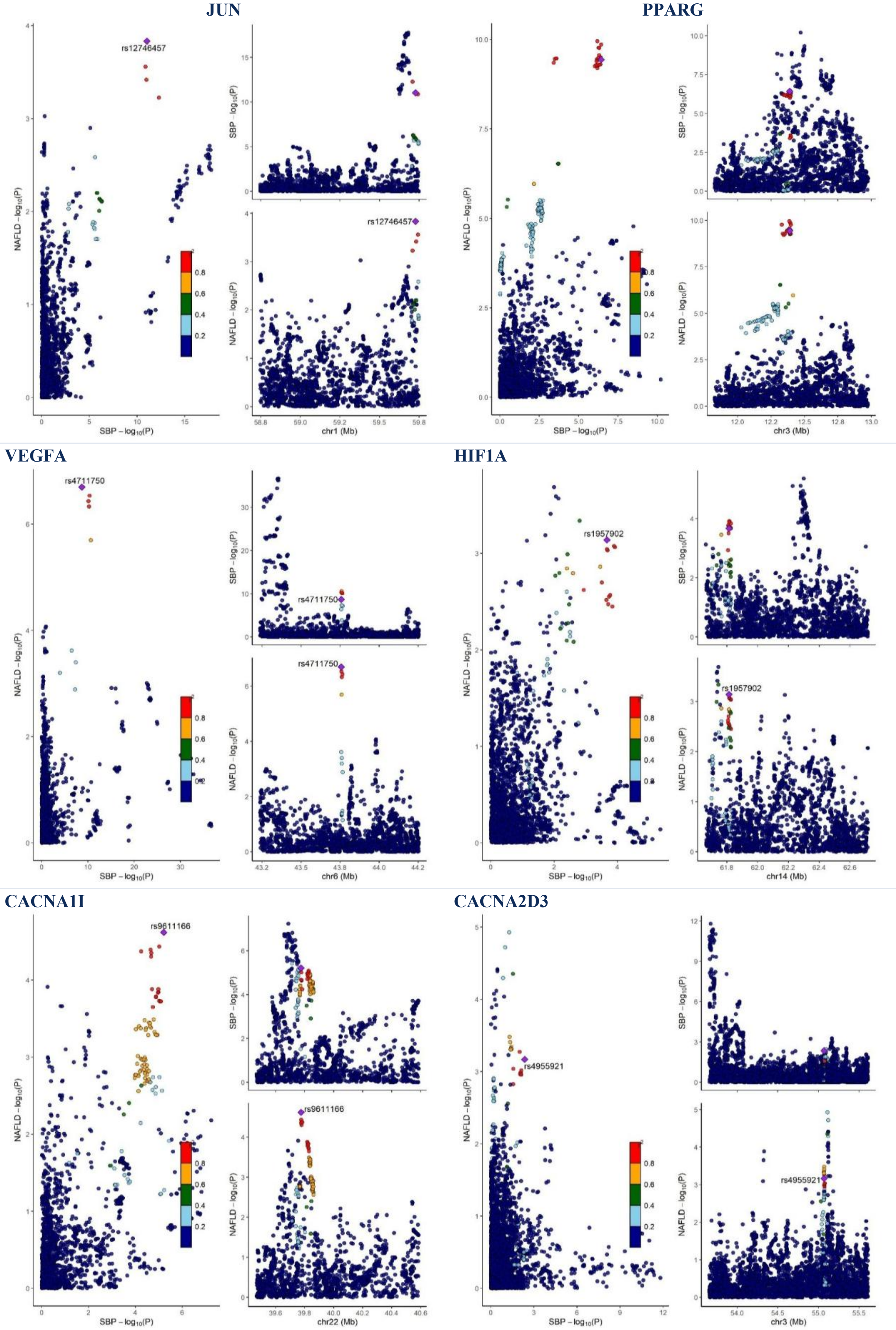

SLC9A1

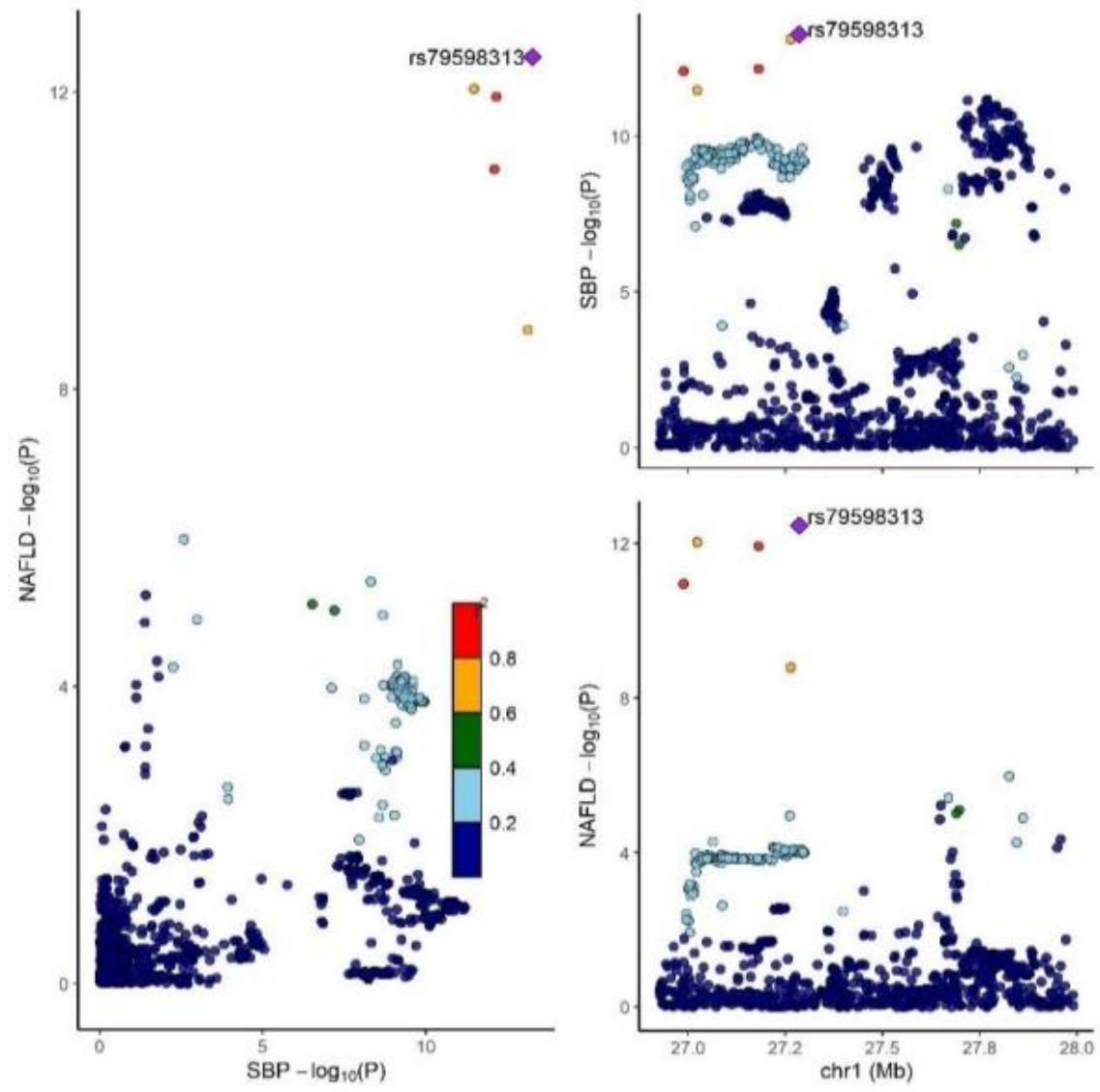

SLC12A1

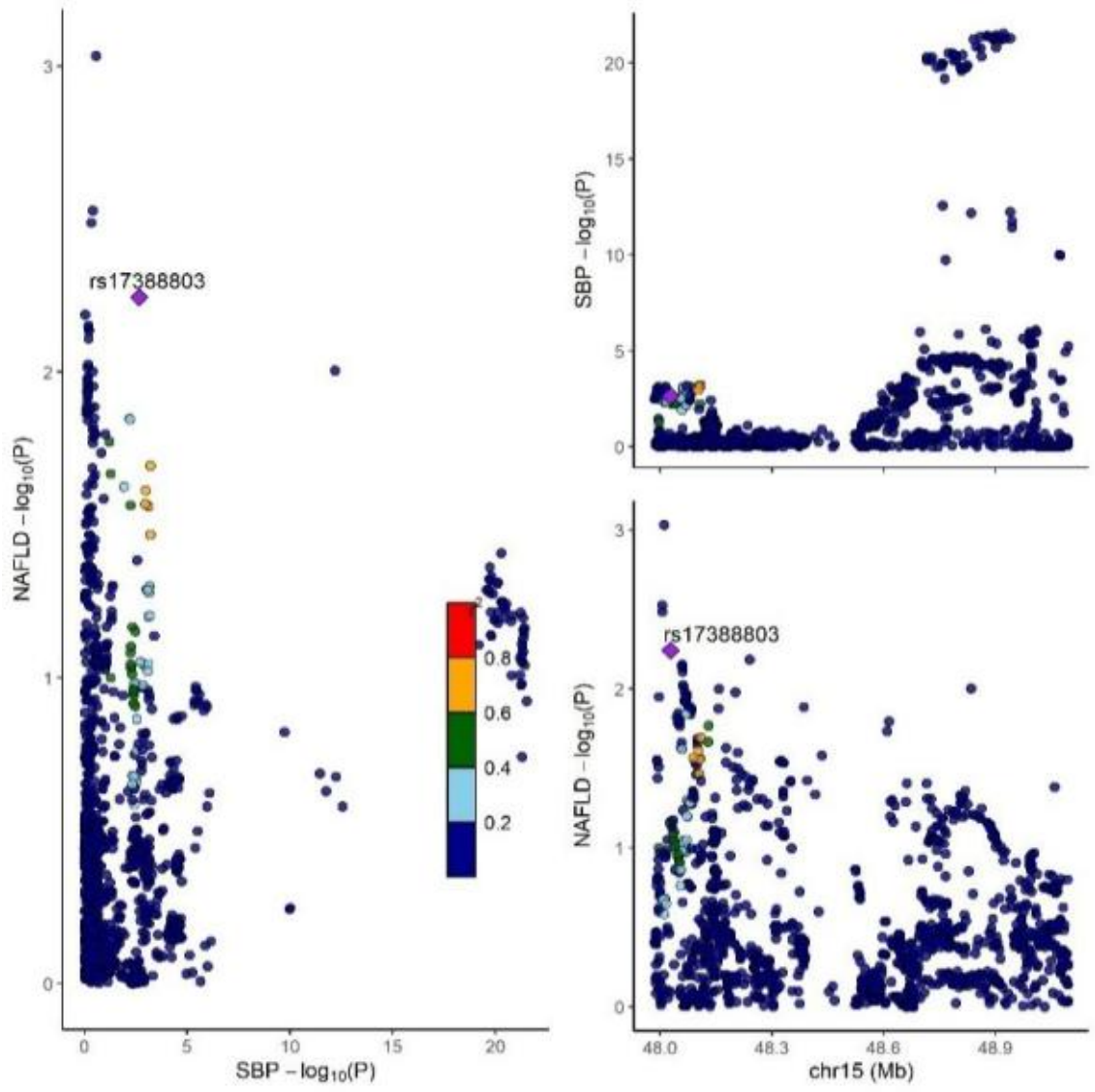

Supplement: Supplementary file 1 [file EMS218174-supplement-Supplemental_Publication_Material.pdf]
